# The photo-isomerization of the cyclononatetraenyl ligand and related rare earth complexes†

**DOI:** 10.1039/d4sc04767b

**Published:** 2024-10-18

**Authors:** Lucie Pedussaut, Nolwenn Mahieu, Camille Chartier, Thayalan Rajeshkumar, Maxime Tricoire, Iskander Douair, Nicolas Casaretto, Laurent Maron, Grégory Danoun, Grégory Nocton

**Affiliations:** a LCM, CNRS, Ecole Polytechnique, Institut Polytechnique de Paris Route de Saclay 91120 Palaiseau France gregory.nocton@polytechnique.edu gregory.danoun@polytechnique.edu; b LPCNO, UMR 5215, Université de Toulouse-CNRS, INSA, UPS Toulouse France

## Abstract

The cyclononatetraenyl (Cnt) ligand is a large monoanionic ligand. It is easily synthesized by ring expansion after cyclopropanation of the cyclooctatetraenyl (Cot) ligand. The Cnt ligand can be reported as the *cis*–*cis*–*cis*–*cis* (*cis*) isomer, where the aromatic ring is flat, and all carbon atoms form a homogenous ring, and as the *cis*–*cis*–*cis*–*trans* (*trans*) isomer, where one carbon places itself inside the ring. The isomerization from the *trans* to the *cis* form has been reported numerous times in previous articles, but no quantitative analysis has been proposed due to contradictory data. This article proposes a detailed analysis involving light to rationalize this intrigue concerning isomerization. A careful synthesis at low temperatures and with light protection yields the ligand in its *trans* form (Cnt-*trans*). The controlled photo-isomerization of the Cnt-*trans* ligand is reported herein. A series of divalent or trivalent rare earth complexes, (Cnt)_2_Sm, and (Cot)(Cnt)Ln (Ln = Y, La, Ce, Pr, Nd, Sm, Gd, Tb, Er, Ho), which synthesis, solid-state X-ray diffraction and solution ^1^H NMR and UV-visible measurements, have been revised according to the synthesis using the Cnt-*trans* ligand. The photo-isomerization of the (Cnt-*trans*)_2_Sm evolves to the intermediate (Cnt-*cis*)(Cnt-*trans*)Sm and the (Cnt-*cis*)_2_Sm complex as the thermodynamical product. The photoisomerization of the trivalent (Cot)(Cnt)Ln complexes highlights the formation of a photostationary state (PSS) after several minutes of irradiation, in which both Cnt-*trans* and Cnt-*cis* ligands are present. The ratio of these two forms varies according to metal and irradiation wavelength: low-energy wavelengths favor the *cis* isomer, and high-energy wavelengths favor the *trans* isomer. DFT and TD-DFT were performed to provide a tentative orbital explanation.

## Abstract 2

Reversible light isomerization in organometallic complexes of lanthanides: the low-energy wavelengths favor the *cis* isomer, and the high-energy wavelengths favor the *trans* isomer.

## Graphical abstract

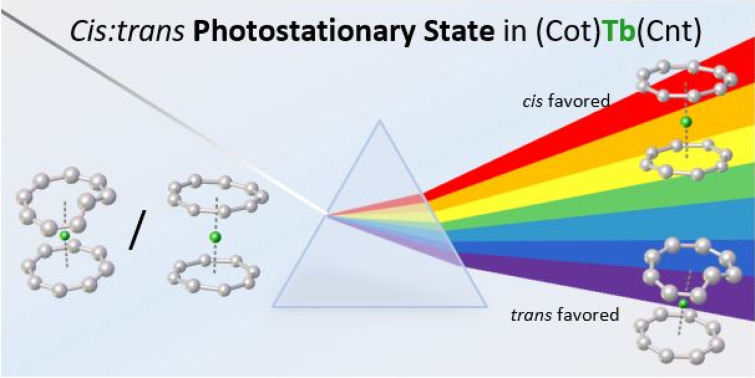

## Introduction

The cyclononatetraenyl ligand, C_9_H_9_^−^ – or Cnt^−^, was synthesized in 1963 by Katz and coworkers.^1,2^ The ligand is anionic and aromatic, with 10 π electrons. Synthetic attempts to aim for sandwich complexes have been made, but – except for notable multimetallic complexes with Pd,^3^ they mostly yielded to the re-organization of this large ligand to the bicyclic 1,5-dihydroindenyl.^4^ Only recently, this ligand has been used with larger ions, first barium, then 4f-elements, the size of which suits the Cnt ligand well.^5–7^

After the report of a series of complexes with Ba,^5^ the cyclononatetraenyl ligand has been used to develop bright luminescence dyes of Eu(ii) in the (Cnt)_2_Eu complex.^6^ Soon after, the family of these sandwich complexes was continued with the classical divalent lanthanides Sm, Yb, and Tm, showing linear isostructures along the series.^7^ Heteroleptic complexes of formulae (Cot)(Cnt)Ln (Ln = La, Ce, Nd, Sm, Tb, Dy, Er, Ho, Tm, and Lu) complexes were made,^8–10^ accommodating the trivalent oxidation state, while the analogs to the historical (Cp)_3_Ln complexes with Cnt, the (Cnt)_3_Ln (Ln = Y, Gd, Tb, Dy, Ho, Er, Tm) complexes were also reported in recent years.^11^

Because of their potential for developing high-performance molecular magnets, lanthanide-based molecular compounds have developed strongly in recent years.^12–19^ Several sandwich complexes in the divalent and trivalent states containing the Cot and Cnt ligands have been employed.^5–9,20–23^ These have given rise to remarkable fundamental chemistry, describing several objects with fascinating structures.^10^

The Cnt ligand is a labile ligand easily displaced by competing coordinating solvents. Notably, it has been shown that the ligand bends and changes its coordination mode when complexed with lanthanide ions of different sizes.^9^ This characteristic has also been used to build complexes, modulating their physical properties by using solvents or controlling the ligand isomerization.^7^ For example, Vitova *et al.* recently demonstrated the photo-modulation of bond covalency in the (Cnt)_2_Sm complex.^24^

**Fig. 1:**
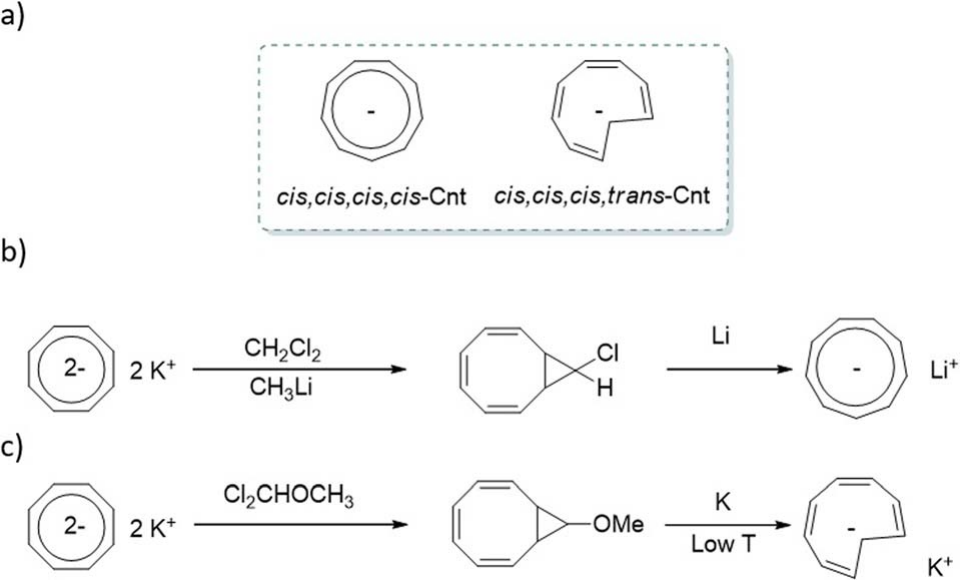
(a) Isomers of the cyclononatetraenyl ligand, (b) original synthetic paths by Lalancette and Benson^2^ and (c) synthetic path favoring the trans isomer.^28^

In addition to its coordination features, the Cnt ligand can be obtained as two different isomers (Fig. 1a). The first is the isomer in its usual aromatic form, with a planar symmetrical ligand and similar internal C–C distances. It is named the *cis*–*cis*–*cis*–*cis* (*cis*) isomer to recall the relative orientation of the alternating four double bonds originating from the 10 π-electrons. Its isomer is described by the reversal of a carbon atom in the ring, which induces the presence of a *trans* bond, hence the name *cis*–*cis*–*cis*–*trans* (*trans*). Aromaticity is limited to the six π-electrons in the ring, while the charge is localized on the reversed carbon. As a result, the NICS(0) value increases from the Cnt-*cis* ligand to the Cnt-*trans* from −15.8 to −20.3 in agreement with more localized aromaticity in the plane,^25^ while the NICS(1) has similar values of −13.7 and – 13.8 for Cnt-*cis* and Cnt-*trans*, respectively. The presence of both isomers has been described in previous work for the sole ligand and its metallic complexes without further explanation.^7,9,26^ For example, some of us used the difference in solubility of the Cnt-*trans* compared to the Cnt*-cis* to improve the yield of the (Cnt)_2_Ln complexes.^7^

The question of the isomers returns to the original ligand synthesis. Initially, the synthesis was performed with dichloromethane or dichloromethyl methyl ether, leading to the *cis* isomer (Fig. 1b).^27^ A modification in the synthetic pathway favors the second isomer (*trans*) (Fig. 1c).^28^ At low temperatures, the methoxide group of the bicycle is favored in the anti-position. When electrons are injected into the system, it is reported that the anion radical is formed within the 8-member ring, leading to a conrotatory ring opening mechanism favoring the *trans* isomer.^29^ A comprehensive study by Boche in 1978 brought much information on the Cnt ligand and its isomerization.^26^ However, several questions remained: one particularly caught our attention. The isomerization was reported from the *trans* form to the *cis* form, implying that the *cis* form is the most thermodynamically stable. Additionally, the process was reported to be faster at 60 °C in THF than at 100 °C in the same solvent. This mention implies a counter-thermodynamic isomerization process, which differs from typical topoisomerization. Boche's comprehensive work is unquestionable, and we've been able to reproduce his results.

In addition to this intriguing result, some of us and Roesky have noted the presence of an isomerization phenomenon contrary to the thermodynamics of ligand isomerization. When the symmetrical (Cot)(Cnt)Tb complex (starting from the Cnt-*cis* ligand) is left at room temperature, the complex evolves towards the *trans* form.^9^ A fast mention in Boche's seminal work about a possible light-induced isomerization attracted our attention since it would lead to original photoswitch systems.^26^

Photochromism is a phenomenon in which molecules can undergo a photochemical reaction to switch between two stable isomers showing different structures or properties. Azobenzene is a prime example of a classical photoswitch.^30,31^ Many related examples exist, and photochromic properties have been used as a light trigger switch in polymers,^32^ surfaced materials, supramolecular chemistry, catalysis,^7^ and many biological applications.^33^ Complexes bearing azobenzene and related scaffolds are also reported to possess photo-switch behavior.^34,35^ One particularity of this motif is the possibility of switching from the *E* to the *Z* form and the reversal using a different light energy. Sometimes, the transfer from one to another isomer is incomplete, and several *E* : *Z* ratios (PSS: photo stationary states) are reported as the stable form upon light irradiation.^36^

**Fig. 2:**
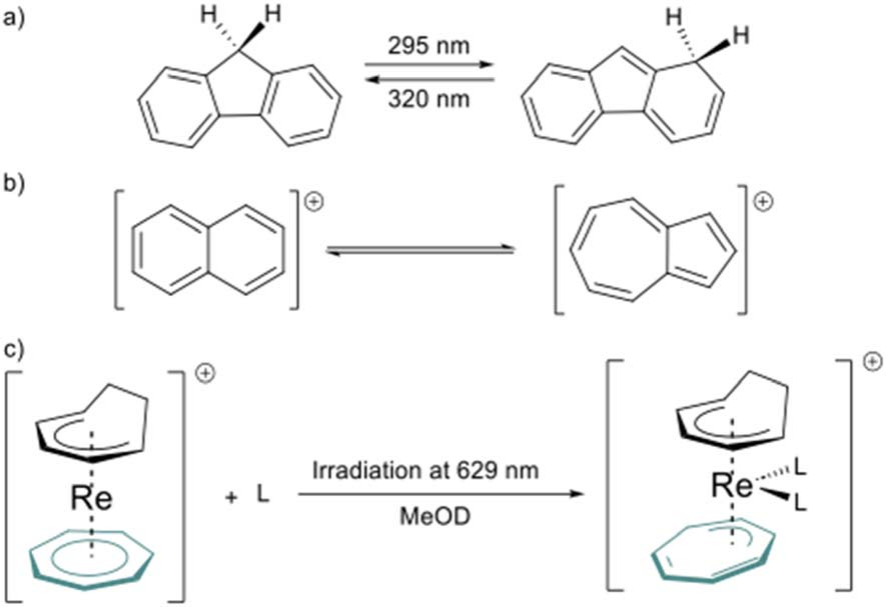
a) Photo-isomerization in aromatic hydrocarbons, 9*H*-fluorene to 1*H*-fluorene, (b) interconversion between the naphthalene and the azulene cation, and (c) photo-induced hapticity switch in rhenium sandwich complexes.

However, this type of photo-induced isomerization is rare in aromatic hydrocarbon ligands. To our knowledge, the only instances reported of the photoinduced isomerization of C–H aromatics ligands are the isomerization of 9*H*-fluorene,^37^ naphthalene, and azulene cations.^38^ Another similar example is the light-induced hapticity switch of the C_7_H_7_ ligand in a Re sandwich complex (Fig. 2).^39^

Here, we report the synthesis and characterization of a series of rare earth complexes with the Cnt-*trans* ligand and study the photo conversion into the *cis* isomer. The finding particularly highlights the formation of a photo-stationary state (PSS), in which the *cis* : *trans* ratio evolves with the wavelength used and with the identity of the lanthanide cation. The study finalizes the rationalization of many observations made over the last 50 years on the cyclononatetraenyl ligand and opens various possibilities to control the *cis* : *trans* ratio.

## Results and discussion

### Synthesis and solid-state studies

#### The cyclononatetraenyl ligand

The ligand can be synthesized and crystalized as a mixture of isomers.^7^ When synthesized from the chloride substrate, which is present in a mixture of diastereoisomers, the Cnt ligand affords a mixture of both *trans* and *cis* isomers (Fig. 1a).^27^ On the other hand, when the methoxide substrate, which is present as only one diastereoisomer, is used as a starting material, the major form is the *trans* isomer. Because isomerization from the *trans* to the *cis* form occurs readily at room temperature, it is convenient to avoid spontaneous isomerization by keeping the temperature at 233 K to yield the Cnt ligand in 32% yield in 80 : 20 *trans* to *cis* ratio. This ratio can be brought to 95 : 5 if the reaction and the work-up are protected from light.

#### The (Cnt)_2_Ln complexes (1)

In the seminal work performed by some of us,^7^ the formation of linear sandwich complexes was described with four divalent lanthanide ions, *e.g.* Sm, Eu, Yb, and Tm. However, because the Cnt ligand was not obtained in the pure *trans* form, the complexation resulted in mixtures of three isomers, the homoleptic (Cnt-*cis*)_2_Ln, (Cnt-*trans*)_2_Ln complexes and the heteroleptic (Cnt-*cis*)(Cnt-*trans*)Ln complexes, as evidenced through solution ^1^H NMR for the Sm mixture. Using the lability of the Cnt ligand, the addition of coordinating solvents led to the *trans*-to-*cis* easy isomerization. It allowed the solid-state characterization of the pure (Cnt-*cis*)_2_Ln (Ln = Sm, Eu, Tm, Yb).^7^

Thanks to the improved method to yield the KCnt ligand in a very high ratio of *trans*, which is reported herein, and also knowing that light causes problems in the reproducibility of the syntheses, the (Cnt-*trans*)_2_Ln were synthesized in the dark and in the absence of any coordinating solvent. The reaction vessel was protected by aluminum foil, and the light of the glovebox was switched off. The reaction between SmI_2_ and KCnt-*trans* was performed in toluene overnight. After filtration, the solvent was evaporated, and the resulting black powder was analyzed as pure (Cnt-*trans*)_2_Sm (1-*trans*) by ^1^H NMR (Fig. S2†). However, our attempts to obtain X-ray suitable single crystals of the pure *trans* compound failed. Despite our efforts to protect the crystallization from light, the few structurally characterized crystals always contained a considerable amount of the *cis* isomer in the solid state (82%). This is likely due to a slow isomerization process occurring even at 233 K over the time needed to grow the crystals and the solubility difference between 1-*trans* and 1-*cis* isomers. The latter being less soluble leads to an overestimation of the *cis* : *trans* ratio from the XRD perspective. This was confirmed by taking the NMR of the bulk material obtained after the crystallization step (See Fig. S3†) and revealed the presence of 49% of pure 1-*trans* and only 11% or conversion to pure 1-*cis*, the leftover 40% being the half-isomerized 1-*cis*–*trans* form.

The 1-*cis* isomer was previously reported in the literature in *P*2_1_/*n* space group.^7^1-*trans* crystallizes in the same space group. In our hands, because of the fast isomerization process while crystallizing, the crystals were found mostly in the *cis* form. Only one Cnt ring was modeled as the superposition of the two eclipsed Cnt isomers (18% *trans*). The carbon atom in the *trans* position crystalizes out of the plane, and is closer to the metal center. All C–C bonds are similar between the *trans* and *cis* isomers (Table 1).

**Table:** Metric parameters of KCnt(OEt_2_), 1-*trans*, 1-*cis*

	KCnt(OEt_2_)^a^	1-*trans*	1-*cis*^a^
C–C all average	1.393 (4)	1.381 (15)	1.388 (13)
C–C(Cnt) inside	1.361 (5)	1.377 (18)	—
C–C(Cnt) ring average	1.402 (4)	1.382 (15)	1.388 (13)
Ln–C(Cnt) inside	—	2.72 (7)	—
Ln–C(Cnt) all average	—	2.85 (4)	2.742 (8)

a Data taken from known literature.^7^

#### The (Cot)(Cnt)Ln complexes (2)

**Fig. 3:**
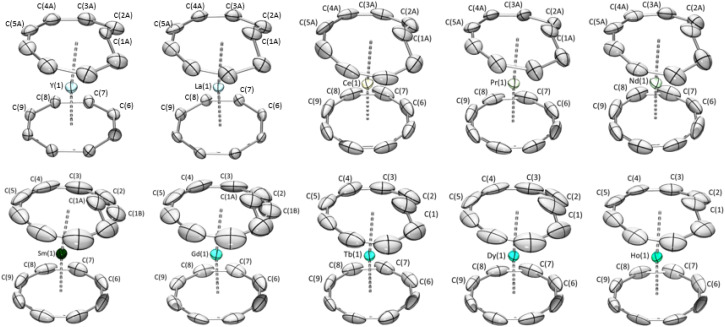
X-ray crystal structures of 2-Y to 2-Ho with the Cnt ligand in *trans*.

Additionally, a better understanding of the impact of light on the speciation of the Cnt ligand (*vide infra*) also allowed the selective isolation of the (Cot)(Cnt)Ln complexes in the *trans* form (2-Ln-*trans*) over the (Cot)(Cnt)Ln complexes in the *cis* form (2-Ln-*cis*) and *vice versa*. As described above, for the former, the synthesis involves protecting all the steps from light with aluminum foil and using a non-coordinating solvent. For the latter, the complexation is performed under ambient light with the addition of acetonitrile to promote the isomerization of the ligand during the synthesis. Still, the solids are protected from light to avoid *cis* to *trans* isomerization over time. In both cases, ^1^H NMR and Single Crystal XRD were used to assess the obtained *trans* : *cis* ratios in the solid state. It was previously reported that the solid-state structure of the (Cot)(Cnt)Ln complexes (with the Cnt-*cis* ligand) could be modeled in two different space groups.^8,9,40^ For La, Ce, Nd, Sm, and Tb, the symmetry of the structure with the metallic ion localized on a given symmetry position led to the resolution in the *Pnma* space group. For the other lanthanide ions placed after Tb in the series, the *P*2_1_/*n* space group was used. Thanks to the new method of synthesis of the KCnt ligand, the *trans* : *cis* ratio is higher. However, despite all our precautions to avoid light during the syntheses, the crystals that were obtained almost all showed a *trans* : *cis* ratio from 100% in the Tb, Dy, and Ho complexes to 71% and 69% in Gd and Sm, respectively and 58%, 55%, 50%, 25%, 73% in Nd, Pr, Ce, La and Y respectively (Fig. 3). In comparison to the previous work, all complexes crystallized in the *Pnma* space group and the hapticity switch of the Cnt ligand in 2-Dy-*cis*, 2-Ho-*cis* and 2-Y-*cis* complexes was not observed for their *trans* form. In this space group, only half of the complex needs to be modeled, and the other half will be constructed through the symmetry operation of this group.

Over the series, three types of behaviors were observed and modeled. First, for the early lanthanide ions (La, Ce, Pr, and Nd), the Cnt ring was modeled as the superposition of a Cnt-*cis* ligand and a Cnt-*trans* ligand in eclipsed positions, as represented in Fig. S35–39.† The occupation number in each ring was linked to the overall *cis* : *trans* ratio. For Sm and Gd, one carbon atom of the Cnt ligand presented a significant disorder with two favored positions: one following the ring and one inside it (Fig. S40 and 41†). The occupation number of each position can be used as a measure of the solid-state *cis* : *trans* ratio. Finally, for Tb, Dy, and Ho, the new synthesis led to the crystallization of purely *trans* complexes, and no disorder was modeled.

The distances between the lanthanide centers and the carbon atoms of the Cot ligand follow the radius decrease along the series of the Ln with values of 2.695 (12) Å for the La and 2.55 (7) Å for Ho in the *cis* complexes and 2.694 (4) Å for the Ce and 2.478 (7) Å for Ho for the *trans* complexes (Table 2). A similar trend is observed with the Cnt-*cis* and Cnt-*trans* as well as the Ln–C(1) of the carbon atom inside the ring, with notable singularities observed for the Ho Cnt-*cis* due to the modification of the coordination mode described in earlier work.^9^

Metric parameters of 2-Y to 2-Ho. Distances in Å and angles in degree

**Table:** 

Ln/Cnt-*trans*	2-Y-*trans*	2-La-*trans*	2-Ce-*trans*	2-Pr-*trans*	2-Nd-*trans*	2-Sm-*trans*	2-Gd-*trans*	2-Tb-*trans*	2-Dy-*trans*	2-Ho-*trans*
Ln–C(Cot) average	2.542 (8)	2.694 (4)	2.654 (4)	2.636 (6)	2.625 (9)	2.595 (6)	2.570 (7)	2.552 (10)	2.541 (5)	2.478 (7)
Ln–C(Cnt–C8) average	2.806 (9)	2.905 (8)	2.881 (15)	2.86 (2)	2.87 (2)	2.833 (8)	2.826 (9)	2.818 (11)	2.802 (8)	2.792 (9)
Ln–C(Cnt) inside	2.657 (10)	2.851 (16)	2.750 (12)	2.749 (15)	2.79 (2)	2.687 (13)	2.703 (17)	2.664 (16)	2.660 (9)	2.673 (11)
Ln–C(Cnt) all average	2.776 (9)	2.894 (10)	2.85 (1)	2.84 (2)	2.85 (2)	2.804 (9)	2.801 (11)	2.787 (13)	2.774 (8)	2.768 (9)

a Data taken from known literature.^9^

**Table:** 

Ln/Cnt-*cis*	2-Y-*cis*	2-La-*cis*^40^	2-Ce-*cis*^40^	2-Pr-*cis*	2-Nd-*cis*^8^	2-Sm-*cis*^8^	2-Gd-*cis*	2-Tb-*cis*^a^	2-Dy-*cis*^a^	2-Ho-*cis*^a^
Ln–C(Cot) average	2.54 (2)	2.695 (12)	2.676 (8)	2.65 (1)	2.65 (1)	2.624 (7)	2.609 (16)	2.58 (2)	2.58 (4)	2.55 (7)
Ln–C(Cnt) average	2.75 (2)	2.944 (15)	2.91 (1)	2.90 (2)	2.88 (1)	2.860 (7)	2.82 (2)	2.82 (3)	2.81 (10)	2.93 (33)
(Cot)^(Cnt) (plane)	21.26	3.69	3.75	3.86	3.62	3.02	2.53	4.80	12.5	26.7
Cot–Ln–Cnt (cent.)	170.67	176.86	177.01	177.39	176.70	177.17	177.34	177.4	172.0	169.6

At this stage, it is essential to note that those solid-state studies and descriptions are only representative of the part of the mixture that did crystalize. Remarkably, the *cis* and the *trans* isomers possess different solubility, with the Cnt-*trans* being more soluble. As such, the observed ratio of isomers in the solid state is necessarily biased. Thus, a description of the speciation in the solution will be instrumental in accessing a complete characterization of the system.

### Solutions studies

#### The cyclononatetraenyl ligand

**Fig. 4:**
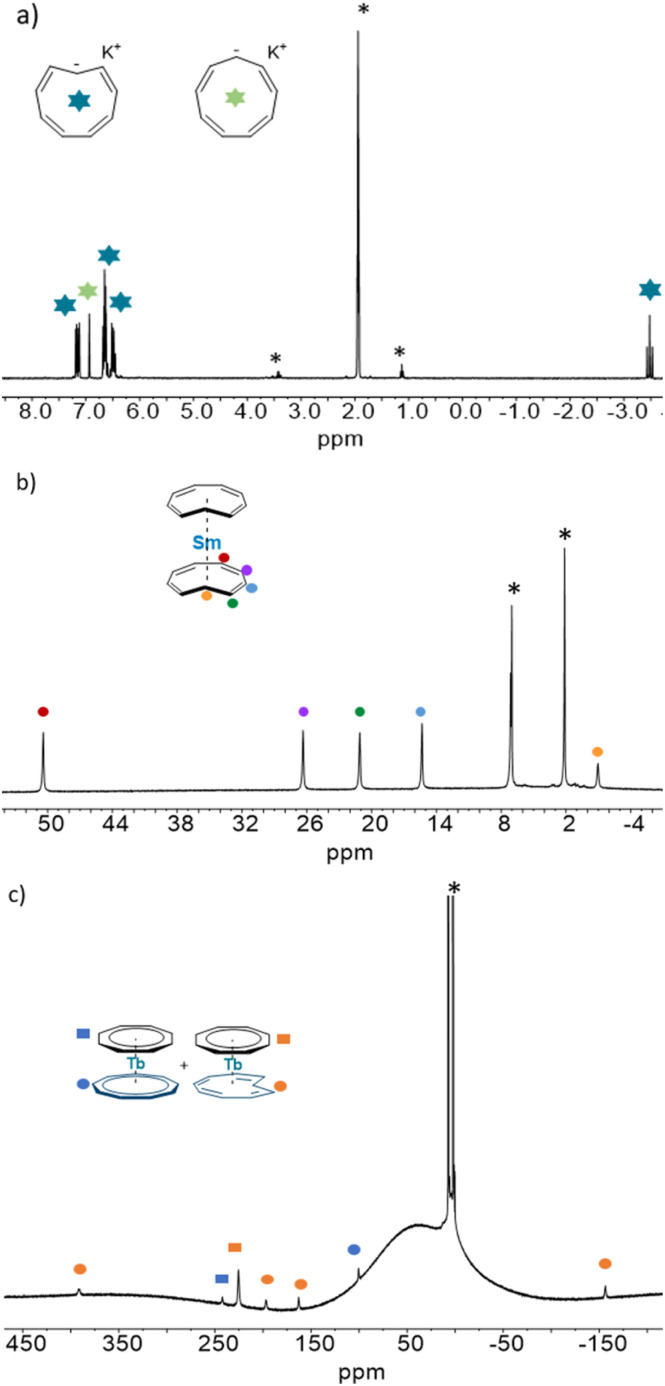
^1^H NMR of the (a) *cis*/*trans* KC_9_H_9_, of (b) 1-*trans* and of (c) 2-Tb-*trans*.

In their pioneering work on the Cnt molecule, the group of Boche heavily relied on the use of solution ^1^H NMR data to study both the isomerization and the topo-isomerization processes.^26,41^ Due to the difference in symmetry, the two isomers have very different signatures: the aromatic Cnt-*cis* ligand possesses 9 identical protons, and the Cnt-*trans* ligand only possesses an axial symmetry with 5 groups of protons as displayed in Fig. 4. Particularly, the signature of the proton attached to the C(1) carbon is significantly upfielded at −3.48 ppm. Thus, the ^1^H data allow the quantification of both isomers.

#### The (Cnt)_2_Ln complexes (1)

The ^1^H NMR data of the mixture of isomers 1-*cis*, 1-*trans*–*cis* and 1-*trans* have been reported in a previous work.^7^ The 1-*trans* shows five ^1^H NMR signals in a 4 : 4 : 4 : 4 : 2 ratio in agreement with equivalent Cnt ligands and a *C*_2v_ symmetry in solution. The chemical shifts have been attributed by a COSY experiment and reflect the orientation of the principal axis of symmetry along the *C*_2_ axis with a negative shift for the proton attached to the C(1) carbon (−0.95 ppm) and a larger chemical shift for the proton in the farthest position in the aromatic backbone of the Cnt ligand (50.4 ppm, red dots in Fig. 4).^42^

#### The (Cot)(Cnt)Ln complexes (2)

The ^1^H NMR data was used in the preliminary discovery that 2-Tb-*cis* would spontaneously isomerize at room temperature from the Cnt-*cis to* the Cnt-*trans*.^9^ As such, the ^1^H NMR of 2-Tb-*trans* evolved from the two-peak pattern corresponding to a heteroleptic compound to a six-peak pattern, in good agreement with a loss of symmetry (Fig. 4). The appearance of the Cnt-*trans* was confirmed through XRD.

The assignment of both isomers was reproduced for all of the compounds and is presented in ESI Fig. S11–S20.† Due to the paramagnetic nature of lanthanide ions, in our hands, only four signals coming from the Cnt-*trans* could be reliably identified in a broad spectral window. The proton attached to the C(1) carbon may be significantly influenced by the Ln ion, which disallowed its observation.

### Photoisomerization of the cyclononatetraenyl ligand and 1

With a reliable method to correctly identify both isomers, the presumed photo-isomerization processes were studied for the Cnt ligand itself and all complexes reported in this article. Solution mixtures were irradiated directly into the NMR tubes at different wavelengths, and then the ratios were quantified by ^1^H NMR.

With the syntheses of the cis compounds at hands,^7–9,40^ but also several of the *trans* compounds (see discussion above), UV-visible spectra of both isomers were recorded (see Fig. S47 to S59†) for all the compounds, except for the 1-*trans*–*cis*, which could not be isolated. In 2, Ln = Er, Tm, Lu, only the *cis* isomers were synthesized; thus, the spectrum of the PSS was generated under 370 nm irradiation before recording.

Under a 390 nm irradiation, the Cnt ligand isomerizes quickly from the Cnt-*trans* to the Cnt-*cis*. Thus, irradiation at a lower energy (427 nm) was used to monitor the process by ^1^H NMR (Fig. S62†). The complete conversion was noted after 5 min of irradiation. The apparent rate of decrease of the KCnt-*trans* in favor of the *cis* isomer displays a pseudo-first-order reaction rate. However, a complete photochemical analysis was not performed. The Cnt-*trans* shows two major absorption bands in the UV region at 345 nm and 275 nm with absorption coefficients of 5000 cm^−1^ M^−1^ and 45 000 cm^−1^ M^−1^, respectively. Meanwhile, the *cis* isomer displays two different absorption bands at 323 nm and 252 nm with absorption coefficients of 6000 cm^−1^ M^−1^ and 75 000 cm^−1^ M^−1^, respectively (Fig. 7). The strong absorption of the Cnt-*cis* isomer is in agreement with a larger HOMO–LUMO gap brought by the symmetry of the π system of the ligand, and thus the π to π* transfer requires more energy. Accordingly, the similar transition is less intense for the Cnt-*trans* ligand and is red-shifted. This energetic situation agrees with a *trans*-to-*cis* isomerization process, which occurs at energies lower than 300 nm, and the reverse process would need higher energy.^26,43^

Under a 427 nm irradiation, the 1-*trans* isomerizes to the 1-*cis* using the 1-*trans*–*cis* as an intermediate within 30 min (Fig. S62†). The apparent rates of decrease of the 1-*trans* to the 1-*trans*–*cis* and the one from the 1-*trans*–*cis* to the 1-*cis* both display a pseudo-first-order reaction rate.

### Photoisomerization of 2 and PSS

Because of the paramagnetic nature of the lanthanide ions and the superimposition of the two isomers' signals, the ^1^H NMR analyses were not possible for 2-Ln (Pr, Nd, Gd, Ho, Er, and Tm). However, for those where the integration is possible, irradiation at different wavelengths led to different ratios of Cnt-*trans vs.* Cnt-*cis*. Kinetic studies performed on the Sm complex (2-Sm) showed that the proper ratio is attained after 30 min and then does not change with time under irradiation. This phenomenon is in line with a PSS (photo-stationary state). In the experiments, the irradiation time was thus determined to ensure that the PSS was obtained in all cases. The PSS at different wavelengths can be reached either from the *cis* or from the *trans* complexes that can be obtained from different synthetic methods.^8,40^

**Fig. 5:**
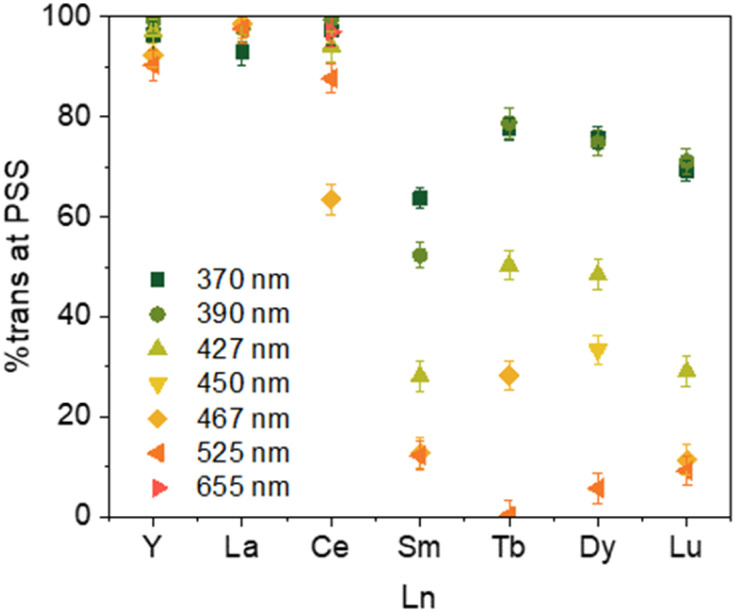
Ratio of 2-Ln at PSS at 370 nm irradiation up to 525 nm depending on the Ln (Y–Ce, Sm, Tb, Dy, Lu).

The PSS reached for the 2-Ln complexes are gathered in Table 3. Along the series, two main behaviors can be observed as illustrated in Fig. 5. First, for early La (2-La) and Ce (2-Ce) as well as Y (2-Y), the modification of the wavelength has only a modest impact on the PSS and high *trans* : *cis* ratios are favored. For example, in La (2-La), the PSS ratio evolves from 93% to 99% at 370 nm and 440 nm, respectively, the Ce (2-Ce), from 97% to 64% at 370 nm and 467 nm, respectively, and the Y (2-Y) from 96% to 90% at 370 nm and 525 nm, respectively.

**Table:** Summary of *trans* ratios obtained from 2-Y to 2-Lu during the synthesis by ^1^H NMR and XRD and the ratio at PSS

	2-Y	2-La	2-Ce	2-Sm	2-Tb	2-Dy	2-Lu
NMR	61%	55%	79%	89%	87%	75%	0%
XRD	73%	25%	50%	69%	100%	100%	0%
PSS 370 nm	96%	93%	97%	64%	78%	76%	69%
PSS 390 nm	99%	98%	100%	52%	79%	75%	71%
PSS 427 nm	97%	99%	94%	28%	50%	48%	29%
PSS 440 nm	95%	99%	86%	23%	39%	—	20%
PSS 450 nm	—	—	—	—	—	33%	—
PSS 467 nm	92%	99%	64%	13%	28%	—	11%
PSS 525 nm	90%	98%	88%	12%	1%	6%	9%

**Fig. 6:**
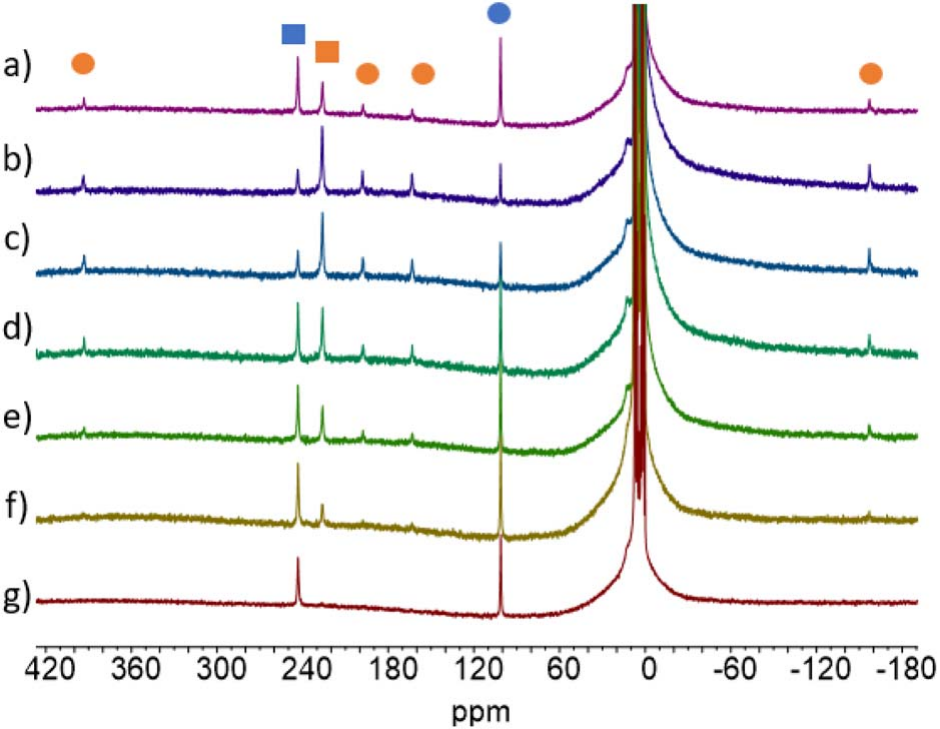
^1^H NMR of 2-Tb at different PPS (after 30 min of irradiation) depending on the wavelength of the irradiation light used (a) *t* = 0, (b) *λ* = 370 nm, (c) *λ* = 390 nm, (d) *λ* = 427 nm, (e) *λ* = 467 nm, (f) *λ* = 490 nm, (g) *λ* = 525 nm. Blue is for 2-*cis* and orange for 2-*trans*. Squares are for the Cot ligand, and circles are for the Cnt ligand.

The situation is different for Sm (2-Sm), Tb (2-Tb), Dy (2-Dy), and Lu (2-Lu) for which the ratios are significantly impacted by the irradiation wavelengths as shown in Fig. 5. For instance, for Tb (2-Tb), the *trans* : *cis* ratio of the PSS at 370 nm is 78 : 22 while it drops to almost zero at 525 nm as shown in Fig. 6. A similar trend is observed for Sm (2-Sm) with values varying from 64% to 12% at 370 nm and 525 nm, respectively, for Dy (2-Dy) with values ranging from 76% to 6% at 370 nm and 525 nm, respectively, and for Lu (2-Lu) with values ranging from 69% to 9% at 370 nm and 525 nm, respectively.

To better understand this difference in speciation, UV-visible absorption spectra of 2 were recorded (Fig. S47–S59†). Similarly to what is known in the azobenzene PSS, it is notable that the overlapping of the absorption bands of both isomers does not favor an effective shift to one or the other isomers with a high PSS ratio upon light irradiation.^43^Fig. 8 reports the energy of the transitions observed before irradiation.

**Fig. 7:**
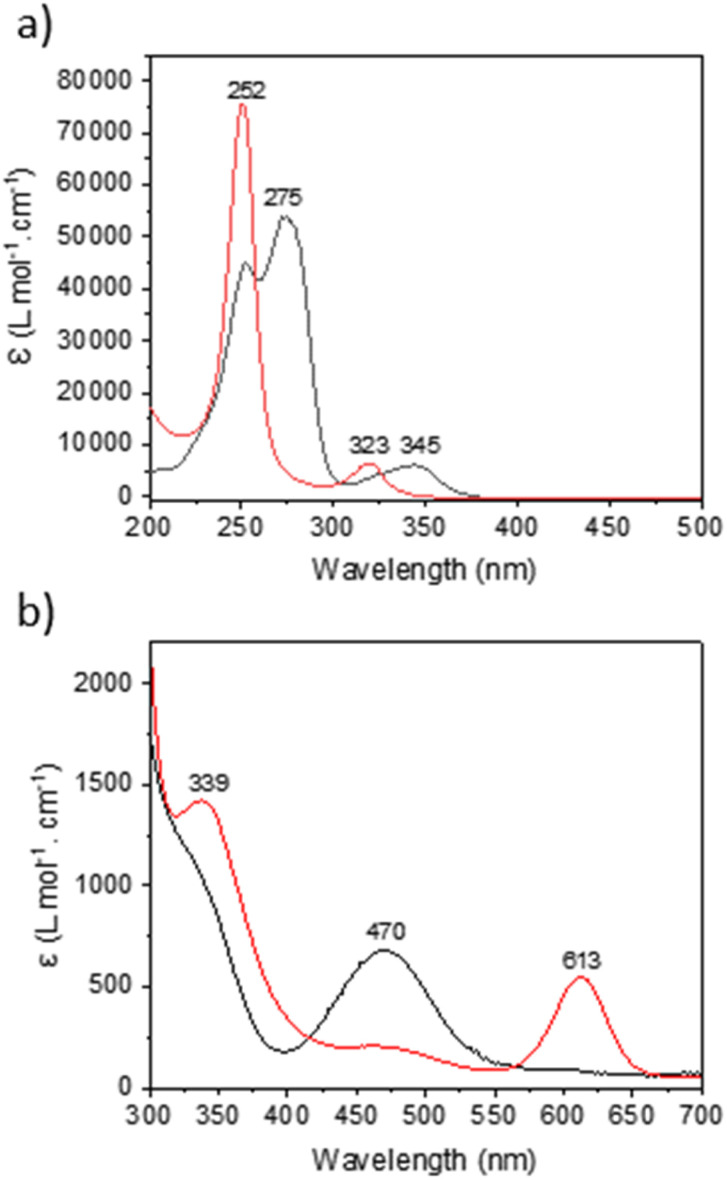
UV-visible of (a) KCnt-*trans* (black) and KCnt-*cis* (red) and (b) *trans* (black) and *cis* (red) complexes of 2-Ce.

**Fig. 8:**
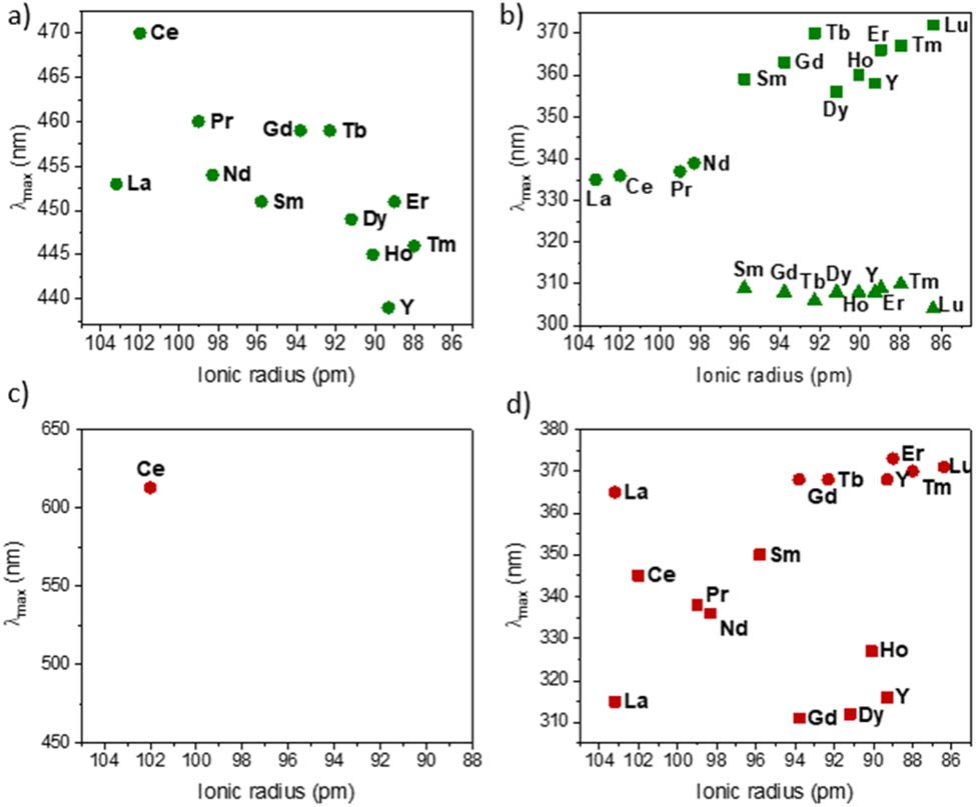
Evolution of the maximum absorption of 2-Ln (the *cis*/*trans* ratio is given in Table 3) complexes wavelength around (a) 450 nm and (b) 300–370 nm and of 2-Ln-*cis* (made from the methodology proposed in literature^9,40^ around) (c) 400–650 nm and (d) 300–380 nm.

Similar to the KCnt ligand, 2 exhibits a strong absorption at low energy, but two maxima can be observed within the 300 to 370 nm region for the Y and late Ln (Sm–Lu). In contrast, only one can be observed for the early Ln (La–Nd) (Fig. 8). Additionally, 2-Ce-*cis* displays a third absorption band at 625 nm (800 cm^−1^ M^−1^) (Fig. 7b). As an additional note, it is important to stress that 2-Ce and 2-Tb were reported to be luminescent,^40^ but only when THF molecules were coordinated, so in the conditions reported here, no emitted light can interfere with the isomerization process.

Based on the sole data we have in our hands, the rationalization of the data is not simple. First, we observe that the PSS of 2-Y and 2-La are unaffected by the different wavelengths. No matter what energy is used, the *trans* form is the most stable one under light irradiation. This is notable because the synthesis of the pure 2-Y-*trans* and 2-La-*trans* was not possible, and either in solution or in the solid-state, a significant amount of the *cis* material is made (See Table 3).

For Sm, Tb, Dy, and Lu, the PSS varies strongly depending on the wavelength used (See Fig. 5). The differences in the UV spectra compared to those of La, Y are minimal. One rationalization is that light brings enough energy to populate a transition state, which then relaxes on either the *trans* form or the *cis* one. Thus, when high energy is used, this allows for relaxing to the *trans* form, while without enough energy, the barrier to relax to the *trans* form is not crossed, and thus, the *trans* : *cis* ratio remains small. This would mean that the barrier is lower in La and Y than in Sm, Tb, Dy, and Lu, which are similar.

A second note is related to the absorption cross-section of the *cis* or the *trans* form at different wavelengths. While the *cis* compounds prepared with the published methodology^9,40^ have almost no absorption above 450 nm, the compounds with a higher *trans* : *cis* ratio do (Fig. S60†). Thus, at lower energy wavelengths, only the *trans* form absorbs, which explains the high *trans* : *cis* ratio for Sm, Tb and Lu. However, this does not explain the low *trans* : *cis* ratio in Y and La (Fig. S45 and S46†). An interesting case is that of 2-Ce. 2-Ce-*cis* has one transition in the visible spectrum centered at 613 nm. At this value, the absorption of 2-Ce-*trans* is low. On the other hand, 2-Ce-*trans* has an absorption maximum at 470 nm and 2-Ce-*cis* has a weaker absorption at this wavelength (Fig. 7b). Following the ratio of *trans* : *cis* depending upon the energy of the wavelength is therefore informative. At high energy, the *trans* : *cis* ratio is high, similar to all compounds. At 467 nm, the *trans* : *cis* decreases to around 60%; following the case of Sm, Tb, Dy, and Lu, but then re-increases at lower energy (525, 88%, and 655 nm, 97%). This shows that the absorption cross-section of the *cis* form strongly influences the *trans* : *cis* ratio. Yet, compared to the KCnt ligand or with 1, where the photo-isomerization converts the *trans* to the *cis*, the reason for the stabilization at high light energies of the *trans* form compared to the *cis* one in 2-Ln was not straightforward. This is the reason why we have turned to theoretical computations.

### DFT and TD-DFT calculations

To gain some insights into the electronic structure of 2, both in the *cis* and in the *trans* forms, DFT calculations were carried out (B3PW91, including dispersion corrections). The structures were optimized for Ce (2-Ce) and Sm (2-Sm), and in both cases, either the Cnt-*cis* or Cnt-*trans* ligands were considered. Interestingly, the two structures are found to be stable minima for both lanthanide metals. However, in the case of Ce (2-Ce), the 2-Ce-*cis* is found to be slightly more stable than the *tran*s-one (3.7 kcal mol^−1^), while for Sm (2-Sm), the *cis* isomer is significantly more stable than the *trans* (6.9 kcal mol^−1^). These energy differences could explain the difference observed in the X-ray where Ce is a superposition of the two structures. In contrast, for Sm, only the disorder of one carbon is observed. In all cases, the optimized distances compare well with the experimental ones (see Tables S24 and S35 in ESI†). The fact that the 2-Ln-*cis* complexes are more stable than the 2-Ln-*trans* well agrees with the thermodynamical stability of 2-Ln-*cis* observed in previous studies.^7–9,11^

Molecular and Natural Bonding Orbitals analysis (NBO) calculations were carried out on these complexes. The unpaired spin density appears to be mainly located at the lanthanide centers (Ce: 1.0, Sm: 5.5). However, these values imply the bonding mode in the two metals. Indeed, for Ce, the unpaired spin density is what is expected for a Ce(iii) complex with a (Cot)^2−^ ligand and, therefore, a (Cnt)^1−^. This is reflected in the Frontier Orbitals (see Fig. S88†), where the SOMO is a pure f-orbital, while the doubly occupied HOMO-1 and HOMO-2 describe the Ce–Cot interaction. The Canonical Molecular Orbital (CMO) analysis of NBO 6.0 shows that in these two orbitals, it is a donation from Cot to an empty mainly 5d (90%, 10% 4f) orbital of Ce. For the Sm, the expected unpaired spin density for a Sm(iii) is 5, while a value of 5.5 is found, which would indicate the presence of a 2.5 oxidation at Sm. This would imply the formation of a (Cot)^1.5−^, which was proposed in the case of the cerocene complex.^44–46^ The latter situation is also proposed since 5 unpaired f electrons are found in the MOs as well as 4 MOs to describe the Sm–Ligand interactions. The CMO analysis clearly indicates that the two Cnt–Sm interactions are pure donation from ligand to metal, while for the Cot–Sm interaction, one is donation from ligand to metal, and the second one is donation from Sm to the Cot ligand (Table S41 and S45†).

**Fig. 9:**
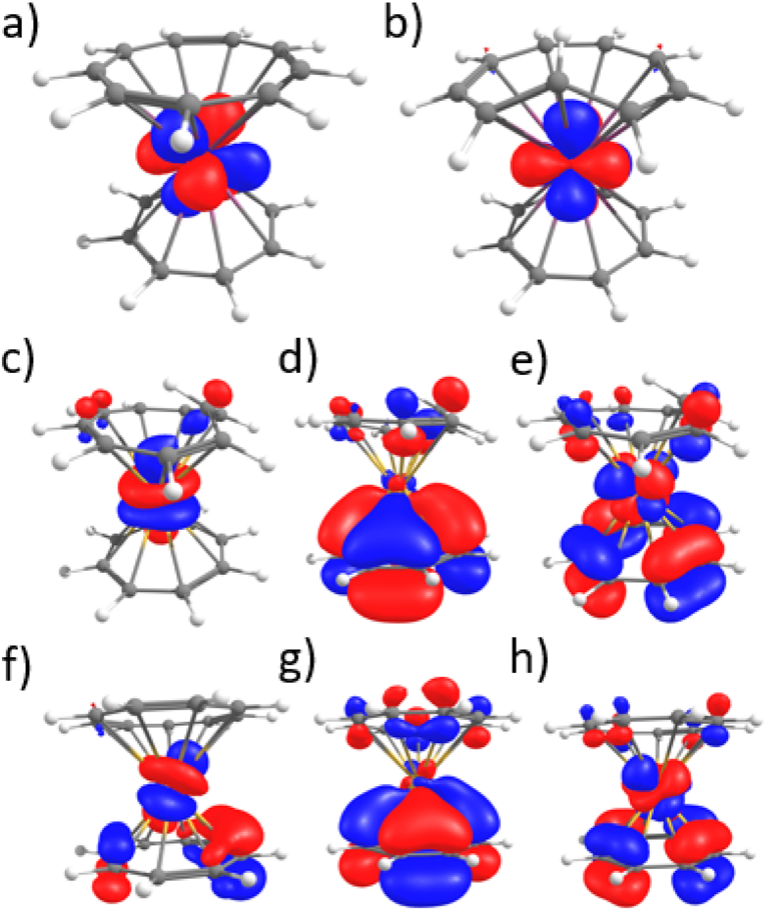
Selected molecular orbitals (a) αMO-HOMO-2 of 2-Ce-*cis* (SOMO). (b) αMO-HOMO-2 2-Ce-*trans* (SOMO) (c) αMO-HOMO-4 of 2-Sm-*trans* (d) αMO-HOMO of 2-Sm-*trans* (e) αMO-LUMO of 2-Sm-*trans* (f) αMO-HOMO-8 of 2-Sm-*cis* (d) αMO-HOMO of 2-Sm-*cis* (e) αMO-LUMO of 2-Sm-*cis*.

One important question that remained was the reason for the higher stabilization of the *trans* form with high-energy light. Although several populated orbitals have little ligand contribution, particularly with the carbon, which is out of the plane (see Fig. 9), the density of the ligand is minimal. However, it is notable that in virtual orbitals, the ligand contribution is significantly higher (see Fig. 9), which could play a role in what was observed in this work. Yet, this would imply a complete analysis of the excited-states and relaxation pathway, which is not in the scope of this article. However, TD-DFT was attempted to track the nature of the transitions, particularly those at lower wavelengths.

TD-DFT calculations were carried out on the different complexes of Ce (2-Ce) and Sm (2-Sm) (see ESI†). For 2-Sm, no remarkable difference could be observed. Most of the transitions are multiconfigurational, and the proximity and the similitude of the transitions between 2-Sm-*trans* and 2-Sm-*cis* do not allow a clear-cut analysis. This relies well on the difficulty of predicting the PSS and the isomerization patterns from the analysis of the optical spectra and of the molecular orbitals.

In the case of 2-Ce, a peak in the visible region is observed for the *trans* complex at 442 nm, in good agreement with the signal at 470 nm. The transition corresponds to an excitation from the unpaired f-electron to the LUMO, which is mainly located on the Ce–(Cnt) interaction. However, although the 2-Ce-*cis* complex shows a signal in lower energy at 613 nm, in our hand, the TD-DFT does not reproduce this data. This would then need a different theoretical framework of the excited states, which is outside the scope of this study.

## Conclusions

This work describes a quantitative study of the isomerization of the Cnt ligand and its lanthanide sandwich complexes using light as the main trigger. A careful synthesis of the compounds was carried out in the absence of light to obtain the *trans* isomer for the Cnt ligand, the Sm(Cnt)_2_, and (Cot)(Cnt)Ln (Ln = Y, La, Ce, Pr, Nd, Sm, Gd, Tb, Er, Ho) complexes. These compounds were characterized through ^1^H NMR and X-ray crystallography.

A fast isomerization rate was observed for the Cnt ligand potassium salt and for the (Cnt)_2_Sm complex, allowing *trans* to *cis* isomerization. While these systems were proven irreversible, a photostationary state (PSS) was observed for the (Cot)(Cnt)Sm complex, in which both isomers were present. A systematic study of the PSS was conducted at different wavelengths (from 370 nm to 525 or 655 nm) on all (Cot)(Cnt)Ln complexes, which were monitored by ^1^H NMR. Under high energy irradiation, with Ln = Sm, Tb, Dy, and Lu, the *trans* isomer was favored, while the *cis* isomer was favored under low energy irradiation. In the case of Ln = La, Ce, and Y, the *trans* isomer was strongly favored at any given wavelength.

Our measurements of the absorption spectra and the theoretical analyses show that a comprehensive analysis of the PSS and the photo-isomerization would require dedicated expertise, which was outside the scope of our synthetic and structural studies. However, we are confident that the findings gathered in this article will open a broad scope of future studies by us and the community.

## Data availability

The data supporting this article have been included as part of the ESI.†

## Author contributions

LP, NM, and CC performed all the experimental work, with equal contributions from LP and NM. TM, ID, and LM performed the theoretical work. NC, MT, and NM worked out the crystal structures with the help of LP. LM, GN, and GD analyzed and interpreted all the data together. GD and GN came up with the idea and oversaw all the work. GN wrote the structure of the article with contributions from all authors.

## Conflicts of interest

There are no conflicts to declare.

## Supplementary Material

SC-OLF-D4SC04767B-s001

SC-OLF-D4SC04767B-s002

SC-OLF-D4SC04767B-s003
