# Supplementary material for: The photo-isomerization of the cyclononatetraenyl ligand and related rare earth complexes
Source: Chem Sci. 2024 Oct 18;15(46):19273–82. doi: 10.1039/d4sc04767b (PMC11575527; doi:10.1039/d4sc04767b)
Supplement: SC-015-D4SC04767B-s002 [file SC-015-D4SC04767B-s002.pdf]

Supplementary Information (SI) for Chemical Science.
This journal is © The Royal Society of Chemistry 2024

Supporting information for the manuscript

Photo-isomerization of the cyclononatetraenyl ligand and related rare earth complexes

Lucie Pedussaut,[a‡] Nolwenn Mahieu,[a‡] Camille Chartier,[a] Thayalan Rajeshkumar,[b] Maxime Tricoire,[a] Iskander Douair,[b] Nicolas Casaretto,[a] Laurent Maron,[b] Grégory Danoun *[a] and Grégory Nocton*[a]

a. LCM, CNRS, Ecole polytechnique, Institut polytechnique de Paris, Route de Saclay, 91120 Palaiseau, France.
b. LPCNO, UMR 5215, Université de Toulouse-CNRS, INSA, UPS, Toulouse, France.
‡ Both authors contributed equally to the work

Summary

1. General information 2
2. Experimental section 3
3. ^{1}H NMR Spectroscopy 7
4. X-ray crystallography 19
5. UV-visible Spectroscopy 49
6. Isomerization study 54
   a) UV and NMR study 55
   b) Photostationary (PSS) study 58
7. Computational details 69
8. References 140

1. General information

All air- and moisture-sensitive reactions were performed using standard Schlenk-line techniques under dry N_2 or Ar atmosphere or in argon-filled gloveboxes (MBraun). All glassware was dried at 140 °C for at least 12 h prior to use. All solvents (Et_2O, toluene, benzene, pentane, C_6D_6, tol-d_8, THF-d_8) were dried over sodium, degassed, and transferred under reduced pressure in a cold flask. Acetonitrile and deuterated acetonitrile were dried over CaH_2, degassed, and transferred under reduced pressure in a cold flask. K_2Cot, KCnt was prepared according to literature procedures. All LnCotI(thf)$_n$ were synthesized according to a modified procedure published literature by using the corresponding LnI_3 except for Ln = Sm where another synthesis pathway was used.[1–4] For Ln = Lu, [(Cot)Lu(BH_4)(thf)]$_2$ was synthesized according to known procedure.[1] Ln(Cot)(*cis*-Cnt) were prepared according to known procedure Ln = Tb-Tm.[1] All other chemicals were obtained from commercial sources and used without further purification. ^{1}H NMR spectra were recorded in 5 mm tubes adapted with a J. Young valve on a Bruker Avance III-300 MHz spectrometer and chemical shifts are expressed relative to internal solvent references in ppm. UV-visible absorption spectra were recorded on a cary60 spectrometer in quartz cuvettes adapted with a J. Young valve. Irradiation were performed using Kessils lamps ans Prizmatix multi-Wavelength FC5-LED. Elemental analyses were obtained from Mikroanalytisches Labor Pascher (Remagen, Germany). IR spectra were recorded using Thermo Scientific Nicolet iS5 spectrometer equipped with the ATR iD7 accessory.

2. Experimental section

a. General procedures:

General procedure A: Synthesis of 2-Ln-*cis*

A mixture of LnCotI(thf)$_n$ (n = 2, 3) (1.05 equiv.) and KCnt (1 equiv.) in toluene and acetonitrile (10:1) was allowed to stir at room temperature. After 12 h, the vessel was protected from light and the solvent were removed under reduced pressure. The reaction mixture was then dissolved in toluene. The evaporation / dissolution in toluene step was repeated once. The colored supernatant was then filtered and concentrated to yield **2**-Ln-*cis*. The desired compound can be obtained as microcrystalline powder through toluene extraction and evaporation. XRD suitable crystals were obtained by concentration of the toluene solution at -40°C.

General procedure B: Synthesis of 2-Ln-*trans*

All steps are protected from light. A mixture or LnCotI(thf)$_n$ (n = 2, 3) (1 equiv.) and KCnt (1 equiv.) in toluene was protected from light with aluminum foil and allowed to stir. After 12 h, the supernatant was filtered, concentrated, and cooled to yield a mixture of isomers with a high ratio of **2**-Ln-*trans*. The desired compound can be obtained as microcrystalline powder through toluene extraction and slow evaporation of dryness. XRD suitable crystals were obtained by concentration of the toluene solution at -40°C.

b. Synthesis of 2-Ln-*cis*:

2-Y-*cis*: Synthesized according to general procedure **A** YCotI(thf)$_3$ (106 mg, 0.20 mmol, 1.0 equiv) and KCnt (31 mg, 0.20 mmol, 1.05 equiv) as yellow micro-crystalline powder (24.9 mg, 40%)
^{1}H NMR (300 MHz, toluene-d$_8$, 293 K): δ (ppm) 6.77 (s, 9H, Cnt), 5.93 (s, 8H, Cot)
Anal. Calcd. for $C_{17}H_{17}Y$ (310.23): C, 65.82; H, 5.52 Found: C, 63.65; H, 5.42.

2-La-*cis*: Synthesized according to general procedure **A** LaCotI(thf)$_3$ (61.1 mg, 0.10 mmol, 1.0 equiv) and KCnt (17.2 mg, 0.11 mmol, 1.05 equiv) as orange micro-crystalline powder (26.9 mg, 71%)
^{1}H NMR (300 MHz, CD_2Cl_2, 293 K): δ (ppm) 7.53 (s, 9H, Cnt), 6.03 (s, 8H, Cot)
Anal. Calcd. for $C_{17}H_{17}La$ (360.04): C, 56.68; H, 4.76 Found: C, 56.75; H, 4.81

2-Ce-*cis*: Synthesized according to general procedure **A** CeCotI(thf)$_3$ (150 mg, 0.25 mmol, 1.0 equiv) and KCnt (42 mg, 0.26 mmol, 1.05 equiv) as green micro-crystalline powder (53 mg, 58%)
^{1}H NMR (300 MHz, toluene-d$_8$, 293 K): δ (ppm) 6.22 (s, 9H, Cnt), 4.56 (s, 8H, Cot)
Anal. Calcd. for $C_{17}H_{17}Ce$ (361.43): C, 56.49; H, 4.74 Found: C, 56.36; H, 4.79.

2-Pr-*cis*: Synthesized according to general procedure **A** PrCotI(thf)$_3$ (70.9 mg, 0.12 mmol, 1.0 equiv) and KCnt (19.9 mg, 0.13 mmol, 1.05 equiv) as light brown micro-crystalline powder (25.6 mg, 59%)
^{1}H NMR (300 MHz, toluene-d$_8$, 293 K): δ (ppm) -0.60 (s, 9H, Cnt), -12.07 (s, 8H, Cot)
Anal. Calcd. for $C_{17}H_{17}Pr$ (362.23): C, 56.37; H, 4.73 Found: C, 55.80; H, 4.74.

2-Nd-*cis*: Synthesized according to general procedure **A** NdCotI(thf)$_3$ (72.1 mg, 0.12 mmol, 1.0 equiv) and KCnt (20.4 mg, 0.13 mmol, 1.05 equiv) as greenish micro-crystalline powder (24.0 mg, 54 %)
^{1}H NMR (300 MHz, toluene-d$_8$, 293 K): δ (ppm), 0.27 (s, 9H, Cnt), -13.70 (br s, 8H, Cot)
Anal. Calcd. for $C_{17}H_{17}Nd$ (363.04): C, 55.86; H, 4.69 Found: C, 55.53; H, 4.68

2-Sm-*cis*: Synthesized according to general procedure **A** SmCotI(thf)$_3$ (100 mg, 0.17 mmol, 1 equiv.) and KCnt (25 mg, 0.18 mmol, 1 equiv.) as small green micro-crystalline powder (24 mg, 38 %)
^{1}H NMR (300 MHz, toluene-d$_8$, 293 K): δ (ppm) 16.47 (br s, 8H, Cot), 11.39 (s, 9H, Cnt)
Anal. Calcd. for $C_{17}H_{17}Sm$ (371.68): C, 54.89; H, 4.57; Found: C, 53.45; H, 4.56.

2-Gd-*cis*: Synthesized according to general procedure **A** GdCotI(thf)$_3$ (79.9 mg, 0.15 mmol, 1.0 equiv) and KCnt (24.8 mg, 0.16 mmol, 1.05 equiv) as small orange needles (22 mg. 36 %)
^{1}H NMR (300 MHz, toluene-d$_8$, 293 K): δ (ppm), 97.94 (br s), 23.50 (br s)
Anal. Calcd. for $C_{17}H_{17}Gd$ (379.06): C, 53.94; H, 4.53 Found: C, 49.18; H, 4.30.

2-Y-*trans*: Synthesized according to general procedure **B** YCotI(thf)$_3$ (29.1 mg, 0.0627 mmol, 1.00 equiv.) and KCnt (10.4 mg, 0.0661 mmol, 1.05 equiv.) as bright orange-red crystals (3.0 mg, 15%, 61% *trans*).
^{1}H NMR (300 MHz, toluene-d$_8$, 293 K): 6.77 (s, Cnt^{cis}), 6.55 (s br, Cnt^{trans}), 6.02 (s, Cot^{trans}), 5.94 (s, Cot^{cis}), -3.19 (t, Cnt^{trans})
Anal. Calcd. for $C_{17}H_{17}Y$ (310.23): C, 65.82; H, 5.52 Found: C, 53.32; H, 4.95.

2-La-*trans*: Synthesized according to general procedure **B** LaCotI(thf)$_3$ (64.7 mg, 0.11 mmol, 1.00 equiv) and KCnt (18.7 mg, 0.12 mmol, 1.09 equiv) as small orange crystalline needles (4.0 mg, 10%, 55% *trans*)
^{1}H NMR (300 MHz, CD_2Cl_2, 293 K): δ (ppm) 7.52 (s, Cnt^{cis}), 7.50(s, 2H, Cnt^{trans}), 6.96 (m, 2H, Cnt^{trans}), 6.83(m, 4H, Cnt^{trans}), 6.12 (s, 8H, Cot-*trans*), 6.02(s, Cot-*cis*), -3.92 (t, 1H, Cnt^{trans}, J = 12 Hz)
Anal. Calcd. for $C_{17}H_{17}La$ (360.04): C, 56.68; H, 4.76 Found: C, 55.80; H, 4.71.

2-Ce-*trans*: Synthesized according to general procedure **B** CeCotI(thf)$_3$ (58.2 mg, 0.099 mmol, 1 equiv) and KCnt (15.8 mg, 0.10 mmol, 1.01 equiv) as small green crystalline needles (4.3 mg, 12%, 79 % *trans*)
^{1}H NMR (300 MHz, CD_2Cl_2, 293 K): δ (ppm) 25.68 (s, 2H, Cnt^{trans}), 15.55 (s, 2H, Cnt^{trans}), 6.75 (br s, Cnt^{cis}), 4.46 (br s, Cot^{cis}), 3.36 (s, 8H, Cot^{trans}), -16.23 (s, 2H, Cnt^{trans}), -50.30 (s, 1H, Cnt^{trans})
Anal. Calcd. for $C_{17}H_{17}Ce$ (361.43): C, 56.49; H, 4.74 Found: C, 55.28; H, 4.66.

2-Pr-*trans*: Synthesized according to general procedure **B** PrCotI(thf)$_3$ (65.5 mg, 0.11 mmol, 1 equiv) and KCnt (18.9 mg, 0.12 mmol, 1.09 equiv) as small orange crystalline needles (5.6 mg, 14%)
^{1}H NMR (300 MHz, CD_2Cl_2, 293 K): δ (ppm) 84.96 (s br, 1H, Cnt^{trans}), 7.67 (S, 2H, Cnt^{trans}), -0.21- -0.49 (m br, 4H, Cnt$^{trans/cis}$), -1.96 (s, 2H, Cnt^{trans}), -11.65 (s, 12H, Cot$^{trans/cis}$), -23.00 (s, 2H, Cnt^{trans})
Anal. Calcd. for $C_{17}H_{17}Pr$ (362.23): C, 56.37; H, 4.73 Found: C, 55.80; H, 4.74.

2-Nd-*trans*: Synthesized according to general procedure **B** NdCotI(thf)$_3$ (62.2 mg, 0.105 mmol, 1 equiv) and KCnt (17.2 mg, 0.109 mmol, 1.04 equiv) as small orange crystalline needles (5.3 mg, 14%)
^{1}H NMR (300 MHz, CD_2Cl_2, 293 K): δ (ppm) 21.16 (s, 2H, Cnt^{trans}), 7.19 (s, 2H, Cnt^{trans}), 6.68 (s, 2H, Cnt^{trans}), -13.84 (s, 8H, Cot^{trans}), -26.83 (s, 2H, Cnt^{trans}).
Anal. Calcd. for $C_{17}H_{17}Nd$ (363.04): C, 55.86; H, 4.69 Found: C, 55.80; H, 4.63

2-Sm-*trans*: Synthesized according to general procedure **B** SmCotI(thf)$_3$ (52 mg, 0.1 mmol, 1 equiv) and KCnt (15 mg, 0.1 mmol, 1 equiv) as small green crystalline needles (5.2 mg, 15% yield, 89% *trans*).

^1H NMR (300 MHz, toluene-d$_8$, 293 K): δ (ppm) 16.45 (br, Cot^{cis}), 15.60 (s, 8H, Cot^{trans}), 15.02 (s, 2H, Cnt^{trans}), 14.59 (s, 2H Cnt^{trans}), 11.41 (s, Cnt^{cis}), 11.00 (s, 2H, Cnt^{trans}), 6.19 (s, 2H, Cnt^{trans}), -40.52 (s, 1H, Cnt^{trans})
Anal. Calcd. for $C_{17}H_{17}Sm$ (371.68): C, 54.89; H, 4.57; Found: C, 54.37; H, 4.55.

2-Gd-*trans*: Synthesized according to general procedure **B** GdCotI(thf)$_2$ (63.1 mg, 0.118 mmol, 1 equiv) and KCnt (19.1 mg, 0.121 mmol, 1.03 equiv) as small orange crystalline needles (6.5 mg, 17%)
^1H NMR (300 MHz, CD_2Cl_2, 293 K): δ (ppm) 34.84-23.62 (br), 23.62 (br), 12.18 (br)
Anal. Calcd. for $C_{17}H_{17}Gd$ (379.06): C, 53.94; H, 4.53 Found: C, 52.14; H, 4.45.

2-Tb-*trans*: Synthesized according to general procedure **B** TbCotI(thf)$_2$ (42.2 mg, 0.079 mmol, 1.00 equiv) and KCnt (13.0 mg, 0.083 mmol, 1.05 equiv) as small orange crystalline needles (8.0 mg, 27% yield, 87% *trans*)
^1H NMR (300 MHz, toluene-d$_8$, 293 K): δ (ppm) 391.13 (s, 2H, Cnt^{trans}), 242.25 (s, Cot^{cis}), 225.40 (s, 8H, Cot^{trans}), 196.81 (s, 2H, Cnt^{trans}), 163.00 (s, 2H, Cnt^{trans}), 100.49 (s, Cnt^{cis}), -156.76 (s, 2H, Cnt^{trans})
Anal. Calcd. for $C_{17}H_{17}Tb$ (380.06): C, 53.70; H, 4.51 Found: C, 53.73; H, 4.54.

2-Dy-*trans*: Synthesized according to general procedure **B** DyCotI(thf)$_2$ (67.7 mg, 0.126 mmol, 1.00 equiv.) and KCnt (21.1 mg, 0.134 mmol, 1.06 equiv.) as small orange crystalline needles (4.5 mg, 9 %, 75% *trans*)
^1H NMR (300 MHz, CD_2Cl_2, 293 K): δ (ppm), 158.33 (br s, 2H, Cnt^{trans}), 115.04 (br s, Cnt^{cis}), 99.55 (br s, 2H, Cnt^{trans}), 72.10 (br s, Cot$^{trans/cis}$), 44.47 (br s, 2H, Cnt^{trans}), -168.91 (br s, 2H, Cnt^{trans})
Anal. Calcd. for $C_{17}H_{17}Dy$ (383.82): C, 53.20; H, 4.46 Found: C, 52.76; H, 4.47.

2-Ho-*trans*: Synthesized according to general procedure **B** HoCotI(thf)$_2$ (54.8 mg, 0.101 mmol, 1.00 equiv.) and KCnt (16.6 mg, 0.106 mmol, 1.05 equiv.) as small orange crystalline needles (5.7 mg, 15 %)
^1H NMR (300 MHz, CD_2Cl_2, 293 K): δ (ppm), 198.17 (br s, Cnt^{trans}), 101.99 (br s, Cnt^{trans}), 87.22 (br s, Cnt^{cis}), 66.81 (br s, Cot^{trans}), 57.81 (br s, Cot^{cis}), 18.98 (br s, Cnt^{trans}), -124.84 (br s, Cnt^{trans})
Anal. Calcd. for $C_{17}H_{17}Ho$ (386.06): C, 52.86; H, 4.44 Found: C, 47.75; H, 4.20

2-Er: Synthesized according to general procedure **B** ErCotI(thf)$_2$ (59.4 mg, 0.109 mmol, 1.00 equiv.) and KCnt (18.6 mg, 0.118 mmol, 1.08 equiv.) as bright orange crystals (4.4 mg, 10%) where only the *cis* product could be obtained.
^1H NMR (300 MHz, toluene-d$_8$, 293 K): δ (ppm), -3.94 (br s), -127.52 (br s)
Anal. Calcd. for $C_{17}H_{17}Er$ (388.58): C, 52.55; H, 4.41 Found: C, 51.37; H, 4.35

2-Tm: Synthesized according to general procedure **B** TmCotI(thf)$_2$ (57.4 mg, 0.105 mmol, 1.00 equiv.) and KCnt (17.4 mg, 0.111 mmol, 1.05 equiv.) as bright orange crystals (3.8 mg, 9%) where only the *cis* product could be obtained.
^1H NMR (300 MHz, toluene-d$_8$, 293 K): δ (ppm), -22.87 (br s), -227.25 (br s)
Anal. Calcd. for $C_{17}H_{17}Tm$ (390.07): C, 52.32; H, 4.39 Found: C, 45.77; H, 4.13.

2-Lu: Synthesized according to general procedure **B** [LuCot(thf)(BH$_4$)]$_2$ (146 mg, 0.33 mmol, 1.00 equiv.) and KCnt (52 mg, 0.33 mmol, 1.0 equiv.) as yellow needles (26 mg, 9%) where only the *cis* product could be obtained.

^{1}H NMR (300 MHz, toluene-d_8, 293 K): δ (ppm), 6.58 (s, 9H, Cnt^{cis}), 5.88 (s, 8H, Cot^{cis})
Anal. Calcd. for $C_{17}H_{17}Lu$ (396.29): C, 51.52; H, 4.32 Found: C,51.14; H, 4.96

c. Synthesis of 1-*trans*

All steps are protected from light. A mixture or SmI_2 (49.1 mg, 12mmol, 1 equiv.) and KCnt (21 mg, 13 mmol, 1.1 equiv.) in toluene was protected from light with aluminum foil and allowed to stir. After 12 h, the supernatant was filtered, and cooled at -40 °C to yield small black blocks (5.2 mg, 11%, 100% *trans,trans*). No crystals suitable for XRD could be obtained without a considerable isomerization of the compound from **1**-*trans* to **1**-*cis*.
^{1}H NMR (300 MHz, toluene-d_8, 293 K): δ (ppm) 51.27 – 49.57 (s, 4H), 26.34 (s, 4H), 21.10 (s, 4H), 15.34 (s, 4H), -0.95 (s, 2H).
Anal. Calcd. for $C_{18}H_{18}Sm$ (384.70): C, 56.2; H, 4.72 Found: C, 52.83; H, 4.57.

3. ^{1}H NMR Spectroscopy

a. The KCnt ligand

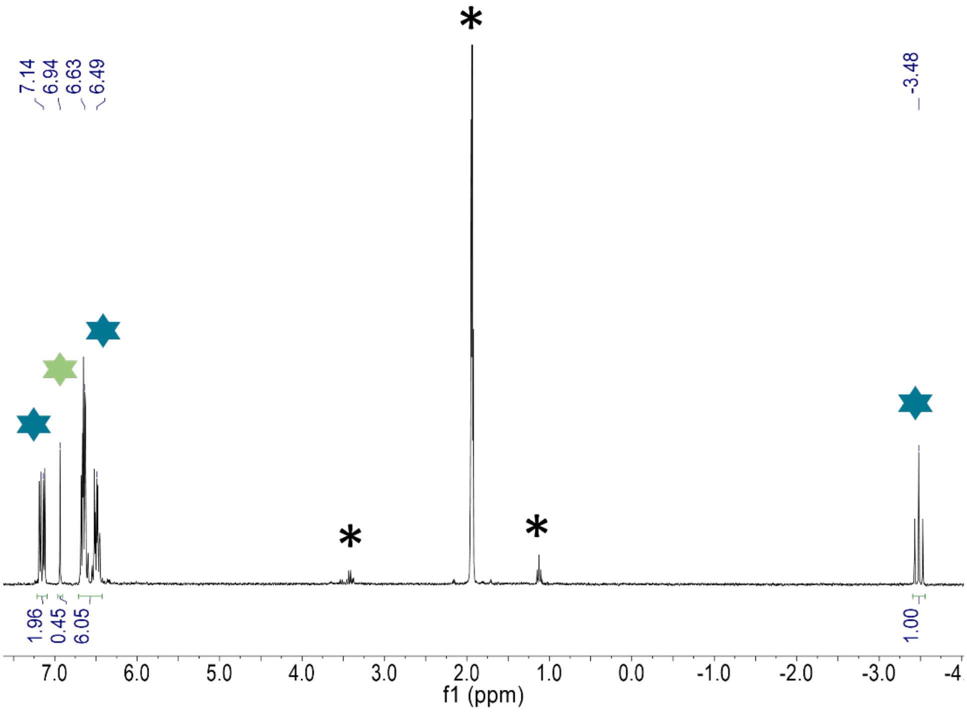

Figure S1: *^{1}H NMR of KCnt-trans in CD_3CN measured at 293 K (* residual protio signal of the solvent). Green star: signal attributed to KCnt-cis. Blue star: signal attributed to KCnt-trans*

b. Complexes **1**

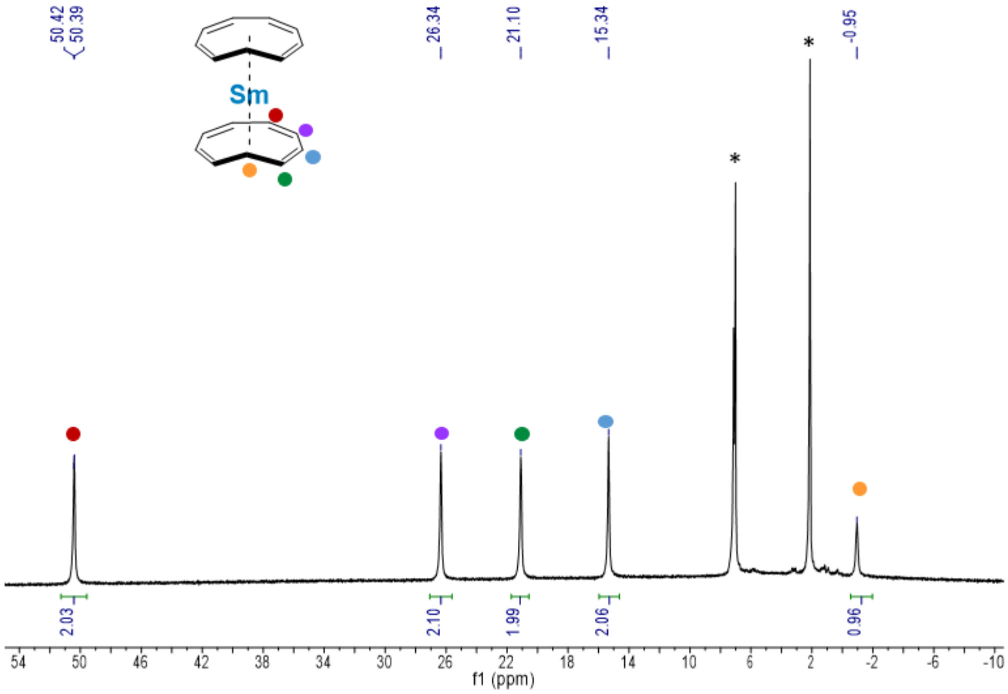

Figure S2: *^{1}H NMR of* ***1-trans*** *in toluene-d_8 measured at 293 K (* residual protio signal of the solvent).*

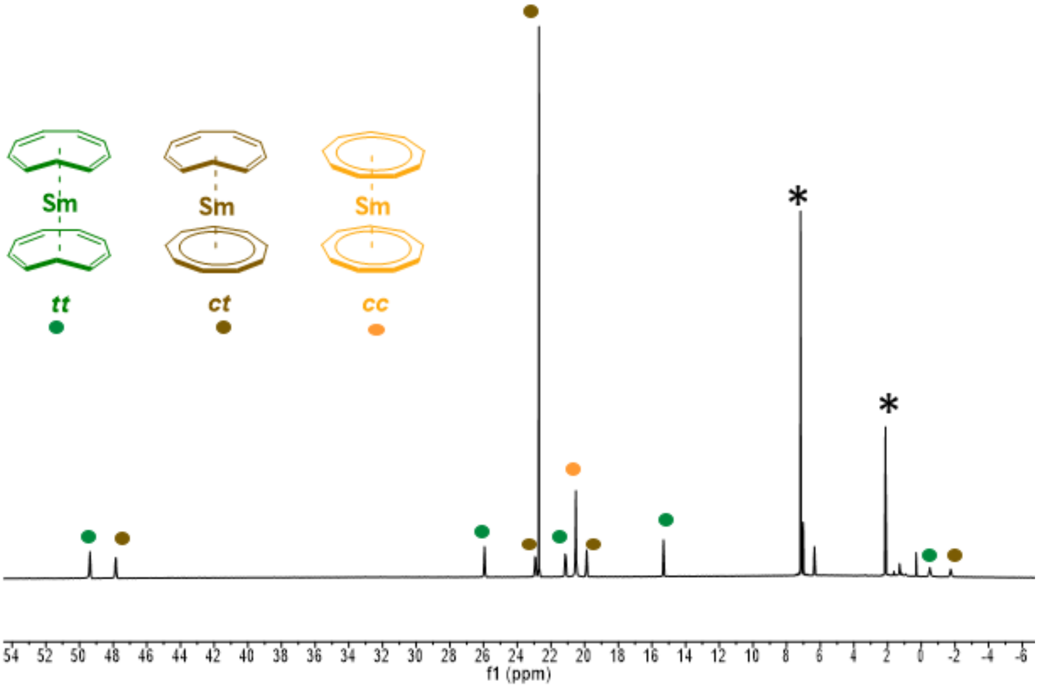

Figure S3: ^{1}H NMR of **1** after crystallization in toluene-d_8 measured at 293 K (49 % of **1**-trans; 40 % **1**-cis-trans; 11 % of **1**-cis (* residual protio signal of the solvent).

c. Complexes **2**

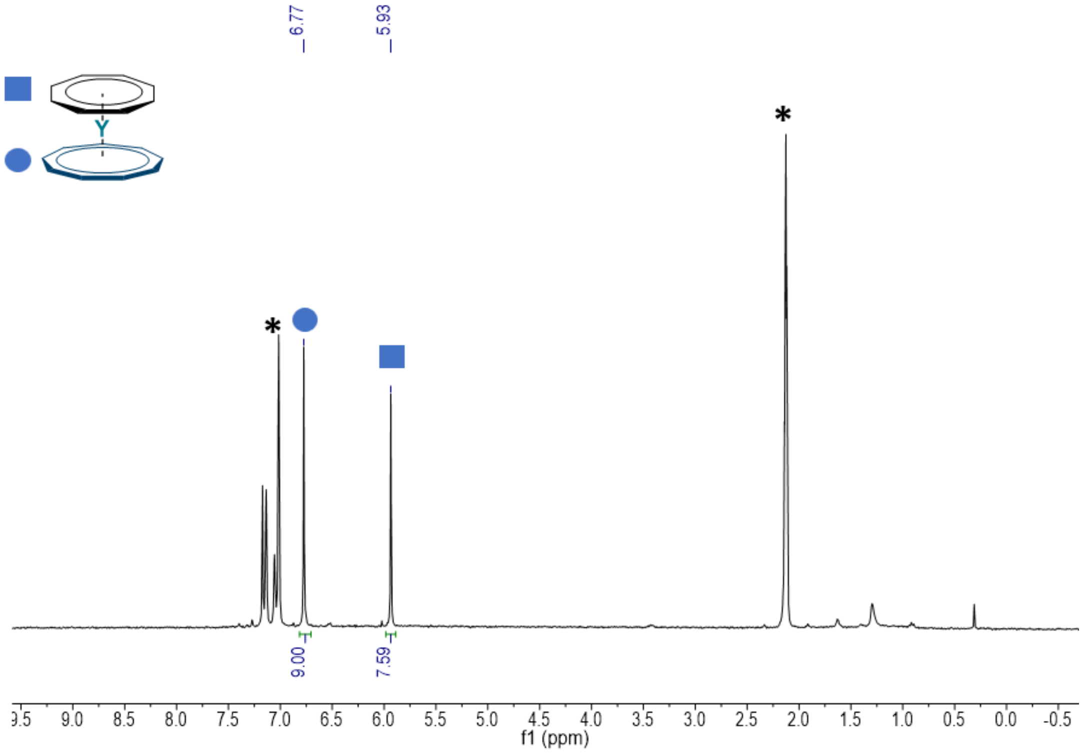

Figure S4: ^{1}H NMR **2**-Y-cis in toluene-d_8 measured at 293 K (* residual protio signal of the solvent)

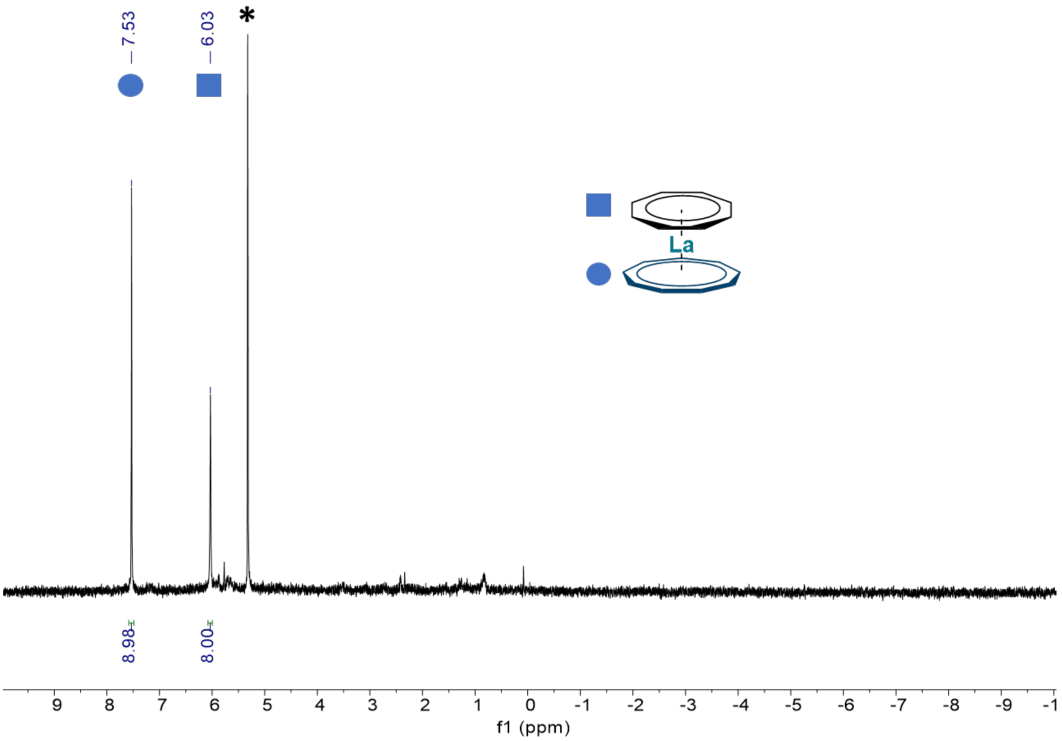

Figure S5: *^{1}H NMR of **2**-La-cis in CD_2Cl_2 measured at 293 K (* residual protio signal of the solvent)*

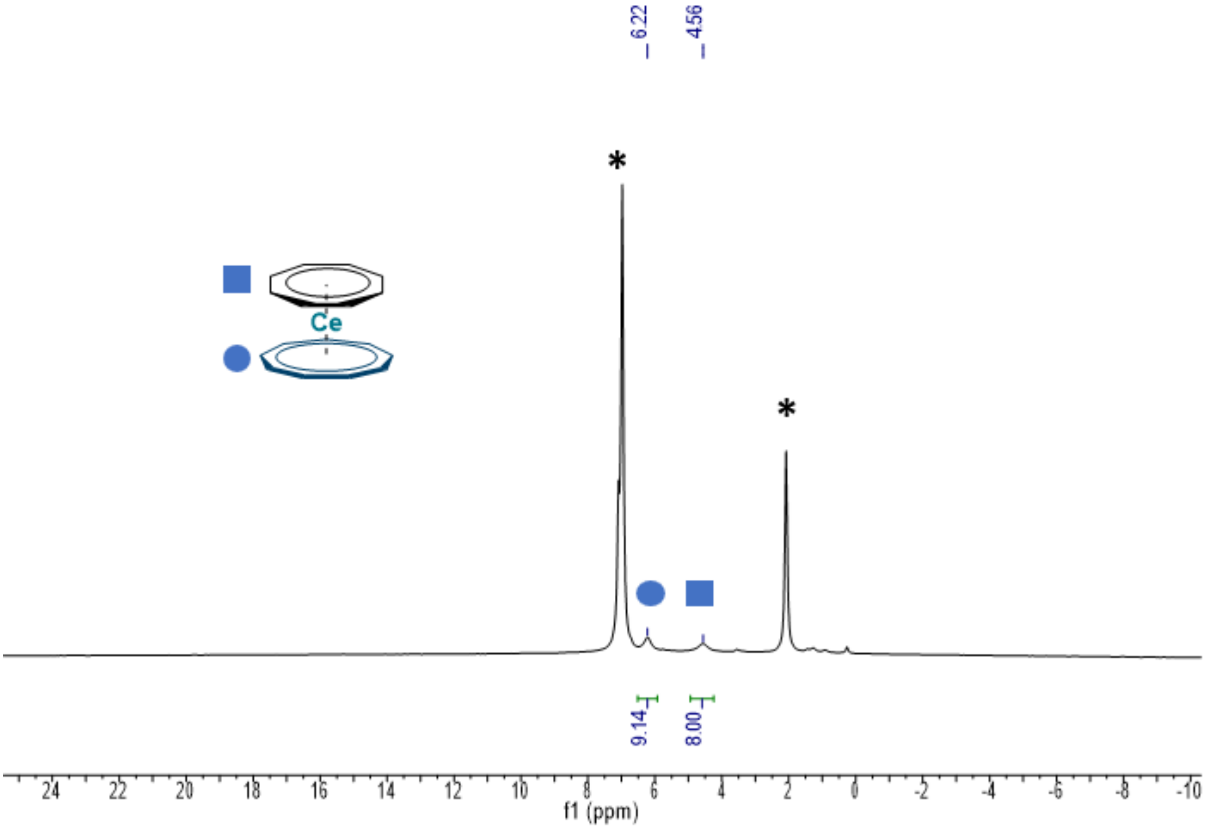

Figure S6: *^{1}H NMR of **2**-Ce-cis in toluene-d_8 measured at 293 K (* residual protio signal of the solvent)*

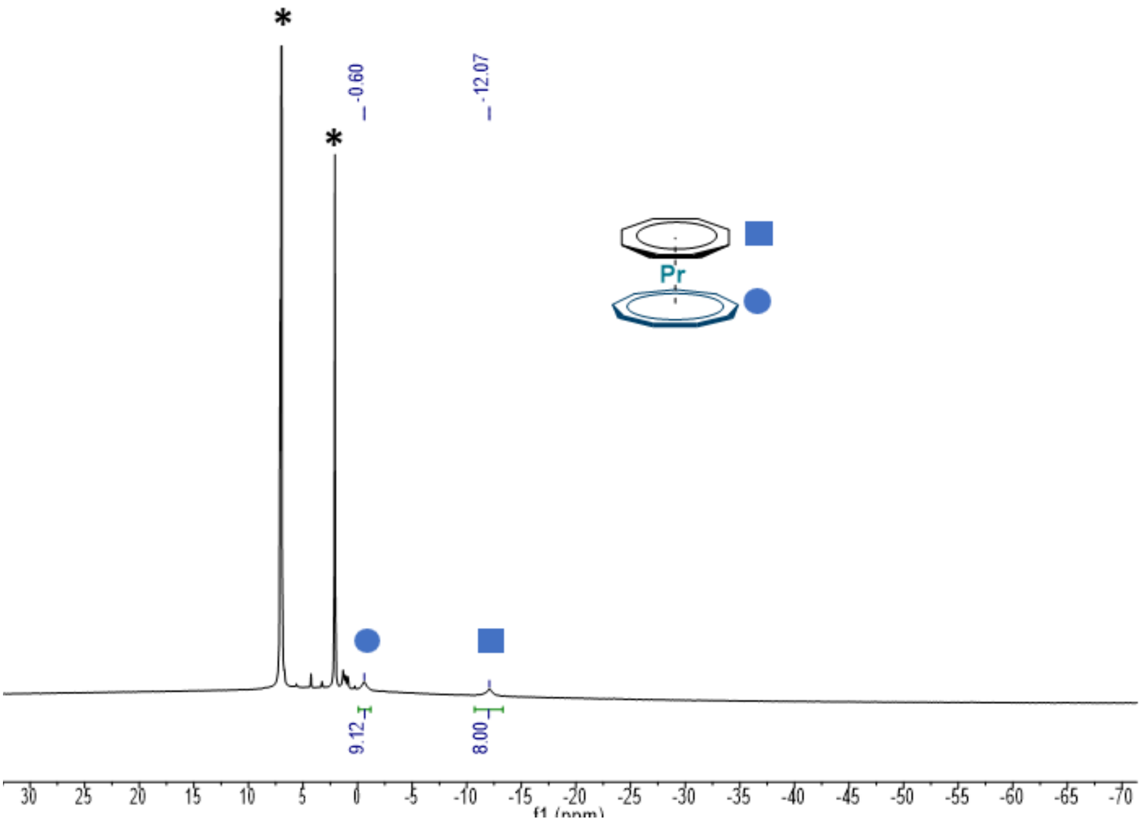

***Figure S7**: ^{1}H NMR of **2**-Pr-cis in toluene-d_8 measured at 293 K (* residual protio signal of the solvent)*

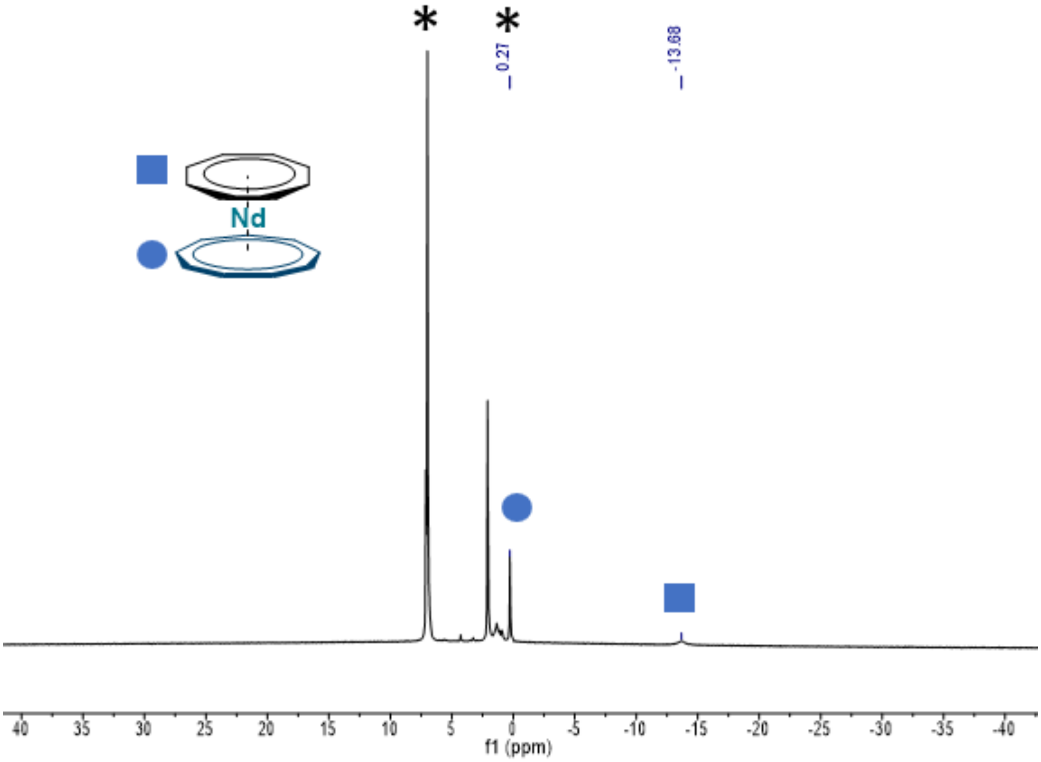

***Figure S8**: ^{1}H NMR of **2**-Nd-cis in toluene-d_8 measured at 293 K (* residual protio signal of the solvent). The uncertainty of integration does not allow for specific attribution.*

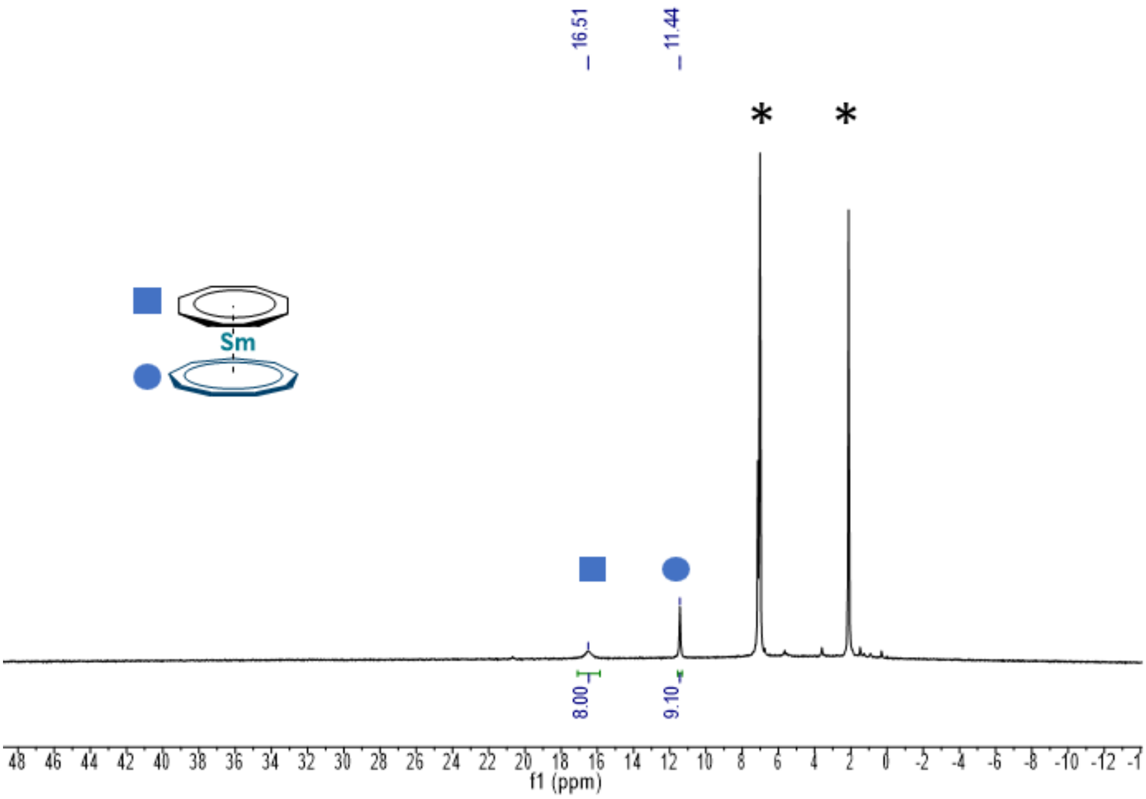

Figure S9: ^{1}H NMR of **2**-Sm-cis in toluene-d_8 measured at 293 K (* residual protio signal of the solvent).

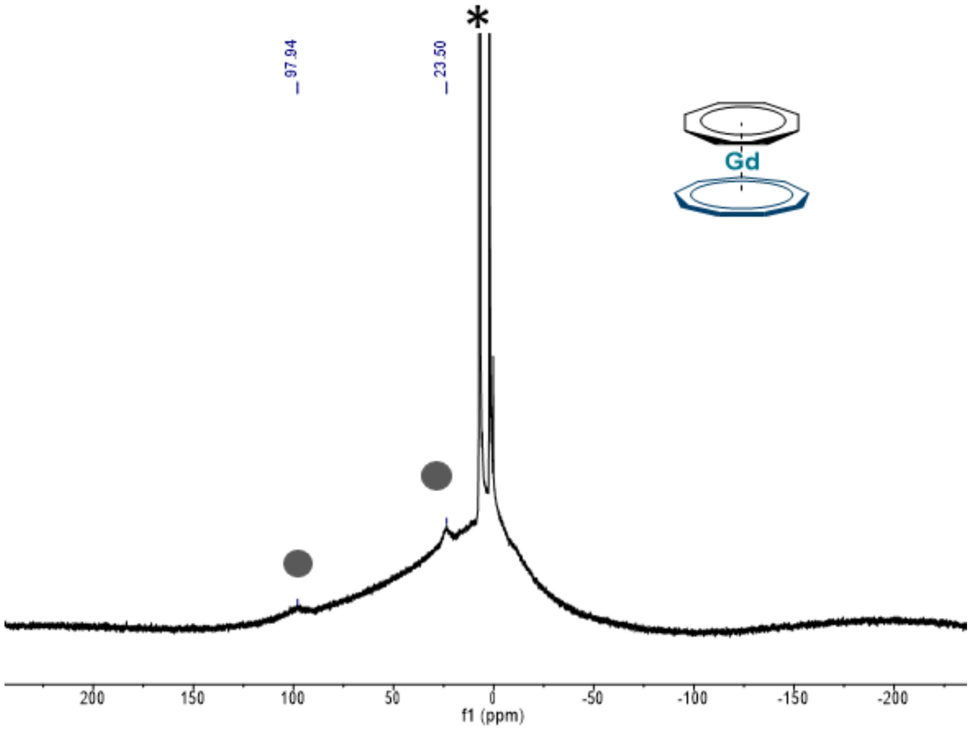

Figure S10: ^{1}H NMR of **2**-Gd-cis in toluene-d_8 measured at 293 K (* residual protio signal of the solvent). The uncertainty of integration does not allow the attribution.

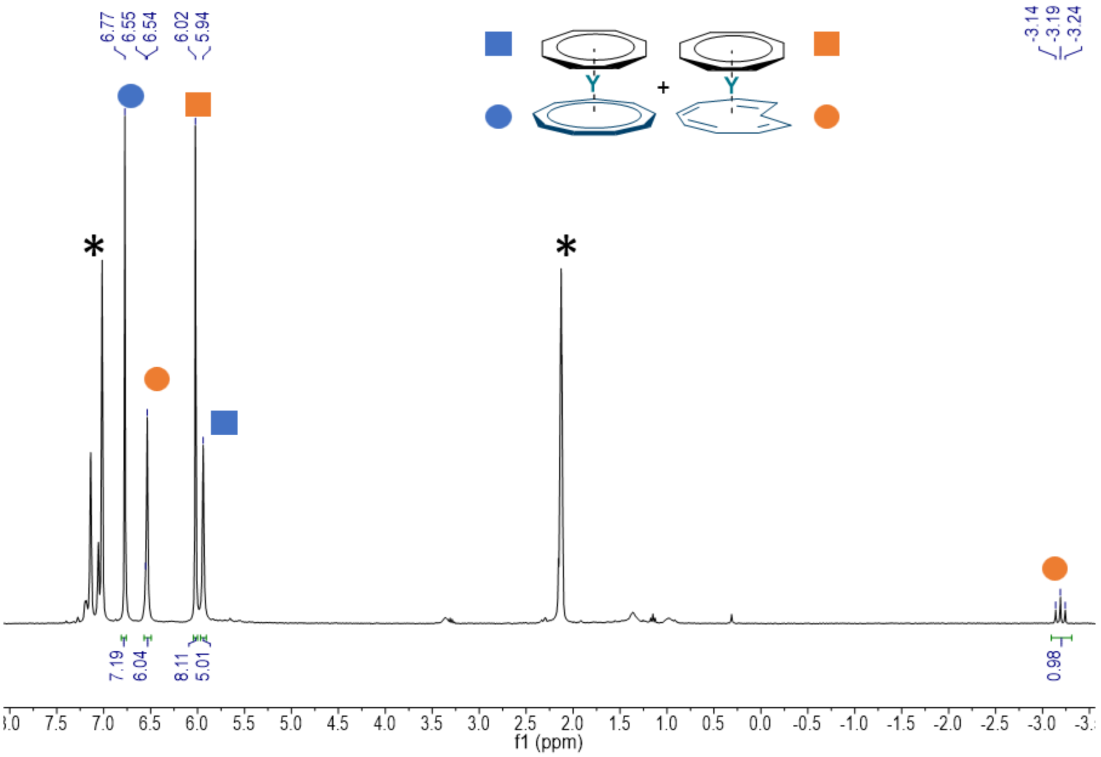

Figure S11: *^{1}H NMR of **2**-Y-trans in toluene-d_8 measured at 293 K (* residual protio signal of the solvent).*

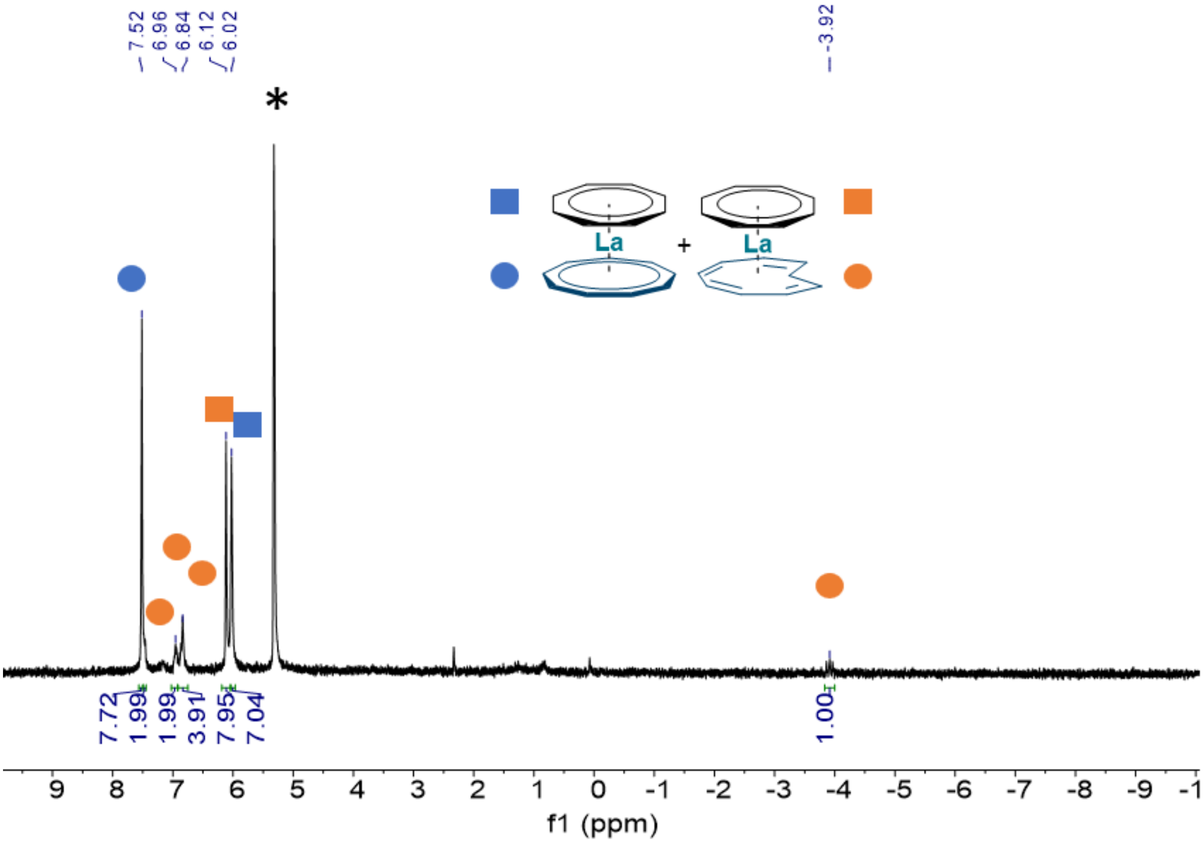

Figure S12: *^{1}H NMR of **2**-La-trans in CD_2Cl_2 measured at 293 K (* residual protio signal of the solvent).*

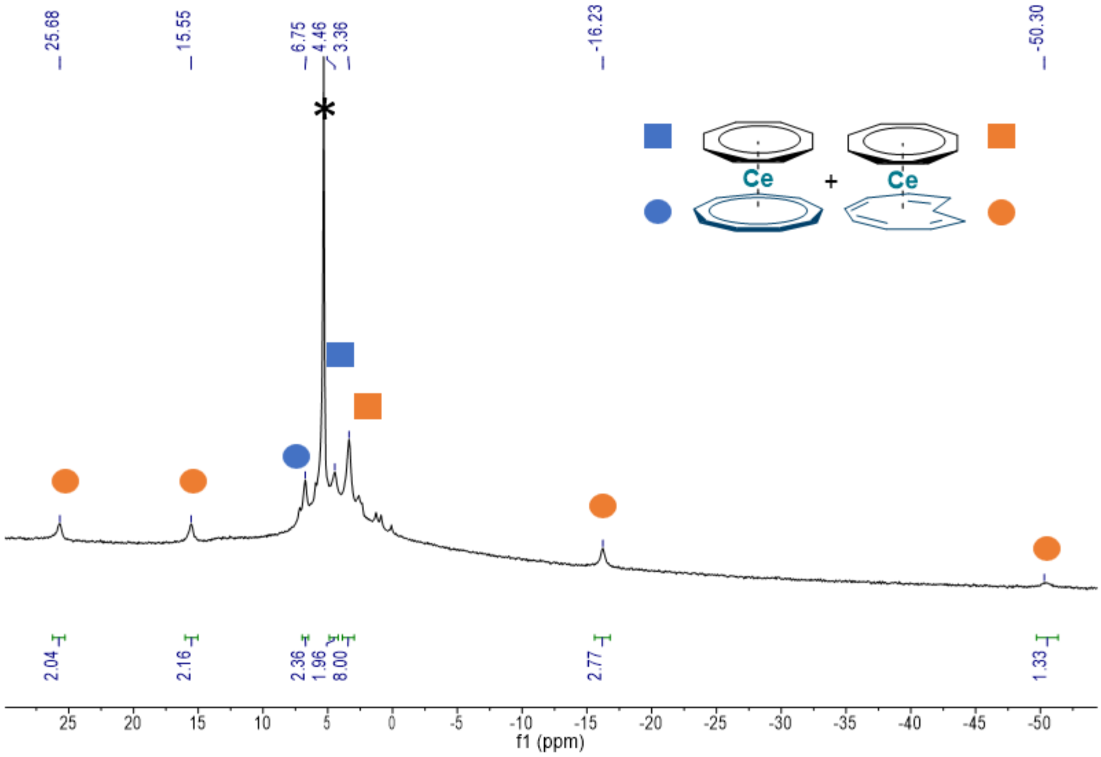

Figure S13: *^{1}H NMR of **2**-Ce-trans in CD_2Cl_2 measured at 293 K (* residual protio signal of the solvent).*

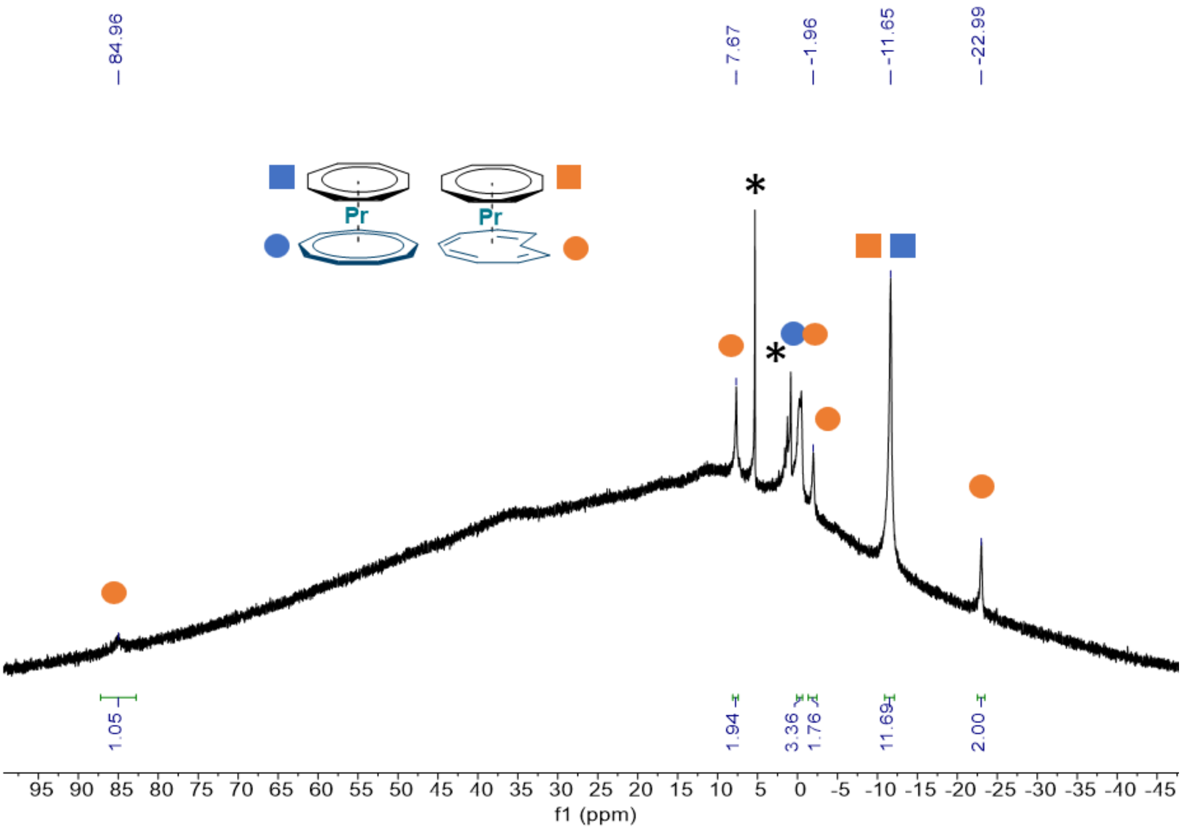

Figure S14: *^{1}H NMR of **2**-Pr-trans in CD_2Cl_2 measured at 293 K (* residual protio signal of the solvent).*

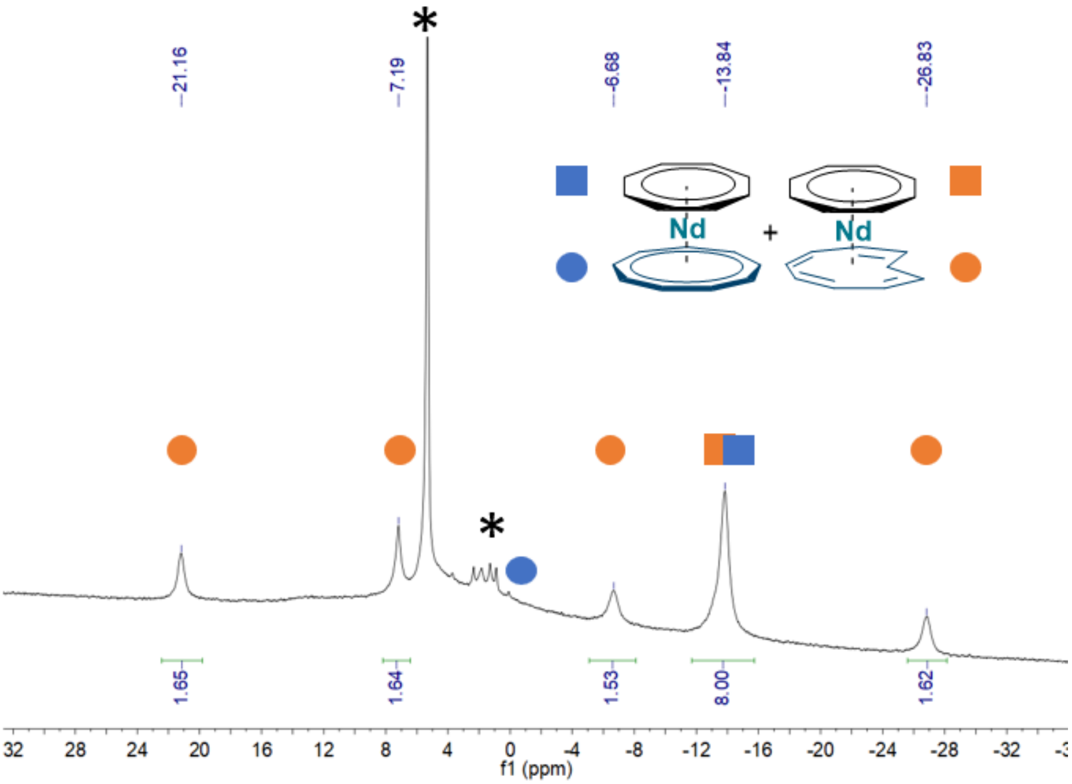

Figure S15: *^{1}H NMR of **2**-Nd-trans in CD_2Cl_2 measured at 293 K (* residual protio signal of the solvent).*

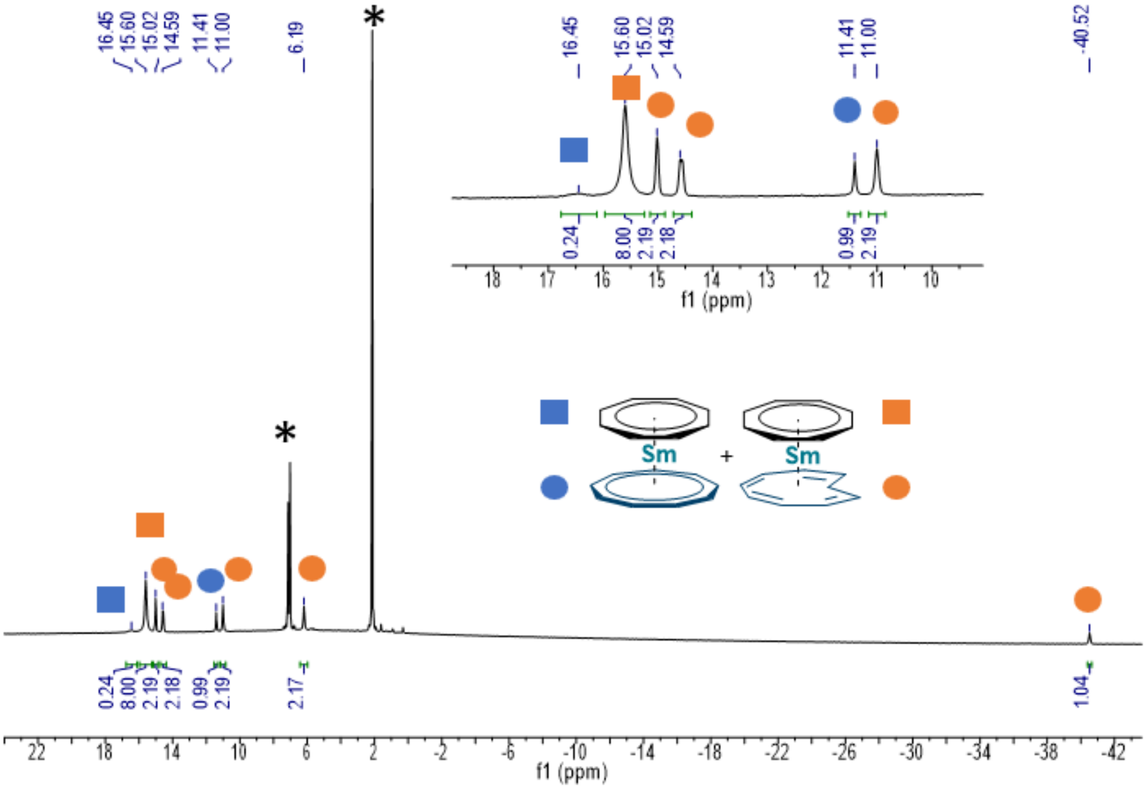

Figure S16: *^{1}H NMR of **2**-Sm-trans in CD_2Cl_2 measured at 293 K (* residual protio signal of the solvent).*

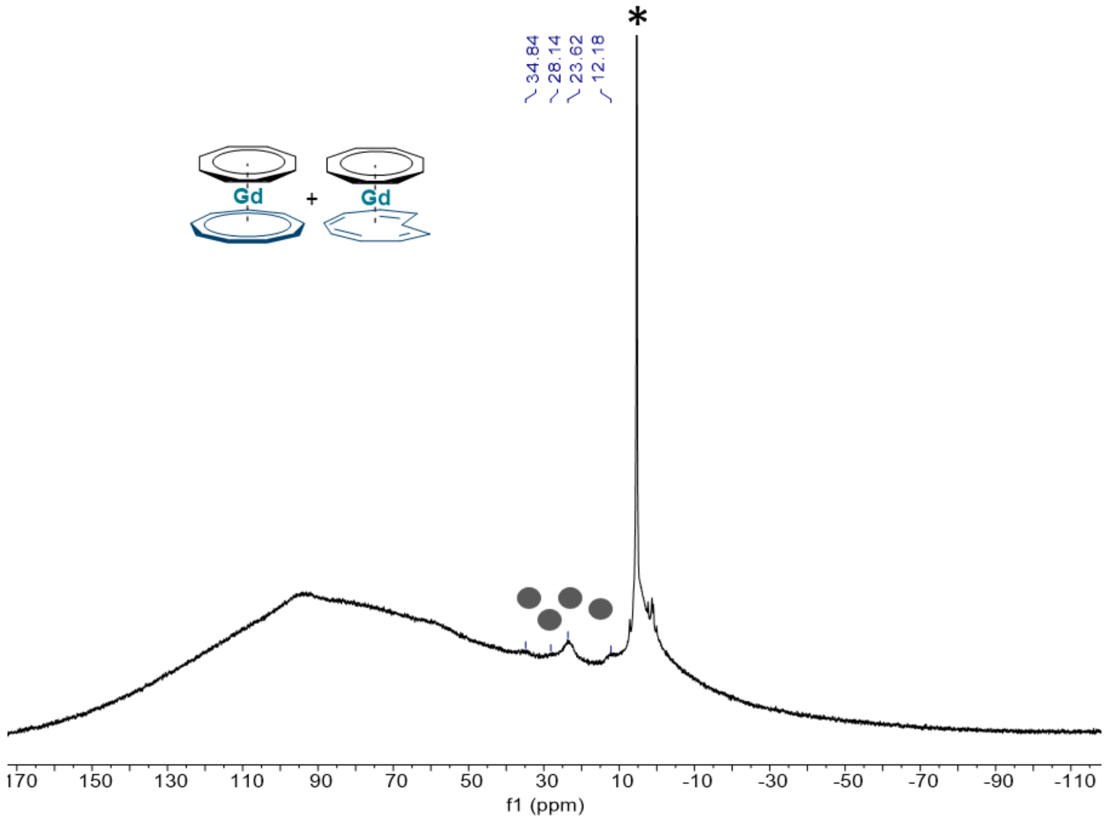

Figure S17: ¹H NMR of **2**-Gd-trans in CD_2Cl_2 measured at 293 K (* residual protio signal of the solvent). The uncertainty of integration does not allow the attribution.

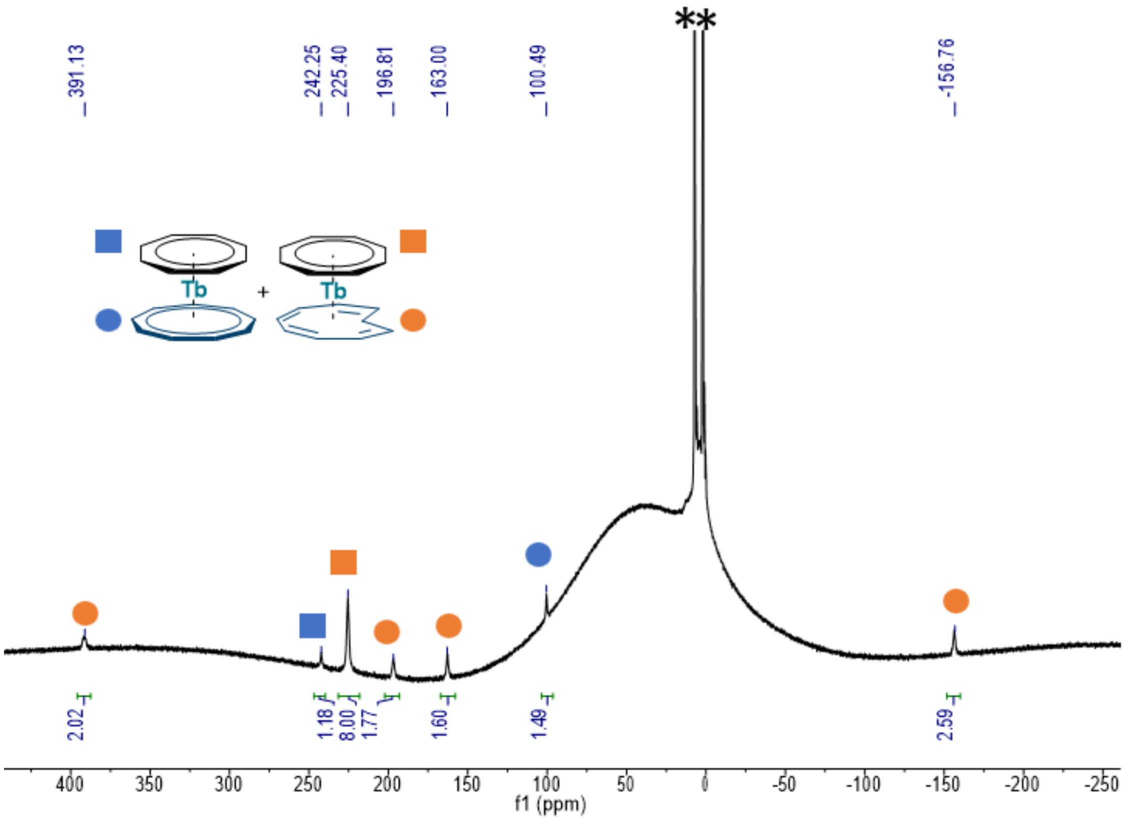

Figure S18: ¹H NMR of **2**-Tb-trans in toluene-d_8 measured at 293 K (* residual protio signal of the solvent).

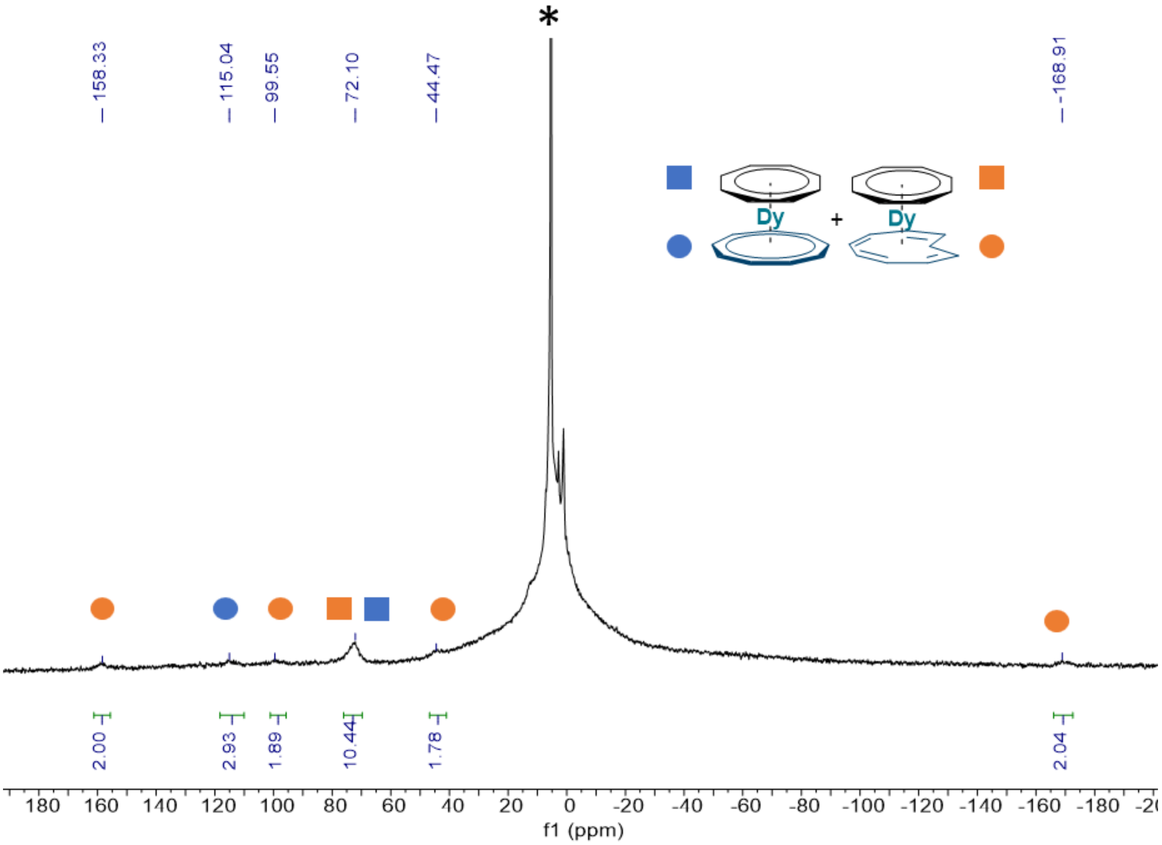

Figure S19: *^1H NMR of **2**-Dy-trans in CD_2Cl_2 measured at 293 K (* residual protio signal of the solvent).*

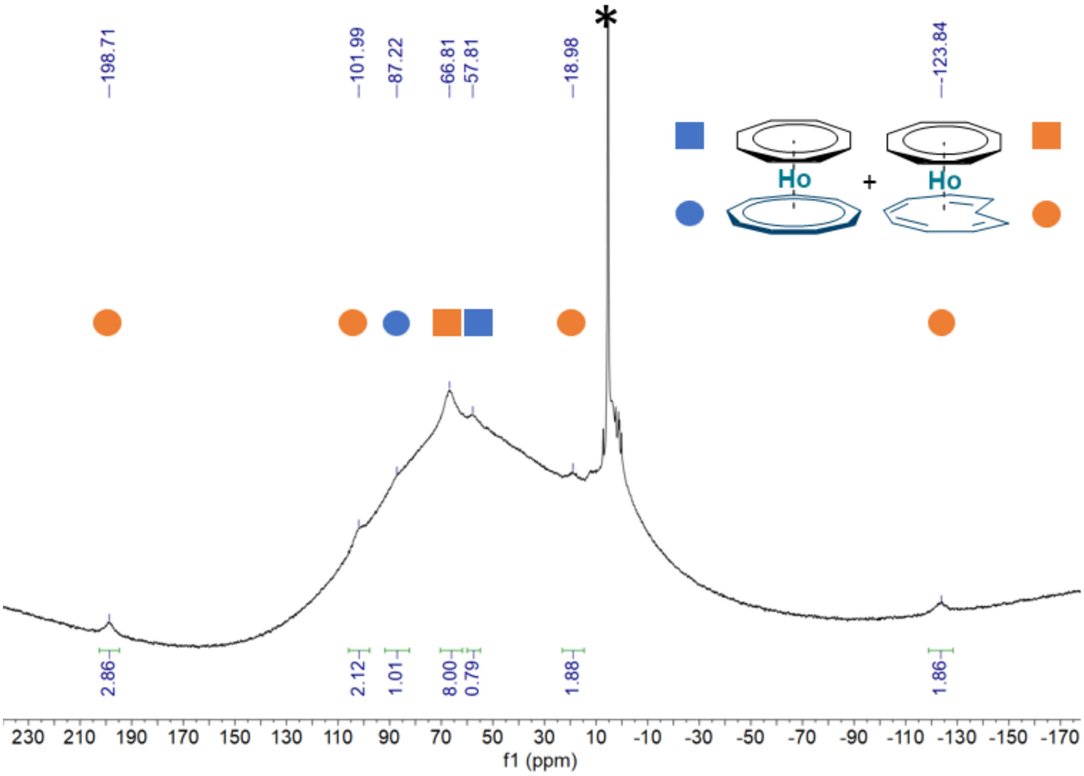

Figure S20: *^1H NMR of **2**-Ho-trans in CD_2Cl_2 measured at 293 K (* residual protio signal of the solvent).*

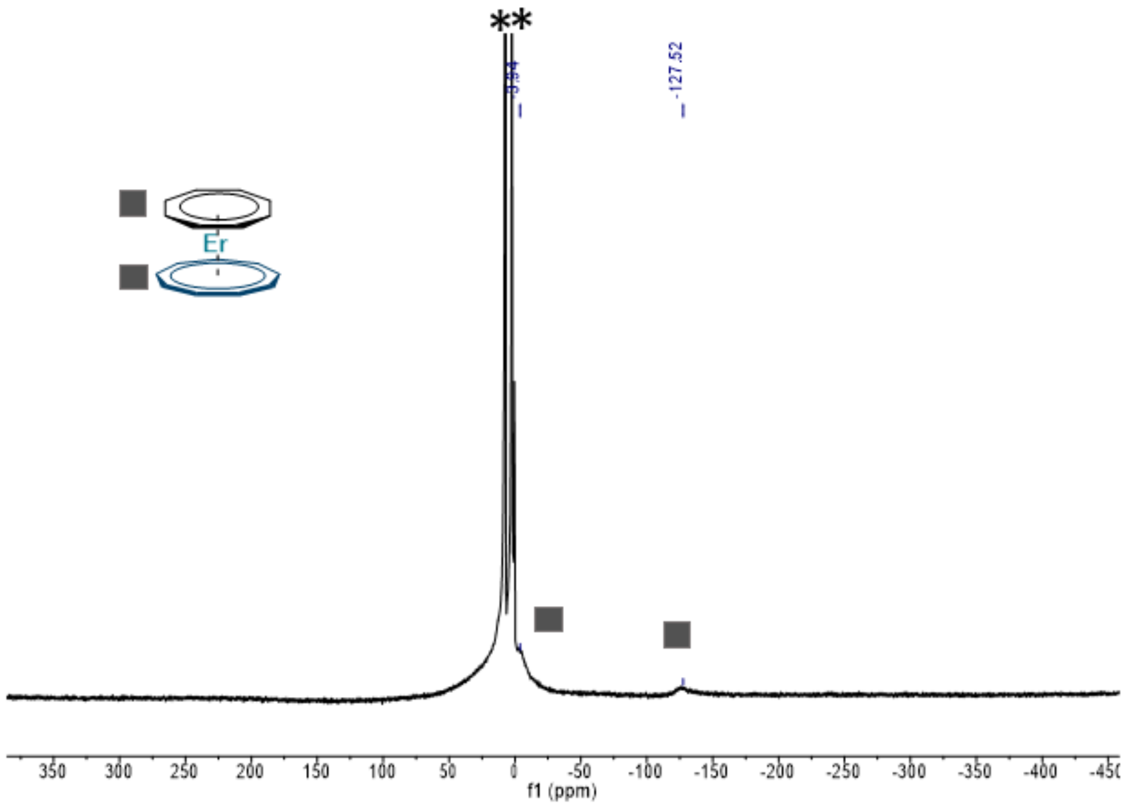

Figure S21: ^1H NMR of **2**-Er-cis in toluene-d_8 measured at 293 K (* residual protio signal of the solvent).

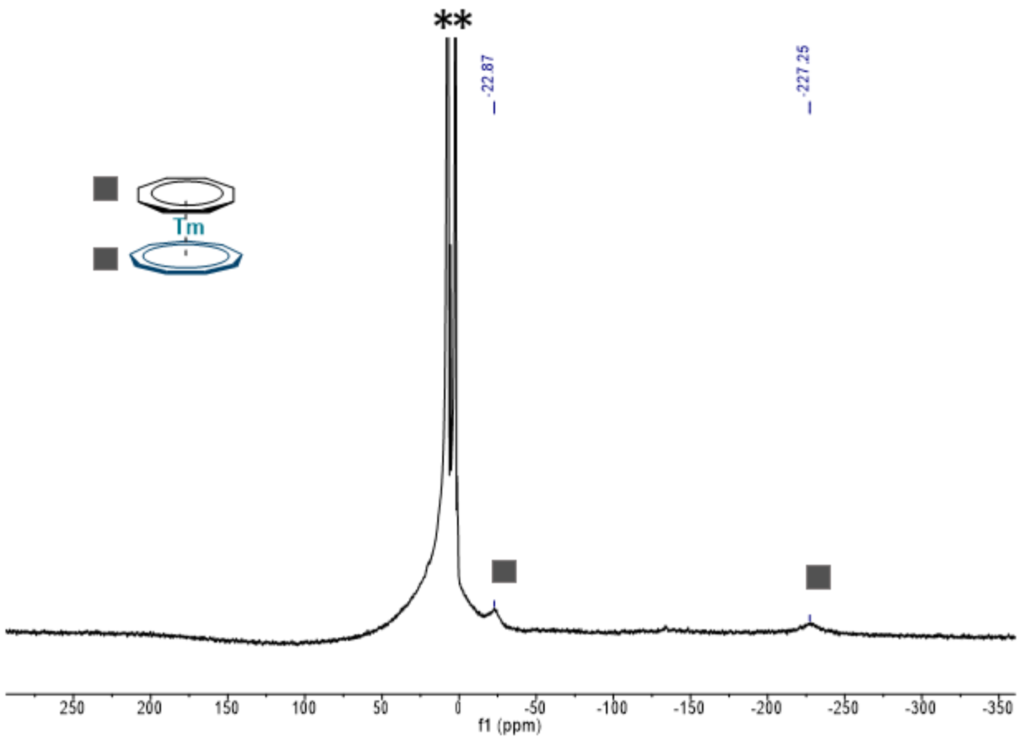

Figure S22: ^1H NMR of **2**-Tm-cis in toluene-d_8 measured at 293 K (* residual protio signal of the solvent).

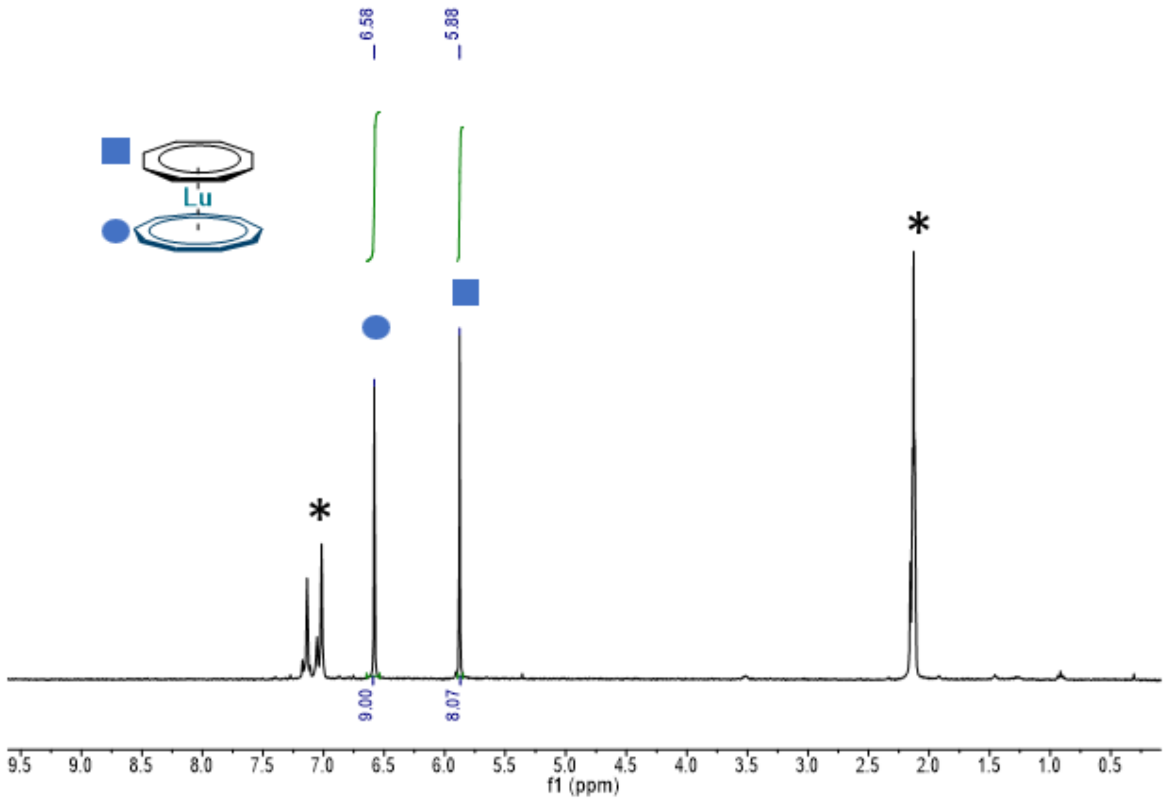

Figure S23: *^{1}H NMR of* ***2****-Lu-cis in toluene-d_8 measured at 293 K (* residual protio signal of the solvent).*

4. IR Spectra

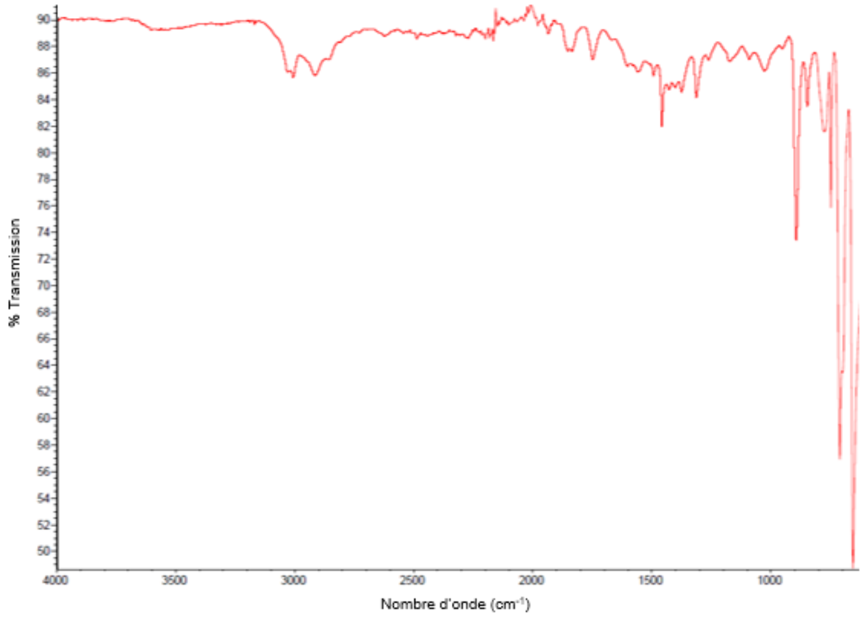

Figure S24: *IR spectrum of* ***2****-Tb-cis*

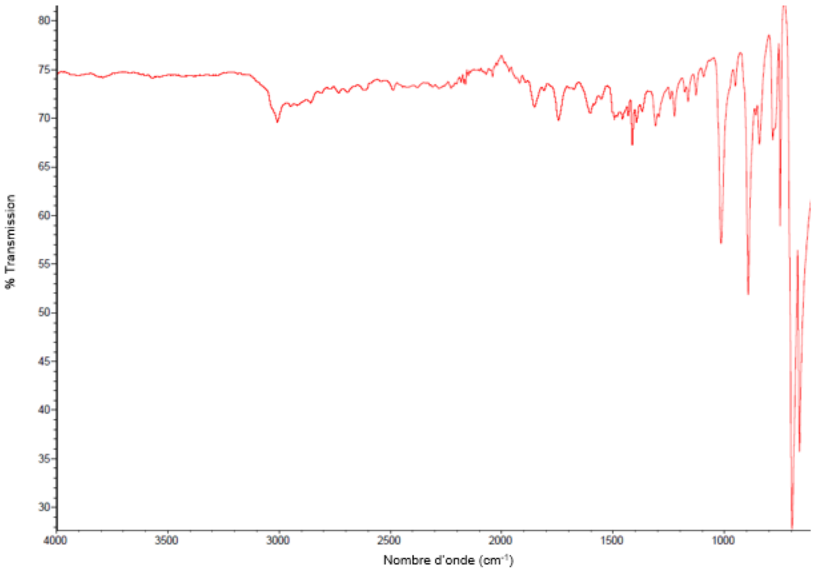

Figure S25: *IR spectrum of* ***2****-Tb-trans*

Table S1: *Summary of stretches observed in both isomers of* ***2****-Tb. The intensity of the signals is characterized by w = weak, m = medium, s = strong*

	**2**-Tb-*cis*	**2**-Tb-*trans*
Wavenumber (cm^{-1})	1457.20 (w)<br>892.06 (m)<br>709.59 (s)<br>654.30 (s)	1013.91 (m)<br>891.95 (m)<br>695.00 (s)<br>661.54 (s)

5. X-ray crystallography

General details. Single crystals of the complexes suitable for X-ray analysis were mounted on a Kapton loop using a Paratone N oil. Two diffractometers were used during this for the data acquisition, either a Bruker diffractometer equipped with an APEX II CCD detector and a graphite Mo-Kα monochromator or a Stoe stadivari diffractometer with a Eiger2 detector and a Mo-Kα microsource. All measurements were done at 150 K (unless otherwise stated) and a refinement method was used for solving the structure. The resolution of the solid-state structure was accomplished using the SHELXS-97 or SHELXT programs. The refinement was performed with the SHELXL program using the Olex2 software. All atoms – except hydrogens – were refined anisotropically.

Atoms denoted C(X') refer to equivalent atoms constructed by the symmetry operation of the associated space group. For the Cnt ligand, the terme Cnt – 8C refers to the 8 atoms of the Cnt ligand excluding the isomerized atoms.

The molecular structure of **2** display as the thermal ellipsoids that are depicted at 50% probability level. The center atom represents the corresponding Ln and are depicted with different colors and carbon atoms are in grey. Hydrogen atoms and disorder of the Cot and the Cnt ligand are omitted for clarity. The blue and red spots represent the constructed centroids for Cnt-*cis* and Cot respectively. The purple and orange spots represent the constructed centroids for Cnt – 8C and Cnt-*trans* respectively. Moreover, the data presents a disorder, typical for this family of complexes. The heavy Y atom is disordered over two positions, close to the symmetry element, resulting with hight calculated residual

electronic density. This peculiar disorder explains checkcif alerts about calculated residual electronic density. The C8 and C9 aromatic ligands are also disordered, resulting in low C-C bond precision.

For the modelisation of the **2-**Ln-*trans* three behaviors have been observed alongside the series. First for the late lanthanides the modelisation of a *trans*-Cnt allowed accurate portrayal of the electronic density showing very low amount of *cis*-Cnt present in the lattice. Then as demonstrated in Fig. S24, Gd and Sm did necessitate the modelisation of a disorder for one carbon atom between the *cis* and *trans* position. Finally, for La, Ce, Pr and Nd, two disordered and eclipsed Cnt rings were used to accurately represent the electronic density. One of them featuring a carbon in the *trans* position and the other one in the *cis* position as shown in Fig. S38-39.

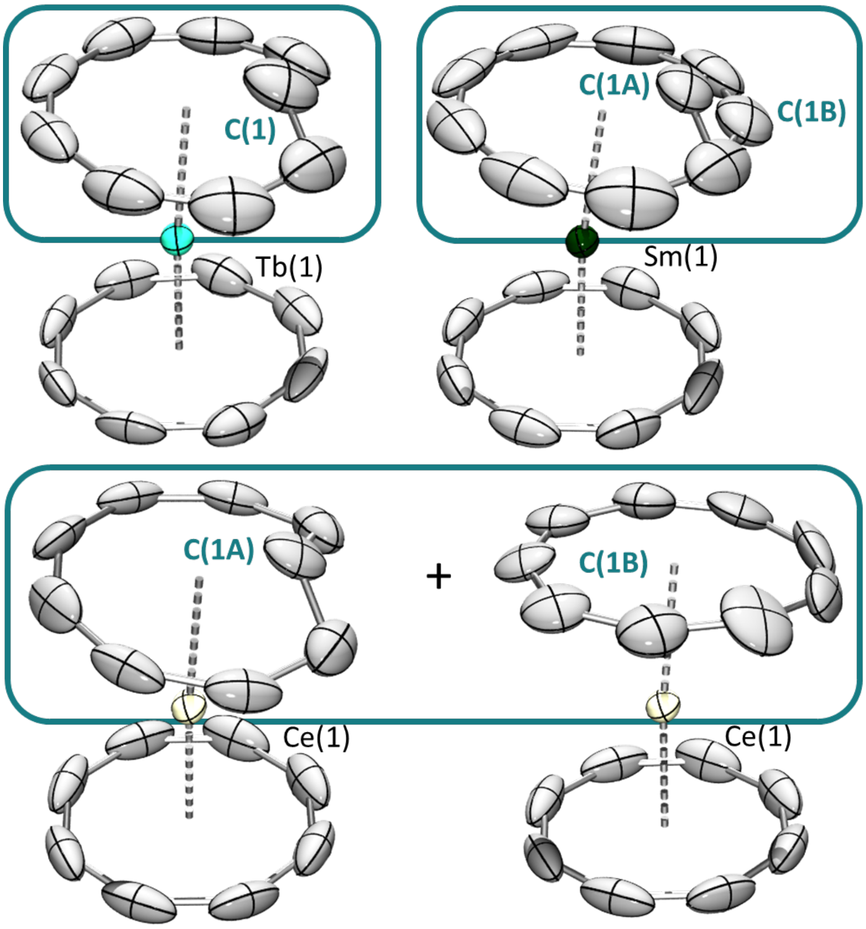

Figure S 26: Representation of the different model used for the LnCot(trans-Cnt) complexes

.

*Table S 2: Crystallographic data, details of data collection and structure refinement parameters of all **2***

**Compound**	**YCot(Cnt-*cis*)**	**LaCot(Cnt-*cis*)**	**CeCot(Cnt-*cis*)**
Formula	$C_{17}H_{17}Y$	$C_{17}H_{17}La$	$C_{17}H_{17}Ce$
Crystal size (mm^3)	0.15x0.04x0.02	0.14 x 0.06 x 0.03	0.2x0.02x0.02
Crystal system	Monoclinic	Orthorhombic	Orthorhombic
Space group	$P2_1/n$	Pnma	Pnma
Volume (Å^3)	671.15(10)	1392.9(6)	1381.6(3)
a (Å)	7.0169(6)	12.187(3)	12.1258(14)
b (Å)	8.7585(8)	12.736(3)	12.7752(16)
c (Å)	11.1560(9)	8.974(2)	8.9190(10)
α (deg)	90	90	90
β (deg)	101.791(6)	90	90
γ (deg)	90	90	90
Z	4	4	4
Formula weight (g/mol)	310.21	360.21	361.42
Density (calcd) (g/cm^3)	3.070	1.718	1.738
Absorption coefficient (mm^{-1})	8.645	3.046	3.273
F(000)	632.0	704.0	708.0
Temp (K)	150	150	150
diffractometer	Stoe Stadivari	Bruker APEX-II CCD	Bruker APEX-II CCD
Radiation	Mo Kα (λ = 0.71073)	MoKα (λ = 0.71073)	MoKα (λ = 0.71073)
2θ range for data collection (deg)	5.962 to 54.968	5.554 to 54.958	5.57 to 56.544
Absorption correction	Multi-scan	Multi-scan	Multi-scan
Total no. reflections	6569	21648	27170
Unique reflections [R_{int}]	1635 [R_{int} = 0.0446]	1674 [R_{int} = 0.1354]	1786 [R_{int} = 0.0965]
Final R indices [I>2σ(I)]	R_1 = 0.1254, wR_2 = 0.2668	R_1 = 0.0363, wR_2 = 0.0785	R_1 = 0.0267, wR_2 = 0.0562
R indices (all data)	R_1 = 0.1367, wR_2 = 0.2712	R_1 = 0.0879, wR_2 = 0.0980	R_1 = 0.0635, wR_2 = 0.0676
Largest diff. peak and hole (e.A^{-3})	3.30/-4.35	0.72/-1.06	0.65/-0.76
GooF	1.171	1.011	1.009

**Compound**	**PrCot(Cnt-*cis*)**	**NdCot(Cnt-*cis*)**	**SmCot(Cnt-*cis*)**
Formula	$C_{17}H_{17}Pr$	$C_{17}H_{17}Nd$	$C_{17}H_{17}Sm$
Crystal size (mm^3)	0.12x0.03x0.03	0.24x0.03x0.02	-
Crystal system	Orthorhombic	Orthorhombic	Orthorhombic
Space group	Pnma	Pnma	Pnma
Volume ($Å^3$)	1365.3(8)	1368.1(14)	1356.78(15)
a (Å)	12.055(4)	12.0748(18)	11.9982(8)
b (Å)	12.777(5)	12.8129(19)	12.8760(8)
c (Å)	8.864(3)	8.8427(14)	8.7824(6)
α (deg)	90	90	90
β (deg)	90	90	90
γ (deg)	90	90	90
Z	4	4	4
Formula weight (g/mol)	362.21	365.54	371.65
Density (calcd) (g/cm^3)	1.762	1.775	1.819
Absorption coefficient (mm^{-1})	3.547	3.773	4.306
F(000)	712.0	716.0	724.0
Temp (K)	150	150	150
diffractometer	Bruker APEX-II CCD	Bruker APEX-II CCD	Bruker APEX-II CCD
Radiation	MoKα (λ = 0.71073)	MoKα (λ = 0.71073)	MoKα (λ = 0.71073)
2θ range for data collection (deg)	5.594 to 51.992	5.598 to 59.346	5.614 to 71.262
Absorption correction	Multi-scan	Multi-scan	Multi-scan
Total no. reflections	7946	28912	107017
Unique reflections [R_{int}]	1404 [R_{int} = 0.1682]	1986 [R_{int} = 0.1464]	3242 [R_{int} = 0.1536]
Final R indices [I>2σ(I)]	R_1 = 0.0528, wR_2 = 0.1097	R_1 = 0.0389, wR_2 = 0.0861	R_1 = 0.0324, wR_2 = 0.0679
R indices (all data)	R_1 = 0.1319, wR_2 = 0.1445	R_1 = 0.1015, wR_2 = 0.1087	R_1 = 0.0631, wR_2 = 0.0822
Largest diff. peak and hole ($e.A^{-3}$)	1.25/-1.22	1.96/-1.07	1.50/-1.41
GooF	1.007	1.022	1.009

**Compound**	**GdCot(Cnt-*cis*)**	**YCot(Cnt-*trans*)**	**LaCot(Cnt-*trans*)**
Formula	$C_{17}H_{17}Gd$	$C_{17}H_{17}Y$	$C_{17}H_{17}La$
Crystal size (mm^3)	0.14x0.03x0.03	0.17x0.08x0.04	0.12x0.06x0.03
Crystal system	Orthorhombic	Orthorhombic	Orthorhombic
Space group	Pnma	Pnma	Pnma
Volume (Å^3)	1348.0(3)	1335.5(3)	1385.3(2)
a (Å)	11.965(15)	11.7622(15)	12.1385(7)
b (Å)	12.9116(19)	13.1214(18)	12.7452(11)
c (Å)	8.7283(10)	8.6532(11)	8.9543(11)
α (deg)	90	90	90
β (deg)	90	90	90
γ (deg)	90	90	90
Z	4	4	4
Formula weight (g/mol)	378.55	310.21	360.21
Density (calcd) (g/cm^3)	1.865	1.543	1.727
Absorption coefficient (mm^{-1})	4.898	4.345	3.062
F(000)	732.0	632.0	704.0
Temp (K)	150	150	150
diffractometer	Bruker APEX-II CCD	Bruker APEX-II CCD	Stoe Stadivari
Radiation	MoKα (λ = 0.71073)	MoKα (λ = 0.71073)	MoKα (λ = 0.71073)
2θ range for data collection (deg)	5.634 to 54.954	5.64 to 55.28	5.56 to 58.68
Absorption correction	Multi-scan	Multi-scan	Multi-scan
Total no. reflections	8390	10027	14590
Unique reflections [R_{int}]	1618 [R_{int} = 0.1237]	1614 [R_{int} = 0.1667]	1840 [R_{int} = 0.0335]
Final R indices [I>2σ(I)]	R_1 = 0.0486, wR_2 = 0.0960	R_1 = 0.0536, wR_2 = 0.1128	R_1 = 0.0231, wR_2 = 0.0537
R indices (all data)	R_1 = 0.1100, wR_2 = 0.1171	R_1 = 0.1092, wR_2 = 0.1370	R_1 = 0.0378, wR_2 = 0.0554
Largest diff. peak and hole (e.A^{-3})	0.88/-1.18	0.55/-0.79	1.24/-0.41
GooF	0.997	1.017	0.918

**Compound**	**CeCot(Cnt-*trans*)**	**PrCot(Cnt-*trans*)**	**NdCot(Cnt-*trans*)**
Formula	$C_{17}H_{17}Ce$	$C_{17}H_{17}Pr$	$C_{17}H_{17}Nd$
Crystal size (mm^3)	0.118x0.03x0.24	0.083x0.049x0.032	0.2x0.04x0.02
Crystal system	Orthorhombic	Orthorhombic	Orthorhombic
Space group	Pnma	Pnma	Pnma
Volume (Å^3)	1381.8(2)	1369.48(16)	1370.2(5)
a (Å)	12.0523(9)	12.0063(7)	11.983(2)
b (Å)	12.8172(12)	12.8295(10)	12.877(3)
c (Å)	8.9448(10)	8.8907(6)	8.8927(17)
α (deg)	90	90	90
β (deg)	90	90	90
γ (deg)	90	90	90
Z	4	4	4
Formula weight (g/mol)	361.52	362.21	365.54
Density (calcd) (g/cm^3)	1.738	1.757	1.769
Absorption coefficient (mm^{-1})	3.273	3.536	3.762
F(000)	708.0	712.0	716.0
Temp (K)	150	150	150
diffractometer	Stoe Stadivari	Stoe Stadivari	Bruker APEX-II CCD
Radiation	MoKα (*λ* = 0.71073)	MoKα (*λ* = 0.71073)	MoKα (*λ* = 0.71073)
2θ range for data collection (deg)	5.554 to 61.224	5.574 to 54.198	5.568 to 52.292
Absorption correction	Multi-scan	Multi-scan	Multi-scan
Total no. reflections	12034	5122	15587
Unique reflections [R_{int}]	2056 [R_{int} = 0.0622]	1536 [R_{int} = 0.0542]	1435 [R_{int} = 0.1352]
Final R indices [I>2σ(I)]	R1 = 0.0328, wR2 = 0.0629	R_1 = 0.0342, wR_2 = 0.0608	R_1 = 0.0396, wR_2 = 0.0849
R indices (all data)	R1 = 0.0714, wR2 = 0.0688	R_1 = 0.0771, wR_2 = 0.0663	R_1 = 0.0885, wR_2 = 0.1043
Largest diff. peak and hole (e.A^{-3})	1.08/-0.71	0.97/-0.64	0.90/-1.89
GooF	0.917	0.788	1.047

**Compound**	**SmCot(Cnt-*trans*)**	**GdCot(Cnt-*trans*)**	**TbCot(Cnt-*trans*)**
Formula	$C_{17}H_{17}Sm$	$C_{17}H_{17}Gd$	$C_{17}H_{17}Tb$
Crystal size (mm^3)	0.47 x 0.287 x 0.07	0.087x0.045x0.032	0.14x0.04x0.04
Crystal system	Orthorhombic	Orthorhombic	Orthorhombic
Space group	Pnma	Pnma	Pnma
Volume (Å^3)	1355.13(14)	1348.36(15)	1335.5(13)
a (Å)	11.8943(7)	11.8553(8)	11.781(6)
b (Å)	12.9414(6)	13.0113(8)	13.017(8)
c (Å)	8.8036(6)	8.7412(6)	8.708(5)
α (deg)	90	90	90
β (deg)	90	90	90
γ (deg)	90	90	90
Z	4	4	4
Formula weight (g/mol)	371.65	378.55	380.22
Density (calcd) (g/cm^3)	1.822	1.865	1.891
Absorption coefficient (mm^{-1})	4.311	4.897	5.273
F(000)	724.0	732.0	736.0
Temp (K)	150	150	150
diffractometer	Stoe Stadivari	Stoe Stadivari	Bruker APEX-II CCD
Radiation	MoKα (*λ* = 0.71073)	MoKα (*λ* = 0.71073)	MoKα (λ = 0.71073)
2θ range for data collection (deg)	5.596 to 54.2	5.614 to 54.206	5.628 to 54.204
Absorption correction	Multi-scan	Multi-scan	Multi-scan
Total no. reflections	7609	6971	17144
Unique reflections [R_{int}]	1454 [R_{int} = 0.0533]	1552 [R_{int} = 0.0699]	1539 [R_{int} = 0.1491]
Final R indices [I>2σ(I)]	R1 = 0.0323, wR2 = 0.0697	R_1 = 0.0397, wR_2 = 0.0866	R_1 = 0.0447, wR_2 = 0.1093
R indices (all data)	R1 = 0.0561, wR2 = 0.0763	R_1 = 0.0643, wR_2 = 0.0915	R_1 = 0.0726, wR_2 = 0.1274
Largest diff. peak and hole (e.A^{-3})	0.93/-0.67	1.38/-0.86	1.60/-2.27
GooF	0.958	0.933	1.101

**Compound**	**DyCot(Cnt-*trans*)**	**HoCot(Cnt-*trans*)**	**$Sm(Cnt)_2$***
Formula	$C_{17}H_{17}Dy$	$C_{17}H_{17}Ho$	$C_{18}H_{18}Sm$
Crystal size (mm^3)	0.64x0.08x0.06	0.1x0.04x0.02	0.097 × 0.09 × 0.055
Crystal system	Orthorhombic	Orthorhombic	Monoclinic
Space group	Pnma	Pnma	$P2_1/n$
Volume ($Å^3$)	1336.6(2)	1336.0(2)	735.6(2)
a (Å)	11.8081(12)	11.7641(10)	7.3830(16)
b (Å)	13.057(10)	13.1227(12)	9.1133(11)
c (Å)	8.6690(9)	8.6544(9)	11.059(2)
α (deg)	90	90	90
β (deg)	90	90	98.630(16)
γ (deg)	90	90	90
Z	4	4	2
Formula weight (g/mol)	383.80	386.23	384.67
Density (calcd) (g/cm^3)	1.907	1.920	1.737
Absorption coefficient (mm^{-1})	5.568	5.900	3.974
F(000)	710.0	744.0	376.0
Temp (K)	150	150	150
diffractometer	Bruker APEX-II CCD	Bruker APEX-II CCD	Stoe Stadivari
Radiation	MoKα (λ = 0.71073)	MoKα (λ = 0.71073)	Mo Kα (λ = 0.71073)
2θ range for data collection (deg)	5.64 to 63.14	5.638 to 52.042	5.82 to 52.008
Absorption correction	Multi-scan	Multi-scan	Multi-scan
Total no. reflections	12046	18294	9911
Unique reflections [R_{int}]	2315 [R_{int} = 0.0701]	1376 [R_{int} = 0.1726]	1448 [R_{int} = 0.1487]
Final R indices [I>2σ(I)]	R_1 = 0.0384, wR_2 = 0.0776	R_1 = 0.0379, wR_2 = 0.0640	R_1 = 0.0959, wR_2 = 0.2434
R indices (all data)	R_1 = 0.0550, wR_2 = 0.0844	R_1 = 0.0615, wR_2 = 0.0677	R_1 = 0.1172, wR_2 = 0.2613
Largest diff. peak and hole ($e.A^{-3}$)	1.52/-1.68	1.10/-1.11	5.14/-2.66
GooF	1.082	0.842	1.004

**cis:trans* ratio of 18:82

***Table S3**: Main metrics parameters for the **2** (trans and cis isomer). The carbon in trans formation is written in red. a) metrics taken from litterature[2]*

	Y		La		Ce		Pr		Nd		Sm	
	trans	cis	trans	cis	trans	cis	trans	cis	trans	cis	trans	cis
Ln - C(Cnt)	2.657(10)	2.87(2)	2.851(16)	2.93(2)	2.750(12)	2.891(13)	2.749(15)	2.89(4)	2.79(2)	2.84(1)	2.687(13)	2.824(9)
	2.827(9)	2.58(2)	2.931(8)	2.944(14)	2.878(15)	2.933(8)	2.87(2)	2.91(2)	2.89(2)	2.89(1)	2.857(9)	2.873(6)
	2.894(9)	2.51(2)	2.913(8)	2.971(13)	2.900(15)	2.942(8)	2.87(2)	2.93(2)	2.91(2)	2.92(1)	2.879(9)	2.892(7)
	2.812(9)	2.60(2)	2.901(8)	2.952(13)	2.887(15)	2.925(8)	2.88(2)	2.90(4)	2.85(2)	2.90(1)	2.834(9)	2.878(7)
	2.692(8)	2.81(3)	2.873(8)	2.927(13)	2.857(15)	2.905(8)	2.815(16)	2.85(2)	2.81(2)	2.86(1)	2.763(6)	2.831(6)
	-	3.13(3)	-	-	-	-	-	-	-	-	-	-
	-	3.53(2)	-	-	-	-	-	-	-	-	-	-
	-	3.60(2)	-	-	-	-	-	-	-	-	-	-
	-	3.30(2)	-	-	-	-	-	-	-	-	-	-
Ln-C(Cot)	2.524(7)	2.63(2)	2.686(4)	2.699(11)	2.640(4)	2.689(7)	2.637(5)	2.67(2)	2.619(8)	2.66(1)	2.594(5)	2.624(6)
	2.554(8)	2.46(3)	2.693(4)	2.694(12)	2.657(4)	2.675(8)	2.631(6)	2.65(2)	2.614(9)	2.65(1)	2.591(6)	2.627(7)
	2.554(8)	2.50(2)	2.693(4)	2.700(13)	2.656(4)	2.684(8)	2.639(6)	2.64(2)	2.641(9)	2.64(1)	2.601(6)	2.629(7)
	2.536(7)	2.43(2)	2.703(4)	2.686(13)	2.664(4)	2.657(8)	2.637(5)	2.64(2)	2.629(9)	2.64(1)	2.595(6)	2.618(6)
	-	2.54(2)	-	-	-	-	-	-	-	-	-	-
	-	2.61(2)	-	-	-	-	-	-	-	-	-	-
	-	2.48(3)	-	-	-	-	-	-	-	-	-	-
	-	2.65(2)	-	-	-	-	-	-	-	-	-	-
Plane (Cot) ^Plane (Cnt)	-	21.26 °	-	3.69 °	-	3.75°	-	3.86 °	-	3.62 °	-	3.02 °
Cent-Cot - Ln - Cent-Cnt	-	170.67 °	-	176.86 °	-	177.01 °	-	177.39 °	-	176.70 °	-	177.17 °
Ln-C(Cot) average	2.542(8)	2.54(2)	2.694(4)	2.695(12)	2.654(4)	2.676(8)	2.636(6)	2.65(1)	2.625(9)	2.65(1)	2.595(6)	2.624(7)
Ln-C(Cnt-C8) average	2.806(9)	-	2.905(8)	-	2.881(15)	-	2.86(2)	-	2.87(2)	-	2.833(8)	-
Ln-C(Cnt) average	2.776(9)	2.75(2)	2.894(10)	2.944(15)	2.85(1)	2.91(1)	2.84(2)	2.90(2)	2.85(2)	2.88(1)	2.804(9)	2.860(7)

	Gd		Tb		Dy		Ho		Er	Tm	Lu
	trans	cis	trans	cis[a]	trans	cis[a]	trans	cis[a]	cis[a]	cis[a]	cis[a]
Ln - C(Cnt)	2.703(17)	2.77(3)	2.664(16)	2.78(2)	2.660(9)	2.675(17)	2.673(11)	2.56(2)	2.55(3)	2.54(3)	2.50(2)
	2.853((10))	2.817(18)	2.837(12)	2.839(16)	2.825(9)	2.723(16)	2.819(9)	2.61(2)	2.59(3)	2.57(2)	2.54(2)
	2.872(9)	2.873(19)	2.885(12)	2.874(15)	2.858(9)	2.738(16)	2.86(1)	2.821(16)	2.61(3)	2.64(2)	2.6(2)
	2.828(10)	2.861(19)	2.828(12)	2.852(16)	2.806(6)	2.787(15)	2.805(8)	3.212(14)	2.881(16)	2.73(2)	2.68(2)
	2.749(9)	2.801(17)	2.722(10)	2.780(14)	2.720(6)	2.860(18)	2.684(7)	3.466(17)	3.443(13)	2.875(13)	2.943(12)
	-	-	-	-	-	2.953(18)	-	3.37(2)	3.763(13)	3.11(2)	3.14(2)
	-	-	-	-	-	2.959(17)	-	3.02(3)	3.597(15)	3.449(9)	3.59(10)
	-	-	-	-	-	2.857(16)	-	2.72(3)	3.10(2)	3.594(12)	3.719(13)
	-	-	-	-	-	2.736(16)	-	2.62(2)	2.69(3)	3.769(8)	3.930(9)
Ln-C(Cot)	2.559(7)	2.607(15)	2.537(9)	2.573(14)	2.534(5)	2.52(2)	2.518(7)	2.493(15)	2.470(13)	2.467(8)	2.461(10)
	2.575(7)	2.630(17)	2.563(11)	2.591(16)	2.544(5)	2.560(16)	2.529(8)	2.519(14)	2.499(15)	2.479(12)	2.457(12)
	2.582(6)	2.613(17)	2.566(9)	2.586(14)	2.543(5)	2.611(16)	2.524(7)	2.580(16)	2.50(12)	2.46(2)	2.465(17)
	2.565(7)	2.586(15)	2.540(9)	2.573(13)	2.544(5)	2.639(16)	2.534(6)	2.652(18)	2.55(3)	2.54(3)	2.495(19)
	-	-	-	-	-	2.600(16)	-	2.61(2)	2.51(3)	2.50(3)	2.45(2)
	-	-	-	-	-	2.615(15)	-	2.55(3)	2.52(3)	2.43(3)	2.44(2)
	-	-	-	-	-	2.590(15)	-	2.47(4)	2.45(3)	2.45(2)	2.47(2)
	-	-	-	-	-	2.536(17)	-	2.464(19)	2.448(16)	2.456(12)	2.468(13)
Plane (Cot) ^Plane (Cnt)	-	2.53 °	-	4.80 °	-	12.5 °	-	26.7 °	29.7 °	31.3 °	34.2 °
Cent-Cot - Ln - Cent-Cnt	-	177.34 °	-	177.4 °	-	172.0 °	-	169.6 °	174.7 °	173.8 °	174.2 °
Ln-C(Cot) average	2.570(7)	2.609(16)	2.552(10)	2.58(2)	2.541(5)	2.58(4)	2.478(7)	2.55(7)	2.50(4)	22.48(3)	2.46(1)
Ln-C(Cnt-C8) average	2.826(9)	-	2.818(11)	-	2.802(8)	-	2.792(9)	-	-	-	-
Ln-C(Cnt) average	2.801(11)	2.82(2)	2.787(13)	2.82(3)	2.774(8)	2.81(10)	2.768(9)	2.93(33)	2.74(21)	2.74(21)	2.73(26)

Table S4: *Main metrics for the* ***2****-Ln-trans. The carbon in trans formation is written in red.*

	Y	La	Ce	Pr	Nd	Sm	Gd	Tb	Dy	Ho
	trans	trans	trans	trans	trans	trans	trans	trans	trans	trans
Ln - C(Cnt)	2.657(10)	2.851(16)	2.750(12)	2.749(15)	2.79(2)	2.687(13)	2.703(17)	2.664(16)	2.660(9)	2.673(11)
	2.827(9)	2.931(8)	2.878(15)	2.87(2)	2.89(2)	2.857(9)	2.853(10)	2.837(12)	2.825(9)	2.819(9)
	2.894(9)	2.913(8)	2.900(15)	2.87(2)	2.91(2)	2.879(9)	2.872(9)	2.885(12)	2.858(9)	2.86(1)
	2.812(9)	2.901(8)	2.887(15)	2.88(2)	2.85(2)	2.834(9)	2.828(10)	2.828(12)	2.806(6)	2.805(8)
	2.692(8)	2.873(8)	2.857(15)	2.815(16)	2.81(2)	2.763(6)	2.749(9)	2.722(10)	2.720(6)	2.684(7)
Ln-C(Cot)	2.524(7)	2.686(4)	2.640(4)	2.637(5)	2.619(8)	2.594(5)	2.559(7)	2.537(9)	2.534(5)	2.518(7)
	2.554(8)	2.693(4)	2.657(4)	2.631(6)	2.614(9)	2.591(6)	2.575(7)	2.563(11)	2.544(5)	2.529(8)
	2.554(8)	2.693(4)	2.656(4)	2.639(6)	2.641(9)	2.601(6)	2.582(6)	2.566(9)	2.543(5)	2.524(7)
	2.536(7)	2.703(4)	2.664(4)	2.637(5)	2.629(9)	2.595(6)	2.565(7)	2.540(9)	2.544(5)	2.534(6)
Ln-C(Cot) average	2.542(8)	2.694(4)	2.654(4)	2.636(6)	2.625(9)	2.595(6)	2.570(7)	2.552(10)	2.541(5)	2.478(7)
Ln-C(Cnt-C8) average	2.806(9)	2.905(8)	2.881(15)	2.86(2)	2.87(2)	2.833(8)	2.826(9)	2.818(11)	2.802(8)	2.792(9)
Ln-C(Cnt)all average	2.776(9)	2.894(10)	2.85(1)	2.84(2)	2.85(2)	2.804(9)	2.801(11)	2.787(13)	2.774(8)	2.768(9)

***Table S5**: Main metrics for the **2**-Ln-cis. a) Metrics taken from known literature*

	Y	La	Ce	Pr	Nd	Sm	Gd	Tb	Dy	Ho	Er	Tm	Lu
	cis	cis	cis	cis	cis	cis	cis	cis[a]	cis[a]	cis[a]	cis[a]	cis[a]	cis[a]
	2.87(2)	2.93(2)	2.891(13)	2.89(4)	2.84(1)	2.824(9)	2.77(3)	2.78(2)	2.675(17)	2.56(2)	2.55(3)	2.54(3)	2.50(2)
	2.58(2)	2.944(14)	2.933(8)	2.91(2)	2.89(1)	2.873(6)	2.817(18)	2.839(16)	2.723(16)	2.61(2)	2.59(3)	2.57(2)	2.54(2)
	2.51(2)	2.971(13)	2.942(8)	2.93(2)	2.92(1)	2.892(7)	2.873(19)	2.874(15)	2.738(16)	2.821(16)	2.61(3)	2.64(2)	2.6(2)
	2.60(2)	2.952(13)	2.925(8)	2.90(4)	2.90(1)	2.878(7)	2.861(19)	2.852(16)	2.787(15)	3.212(14)	2.881(16)	2.73(2)	2.68(2)
Ln - C(Cnt)	2.81(3)	2.927(13)	2.905(8)	2.85(2)	2.86(1)	2.831(6)	2.801(17)	2.780(14)	2.860(18)	3.466(17)	3.443(13)	2.875(13)	2.943(12)
	3.13(3)	-	-	-	-	-	-	-	2.953(18)	3.37(2)	3.763(13)	3.11(2)	3.14(2)
	3.53(2)	-	-	-	-	-	-	-	2.959(17)	3.02(3)	3.597(15)	3.449(9)	3.59(10)
	3.60(2)	-	-	-	-	-	-	-	2.857(16)	2.72(3)	3.10(2)	3.594(12)	3.719(13)
	3.30(2)	-	-	-	-	-	-	-	2.736(16)	2.62(2)	2.69(3)	3.769(8)	3.930(9)
	2.63(2)	2.699(11)	2.689(7)	2.67(2)	2.66(1)	2.624(6)	2.607(15)	2.573(14)	2.52(2)	2.493(15)	2.470(13)	2.467(8)	2.461(10)
	2.46(3)	2.694(12)	2.675(8)	2.65(2)	2.65(1)	2.627(7)	2.630(17)	2.591(16)	2.560(16)	2.519(14)	2.499(15)	2.479(12)	2.457(12)
	2.50(2)	2.700(13)	2.684(8)	2.64(2)	2.64(1)	2.629(7)	2.613(17)	2.586(14)	2.611(16)	2.580(16)	2.50(12)	2.46(2)	2.465(17)
Ln-C(Cot)	2.43(2)	2.686(13)	2.657(8)	2.64(2)	2.64(1)	2.618(6)	2.586(15)	2.573(13)	2.639(16)	2.652(18)	2.55(3)	2.54(3)	2.495(19)
	2.54(2)	-	-	-	-	-	-	-	2.600(16)	2.61(2)	2.51(3)	2.50(3)	2.45(2)
	2.61(2)	-	-	-	-	-	-	-	2.615(15)	2.55(3)	2.52(3)	2.43(3)	2.44(2)
	2.48(3)	-	-	-	-	-	-	-	2.590(15)	2.47(4)	2.45(3)	2.45(2)	2.47(2)
	2.65(2)	-	-	-	-	-	-	-	2.536(17)	2.464(19)	2.448(16)	2.456(12)	2.468(13)
Plane (Cot) ^Plane (Cnt)	21.26 °	3.69 °	3.75°	3.86 °	3.62 °	3.02 °	2.53 °	4.80 °	12.5 °	26.7 °	29.7 °	31.3 °	34.2 °
Cent-Cot - Ln - Cent-Cnt	170.67 °	176.86 °	177.01 °	177.39 °	176.70 °	177.17 °	177.34 °	177.4 °	172.0 °	169.6 °	174.7 °	173.8 °	174.2 °
Ln-C(Cot) average	2.54(2)	2.695(12)	2.676(8)	2.65(1)	2.65(1)	2.624(7)	2.609(16)	2.58(2)	2.58(4)	2.55(7)	2.50(4)	22.48(3)	2.46(1)
Ln-C(Cnt) average	2.75(2)	2.944(15)	2.91(1)	2.90(2)	2.88(1)	2.860(7)	2.82(2)	2.82(3)	2.81(10)	2.93(33)	2.74(21)	2.74(21)	2.73(26)

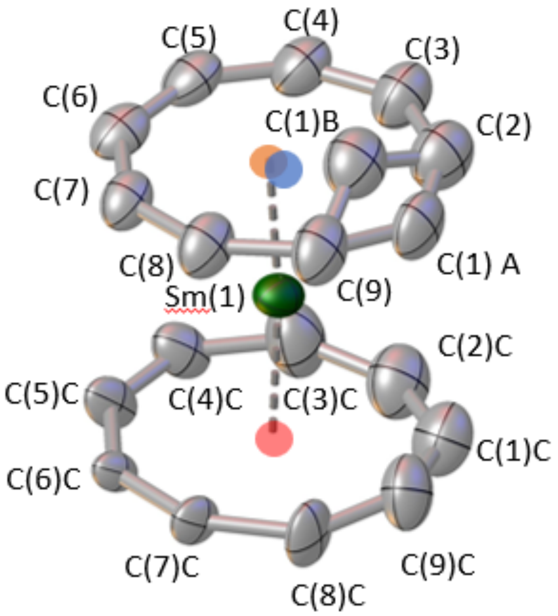

Figure S27: *Molecular structure of* ***1-trans*** *(18% trans ratio)*

Table S6: *Summary of bond length and angles from the ligands to the metal center in* ***1****.*

	Main distances in Å
Sm-C1C(Cnt)	2.83(4)
Sm – C2 (Cnt)	2.89(4)
Sm – C3 (Cnt)	2.92(3)
Sm – C4 (Cnt)	2.77(6)
Sm – C5 (Cnt)	2.90(4)
Sm– C6 (Cnt)	2.92(3)
Sm – C7 (Cnt)	2.95(2)
Sm – C8 (Cnt)	2.92(3)
Sm – C9 (Cnt)	2.92(4)
Sm – Cent (Cnt cis)	2.058 (12)
Sm – C1A (Cnt)	2.85(5)
Sm-C1B(Cnt)	2.72(7)
Sm – C2 (Cnt)	2.81(3)
Sm – C3 (Cnt)	2.83(3)
Sm – C4 (Cnt)	2.82(3)
Sm – C5 (Cnt)	2.82(3)
Sm– C6 (Cnt)	2.85(3)
Sm – C7 (Cnt)	2.84(3)
Sm – C8 (Cnt)	2.87(3)
Sm – C9 (Cnt)	2.87(4)
Sm – Cent (Cnt)	2,075 (13)
Sm – Cent (Cnt) –9C cis	2,022 (9)
Plane (Cnt cis) ^Plane (Cnt trans)	2,7
Cent-Cntcis -Sm- Cent-Cnttrans	176,2
Plane (C2/C1/C2[1]) ^Plane (Cnt 9C cis)	48

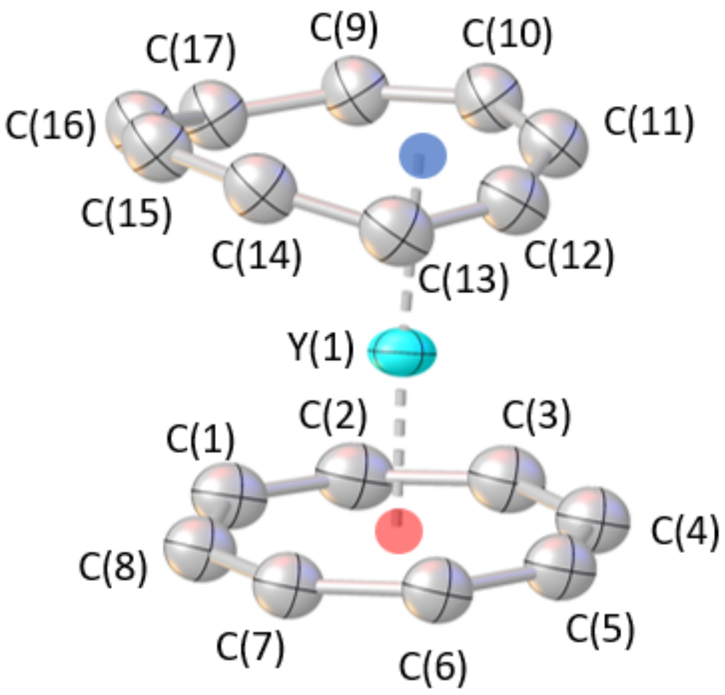

***Figure S28**: Molecular structure of **2**-Y-cis*

***Table S7**: Summary of bond length and angles from the ligands to the metal center.[1] Only the C coordinated to the metal are taken into account.*

	Main distances in Å
Y(1) – C(1) (Cot)	2.63(2)
Y(1) – C(2) (Cot)	2.46(3)
Y(1) – C(3) (Cot)	2.50(2)
Y(1) – C(4) (Cot)	2.43(2)
Y(1) – C(5) (Cot)	2.54(2)
Y(1) – C(6) (Cot)	2.61(2)
Y(1) – C(7) (Cot)	2.48(3)
Y(1) – C(8) (Cot)	2.65(2)
Y(1) – Cent (Cot)	1.734(9)
Y(1) – C(9) (Cnt)	2.87(2)
Y(1) – C(10) (Cnt)	2.58(2)
Y(1) – C(11) (Cnt)	2.51(2)
Y(1) – C(12) (Cnt)	2.60(2)
Y(1) – C(13) (Cnt)	2.81(3)
Y(1) – C(14) (Cnt)	3.13(3)
Y(1) – C(15) (Cnt)	3.53(2)
Y(1) – C(16) (Cnt)	3.60(2)
Y(1) – C(17) (Cnt)	3.30(2)
Y(1) – Cent (Cnt)[1]	2.081(11)
Plane (Cot) ^Plane (Cnt)[1]	21.26 °
Cent (Cot) - Y(1) – Cent (Cnt)[1]	170.67 °

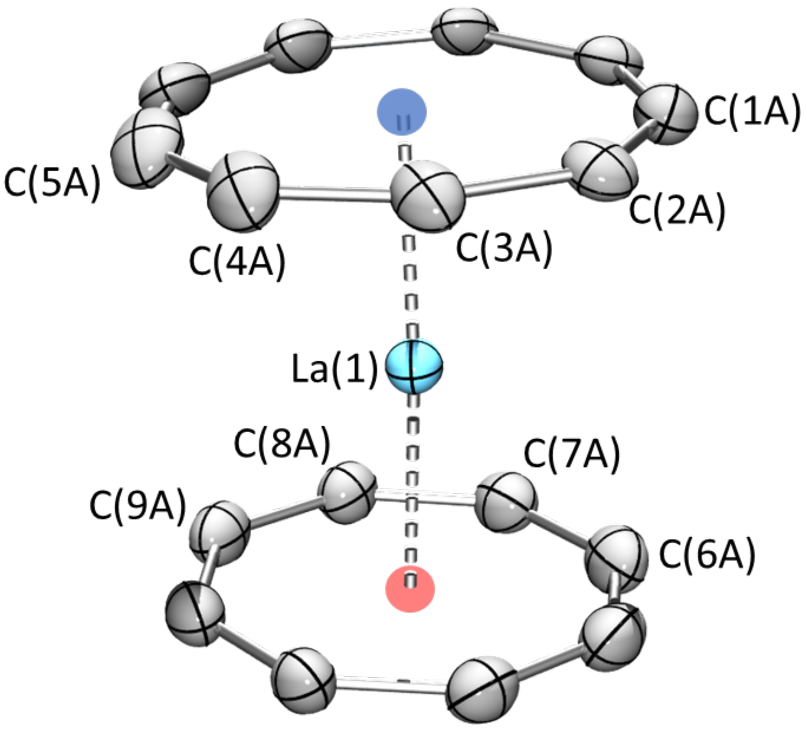

Figure S29: *Molecular structure of **2**-La-cis*

Table S8: *Summary of bond length and angles from the ligands to the metal center.*

	Main distances in Å
La(1) – C(6A) (Cot)	2.699(11)
La(1) – C(7A) (Cot)	2.694(12)
La(1) – C(8A) (Cot)	2.700(13)
La(1) – C(9A) (Cot)	2.686(13)
La(1) – Cent (Cot)	1.98
La(1) – C(1A) (Cnt)	2.93(2)
La(1) – C(2A) (Cnt)	2.944(14)
La(1) – C(3A) (Cnt)	2.971(13)
La(1) – C(4A) (Cnt)	2.952(13)
La(1) – C(5A) (Cnt)	2.927(13)
La(1) – Cent (Cnt)	2.14
Plane (Cot) ^Plane (Cnt)	3.69 °
Cent (Cot) - La(1) – Cent (Cnt)	176.86 °

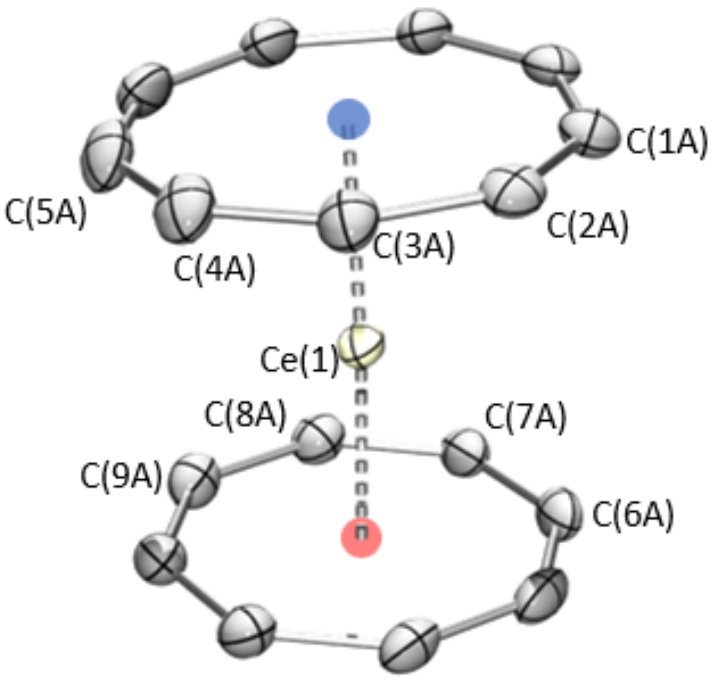

*Figure S 30: Molecular structure of **2**-Ce-cis*

***Table S9**: Summary of bond length and angles from the ligands to the metal center.*

	Main distances in Å
Ce(1) – C(6A) (Cot)	2.689(7)
Ce(1) – C(7A) (Cot)	2.675(8)
Ce(1) – C(8A) (Cot)	2.684(8)
Ce(1) – C(9A) (Cot)	2.657(8)
Ce(1) – Cent (Cot)	1.947
Ce(1) – C(1A) (Cnt)	2.891(13)
Ce(1) – C(2A) (Cnt)	2.933(8)
Ce(1) – C(3A) (Cnt)	2.942(8)
Ce(1) – C(4A) (Cnt)	2.925(8)
Ce(1) – C(5A) (Cnt)	2.905(8)
Ce(1) – Cent (Cnt)	2.095
Plane (Cot) ^Plane (Cnt)	3.75°
Cent (Cot) - Ce(1) – Cent (Cnt)	177.01°

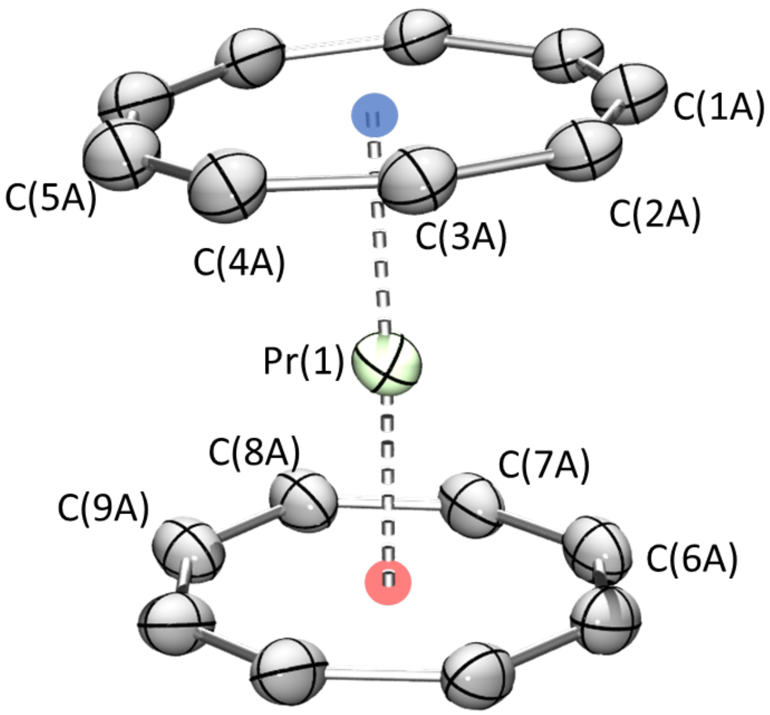

Figure S31: *Molecular structure of **2**-Pr-cis*

Table S10: *Summary of bond length and angles from the ligands to the metal center.*

	Main distances in Å
Pr(1) – C(6A) (Cot)	2.67(2)
Pr(1) – C(7A) (Cot)	2.65(2)
Pr(1) – C(8A) (Cot)	2.64(2)
Pr(1) – C(9A) (Cot)	2.64(2)
Pr(1) – Cent (Cot)	1.91
Pr(1) – C(1A) (Cnt)	2.89(4)
Pr(1) – C(2A) (Cnt)	2.91(2)
Pr(1) – C(3A) (Cnt)	2.93(2)
Pr(1) – C(4A) (Cnt)	2.90(4)
Pr(1) – C(5A) (Cnt)	2.85(2)
Pr(1) – Cent (Cnt)	2.07
Plane (Cot) ^Plane (Cnt)	3.86 °
Cent-Cot - Pr(1) - Cent-Cnt	177.39 °

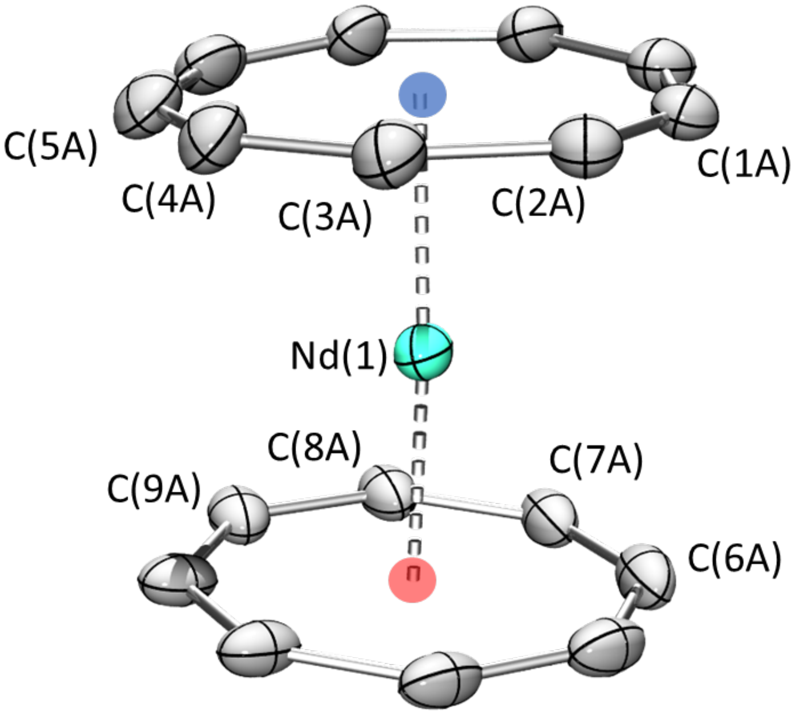

Figure S32: *Molecular structure of **2**-Nd-cis*

Table S11: *Summary of bond length and angles from the ligands to the metal center.*

	Main distances in Å
Nd(1) – C(6A) (Cot)	2.66(1)
Nd(1) – C(7A) (Cot)	2.65(1)
Nd(1) – C(8A) (Cot)	2.64(1)
Nd(1) – C(9A) (Cot)	2.64(1)
Nd(1) – Cent (Cot)	1.90
Nd(1) – C(1A) (Cnt)	2.84(1)
Nd(1) – C(2A) (Cnt)	2.89(1)
Nd(1) – C(3A) (Cnt)	2.92(1)
Nd(1) – C(4A) (Cnt)	2.90(1)
Nd(1) – C(5A) (Cnt)	2.86(1)
Nd(1) – Cent (Cnt)	2.05
Plane (Cot) ^Plane (Cnt)	3.62 °
Cent-Cot - Nd(1) - Cent-Cnt	176.70 °

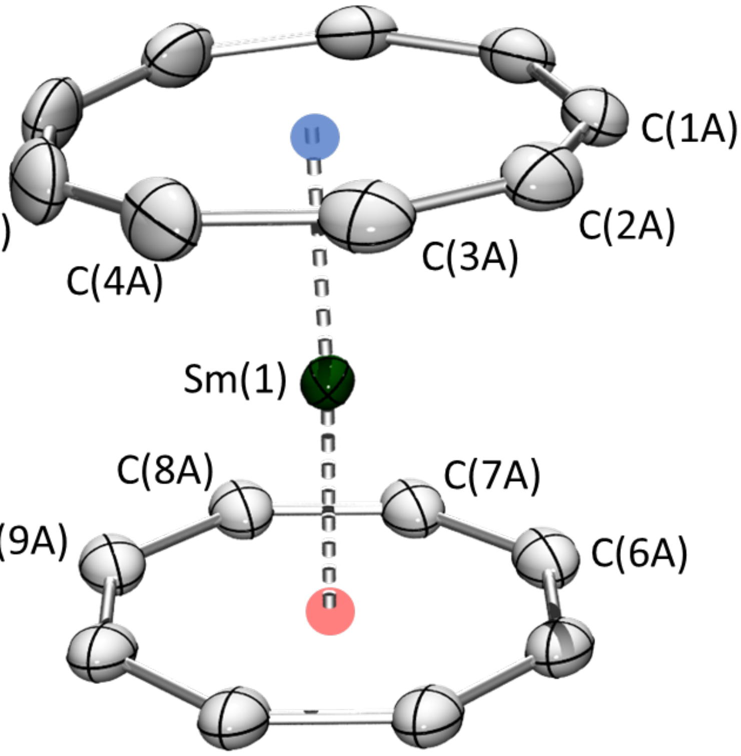

***Figure S33**: Molecular structure of **2**-Sm-cis*

***Table S12**: Summary of bond length and angles from the ligands to the metal center.*

	Main distances in Å
Sm(1) – C(6A) (Cot)	2.624(6)
Sm(1) – C(7A) (Cot)	2.627(7)
Sm(1) – C(8A) (Cot)	2.629(7)
Sm(1) – C(9A) (Cot)	2.618(6)
Sm(1) – Cent (Cot)	1.87
Sm(1) – C(1A) (Cnt)	2.824(9)
Sm(1) – C(2A) (Cnt)	2.873(6)
Sm(1) – C(3A) (Cnt)	2.892(7)
Sm(1) – C(4A) (Cnt)	2.878(7)
Sm(1) – C(5A) (Cnt)	2.831(6)
Sm(1) – Cent (Cnt)	2.01
Plane (Cot) ^Plane (Cnt)	3.02 °
Cent-Cot - Sm(1) - Cent-Cnt	177.17 °

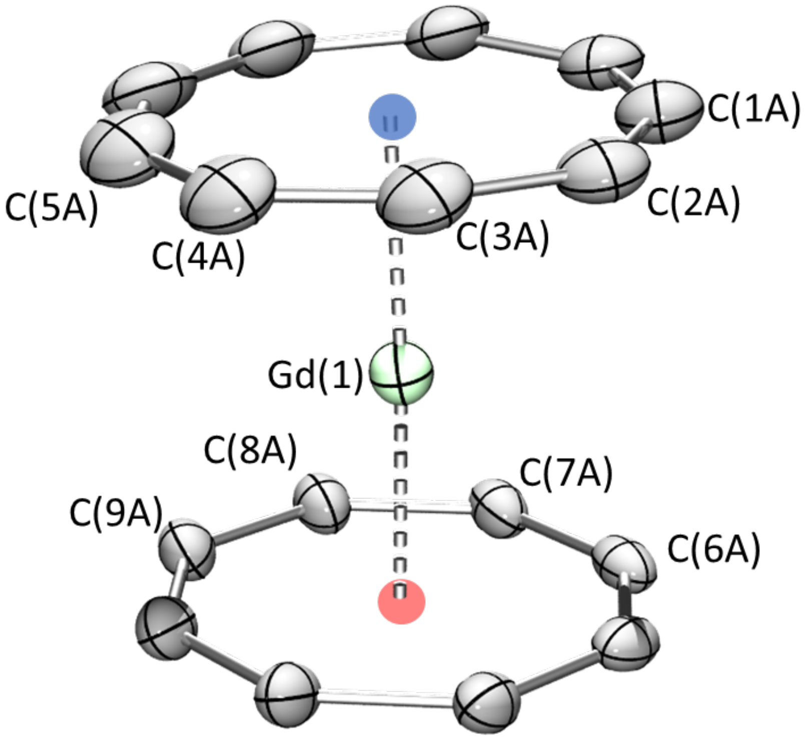

***Figure S34**: Molecular structure of **2**-Gd-cis*

***Table S13**: Summary of bond length and angles from the ligands to the metal center.*

	Main distances in Å
Gd(1) – C(6A) (Cot)	2.607(15)
Gd(1) – C(7A) (Cot)	2.630(17)
Gd(1) – C(8A) (Cot)	2.613(17)
Gd(1) – C(9A) (Cot)	2.586(15)
Gd(1) – Cent (Cot)	1.83
Gd(1) – C(1A) (Cnt)	2.77(3)
Gd(1) – C(2A) (Cnt)	2.817(18)
Gd(1) – C(3A) (Cnt)	2.873(19)
Gd(1) – C(4A) (Cnt)	2.861(19)
Gd(1) – C(5A) (Cnt)	2.801(17)
Gd(1) – Cent (Cnt)	2.00
Plane (Cot) ^Plane (Cnt)	2.53 °
Cent-Cot - Gd(1) - Cent-Cnt	177.34 °

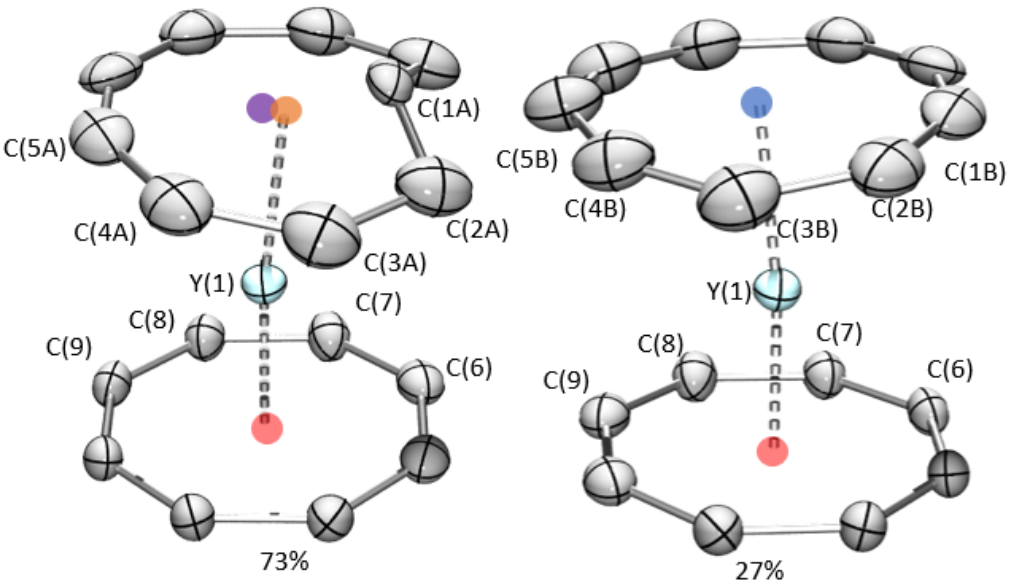

Figure S35: *Molecular structure of **2**-Y-trans (73% ratio).*

Table S14: *Summary of bond length and angles from the ligands to the metal center.*

	Main distances in Å
Y(1) – C(6) (Cot)	2.524(7)
Y(1) – C(7) (Cot)	2.554(8)
Y(1) – C(8) (Cot)	2.554(8)
Y(1) – C(9) (Cot)	2.536(7)
Y(1) – Cent (Cot)	1.758(4)
Y(1) – C(1A) (Cnt)	2.657(10)
Y(1) – C(1B) (Cnt)	2.66(6)
Y(1) – C(2A) (Cnt)	2.827(9)
Y(1) – C(2B) (Cnt)	2.83(3)
Y(1) – C(3A) (Cnt)	2.894(9)
Y(1) – C(3B) (Cnt)	2.93(2)
Y(1) – C(4A) (Cnt)	2.812(9)
Y(1) – C(4B) (Cnt)	3.00(2)
Y(1) – C(5A) (Cnt)	2.692(8)
Y(1) – C(5B) (Cnt)	2.85(2)
Y(1) – Cent (Cnt^{trans})	1.995(4)
Y(1) – Cent (Cnt^{cis})	2.050(13)
Y(1) – Cent (Cnt-8C)	1.948(5)
Plane (Cot) ^Plane (Cnt^{cis})	1.4 °
Plane (Cot) ^Plane (Cnt-8C)	4.3 °
Cent (Cot) - Y(1) – Cent (Cnt^{trans})	176.7 °
Cent (Cot) - Y(1) - Cent (Cnt^{cis})	175.9 °
Cent (Cot) - Y(1) - Cent (Cnt-8C)	179.1 °
Plane (C(2)/C(1)/C(2’)) ^Plane (Cnt^{cis})	47.9 °

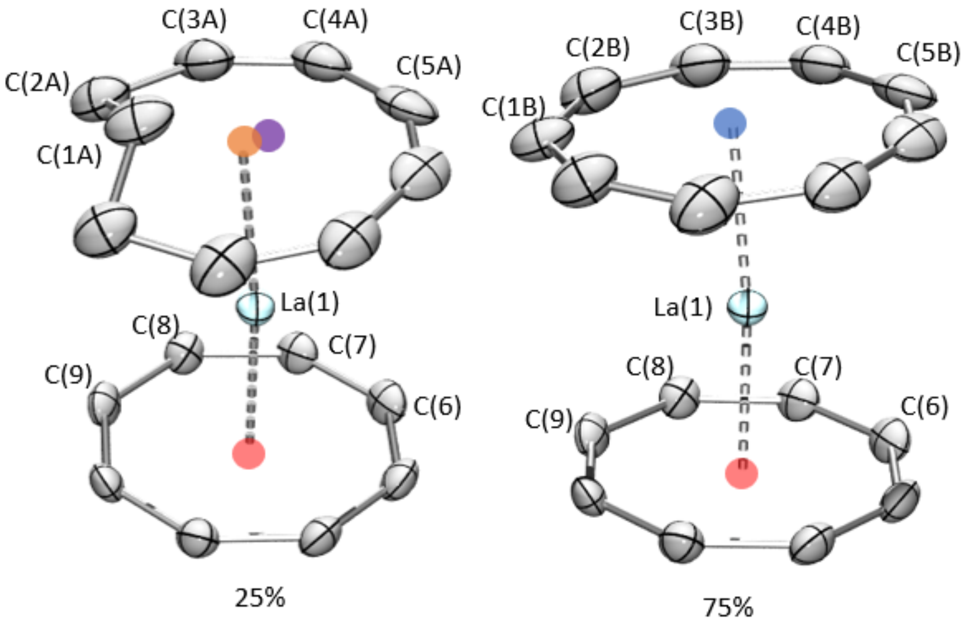

***Figure S36**: Molecular structure of **2**-La-trans (25% ratio).*

***Table S15**: Summary of bond length and angles from the ligands to the metal center.*

	Main distances in Å
La(1) – C(6) (Cot)	2.686(4)
La(1) – C(7) (Cot)	2.693(4)
La(1) – C(8) (Cot)	2.693(4)
La(1) – C(9) (Cot)	2.703(4)
La(1) – Cent (Cot)	1.970(2)
La(1) – C(1A) (Cnt)	2.851 (16)
La(1) – C(1B) (Cnt)	2.897(11)
La(1) – C(2A) (Cnt)	2.931(8)
La(1) – C(2B) (Cnt)	2.947(7)
La(1) – C(3A) (Cnt)	2.913(8)
La(1) – C(3B) (Cnt)	2.965(7)
La(1) – C(4A) (Cnt)	2.901(8)
La(1) – C(4B) (Cnt)	2.952(7)
La(1) – C(5A) (Cnt)	2.873(8)
La(1) – C(5B) (Cnt)	2.951(7)
La(1) – Cent (Cnt^{trans})	2.157(4)
La(1) – Cent (Cnt^{cis})	2.138(3)
La(1) – Cent (Cnt-8C)	2.110(4)
Plane (Cot) ^Plane (Cnt^{cis})	3.66 °
Plane (Cot) ^Plane (Cnt-8C)	5.03 °
Cent (Cot) - La(1) – Cent (Cnt^{trans})	177.13 °
Cent (Cot) - La(1) - Cent (Cnt^{cis})	176.25 °
Cent (Cot) - La(1) - Cent (Cnt-8C)	178.76 °
Plane (C(2)/C(1)/C(2’)) ^Plane (Cnt^{cis})	32.4 °

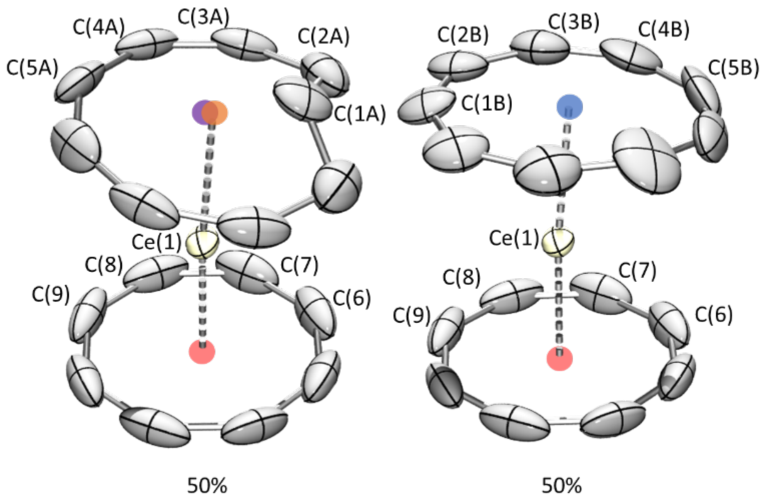

***Figure S37**: Molecular structure of **2**-Ce-trans* (50% ratio).

***Table S16**: Summary of bond length and angles from the ligands to the metal center.*

	Main distances in Å
Ce(1) – C(6) (Cot)	2.640(4)
Ce(1) – C(7) (Cot)	2.657(4)
Ce(1) – C(8) (Cot)	2.656(4)
Ce(1) – C(9) (Cot)	2.664(4)
Ce(1) – Cent (Cot)	1.94
Ce(1) – C(1A) (Cnt)	2.750(12)
Ce(1) – C(1B) (Cnt)	2.84(2)
Ce(1) – C(2A) (Cnt)	2.878(15)
Ce(1) – C(2B) (Cnt)	2.88(2)
Ce(1) – C(3A) (Cnt)	2.900(15)
Ce(1) – C(3B) (Cnt)	2.89(2)
Ce(1) – C(4A) (Cnt)	2.887(15)
Ce(1) – C(4B) (Cnt)	2.90(2)
Ce(1) – C(5A) (Cnt)	2.857(15)
Ce(1) – C(5B) (Cnt)	2.94(2)
Ce(1) – Cent (Cnt-*trans*)	2.08
Ce(1) – Cent (Cnt-*cis*)	2.14
Ce(1) – Cent (Cnt-8C)	2.04
Plane (Cot) ^Plane (Cnt-*cis*)	5.53 °
Plane (Cot) ^Plane (Cnt-8C)	3.39 °
Cent-Cot - Ce(1) - Cent-Cnt-*trans*	176.27 °
Cent-Cot - Ce(1)- Cent-Cnt-*cis*	176.41 °
Cent-Cot - Ce(1)- Cent-Cnt-8C	179.6 °
Plane (C(2)/C(1)/C(2’)) ^Plane (Cnt-*cis*)	5.53 °

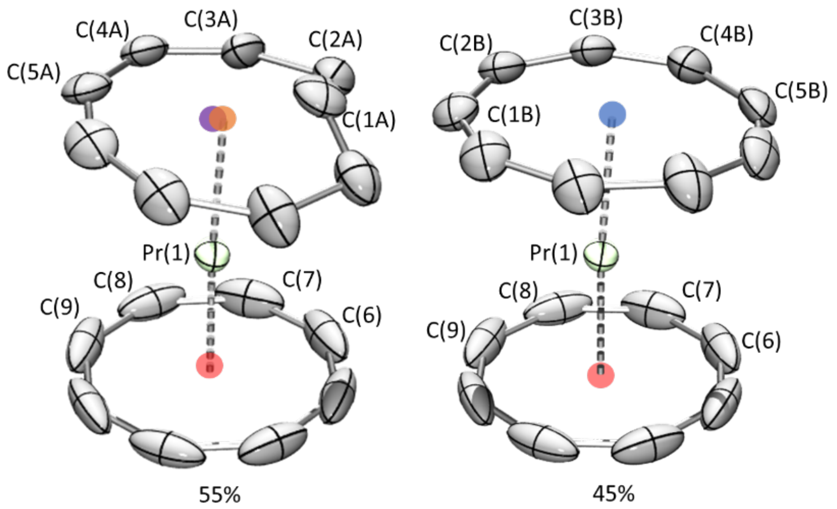

***Figure S38**: Molecular structure of **2**-Pr-trans (55% ratio).*

***Table S17**: Summary of bond length and angles from the ligands to the metal center.*

	Main distances in Å
Pr(1) – C(6) (Cot)	2.637(5)
Pr(1) – C(7) (Cot)	2.631(6)
Pr(1) – C(8) (Cot)	2.639(6)
Pr(1) – C(9) (Cot)	2.637(5)
Pr(1) – Cent (Cot)	1.91
Pr(1) – C(1A) (Cnt)	2.749(15)
Pr(1) – C(1B) (Cnt)	2.84(2)
Pr(1) – C(2A) (Cnt)	2.87(2)
Pr(1) – C(2B) (Cnt)	2.87(2)
Pr(1) – C(3A) (Cnt)	2.87(2)
Pr(1) – C(3B) (Cnt)	2.88(2)
Pr(1) – C(4A) (Cnt)	2.88(2)
Pr(1) – C(4B) (Cnt)	2.89(2)
Pr(1) – C(5A) (Cnt)	2.815(16)
Pr(1) – C(5B) (Cnt)	2.90(2)
Pr(1) – Cent (Cnt^{trans})	2.09
Pr(1) – Cent (Cnt^{cis})	2.08
Pr(1) – Cent (Cnt-8C)	2.04
Plane (Cot) ^Plane (Cnt^{cis})	5.12°
Plane (Cot) ^Plane (Cnt-8C)	2.88°
Cent (Cot) - Pr(1) – Cent (Cnt^{trans})	176.76°
Cent (Cot) - Pr(1)- Cent (Cnt^{cis})	176.16°
Cent (Cot) - Pr(1)- Cent (Cnt-8C)	178.99°
Plane (C(2)/C(1)/C(2')) ^Plane (Cnt^{cis})	47.68 °

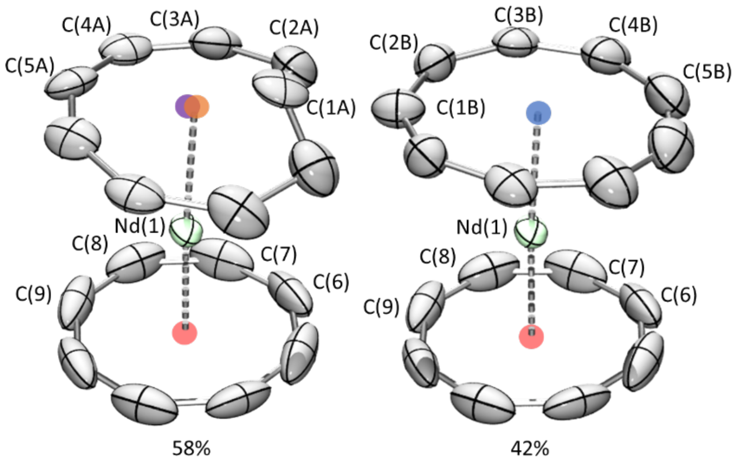

***Figure S39**: Molecular structure of **2**-Nd-trans (58% ratio).*

***Table S18**: Summary of bond length and angles from the ligands to the metal center.*

	Main distances in Å
Nd(1) – C(6) (Cot)	2.619(8)
Nd(1) – C(7) (Cot)	2.614(9)
Nd(1) – C(8) (Cot)	2.641(9)
Nd(1) – C(9) (Cot)	2.629(9)
Nd(1) – Cent (Cot)	1.90
Nd(1) – C(1A) (Cnt)	2.79(2)
Nd(1) – C(1B) (Cnt)	2.84(2)
Nd(1) – C(2A) (Cnt)	2.89(2)
Nd(1) – C(2B) (Cnt)	2.88(2)
Nd(1) – C(3A) (Cnt)	2.91(2)
Nd(1) – C(3B) (Cnt)	2.91(2)
Nd(1) – C(4A) (Cnt)	2.85(2)
Nd(1) – C(4B) (Cnt)	2.89(2)
Nd(1) – C(5A) (Cnt)	2.81(2)
Nd(1) – C(5B) (Cnt)	2.86(2)
Nd(1) – Cent (Cnt-*trans*)	2.08
Nd(1) – Cent (Cnt-*cis*)	2.06
Nd(1) – Cent (Cnt-8C)	2.03
Plane (Cot) ^Plane (Cnt-*cis*)	3.7 °
Plane (Cot) ^Plane (Cnt-8C)	4.44 °
Cent-Cot - Nd(1) - Cent-Cnt-*trans*	177.00 °
Cent-Cot - Nd(1)- Cent-Cnt-*cis*	176.8 °
Cent-Cot - Nd(1)- Cent-Cnt-8C	178.78 °
Plane (C(2)/C(1)/C(2’)) ^Plane (Cnt-*cis*)	50.15 °

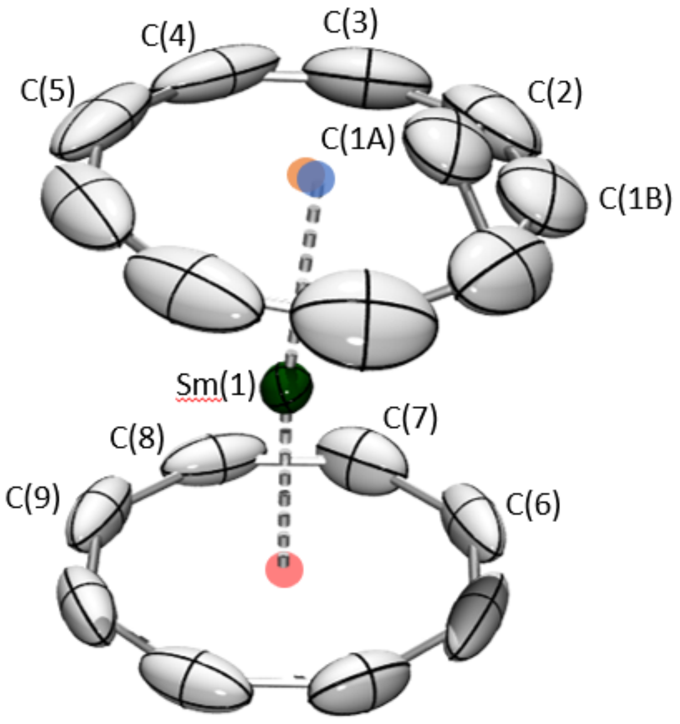

Figure S40: *Molecular structure of **2**-Sm-trans (69% ratio).*

Table S19: *Summary of bond length and angles from the ligands to the metal center.*

	Main distances in Å
Sm(1) – C(6) (Cot)	2.594(5)
Sm(1) – C(7) (Cot)	2.591(6)
Sm(1) – C(8) (Cot)	2.601(6)
Sm(1) – C(9) (Cot)	2.595(6)
Sm(1) – Cent (Cot)	1.85
Sm(1) – C(1A) (Cnt)	2.687(13)
Sm(1) – C(1B) (Cnt)	2.82(3)
Sm(1) – C(2) (Cnt)	2.857(9)
Sm(1) – C(3) (Cnt)	2.879(9)
Sm(1) – C(4) (Cnt)	2.834(9)
Sm(1) – C(5) (Cnt)	2.763(6)
Sm(1) – Cent (Cnt-*trans*)	2.054
Sm(1) – Cent (Cnt-*cis*)	2.01
Plane (Cot) ^Plane (Cnt-*cis*)	3.54 °
Cent-Cot - Sm(1) - Cent-Cnt-*trans*	175.96 °
Cent-Cot - Sm(1)- Cent-Cnt-*cis*	173.37 °
Plane (C(2)/C(1)/C(2’)) ^Plane (Cnt-*cis*)	42.80 °

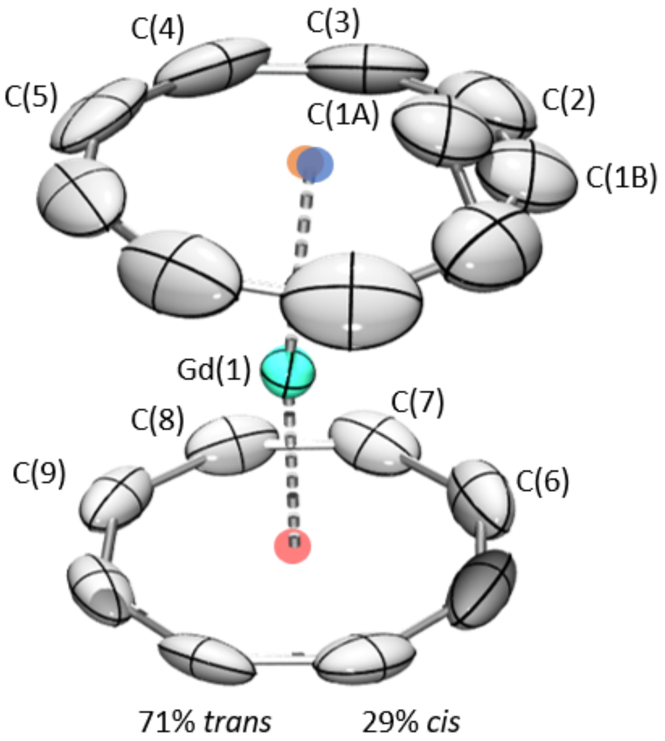

Figure S41: *Molecular structure of* ***2****-Gd-trans (71% ratio).*

Table S20: *Summary of bond length and angles from the ligands to the metal center.*

	Main distances in Å
Gd(1) – C(6) (Cot)	2.559(7)
Gd(1) – C(7) (Cot)	2.575(7)
Gd(1) – C(8) (Cot)	2.582(6)
Gd(1) – C(9) (Cot)	2.565(7)
Gd(1) – Cent (Cot)	1.82
Gd(1) – C(1A) (Cnt)	2.703(17)
Gd(1) – C(1B) (Cnt)	2.81(5)
Gd(1) – C(2) (Cnt)	2.853(10)
Gd(1) – C(3) (Cnt)	2.872(9)
Gd(1) – C(4) (Cnt)	2.828(10)
Gd(1) – C(5) (Cnt)	2.749(9)
Gd(1) – Cent (Cnt-*trans*)	2.04
Gd(1) – Cent (Cnt-*cis*)	2.003
Plane (Cot) ^Plane (Cnt-*cis*)	3.63 °
Cent-Cot - Gd(1) - Cent-Cnt-*trans*	175.04 °
Cent-Cot - Gd(1)- Cent-Cnt-*cis*	172.72 °
Plane (C(2)/C(1)/C(2')) ^Plane (Cnt-*cis*)	45.34 °

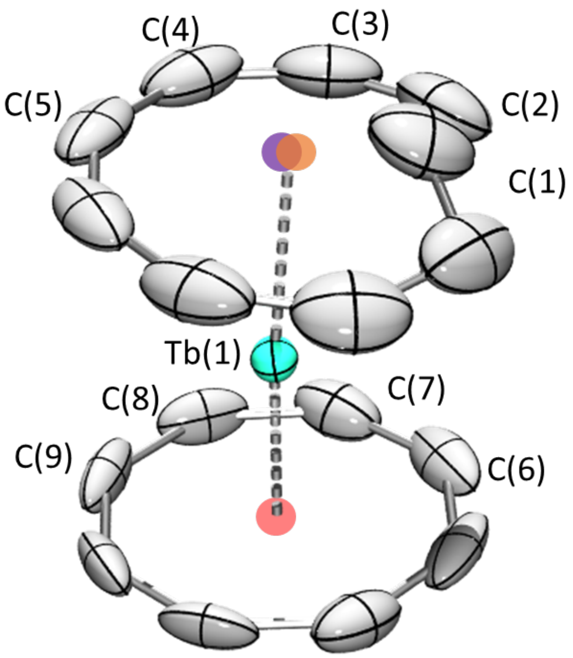

Figure S42: *Molecular structure of* ***2****-Tb-trans (100% ratio).*

Table S21*: Summary of bond length and angles from the ligands to the metal center.*

	Main distances in Å
Tb(1) – C(6) (Cot)	2.537(9)
Tb(1) – C(7) (Cot)	2.563(11)
Tb(1) – C(8) (Cot)	2.566(9)
Tb(1) – C(9) (Cot)	2.540(9)
Tb(1) – Cent (Cot)	1.80
Tb(1) – C(1) (Cnt)	2.664(16)
Tb(1) – C(2) (Cnt)	2.837(12)
Tb(1) – C(3) (Cnt)	2.885(12)
Tb(1) – C(4) (Cnt)	2.828(12)
Tb(1) – C(5) (Cnt)	2.722(10)
Tb(1) – Cent (Cnt)	2.01
Tb(1) – Cent (Cnt) –8C	1.97
Plane (Cot) ^Plane (Cnt 8C)	3.94 °
Cent-Cot - Tb(1) - Cent-Cnt	175.74 °
Cent-Cot - Tb(1)- Cent-Cnt 8C	179.97 °
Plane (C(2)/C(1)/C(2’)) ^Plane (Cnt 8C)	48.14 °

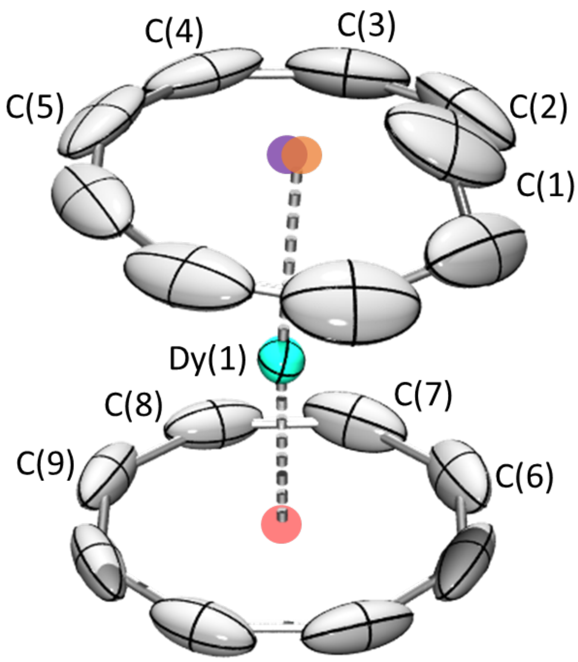

***Figure S43**: Molecular structure of **2**-Dy-trans (100% ratio).*

***Table S22**: Summary of bond length and angles from the ligands to the metal center.*

	Main distances in Å
Dy(1) – C(6) (Cot)	2.534(5)
Dy(1) – C(7) (Cot)	2.544(5)
Dy(1) – C(8) (Cot)	2.543(5)
Dy(1) – C(9) (Cot)	2.544(5)
Dy(1) – Cent (Cot)	1.78
Dy(1) – C(1) (Cnt)	2.660(9)
Dy(1) – C(2) (Cnt)	2.825(9)
Dy(1) – C(3) (Cnt)	2.858(9)
Dy(1) – C(4) (Cnt)	2.806(6)
Dy(1) – C(5) (Cnt)	2.720(6)
Dy(1) – Cent (Cnt)	1.99
Dy(1) – Cent (Cnt) –8C	1.96
Plane (Cot) ^Plane (Cnt 8C)	3.52 °
Cent-Cot - Dy(1) - Cent-Cnt	179.57 °
Cent-Cot - Dy(1)- Cent-Cnt 8C	175.75 °
Plane (C(2)/C(1)/C(2’)) ^Plane (Cnt 8C)	52.63 °

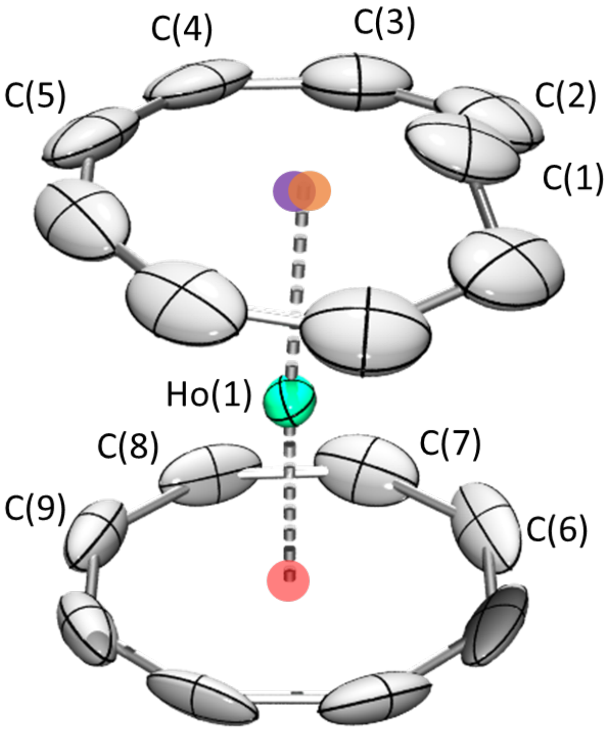

***Figure S44**: Molecular structure of **2**-Ho-trans (100% ratio).*

***Table S23**: Summary of bond length and angles from the ligands to the metal center.*

	Main distances in Å
Ho(1) – C(6) (Cot)	2.518(7)
Ho(1) – C(7) (Cot)	2.529(8)
Ho(1) – C(8) (Cot)	2.524(7)
Ho(1) – C(9) (Cot)	2.534(6)
Ho(1) – Cent (Cot)	1.76
Ho(1) – C(1) (Cnt)	2.673(11)
Ho(1) – C(2) (Cnt)	2.819(9)
Ho(1) – C(3) (Cnt)	2.86(1)
Ho(1) – C(4) (Cnt)	2.805(8)
Ho(1) – C(5) (Cnt)	2.684(7)
Ho(1) – Cent (Cnt)	2.00
Ho(1) – Cent (Cnt) –8C	1.95
Plane (Cot) ^Plane (Cnt 8C)	3.36 °
Cent-Cot - Ho(1) - Cent-Cnt	176.73 °
Cent-Cot - Ho(1)- Cent-Cnt 8C	178.81 °
Plane (C(2)/C(1)/C(2’)) ^Plane (Cnt 8C)	50.85 °

6. UV-visible Spectroscopy

a. KCnt

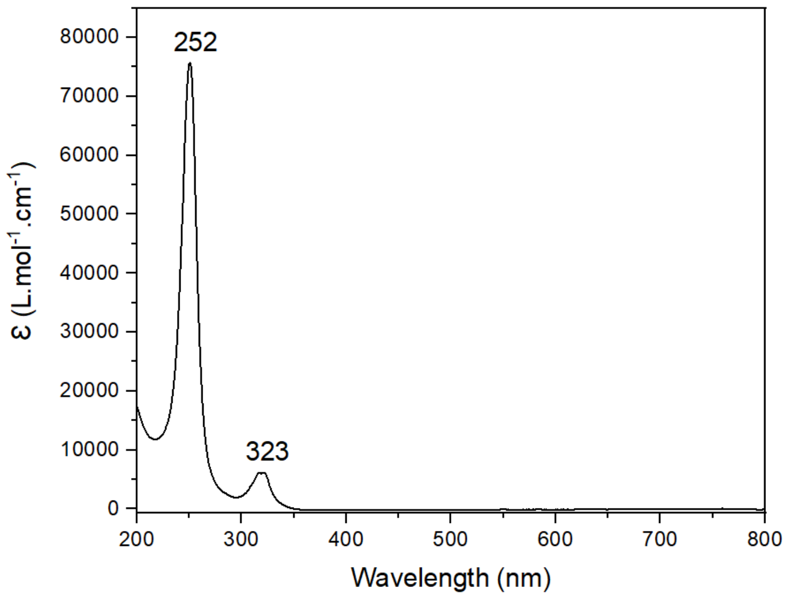

***Figure S45**: UV-Visible spectrum of KCnt-cis in MeCN (3.0*10^{-5} M)*

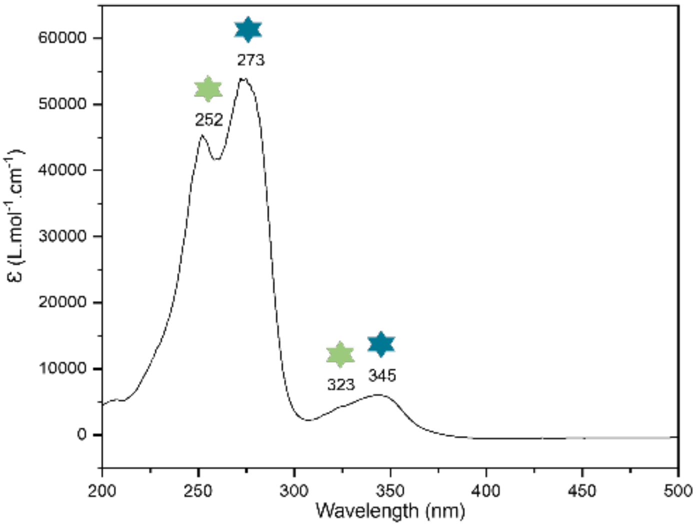

***Figure S46**: UV-Visible spectrum of KCnt-trans in MeCN (3.07*10-4M). KCnt-trans in blue, KCnt-cis in green.*

b. LnCotCnt

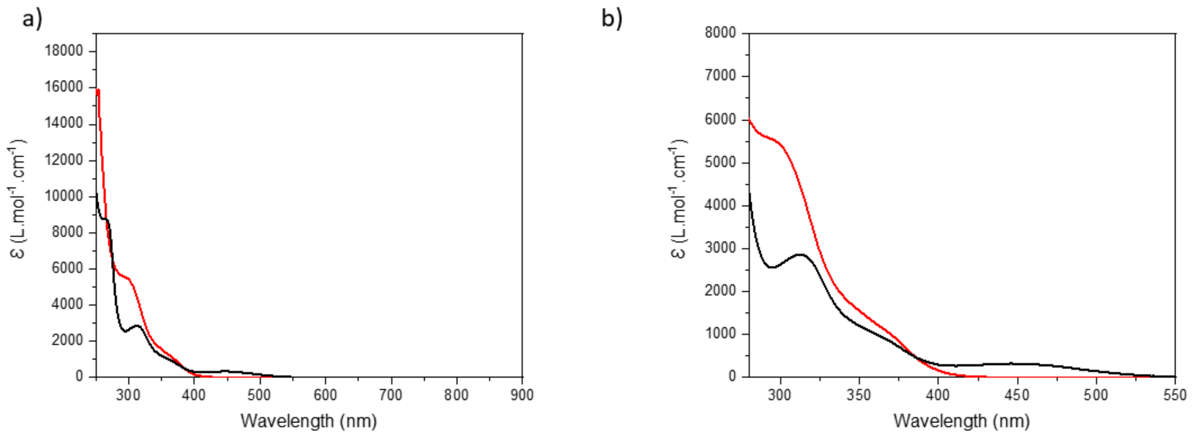

***Figure S47**: UV-Visible spectra of **2**-Y (trans isomer in black 1.16*10^{-3} M cis isomer in red 1.16*10^{-3}M) in DCM. b) zoom in on the irradiation wavelength*

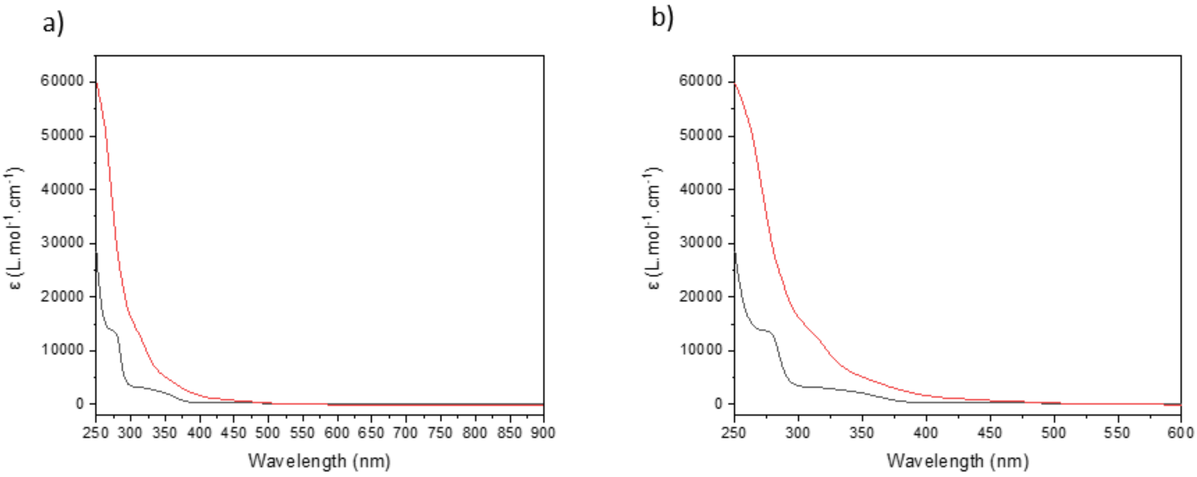

***Figure S48**: a) UV-Visible spectra of **2**-La (cis isomer in red 2.57*10^{-4} M, trans isomer in black 4.74*10^{-4} M) in DCM. b) zoom in on the irradiation wavelength*

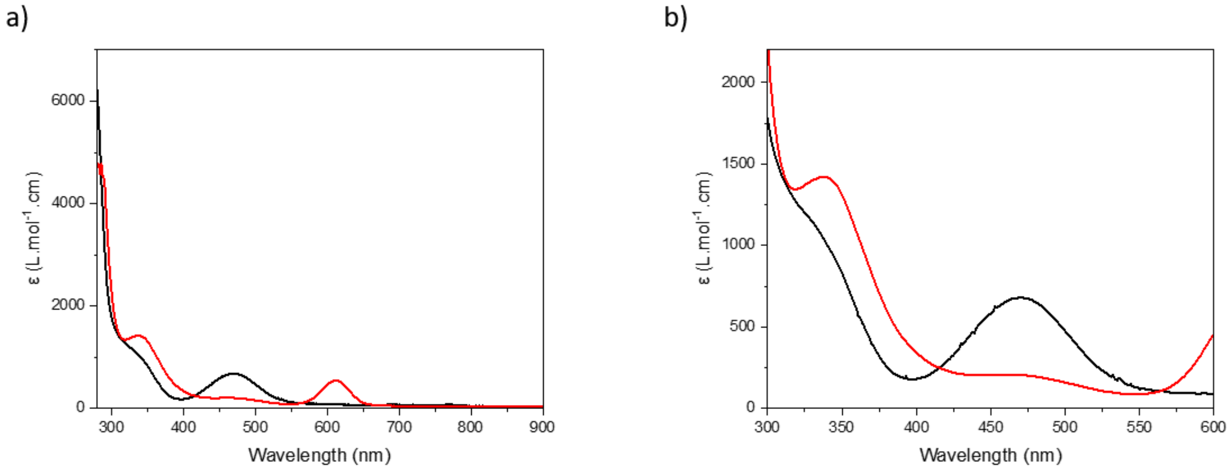

***Figure S49**: UV-Visible spectra of **2**-Ce (cis isomer in red 4.15*10^{-4} M, trans isomer in black 3.32*10^{-4} M) in DCM. b) zoom in on the irradiation wavelength*

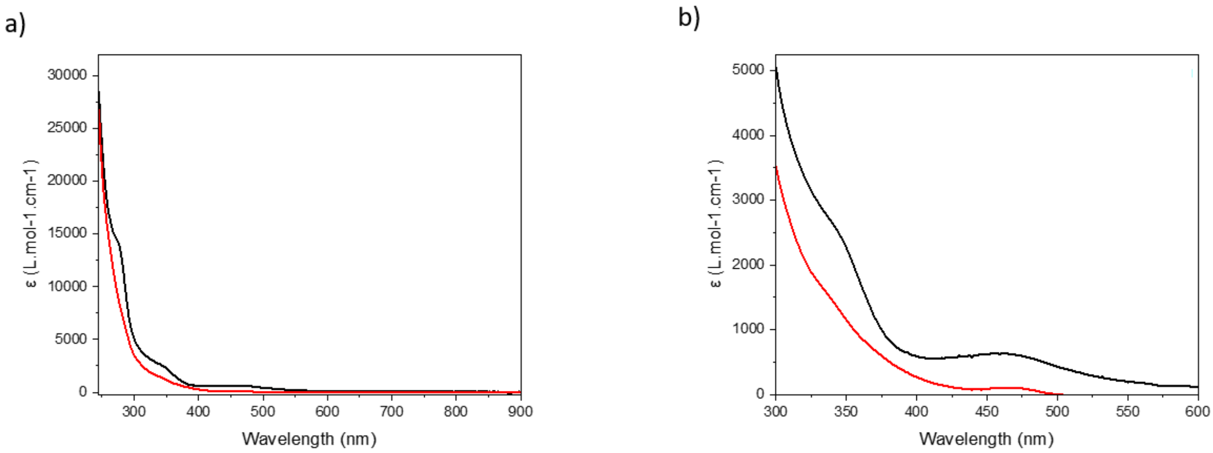

Figure S50: *UV-Visible spectra of* ***2****-Pr (cis isomer in red 4.83*10*$^{-4}$ *M, trans isomer in black 2.76*10*$^{-4}$ *M) in DCM. b) zoom in on the irradiation wavelength*

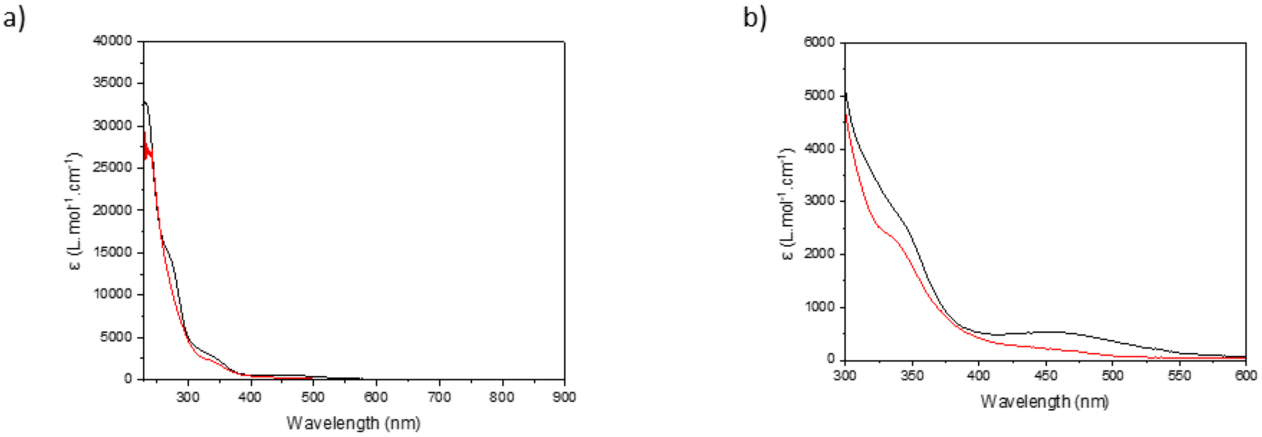

Figure S51: *UV-Visible spectra of* ***2****-Nd (cis isomer in red 6.84*10*$^{-4}$ *M, trans isomer in black 3.56*10*$^{-4}$ *M) in DCM. b) zoom in on the irradiation wavelength*

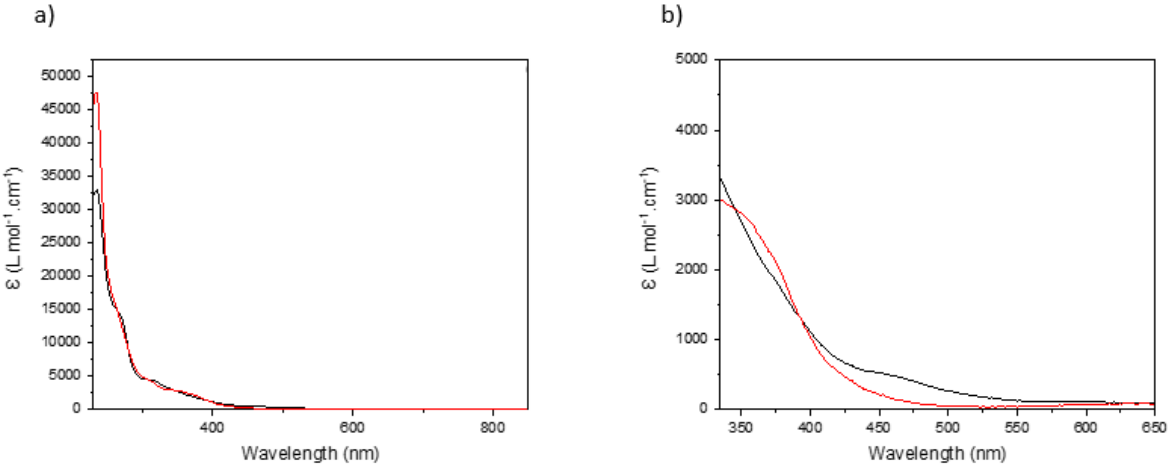

Figure S52: *a) UV-Visible spectra of* ***2****-Sm (cis isomer in red 2.7*10*$^{-4}$ *M, trans isomer in black 3.5*10*$^{-4}$ *M) in DCM. b) zoom in on the irradiation wavelength*

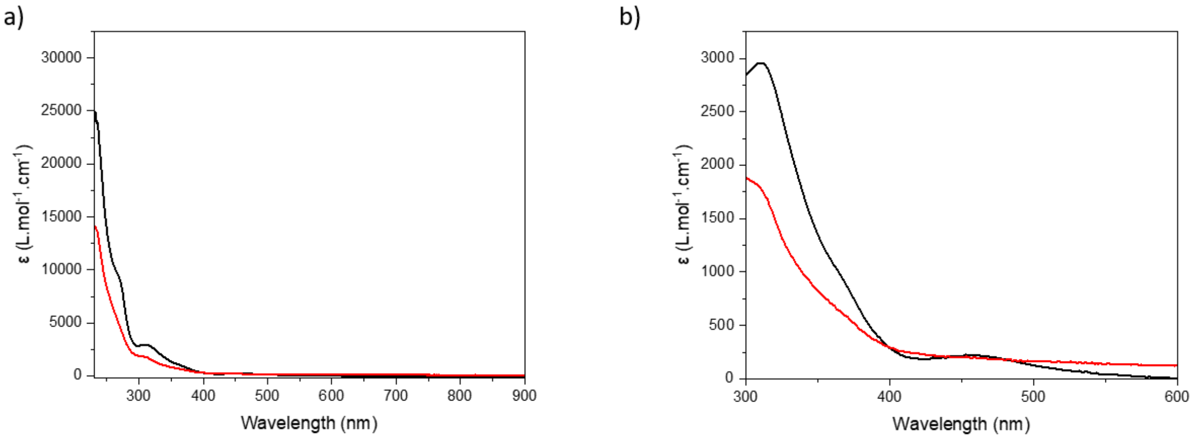

***Figure S53**: UV-Visible spectrum of **2**-Gd (cis isomer in red 7.04*10-4 M, trans isomer in black 6.34*10-4 M) in DCM. b) zoom in on the irradiation wavelength*

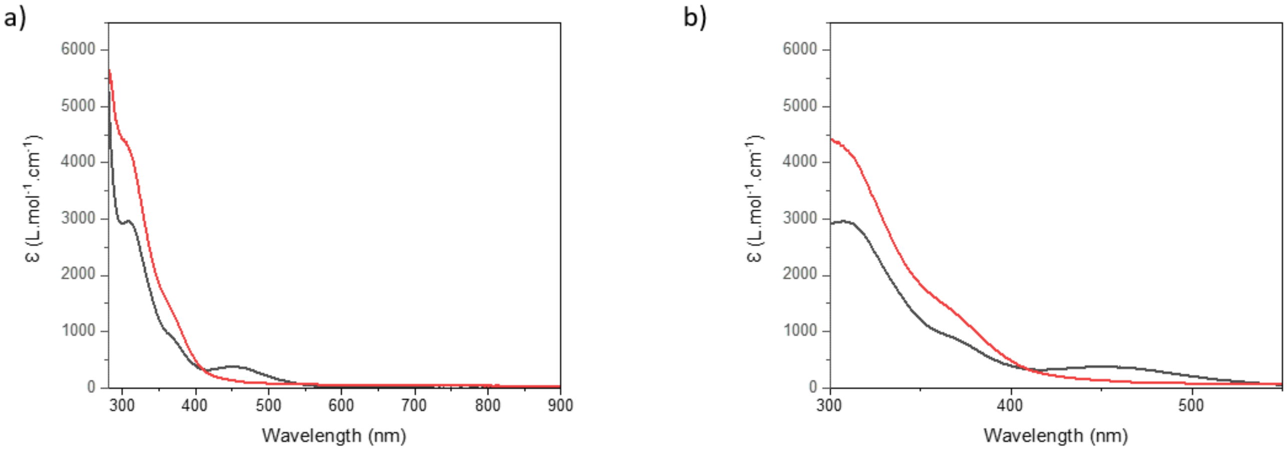

***Figure S54**: UV-Visible spectrum of **2**-Tb (cis isomer in red 3.42*10-3 M, - trans isomer in black 1.05*10-3 M) in DCM. b) zoom in on the irradiation wavelength*

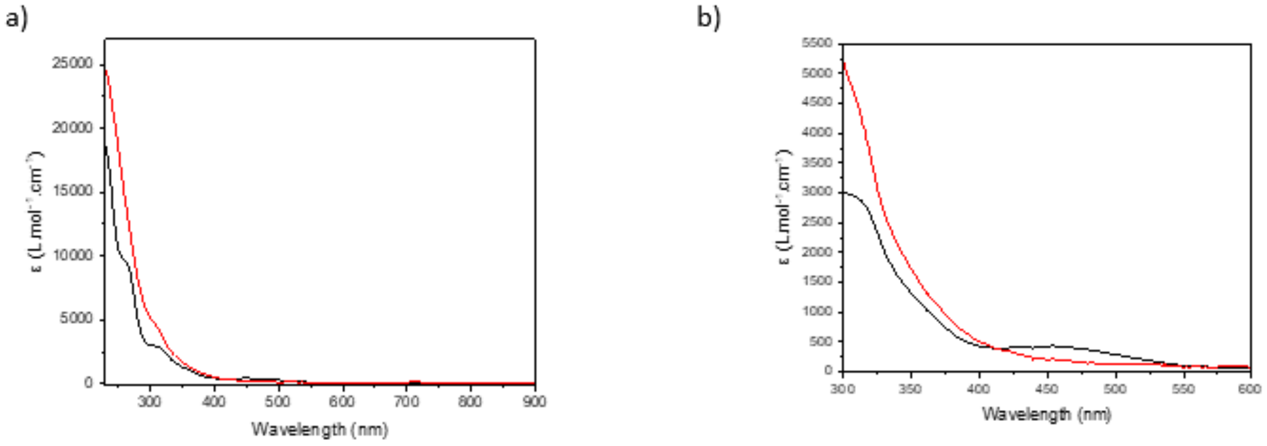

***Figure S55:** UV-Visible spectrum of **2**-Dy (cis isomer in red 1.69*10-4 M, trans isomer in black 2.60*10-4 M) in DCM. b) zoom in on the irradiation wavelength*

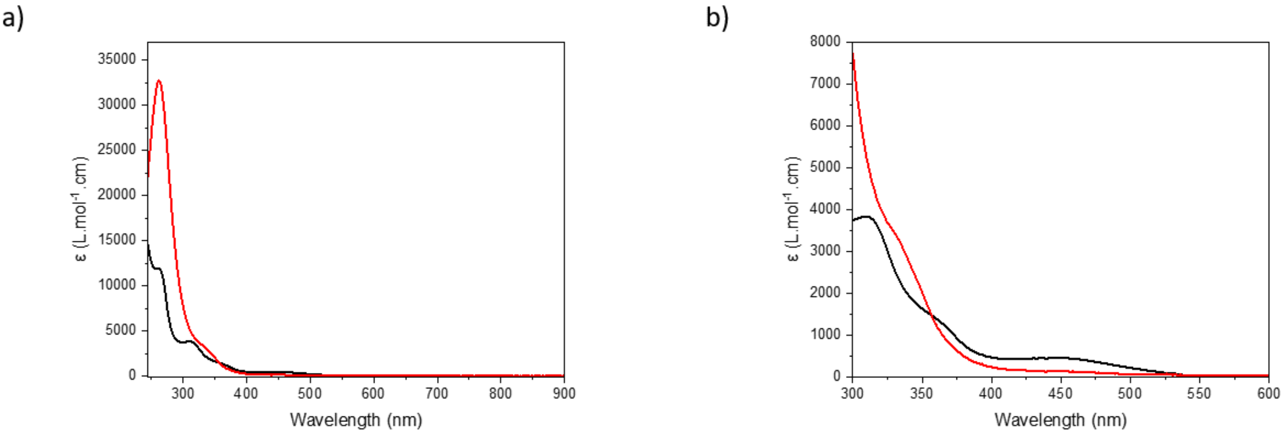

***Figure S56**: UV-Visible spectrum of **2**-Ho (cis isomer in red 2.80*10-4 M, trans isomer in black 3.37*10-4 M) in DCM. b) zoom in on the irradiation wavelength*

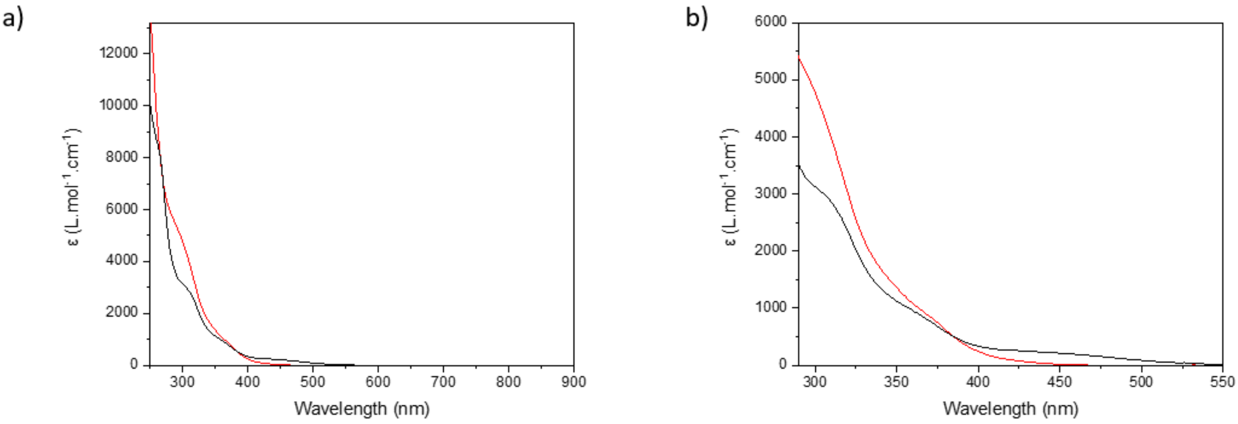

***Figure S57**: a) UV-Visible spectrum of **2**-Er (cis isomer in red 9.43*10-4 M, trans isomer in black 9.43*10-4 M) in DCM. b) zoom in on the irradiation wavelength. The trans isomer was generated through photoirradiation at 370nm for 10 min.*

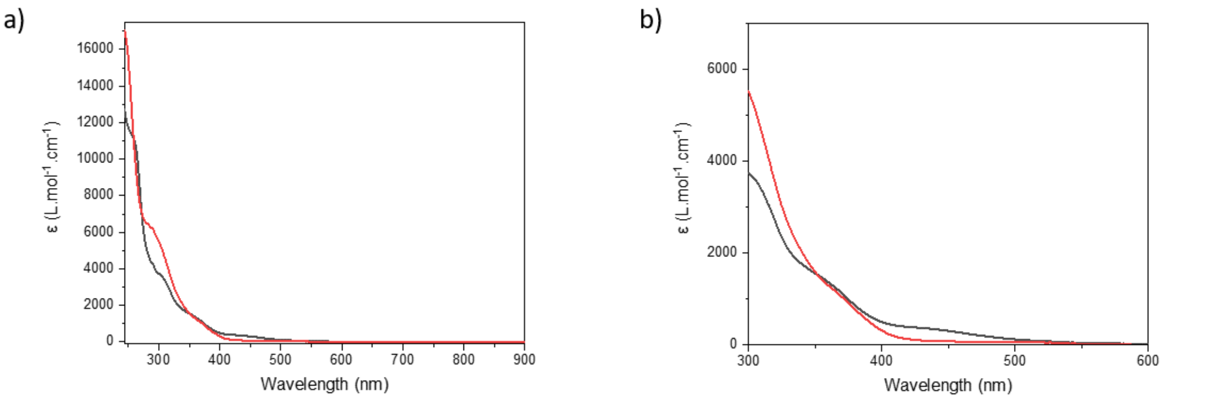

***Figure S58**: a) UV-Visible spectrum of **2**-Tm (cis isomer in red 7.68*10-4 M, trans isomer in black 7.68*10-4 M) in DCM. b) zoom in on the irradiation wavelength. The trans isomer was generated through photoirradiation at 370nm for 10 min.*

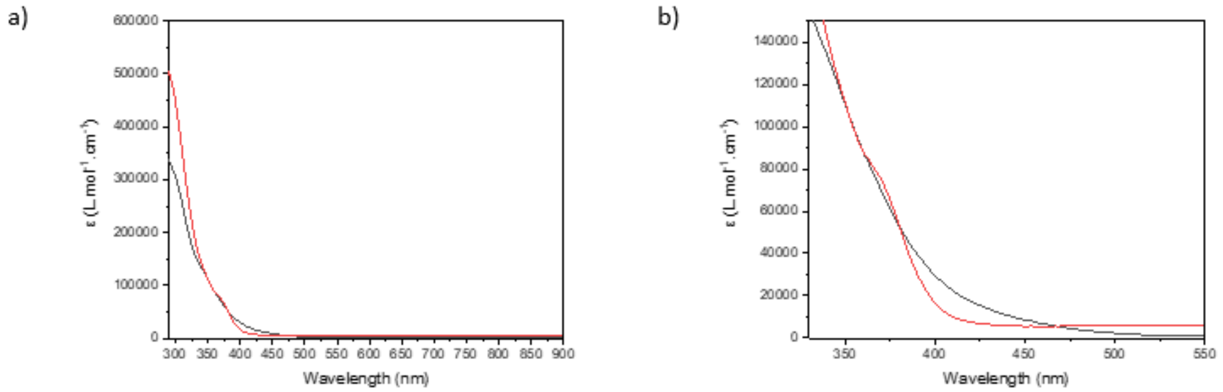

Figure S59**: a) UV-Visible spectrum of* ***2*-Lu (cis isomer in red 3.28*10^{-3} M, trans isomer in black 3.28*10^{-3} M) in DCM. b) zoom in on the irradiation wavelength The trans isomer was generated through photoirradiation at 370nm for 10 min.*

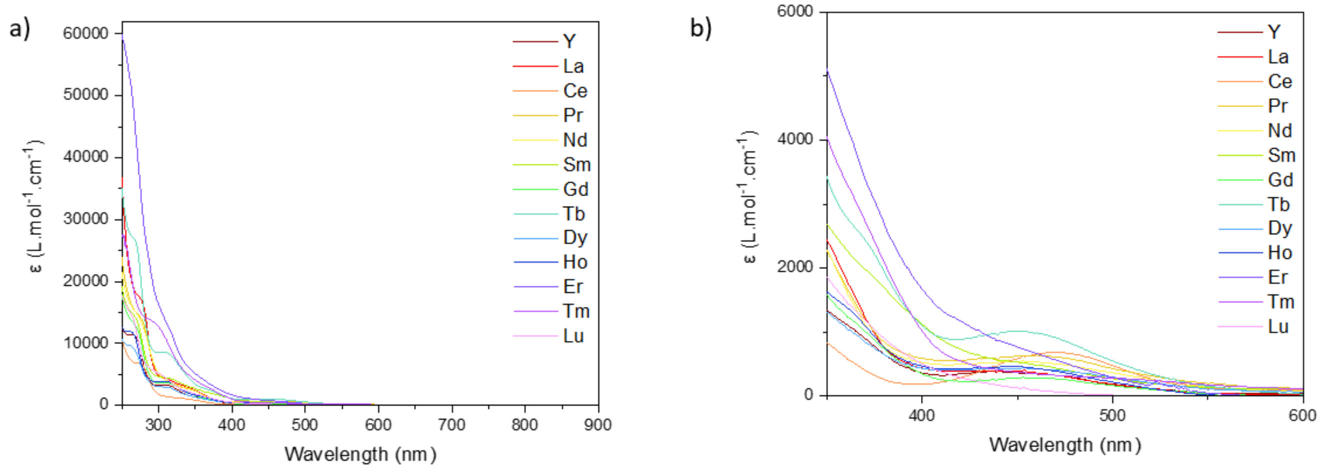

Figure S60**: a) UV-Visible spectra of all* ***2*-Ln-trans in DCM. b) zoom in on the irradiation wavelength*

7. Isomerization study

General details. Studies were conducted in an NMR tube in toluene-*d$_8$* and irradiated in a black box with an adapted NMR tube holder. All tubes were protected from the light in aluminum foil during the convoy from the irradiation box to the NMR spectrometer.
For the Photo stationary state study, all samples were irradiated 30 min at each λ (2 X 15min) to ensure that the PSS was reached. The irradiation range was performed on Kessil's lamp (370 nm, 390 nm, 427 nm, 440 nm, 450 nm, 467 nm, and 525 nm). The irradiation started from 370 nm to 525 nm, and in order to test the reversibility of the isomerization, the samples were irradiated stepwise from 525 nm to 370 nm. For **2**-Ce, an additional lamp was used from Prizmatix FC5-LED Multi channel light source (655 nm). The irradiation took place inside the NMR with an adapted Young NMR tube to put an optic fiber inside. The irradiation lasted a total of 2h 50 min (1h 40 min + 50 min to reach the PSS)
It must be noted that all UV-Vis spectra of the **2**-Ln-*trans* display a mixture of the *cis* and *trans* isomer.

a) UV and NMR study

i. KCnt Isomerization

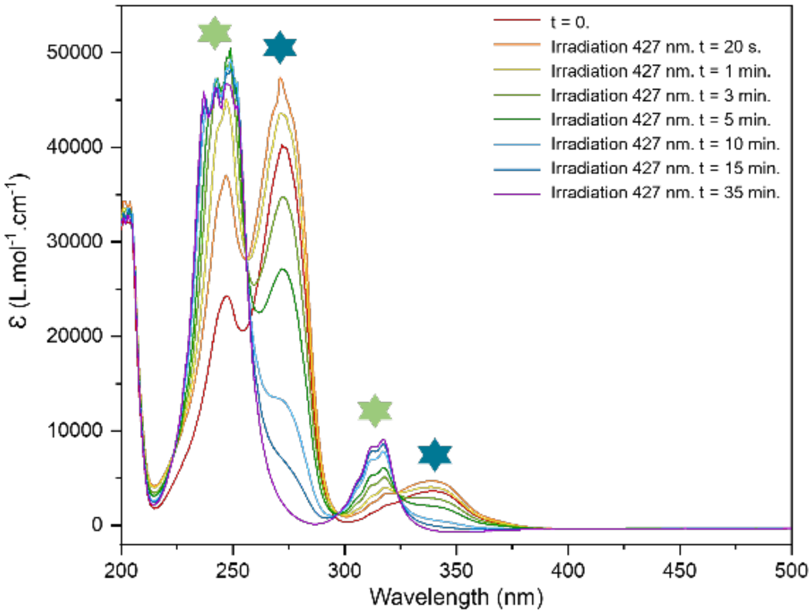

Figure S61: *Isomerization study by UV-visible spectroscopy of KCnt in THF. Green star: absorption band attributed to KCnt-cis; Blue star: absorption band attributed to KCnt-trans*

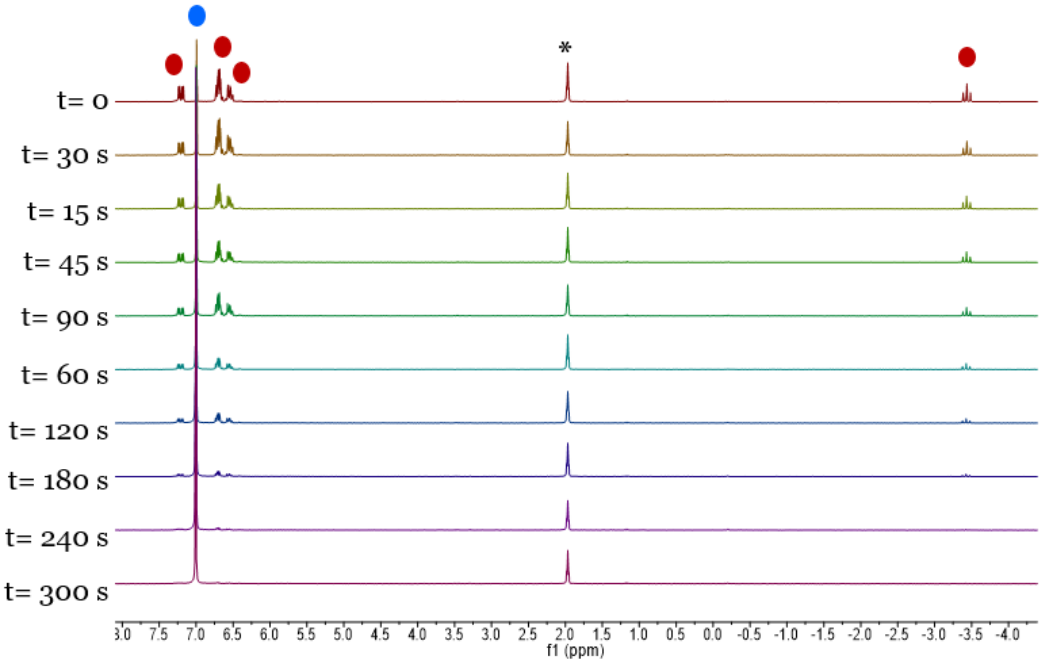

Figure S62: *Isomerization study by ^{1}H NMR of KCnt in CD_3CN at 427 nm. Red dots: signals attributed to the trans isomer; blue dot: signal attributed to the cis isomer*

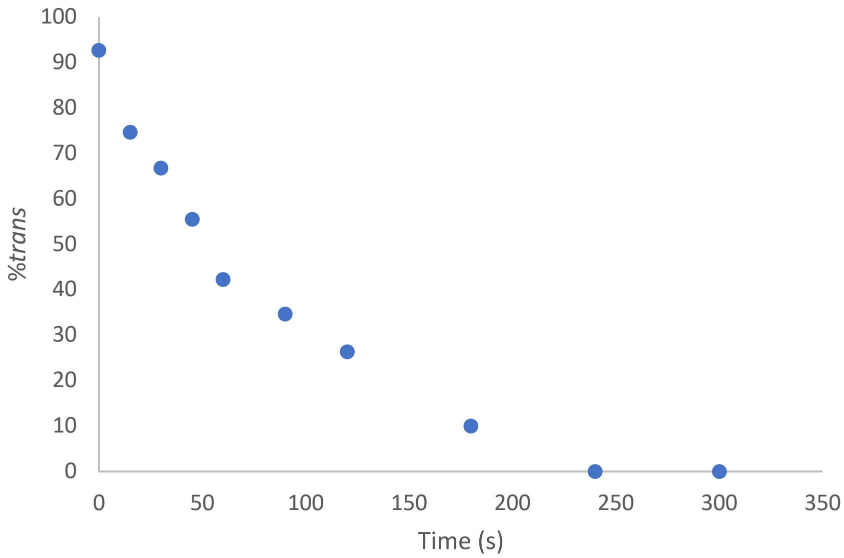

Figure S63: *Plotted data of KCnt isomerization*

ii. $Sm(Cnt)_2$

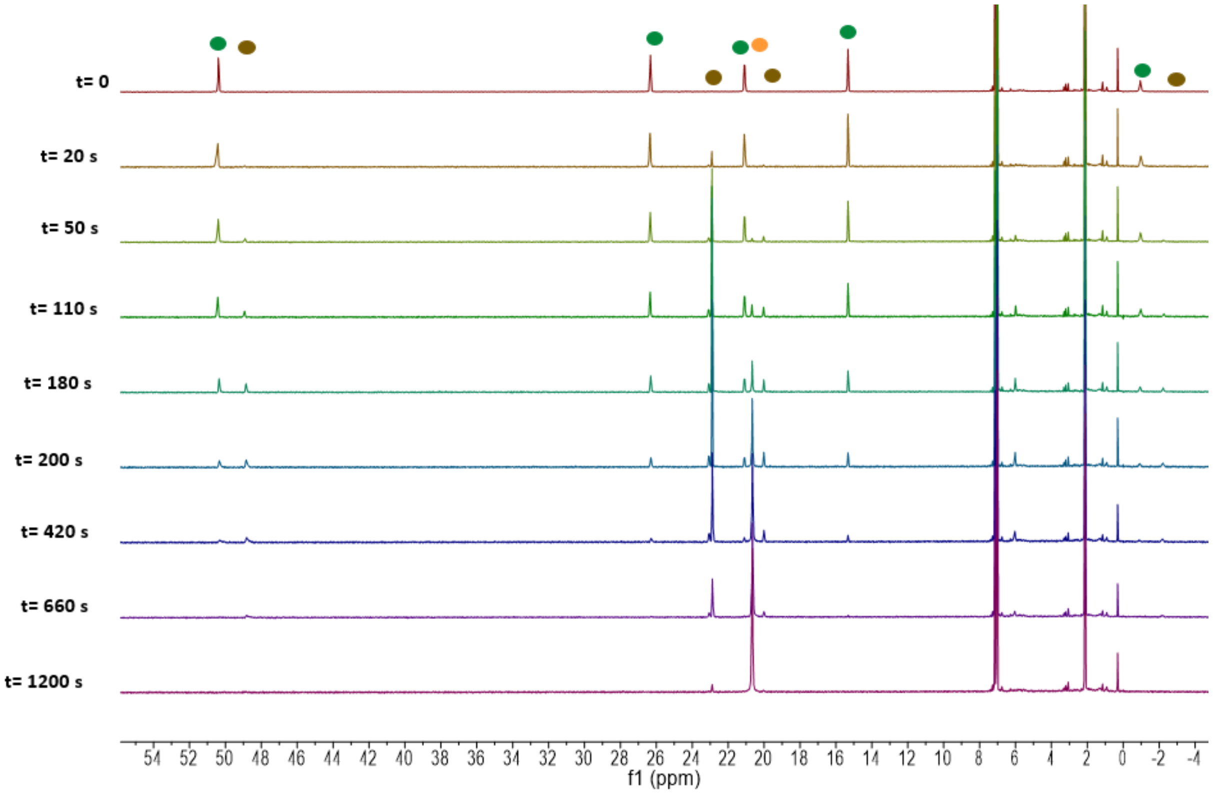

Figure S64: *Isomerization study by* ^{1}*H NMR of* ***1*** *in toluene at 427 nm. Green dots: signals attributed to the trans, trans isomer; brown dots: signals attributed to the cis, trans isomer; yellow dot: signals attributed to the cis, cis isomer.*

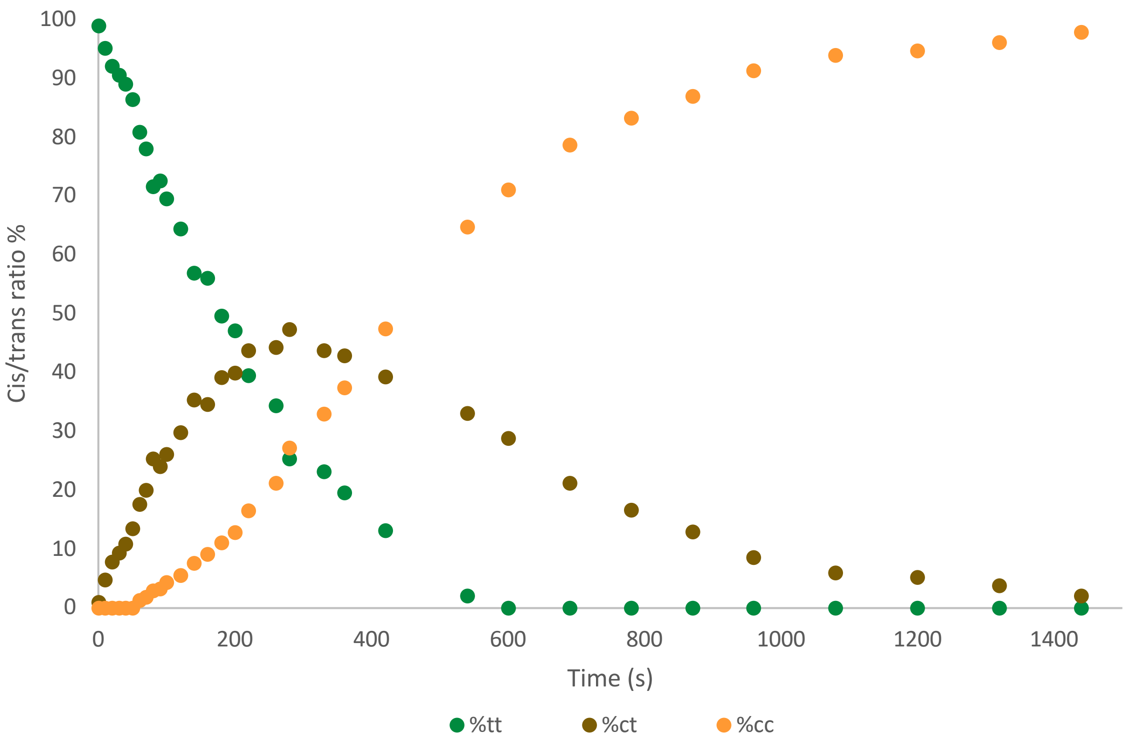

Figure S65: *Plotted data of **1** in toluene at 427 nm.*

iii. SmCotCnt

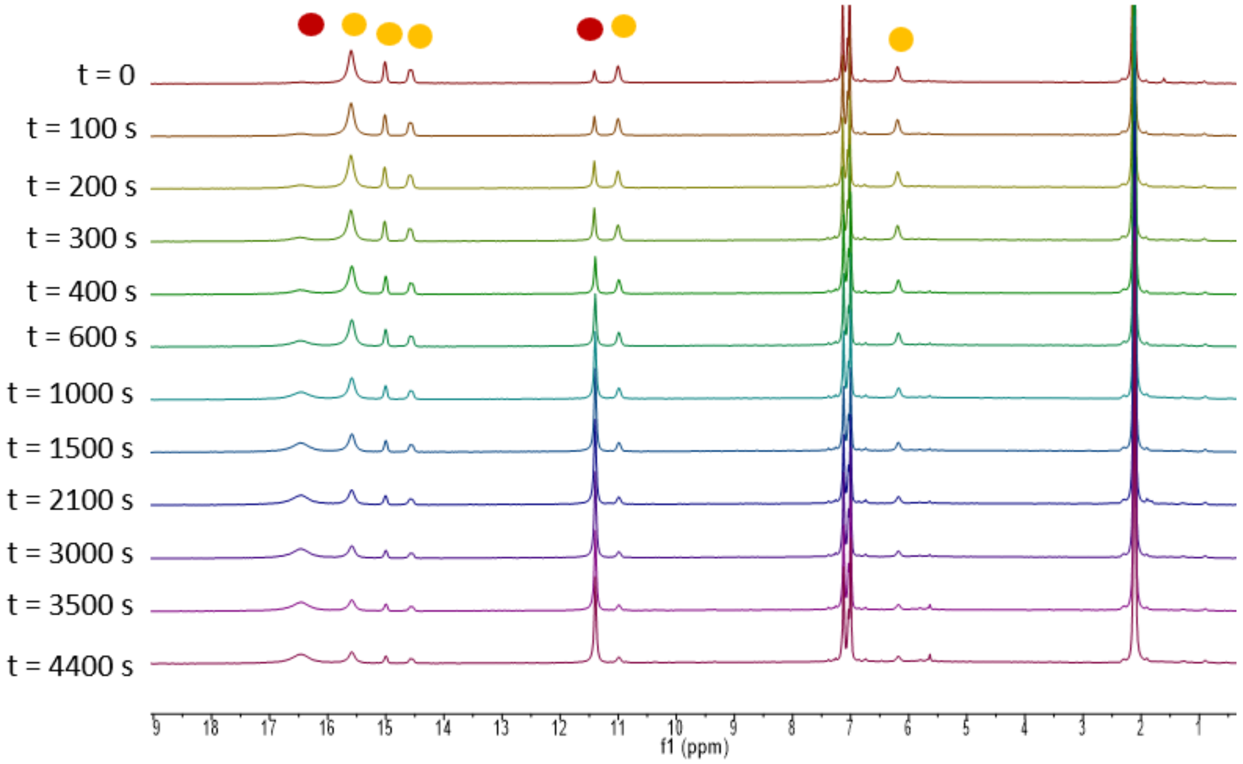

Figure S66: *Isomerization study by ^{1}H NMR of **2**-Sm in toluene at 427 nm. Red dots: signals attributed to the trans isomer; yellow dots: signals attributed to the cis isomer.*

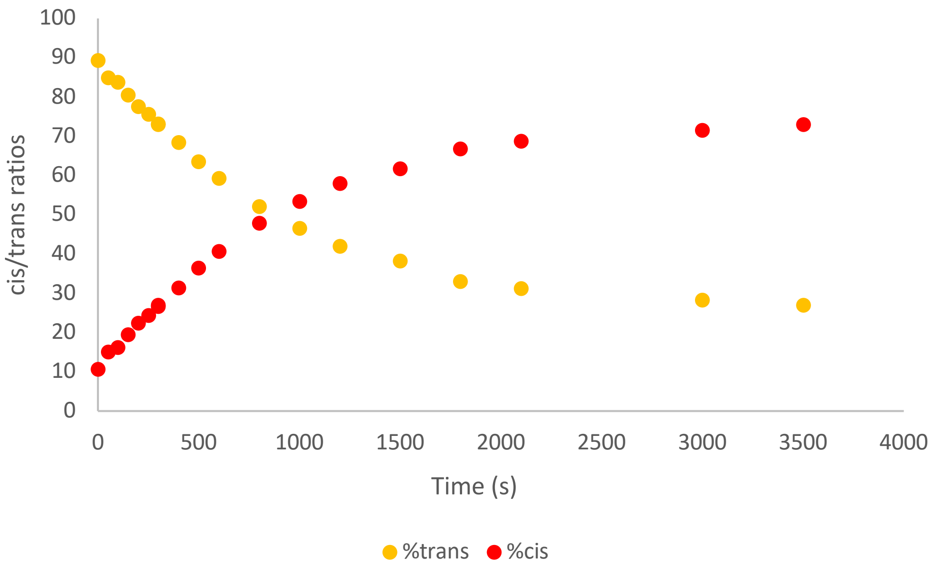

Figure S67: *Plotted data of **2**-Sm in toluene at 427 nm*

b) Photostationary (PSS) study

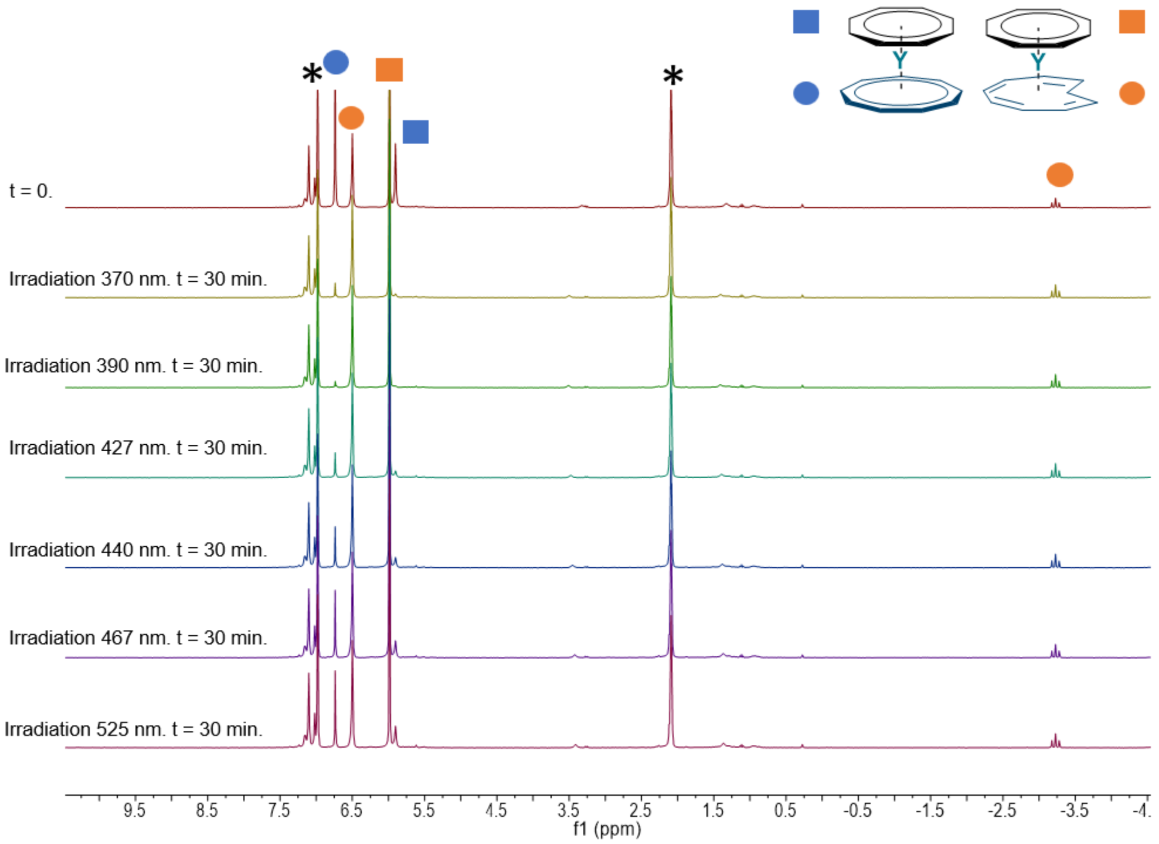

Figure S68: *Isomerization study by ^{1}H NMR of **2**-Y in toluene-d_8 measured at 293 K at different wavelength (* residual protio signal of the solvent)*

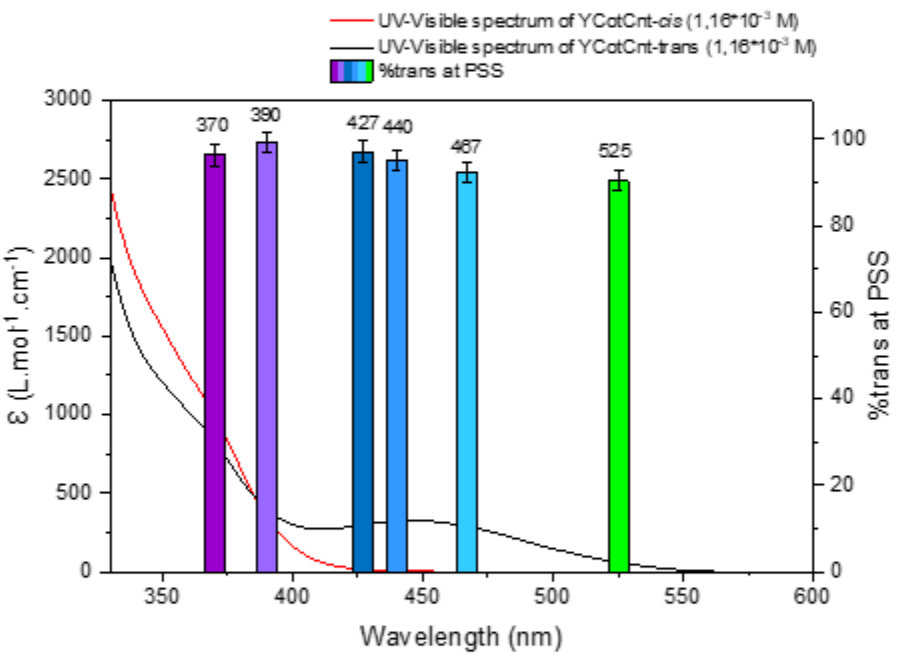

***Figure S69**: Evolution of the trans/cis ratios of **2**-Y under different wavelengths superimposed with UV spectra of trans and cis isomer*

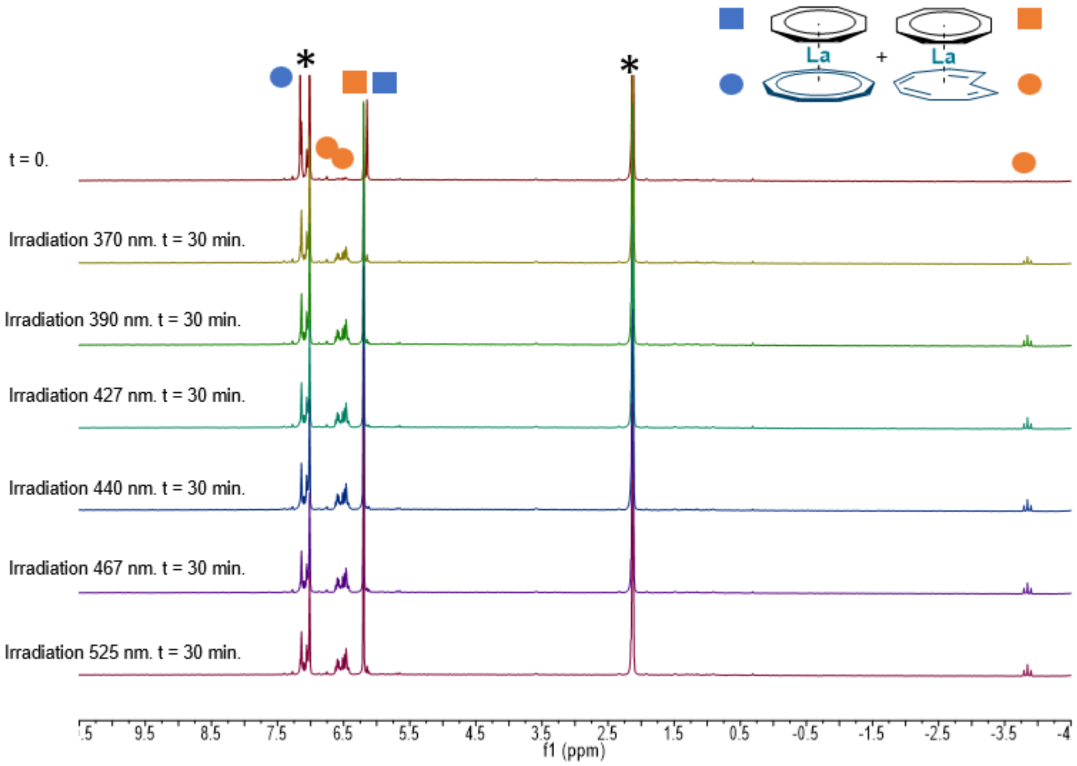

***Figure S70**: Isomerization study by ^{1}H NMR of **2**-La in toluene-d_8 measured at 293 K at different wavelengths (* residual protio signal of the solvent)*

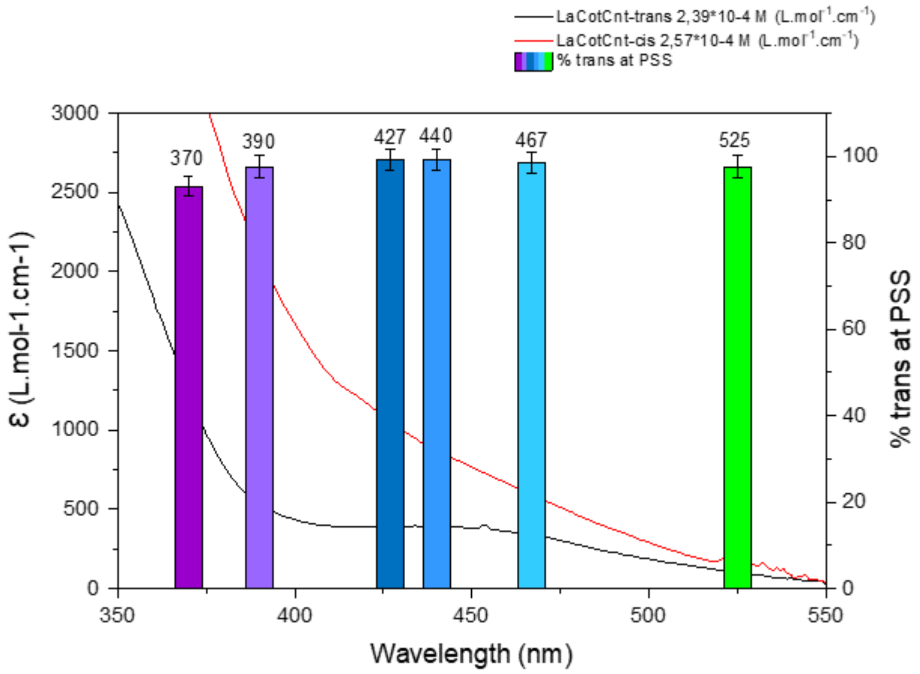

Figure S71: *Evolution of the trans/cis ratios of* ***2****-La under different wavelengths superimposed with UV spectra of trans and cis isomer*

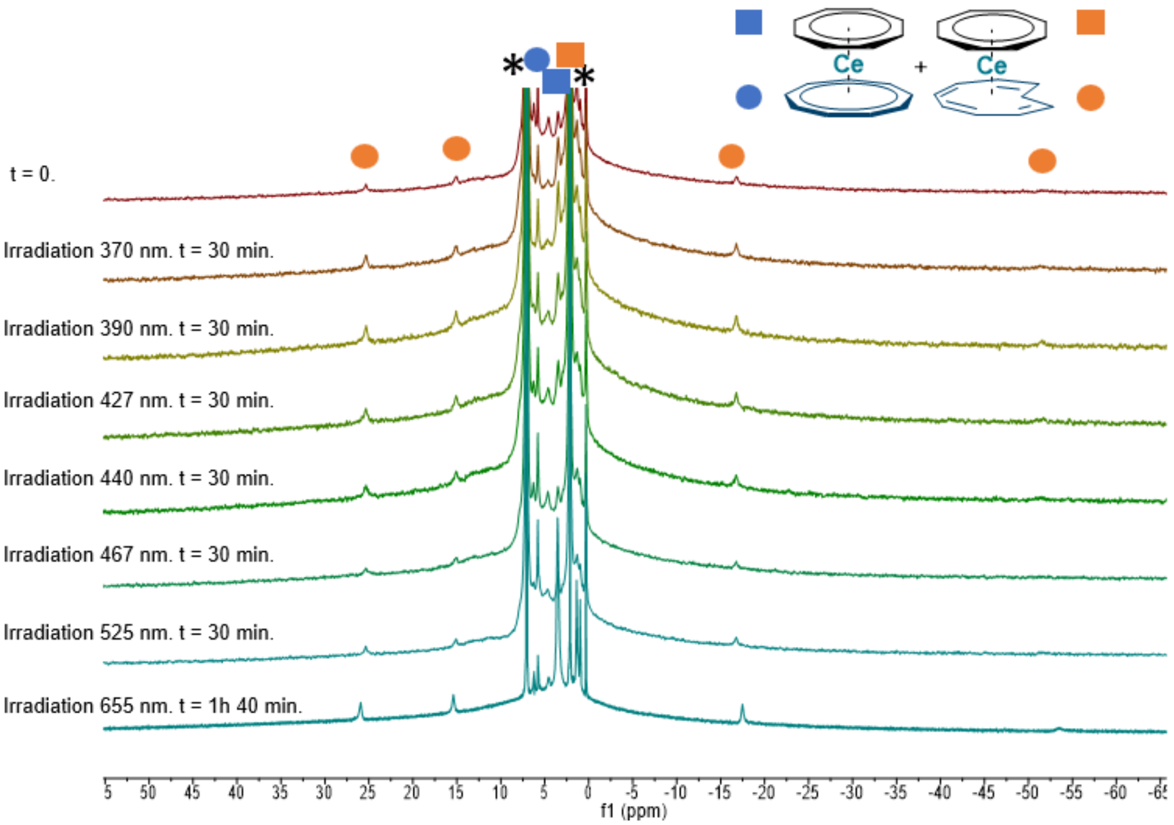

Figure S72 *: Isomerization study by* ^{1}H *NMR of* ***2****-Ce in toluene-*d_8 *measured at 293 K at different wavelengths (* residual protio signal of the solvent)*

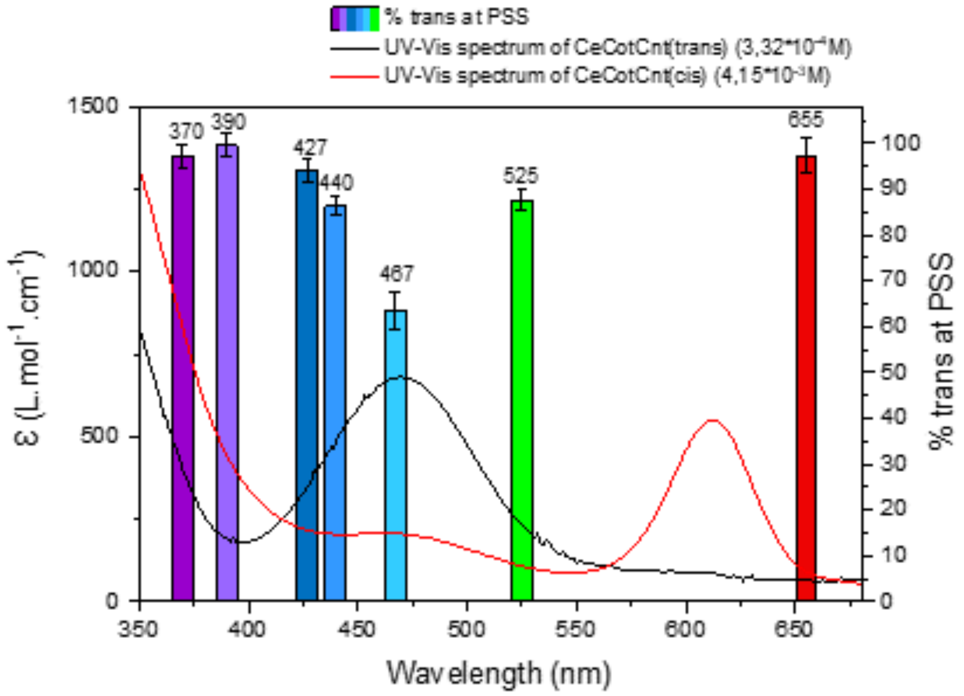

***Figure S73**: Evolution of the trans/cis ratios of **2**-Ce under different wavelengths superimposed with UV spectra of trans isomer*

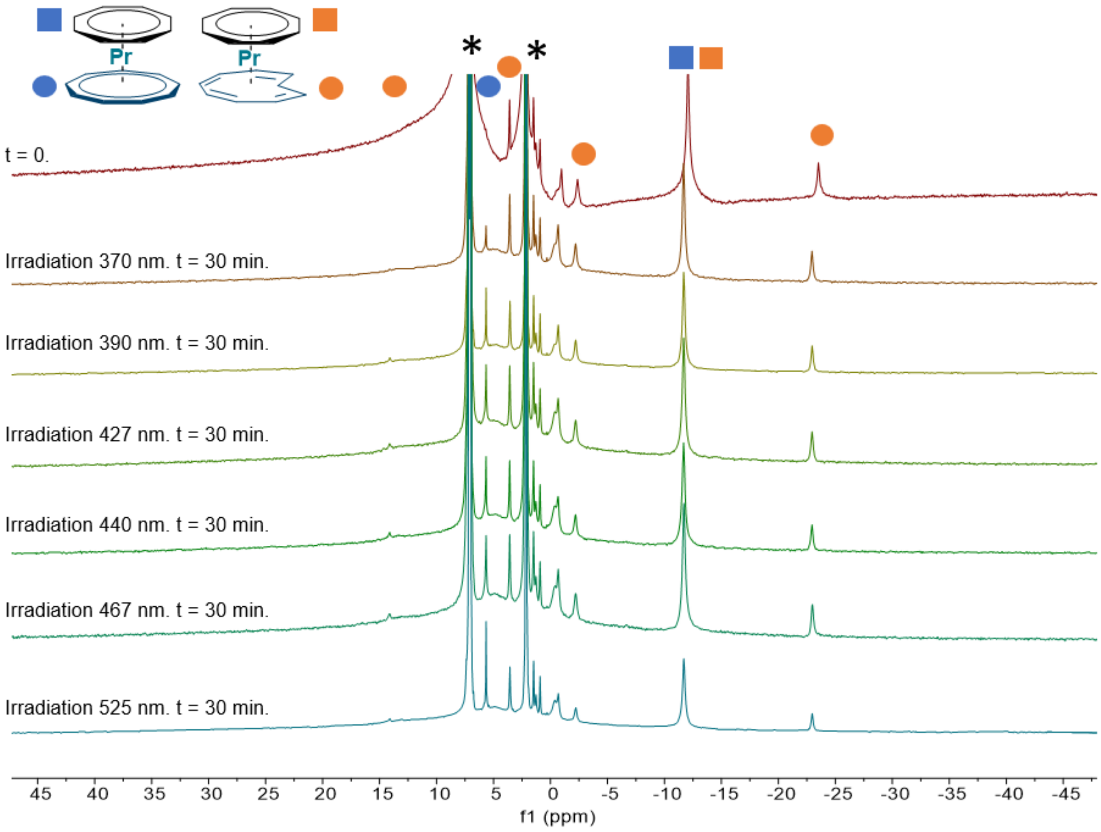

***Figure S74**: Isomerization study by ^{1}H NMR of **2**-Pr in toluene-d_8 measured at 293 K at different wavelengths (* residual protio signal of the solvent)*

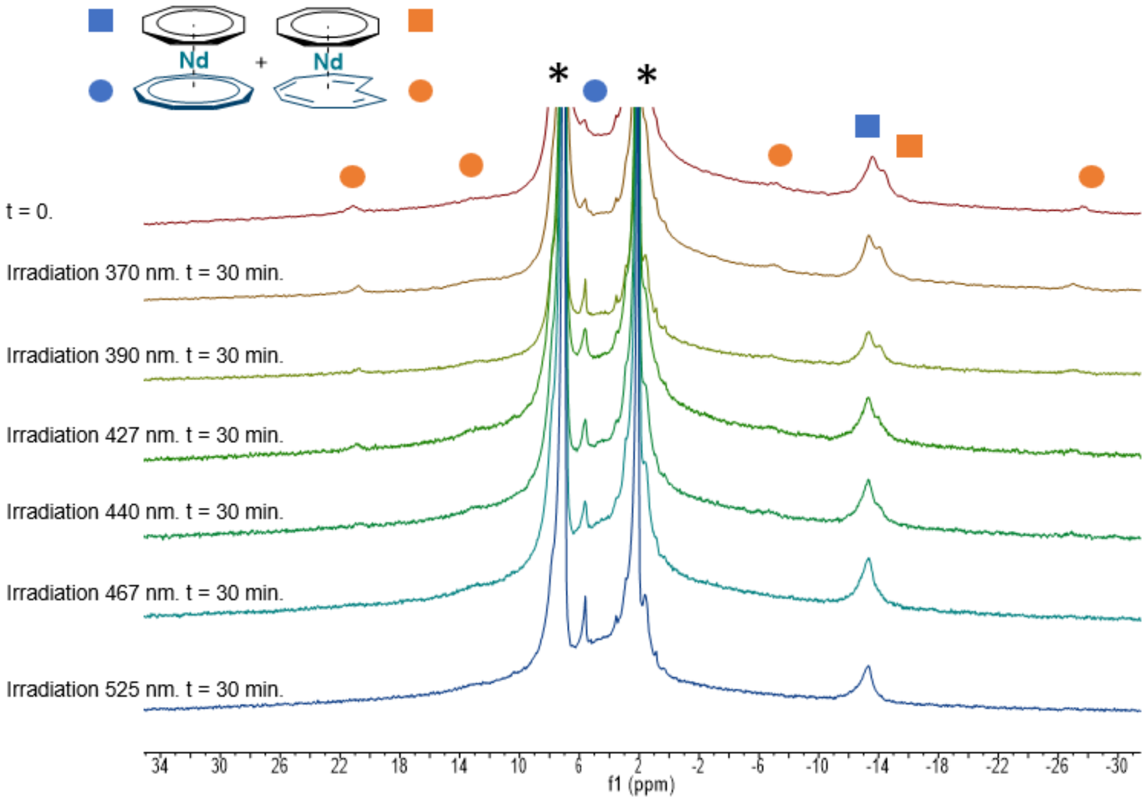

Figure S75: *Isomerization study by* ^{1}H *NMR of* ***2****-Nd in toluene-*d_8 *measured at 293 K at different wavelengths (* residual protio signal of the solvent)*

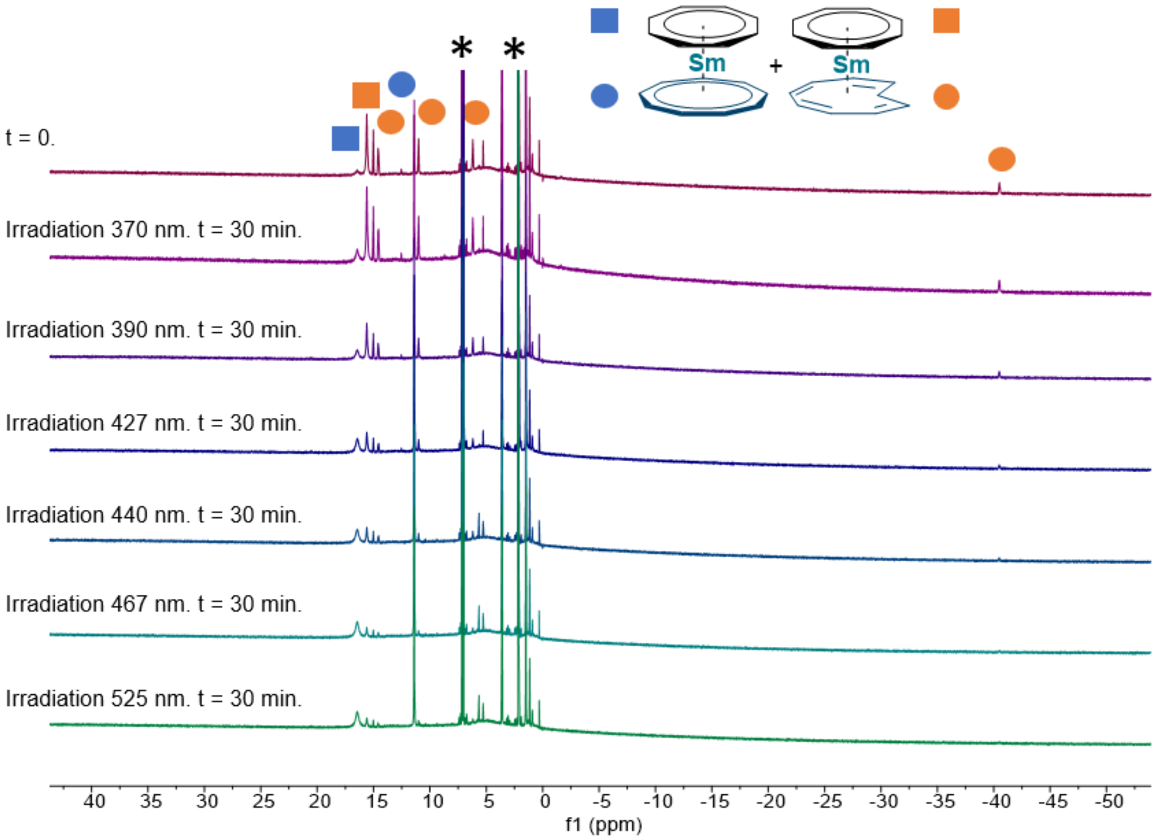

Figure S76: *Isomerization study by* ^{1}H *NMR of* ***2****-Sm in toluene-*d_8 *measured at 293 K at different wavelengths (* residual protio signal of the solvent)*

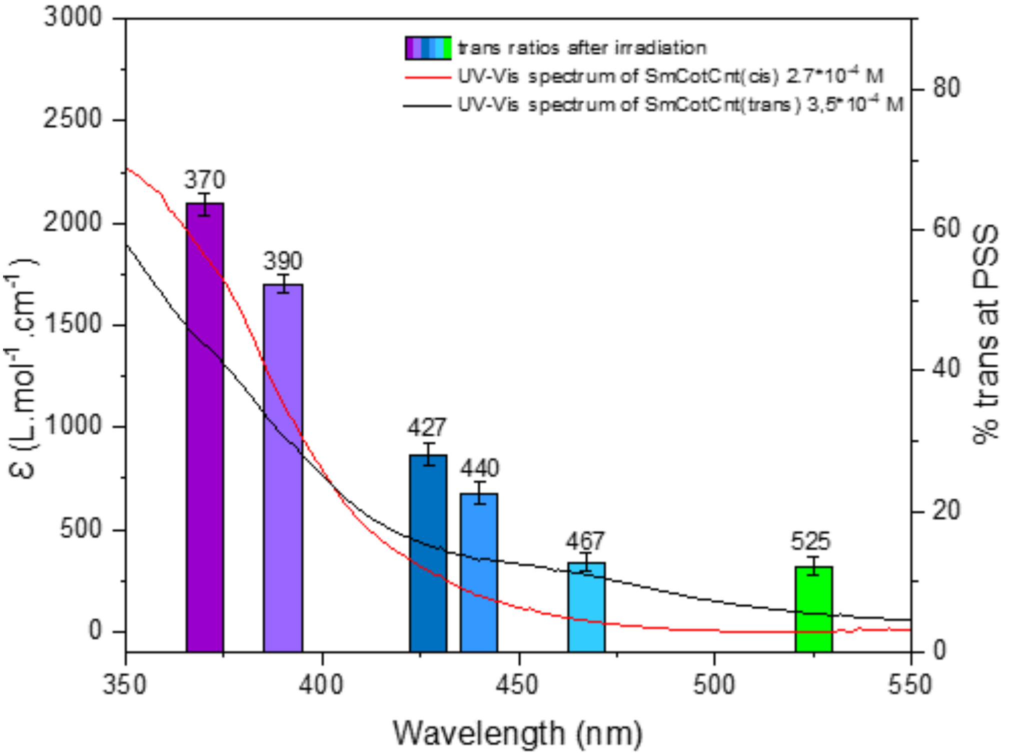

Figure S77: *Evolution of the trans/cis ratios of* ***2****-Sm under different wavelengths superimposed with UV spectra of the cis and trans isomers*

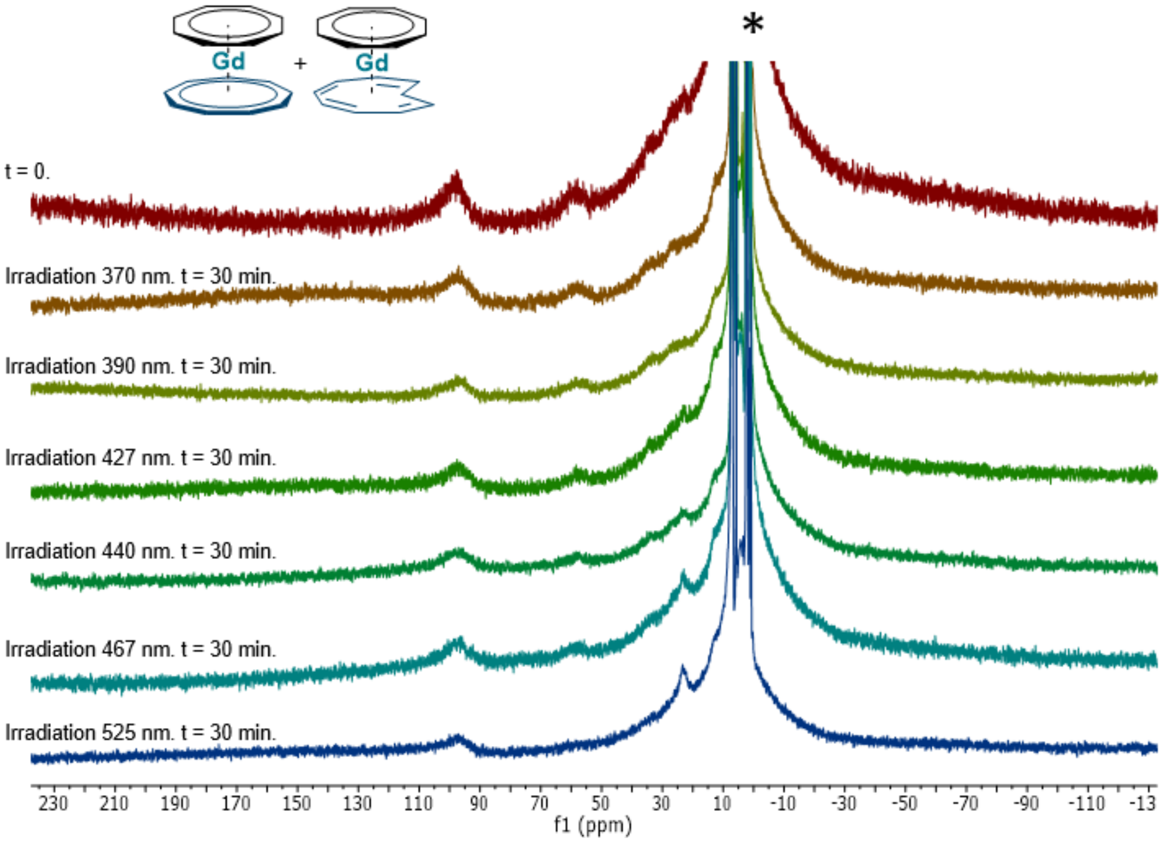

Figure S78: *Isomerization study by* ^{1}H *NMR of* ***2****-Gd in toluene-*d_8 *measured at 293 K at different wavelengths (* residual protio signal of the solvent)*

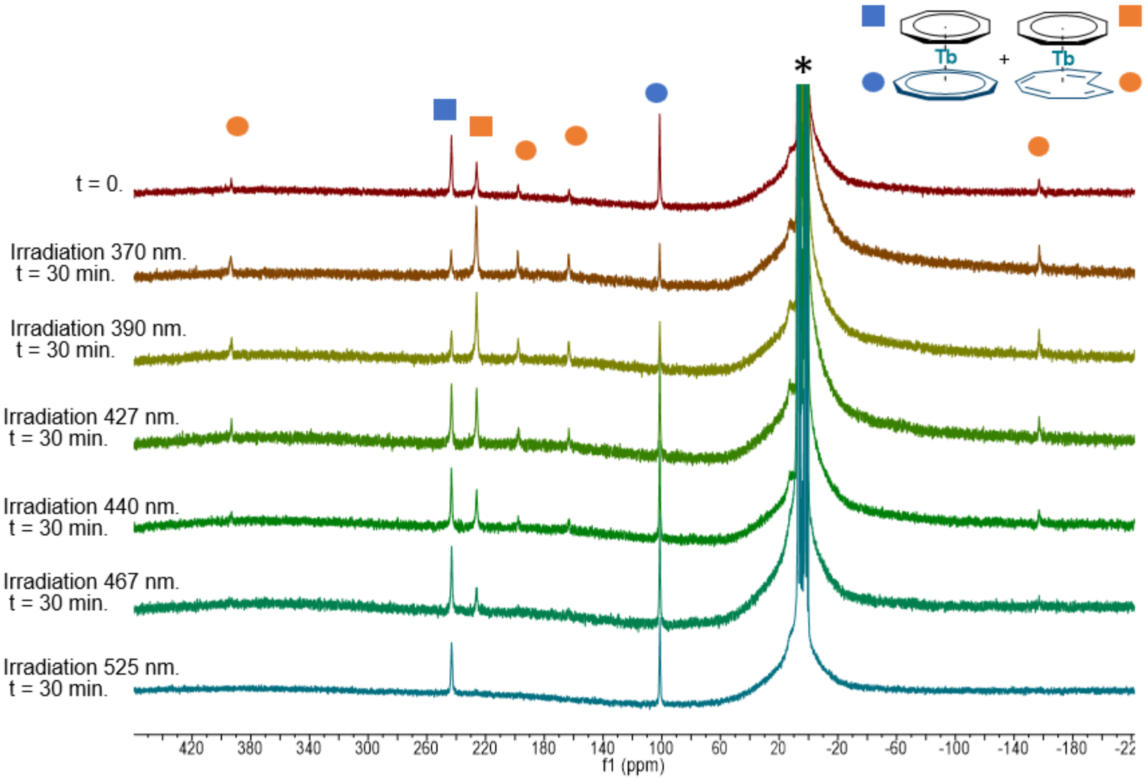

Figure S79: *Isomerization study by ¹H NMR of **2**-Tb in toluene-d_8 measured at 293 K at different wavelengths (* residual protio signal of the solvent)*

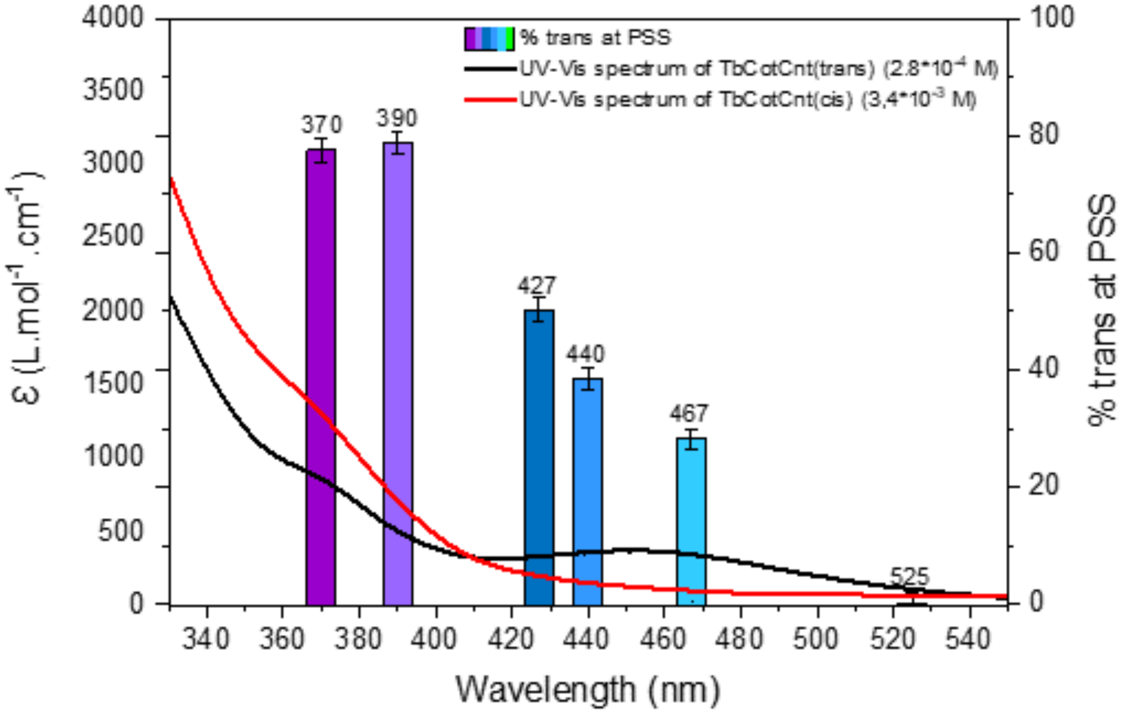

Figure S80: *Evolution of the trans/cis ratios of **2**-Tb under different wavelength under different wavelengths superimposed with UV spectra of the trans isomers*

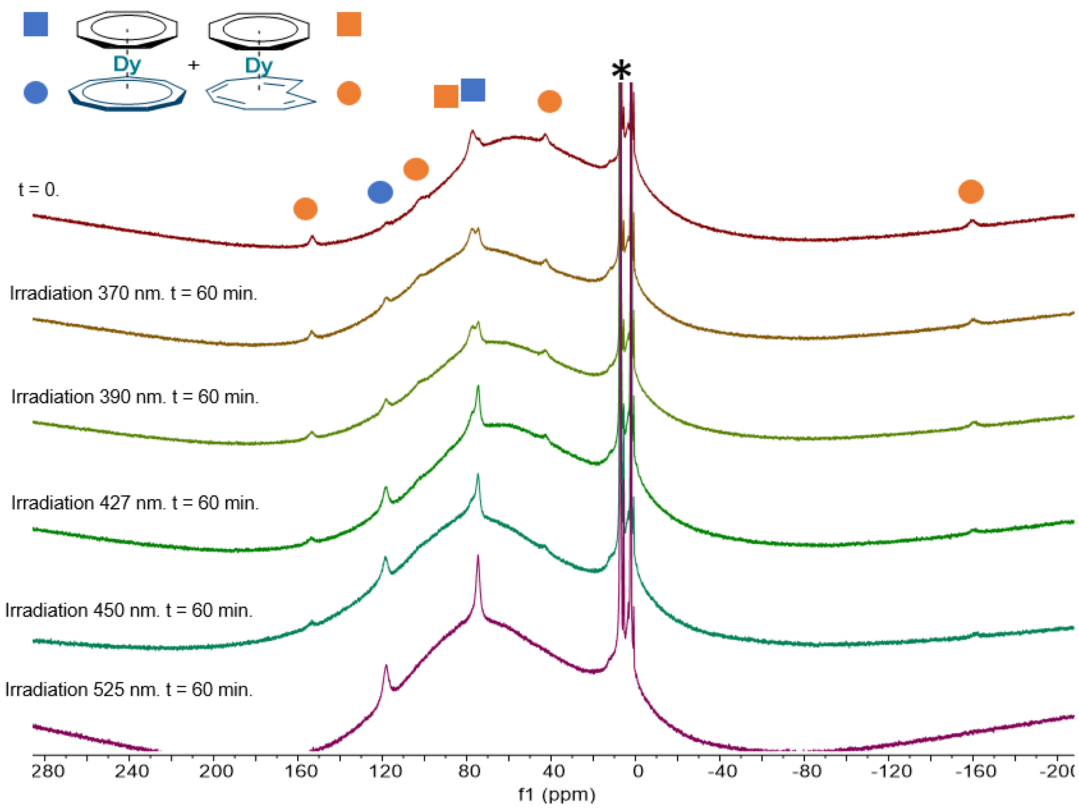

***Figure S81**: Isomerization study by ¹H NMR of **2**-Dy in toluene-d_8 measured at 293 K at different wavelengths (* residual protio signal of the solvent)*

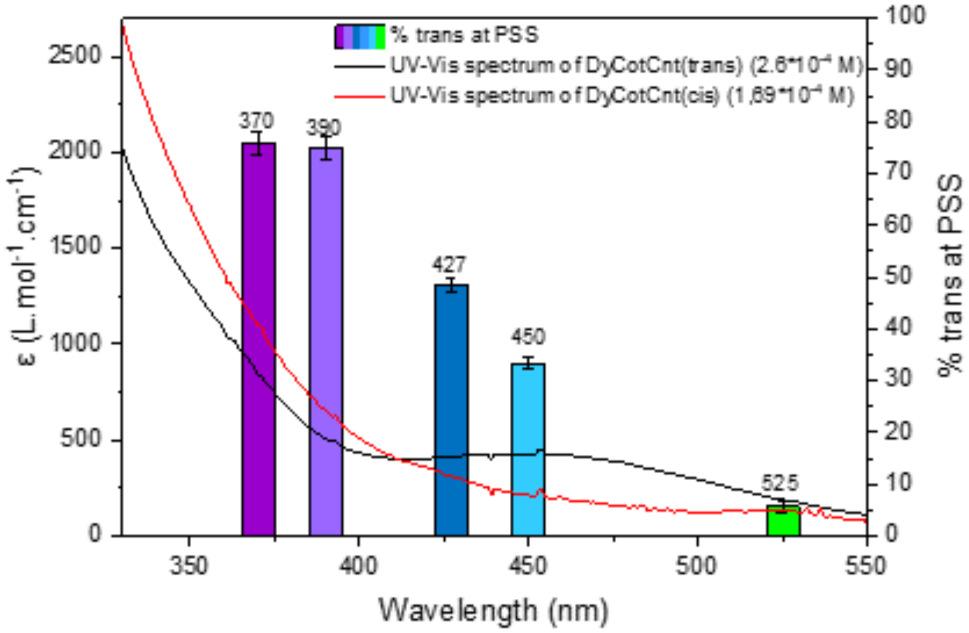

***Figure S82**: Evolution of the trans/cis ratios of **2**-Dy under different wavelengths superimposed with UV spectra of the trans isomers*

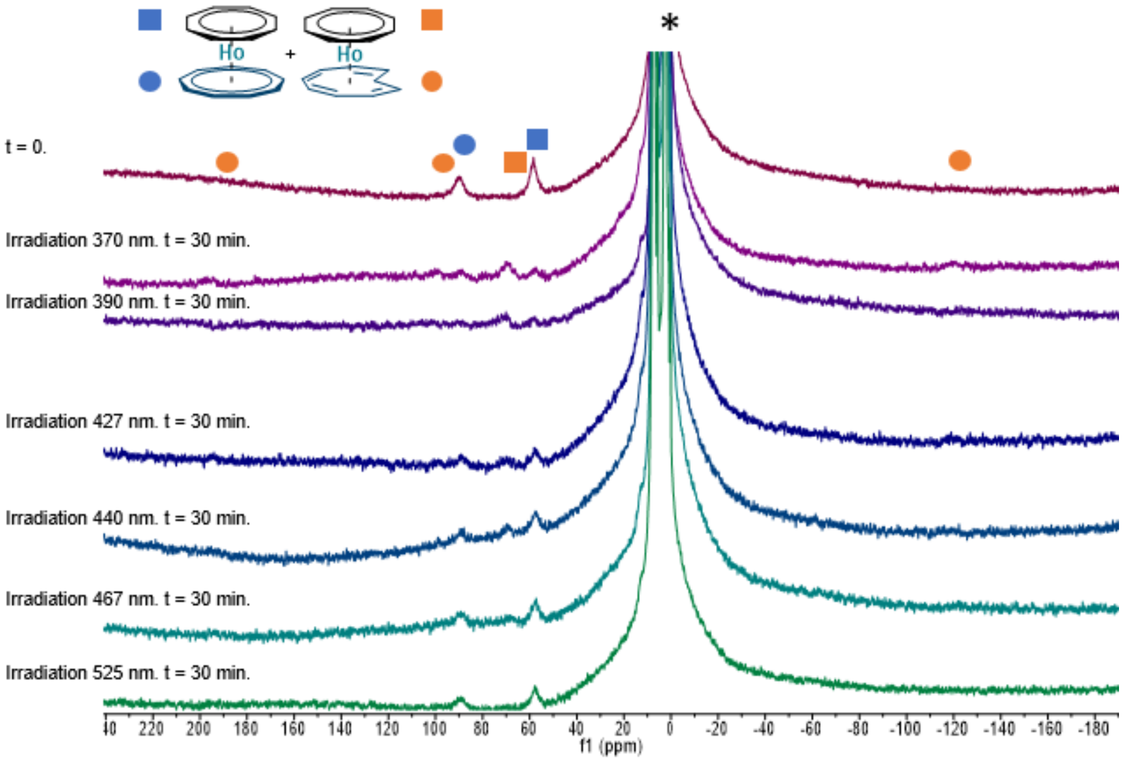

Figure S83: *Isomerization study by ¹H NMR of* ***2***-*Ho in toluene-d$_8$ measured at 293 K at different wavelengths (* residual protio signal of the solvent)*

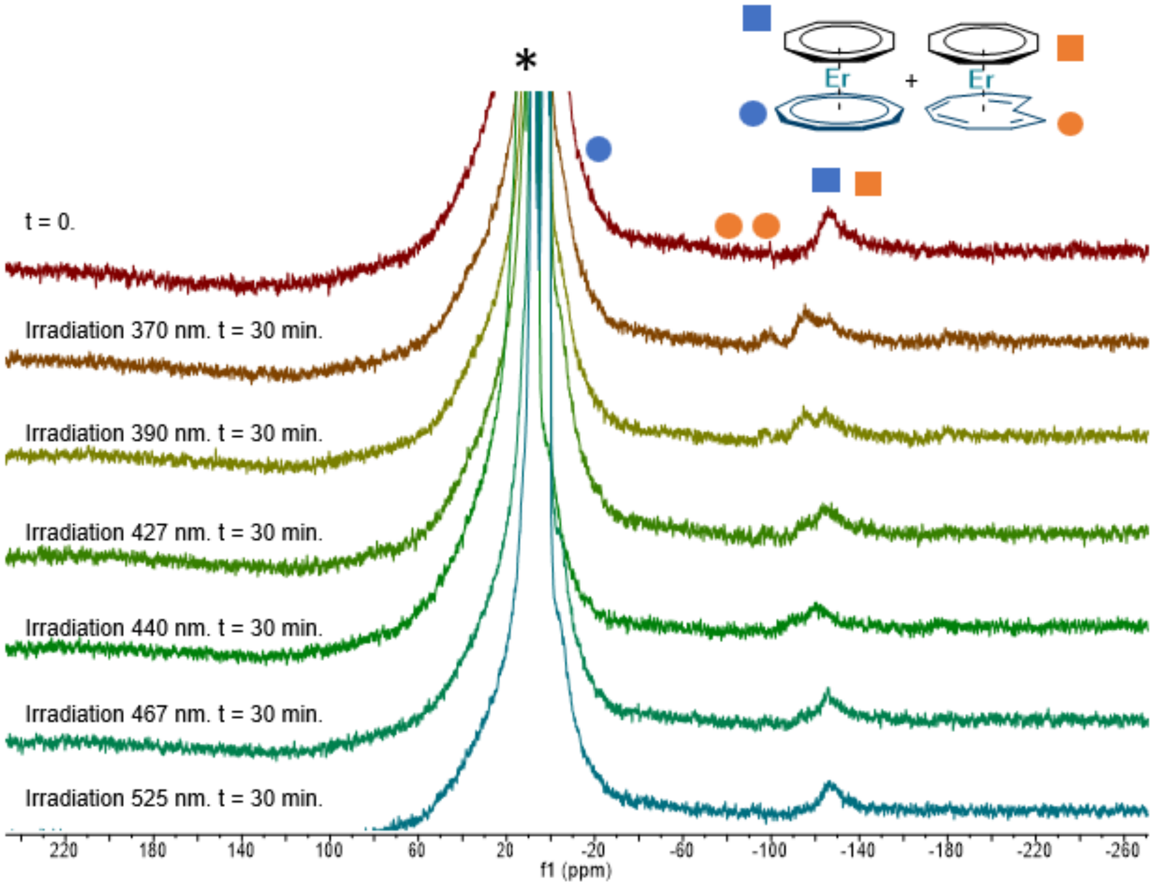

Figure S84: *Isomerization study by ¹H NMR of* ***2***-*Er in toluene-d$_8$ measured at 293 K at different wavelengths (* residual protio signal of the solvent).*

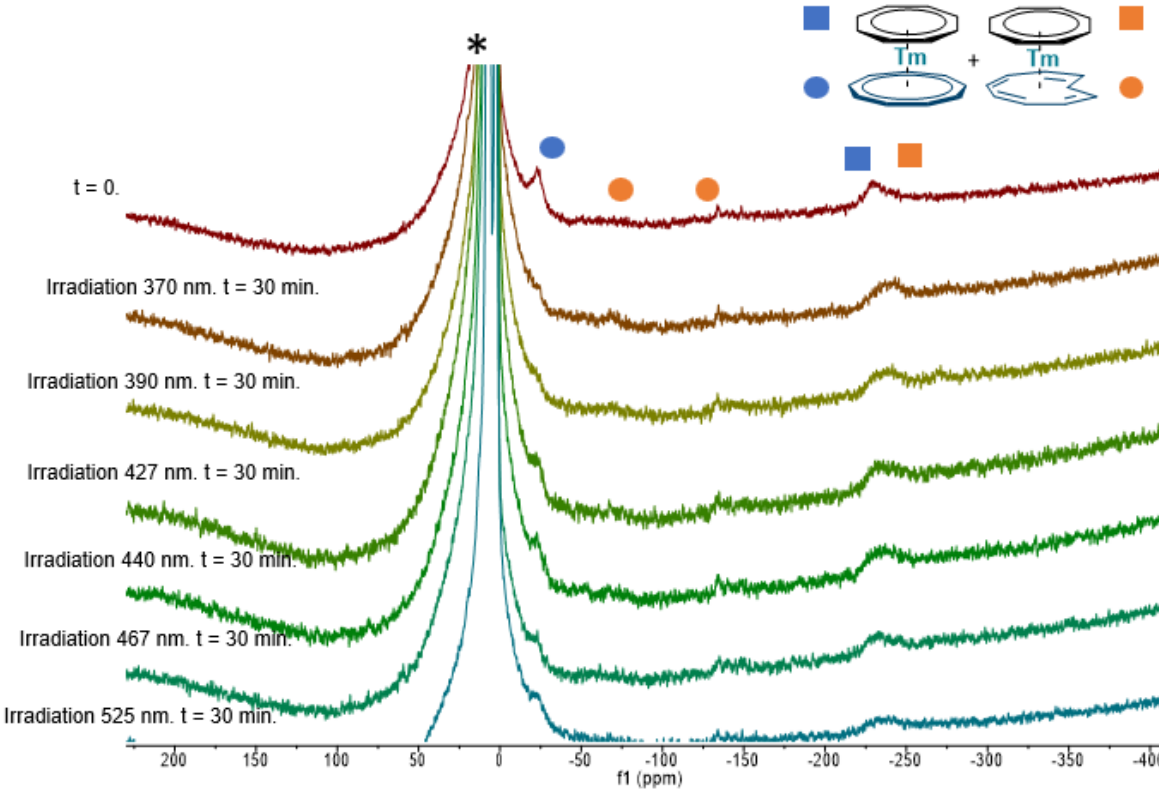

Figure S85*: Isomerization study by* ^{1}*H NMR of* ***2****-Tm in toluene-*d_8 *measured at 293 K at different wavelengths (* residual protio signal of the solvent).*

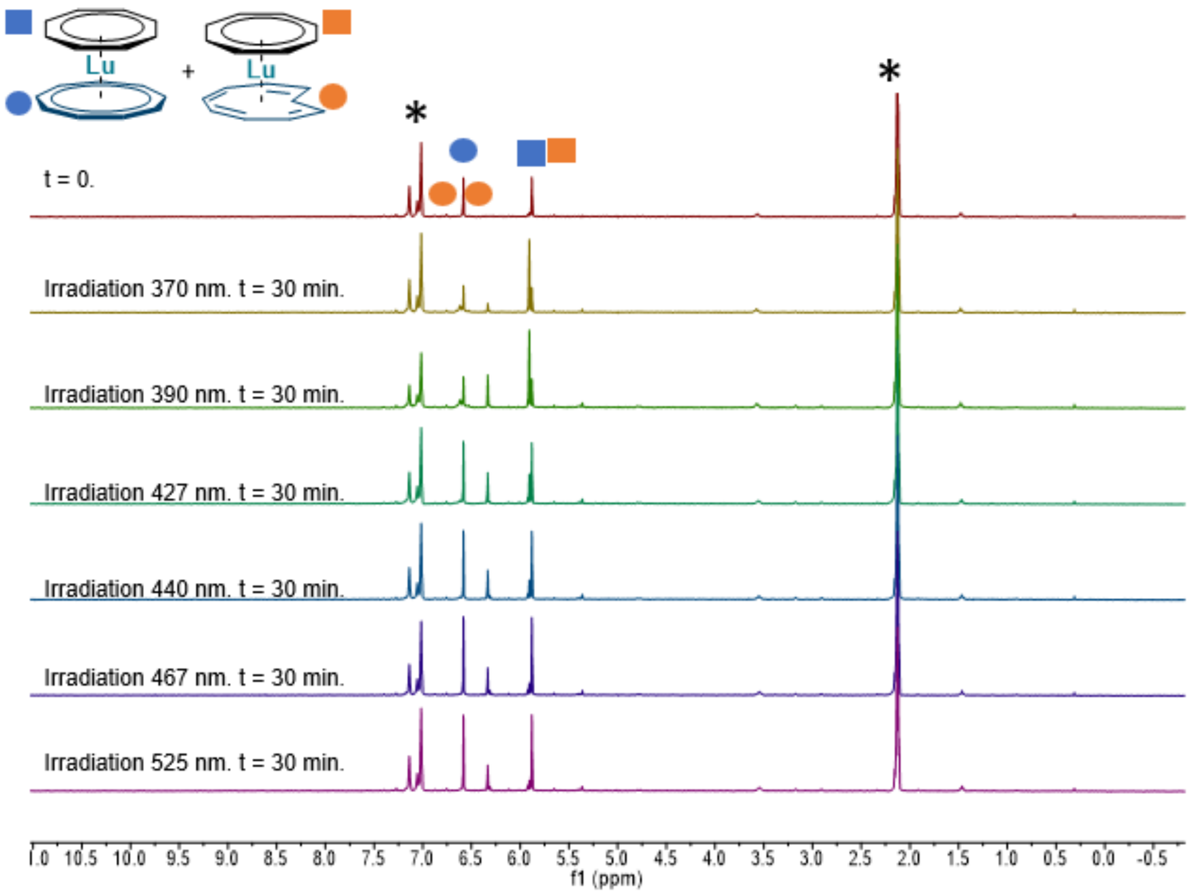

***Figure S86**: Isomerization study by ^{1}H NMR of **2**-Lu in toluene-d_8 measured at 293 K at different wavelengths (* residual protio signal of the solvent).*

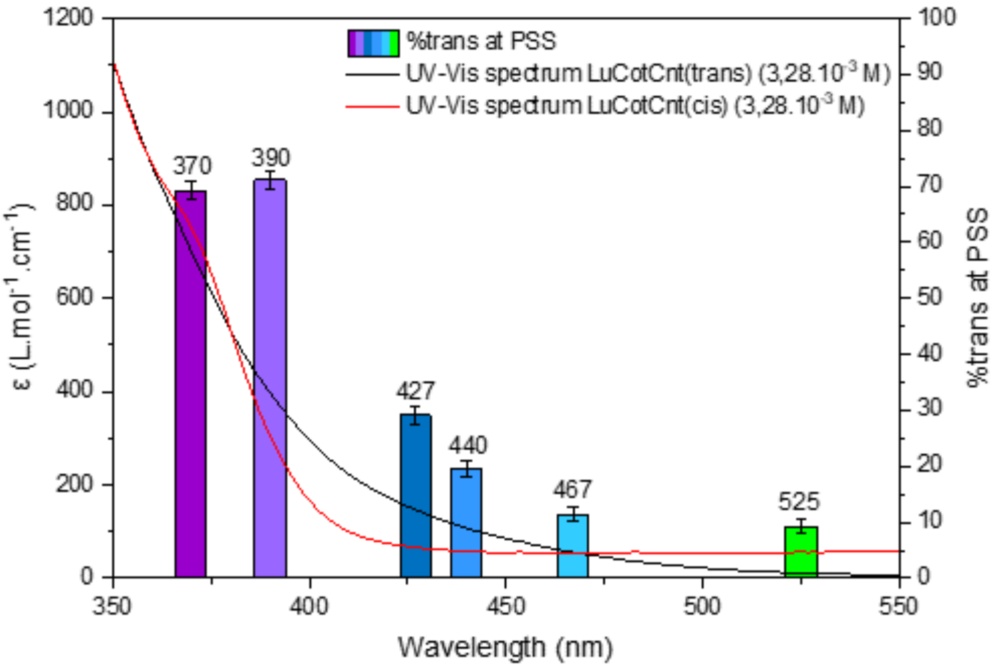

***Figure S87**: Evolution of the trans/cis ratios of **2**-Lu under different wavelengths superimposed with UV spectra of the trans isomers.*

8. Computational details

The optimization of reactants, transition states, IRC, and products were carried out by employing DFT hybrid functional (B3PW91)[5] along with small core pseudopotential Stuttgart basis set[6,7] for samarium atom and Pople basis set[8–10] (6-31G**) for the rest of the atoms. Frequency calculations were performed to locate saddle points for transition state structures, minima for the rest of the structures, and for obtaining thermal corrections over the energies. All the calculations were performed using Gaussian 16 suite of programs.[11] Dispersion corrections were accounted for using the GD3-BJ approach.

NCIS calculations were done using the GIAO method.[12–15]

Energetics (kcal/mol) between two isomers of [Sm(C8H8)(C9H9)], **2-Sm**

	ΔH	ΔG
**1 (S=5/2)**	6.9	4.9
**2 (S=5/2)**	0.0	0.0

Energetics (kcal/mol) between two isomers of [Ce(C8H8)(C9H9)], **2-Ce**

	ΔH	ΔG
**3 (S=1/2)**	3.7	4.3
**4 (S=1/2)**	0.0	0.0

***Table S24**: Selected structural parameters (Dispersion) for **2-Ce-trans**.*

	Bond distance
Ce1-C2	2.98
Ce1-C4	3.97
Ce1-C6	3.00
Ce1-C8	2.91
Ce1-C10	2.86
Ce1-C11	2.66
Ce1-C13	2.66
Ce1-C15	2.66
Ce1-C17	2.67
Ce1-C20	2.98
Ce1-C22	3.07
Ce1-C24	3.00
Ce1-C26	2.91
Ce1-C28	2.66
Ce1-C30	2.66
Ce1-C32	2.66
Ce1-C34	2.67

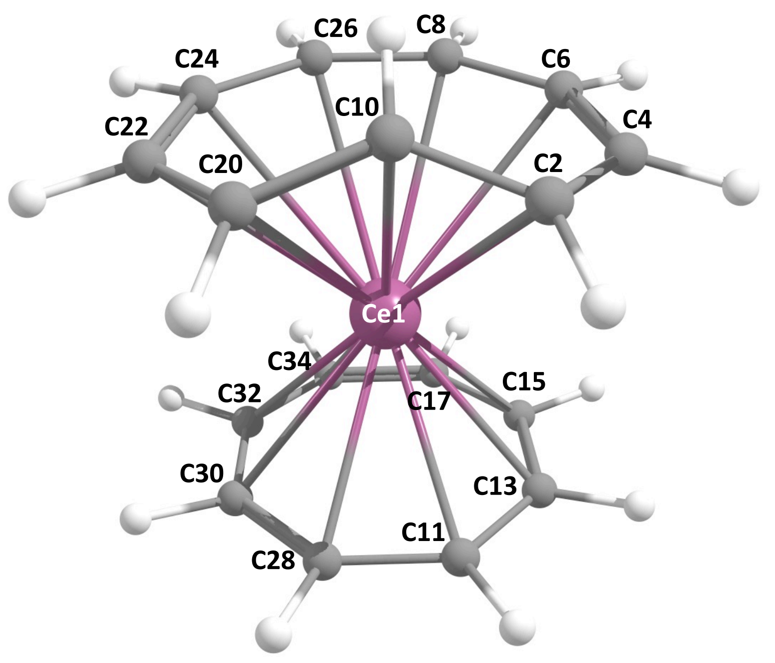

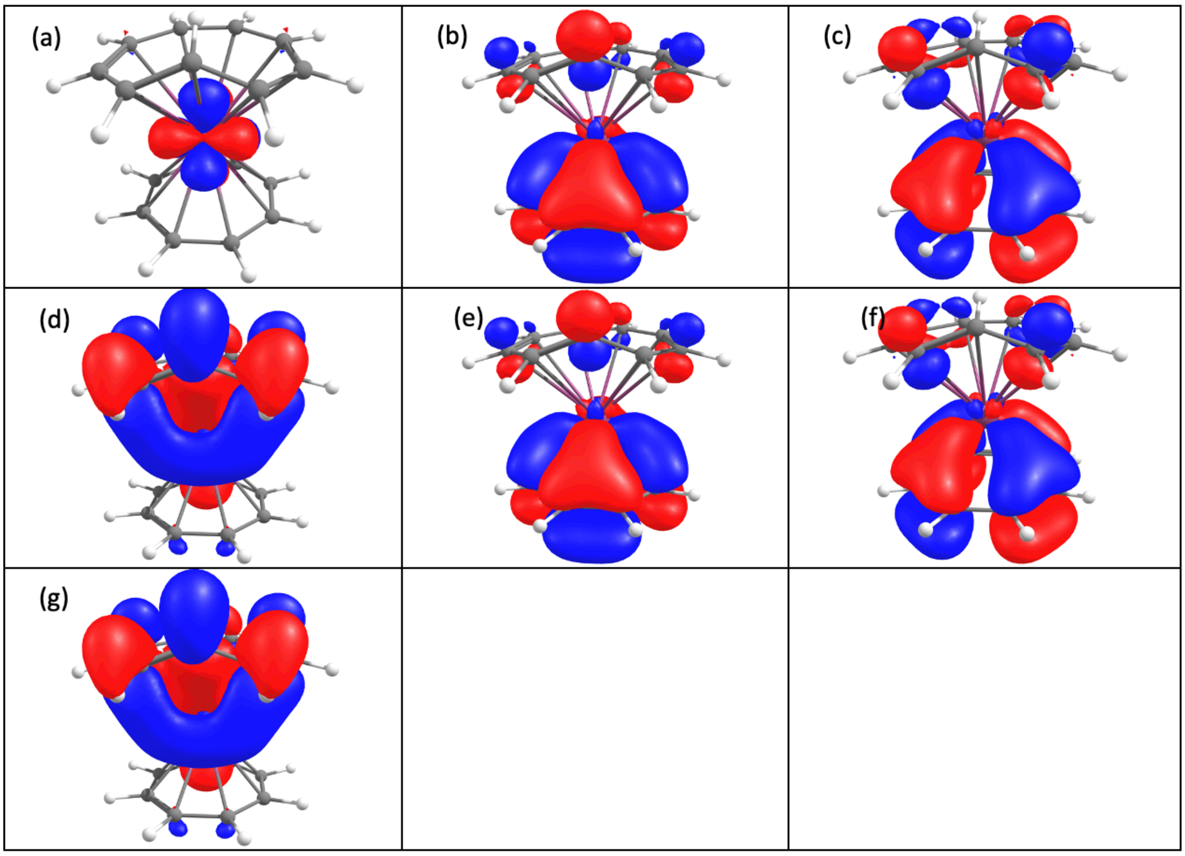

***Figure S88**: Computed MOs for **2-Ce-trans**. (a)AMO-HOMO-2 (b)AMO-HOMO-1 (c)AMO-HOMO (d)AMO-LUMO (e)BMO-HOMO-1 (f)BMO-HOMO (g)BMO-LUMO*

Table S25**: Selected structural parameters (Dispersion) for **2-Ce-cis.

	Bond distance	
Ce1-C2	3.01	
Ce1-C4	3.01	
Ce1-C6	3.02	
Ce1-C8	3.02	
Ce1-C10	2.89	
Ce1-C12	2.89	
Ce1-C14	2.89	
Ce1-C16	2.90	
Ce1-C18	2.89	
Ce1-C20	2.65	
Ce1-C22	2.65	
Ce1-C24	2.65	
Ce1-C26	2.65	
Ce1-C28	2.65	
Ce1-C30	2.65	

Ce1-C32	2.65	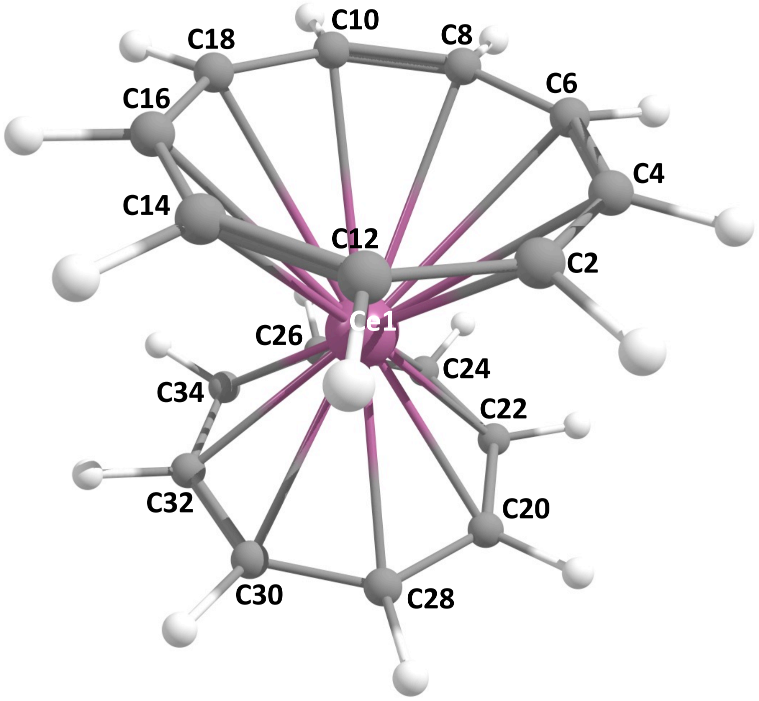
Ce1-C34	2.65	

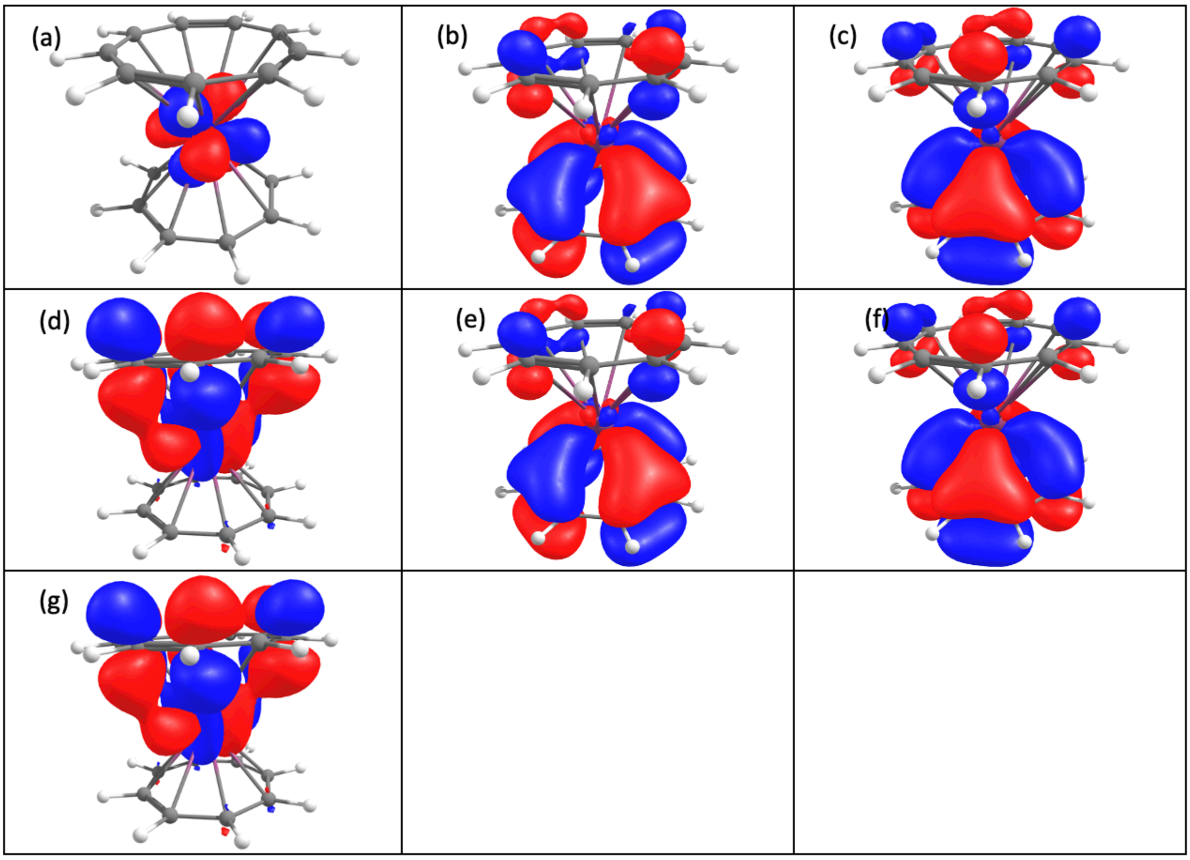

***Figure S89**: Computed MOs for **2-Ce-cis**. (a)AMO-HOMO-2 (b)AMO-HOMO-1 (c)AMO-HOMO (d)AMO-LUMO (e)BMO-HOMO-1 (f)BMO-HOMO (g)BMO-LUMO*

Table S26: *Computed natural charges for* ***2-Ce-trans.***

Atom labels	Natural charges
Ce1	1.36079
C2	-0.29364
C4	-0.28918
C6	-0.33215
C8	-0.36598
C10	-0.31992
C11	-0.37842
C13	-0.37206
C15	-0.37218
C17	-0.37819
C20	-0.29364
C22	-0.28918
C24	-0.33215
C26	-0.36598
C28	-0.37842
C30	-0.37206
C32	-0.37218
C34	-0.37819

Table S27: *Computed Wiberg bond index for* ***2-Ce-trans.***

Atom labels	Wiberg bond index	Atom labels	Wiberg bond index	Atom labels	Wiberg bond index	Atom labels	Wiberg bond index
Ce1	0.0000	Ce1	0.0000	Ce1	0.0000	Ce1	0.0000
C2	0.1055	C4	0.0960	C6	0.1035	C8	0.1097
Atom labels	Wiberg bond index	Atom labels	Wiberg bond index	Atom labels	Wiberg bond index	Atom labels	Wiberg bond index
Ce1	0.0000	Ce1	0.0000	Ce1	0.0000	Ce1	0.0000
C10	0.1277	C11	0.2454	C13	0.2409	C15	0.2388
Atom labels	Wiberg bond index	Atom labels	Wiberg bond index	Atom labels	Wiberg bond index	Atom labels	Wiberg bond index
Ce1	0.0000	Ce1	0.0000	Ce1	0.0000	Ce1	0.0000
C17	0.2422	C20	0.1055	C22	0.0960	C24	0.1035
Atom labels	Wiberg bond index	Atom labels	Wiberg bond index	Atom labels	Wiberg bond index	Atom labels	Wiberg bond index
Ce1	0.0000	Ce1	0.0000	Ce1	0.0000	Ce1	0.0000
C26	0.1097	C28	0.2454	C30	0.2409	C32	0.2388
Atom labels	Wiberg bond index						
Ce1	0.0000						
C34	0.2422						

Table S28**: DFT computed NBO second order perturbation analysis for **2-Ce-trans.

Donor NBO	Acceptor NBO	E(2) kcal/mol
(0.52166) LP (1) C 10<br>s(0.52%)p99.99(99.45%)d 0.06(0.03%)	(0.11692) LV (3)Ce 1<br>s(0.06%)p 3.95(0.24%)d99.99(98.98%)f11.35(0.68%)g 0.73(0.04%)	5.49
(0.52166) LP (1) C 10<br>s(0.52%)p99.99(99.45%)d 0.06(0.03%)	(0.07166) LV (5)Ce 1<br>s(3.03%)p 0.02(0.05%)d19.39(58.80%)f12.56(38.10%)g 0.01(0.02%)	13.79
(0.52166) LP (1) C 10<br>s(0.52%)p99.99(99.45%)d 0.06(0.03%)	(0.04854) LV (7)Ce 1<br>s(92.48%)p 0.01(0.90%)d 0.01(1.25%)f 0.06(5.29%)g 0.00(0.07%)	4.43
(0.83169) BD (2) C 2- C 4<br>(49.91%) 0.7065* C 2<br>s(0.58%)p99.99(99.35%)d 0.12(0.07%)<br>(50.09%) 0.7077* C 4<br>s(0.31%)p99.99(99.64%)d 0.16(0.05%)	(0.11692) LV (3)Ce 1<br>s(0.06%)p 3.95(0.24%)d99.99(98.98%)f11.35(0.68%)g 0.73(0.04%)	3.00
(0.83169) BD (2) C 2- C 4<br>(49.91%) 0.7065* C 2<br>s(0.58%)p99.99(99.35%)d 0.12(0.07%)<br>(50.09%) 0.7077* C 4<br>s(0.31%)p99.99(99.64%)d 0.16(0.05%)	(0.11537) LV (4)Ce 1<br>s(0.00%)p 1.00(0.17%)d99.99(98.31%)f 8.37(1.45%)g 0.40(0.07%)	4.58
(0.98776) BD (1) C 2- C 10<br>(49.37%) 0.7027* C 2 s(35.12%)p 1.85(64.82%)d 0.00(0.06%)<br>(50.63%) 0.7115* C 10 s(36.72%)p 1.72(63.22%)d 0.00(0.06%)	(0.11692) LV (3)Ce 1<br>s(0.06%)p 3.95(0.24%)d99.99(98.98%)f11.35(0.68%)g 0.73(0.04%)	2.77
(0.98757) BD (1) C 4- C 6<br>(49.54%) 0.7038* C 4 s(35.58%)p 1.81(64.36%)d 0.00(0.07%)<br>(50.46%) 0.7104* C 6 s(37.08%)p 1.70(62.86%)d 0.00(0.06%)	(0.11537) LV (4)Ce 1<br>s(0.00%)p 1.00(0.17%)d99.99(98.31%)f 8.37(1.45%)g 0.40(0.07%)	2.43
(0.85222) BD (2) C 6- C 8<br>(49.33%) 0.7024* C 6<br>s(0.40%)p99.99(99.56%)d 0.09(0.03%)<br>(50.67%) 0.7118* C 8<br>s(0.37%)p99.99(99.60%)d 0.09(0.03%)	(0.11692) LV (3)Ce 1<br>s(0.06%)p 3.95(0.24%)d99.99(98.98%)f11.35(0.68%)g 0.73(0.04%)	3.05
(0.85222) BD (2) C 6- C 8<br>(49.33%) 0.7024* C 6<br>s(0.40%)p99.99(99.56%)d 0.09(0.03%)<br>(50.67%) 0.7118* C 8<br>s(0.37%)p99.99(99.60%)d 0.09(0.03%)	(0.11537) LV (4)Ce 1<br>s(0.00%)p 1.00(0.17%)d99.99(98.31%)f 8.37(1.45%)g 0.40(0.07%)	3.60
(0.85222) BD (2) C 6- C 8<br>(49.33%) 0.7024* C 6<br>s(0.40%)p99.99(99.56%)d 0.09(0.03%)<br>(50.67%) 0.7118* C 8<br>s(0.37%)p99.99(99.60%)d 0.09(0.03%)	(0.04854) LV (7)Ce 1<br>s(92.48%)p 0.01(0.90%)d 0.01(1.25%)f 0.06(5.29%)g 0.00(0.07%)	2.65
(0.98541) BD (1) C 8- C 26<br>(50.00%) 0.7071* C 8 s(37.45%)p 1.67(62.49%)d 0.00(0.06%)	(0.11692) LV (3)Ce 1<br>s(0.06%)p 3.95(0.24%)d99.99(98.98%)f11.35(0.68%)g 0.73(0.04%)	3.48

(50.00%) 0.7071* C 26 s(37.45%)p 1.67(62.49%)d 0.00(0.06%)		
(0.98541) BD (1) C 8- C 26 (50.00%) 0.7071* C 8 s(37.45%)p 1.67(62.49%)d 0.00(0.06%) (50.00%) 0.7071* C 26 s(37.45%)p 1.67(62.49%)d 0.00(0.06%)	(0.07166) LV (5)Ce 1 s(3.03%)p 0.02(0.05%)d19.39(58.80%)f12.56(38.10%)g 0.01(0.02%)	2.74
(0.98776) BD (1) C 10- C 20 (50.63%) 0.7115* C 10 s(36.72%)p 1.72(63.22%)d 0.00(0.06%) (49.37%) 0.7027* C 20 s(35.12%)p 1.85(64.82%)d 0.00(0.06%)	(0.11692) LV (3)Ce 1 s(0.06%)p 3.95(0.24%)d99.99(98.98%)f11.35(0.68%)g 0.73(0.04%)	2.77
(0.83170) BD (2) C 20- C 22 (49.91%) 0.7065* C 20 s(0.58%)p99.99(99.35%)d 0.12(0.07%) (50.09%) 0.7077* C 22 s(0.31%)p99.99(99.64%)d 0.16(0.05%)	(0.11692) LV (3)Ce 1 s(0.06%)p 3.95(0.24%)d99.99(98.98%)f11.35(0.68%)g 0.73(0.04%)	3.00
(0.83170) BD (2) C 20- C 22 (49.91%) 0.7065* C 20 s(0.58%)p99.99(99.35%)d 0.12(0.07%) (50.09%) 0.7077* C 22 s(0.31%)p99.99(99.64%)d 0.16(0.05%)	(0.11537) LV (4)Ce 1 s(0.00%)p 1.00(0.17%)d99.99(98.31%)f 8.37(1.45%)g 0.40(0.07%)	4.58
(0.98757) BD (1) C 22- C 24 (49.54%) 0.7038* C 22 s(35.58%)p 1.81(64.36%)d 0.00(0.07%) (50.46%) 0.7104* C 24 s(37.08%)p 1.70(62.86%)d 0.00(0.06%)	(0.11537) LV (4)Ce 1 s(0.00%)p 1.00(0.17%)d99.99(98.31%)f 8.37(1.45%)g 0.40(0.07%)	2.43
(0.85222) BD (2) C 24- C 26 (49.33%) 0.7024* C 24 s(0.40%)p99.99(99.56%)d 0.09(0.03%) (50.67%) 0.7118* C 26 s(0.37%)p99.99(99.60%)d 0.09(0.03%)	(0.11692) LV (3)Ce 1 s(0.06%)p 3.95(0.24%)d99.99(98.98%)f11.35(0.68%)g 0.73(0.04%)	3.05
(0.85222) BD (2) C 24- C 26 (49.33%) 0.7024* C 24 s(0.40%)p99.99(99.56%)d 0.09(0.03%) (50.67%) 0.7118* C 26 s(0.37%)p99.99(99.60%)d 0.09(0.03%)	(0.11537) LV (4)Ce 1 s(0.00%)p 1.00(0.17%)d99.99(98.31%)f 8.37(1.45%)g 0.40(0.07%)	3.60
(0.85222) BD (2) C 24- C 26 (49.33%) 0.7024* C 24 s(0.40%)p99.99(99.56%)d 0.09(0.03%) (50.67%) 0.7118* C 26 s(0.37%)p99.99(99.60%)d 0.09(0.03%)	(0.04854) LV (7)Ce 1 s(92.48%)p 0.01(0.90%)d 0.01(1.25%)f 0.06(5.29%)g 0.00(0.07%)	2.65
(0.97962) BD (1) C 11- C 13 (50.09%) 0.7078* C 11 s(35.29%)p 1.83(64.65%)d 0.00(0.06%) (49.91%) 0.7065* C 13 s(35.38%)p 1.82(64.55%)d 0.00(0.06%)	(0.11692) LV (3)Ce 1 s(0.06%)p 3.95(0.24%)d99.99(98.98%)f11.35(0.68%)g 0.73(0.04%)	4.53
(0.97962) BD (1) C 11- C 13 (50.09%) 0.7078* C 11 s(35.29%)p 1.83(64.65%)d 0.00(0.06%)	(0.11537) LV (4)Ce 1 s(0.00%)p 1.00(0.17%)d99.99(98.31%)f 8.37(1.45%)g 0.40(0.07%)	4.91

(49.91%) 0.7065* C 13 s(35.38%)p<br>1.82(64.55%)d 0.00(0.06%)		
(0.85428) BD (2) C 11- C 13<br>(49.87%) 0.7062* C 11<br>s(1.47%)p66.94(98.47%)d 0.04(0.06%)<br>(50.13%) 0.7080* C 13<br>s(1.42%)p69.36(98.52%)d 0.04(0.06%)	(0.19683) LV (2)Ce 1<br>s(0.00%)p 0.00(0.00%)d 1.00(90.71%)f<br>0.10(9.24%)g 0.00(0.05%)	7.67
(0.85428) BD (2) C 11- C 13<br>(49.87%) 0.7062* C 11<br>s(1.47%)p66.94(98.47%)d 0.04(0.06%)<br>(50.13%) 0.7080* C 13<br>s(1.42%)p69.36(98.52%)d 0.04(0.06%)	(0.11692) LV (3)Ce 1<br>s(0.06%)p 3.95(0.24%)d99.99(<br>98.98%)f11.35(0.68%)g 0.73(0.04%)	5.84
(0.85428) BD (2) C 11- C 13<br>(49.87%) 0.7062* C 11<br>s(1.47%)p66.94(98.47%)d 0.04(0.06%)<br>(50.13%) 0.7080* C 13<br>s(1.42%)p69.36(98.52%)d 0.04(0.06%)	(0.11537) LV (4)Ce 1<br>s(0.00%)p 1.00(0.17%)d99.99(98.31%)f<br>8.37(1.45%)g 0.40(0.07%)	6.97
(0.85428) BD (2) C 11- C 13<br>(49.87%) 0.7062* C 11<br>s(1.47%)p66.94(98.47%)d 0.04(0.06%)<br>(50.13%) 0.7080* C 13<br>s(1.42%)p69.36(98.52%)d 0.04(0.06%)	(0.04854) LV (7)Ce 1<br>s(92.48%)p 0.01(0.90%)d 0.01(1.25%)f<br>0.06(5.29%)g 0.00(0.07%)	3.63
(0.97768) BD (1) C 11- C 28<br>(50.00%) 0.7071* C 11 s(36.66%)p<br>1.73(63.28%)d 0.00(0.06%)<br>(50.00%) 0.7071* C 28 s(36.66%)p<br>1.73(63.28%)d 0.00(0.06%)	(0.11692) LV (3)Ce 1<br>s(0.06%)p 3.95(0.24%)d99.99(<br>98.98%)f11.35(0.68%)g 0.73(0.04%)	10.45
(0.97768) BD (1) C 11- C 28<br>(50.00%) 0.7071* C 11 s(36.66%)p<br>1.73(63.28%)d 0.00(0.06%)<br>(50.00%) 0.7071* C 28 s(36.66%)p<br>1.73(63.28%)d 0.00(0.06%)	(0.04854) LV (7)Ce 1<br>s(92.48%)p 0.01(0.90%)d 0.01(1.25%)f<br>0.06(5.29%)g 0.00(0.07%)	2.78
(0.97749) BD (1) C 13- C 15<br>(50.03%) 0.7073* C 13 s(36.84%)p<br>1.71(63.10%)d 0.00(0.06%)<br>(49.97%) 0.7069* C 15 s(36.77%)p<br>1.72(63.16%)d 0.00(0.07%)	(0.11537) LV (4)Ce 1<br>s(0.00%)p 1.00(0.17%)d99.99(98.31%)f<br>8.37(1.45%)g 0.40(0.07%)	11.16
(0.97749) BD (1) C 13- C 15<br>(50.03%) 0.7073* C 13 s(36.84%)p<br>1.71(63.10%)d 0.00(0.06%)<br>(49.97%) 0.7069* C 15 s(36.77%)p<br>1.72(63.16%)d 0.00(0.07%)	(0.04854) LV (7)Ce 1<br>s(92.48%)p 0.01(0.90%)d 0.01(1.25%)f<br>0.06(5.29%)g 0.00(0.07%)	2.67
(0.97994) BD (1) C 15- C 17<br>(49.95%) 0.7067* C 15 s(35.42%)p<br>1.82(64.52%)d 0.00(0.06%)<br>(50.05%) 0.7075* C 17 s(35.32%)p<br>1.83(64.61%)d 0.00(0.06%)	(0.11692) LV (3)Ce 1<br>s(0.06%)p 3.95(0.24%)d99.99(<br>98.98%)f11.35(0.68%)g 0.73(0.04%)	5.11
(0.97994) BD (1) C 15- C 17<br>(49.95%) 0.7067* C 15 s(35.42%)p<br>1.82(64.52%)d 0.00(0.06%)	(0.11537) LV (4)Ce 1<br>s(0.00%)p 1.00(0.17%)d99.99(98.31%)f<br>8.37(1.45%)g 0.40(0.07%)	4.85

(50.05%) 0.7075* C 17 s(35.32%)p 1.83(64.61%)d 0.00(0.06%)		
(0.97994) BD (1) C 15- C 17 (49.95%) 0.7067* C 15 s(35.42%)p 1.82(64.52%)d 0.00(0.06%) (50.05%) 0.7075* C 17 s(35.32%)p 1.83(64.61%)d 0.00(0.06%)	(0.04854) LV (7)Ce 1 s(92.48%)p 0.01(0.90%)d 0.01(1.25%)f 0.06(5.29%)g 0.00(0.07%)	2.34
(0.85292) BD (2) C 15- C 17 (49.82%) 0.7059* C 15 s(1.41%)p69.96(98.53%)d 0.04(0.06%) (50.18%) 0.7084* C 17 s(1.40%)p70.43(98.54%)d 0.04(0.06%)	(0.19683) LV (2)Ce 1 s(0.00%)p 0.00(0.00%)d 1.00(90.71%)f 0.10(9.24%)g 0.00(0.05%)	8.50
(0.85292) BD (2) C 15- C 17 (49.82%) 0.7059* C 15 s(1.41%)p69.96(98.53%)d 0.04(0.06%) (50.18%) 0.7084* C 17 s(1.40%)p70.43(98.54%)d 0.04(0.06%)	(0.11692) LV (3)Ce 1 s(0.06%)p 3.95(0.24%)d99.99(98.98%)f11.35(0.68%)g 0.73(0.04%)	6.75
(0.85292) BD (2) C 15- C 17 (49.82%) 0.7059* C 15 s(1.41%)p69.96(98.53%)d 0.04(0.06%) (50.18%) 0.7084* C 17 s(1.40%)p70.43(98.54%)d 0.04(0.06%)	(0.11537) LV (4)Ce 1 s(0.00%)p 1.00(0.17%)d99.99(98.31%)f 8.37(1.45%)g 0.40(0.07%)	6.98
(0.85292) BD (2) C 15- C 17 (49.82%) 0.7059* C 15 s(1.41%)p69.96(98.53%)d 0.04(0.06%) (50.18%) 0.7084* C 17 s(1.40%)p70.43(98.54%)d 0.04(0.06%)	(0.04854) LV (7)Ce 1 s(92.48%)p 0.01(0.90%)d 0.01(1.25%)f 0.06(5.29%)g 0.00(0.07%)	4.00
(0.97823) BD (1) C 17- C 34 (50.00%) 0.7071* C 17 s(36.71%)p 1.72(63.23%)d 0.00(0.06%) (50.00%) 0.7071* C 34 s(36.71%)p 1.72(63.23%)d 0.00(0.06%)	(0.11692) LV (3)Ce 1 s(0.06%)p 3.95(0.24%)d99.99(98.98%)f11.35(0.68%)g 0.73(0.04%)	11.43
(0.97823) BD (1) C 17- C 34 (50.00%) 0.7071* C 17 s(36.71%)p 1.72(63.23%)d 0.00(0.06%) (50.00%) 0.7071* C 34 s(36.71%)p 1.72(63.23%)d 0.00(0.06%)		
(0.97962) BD (1) C 28- C 30 (50.09%) 0.7078* C 28 s(35.29%)p 1.83(64.65%)d 0.00(0.06%) (49.91%) 0.7065* C 30 s(35.38%)p 1.82(64.55%)d 0.00(0.06%)	(0.11692) LV (3)Ce 1 s(0.06%)p 3.95(0.24%)d99.99(98.98%)f11.35(0.68%)g 0.73(0.04%)	4.53
(0.97962) BD (1) C 28- C 30 (50.09%) 0.7078* C 28 s(35.29%)p 1.83(64.65%)d 0.00(0.06%) (49.91%) 0.7065* C 30 s(35.38%)p 1.82(64.55%)d 0.00(0.06%)	(0.11537) LV (4)Ce 1 s(0.00%)p 1.00(0.17%)d99.99(98.31%)f 8.37(1.45%)g 0.40(0.07%)	4.92
(0.85428) BD (2) C 28- C 30 (49.87%) 0.7062* C 28 s(1.47%)p66.94(98.47%)d 0.04(0.06%)	(0.19683) LV (2)Ce 1 s(0.00%)p 0.00(0.00%)d 1.00(90.71%)f 0.10(9.24%)g 0.00(0.05%)	7.67

(50.13%) 0.7080* C 30<br>s(1.42%)p69.36(98.52%)d 0.04(0.06%)		
(0.85428) BD (2) C 28- C 30<br>(49.87%) 0.7062* C 28<br>s(1.47%)p66.94(98.47%)d 0.04(0.06%)<br>(50.13%) 0.7080* C 30<br>s(1.42%)p69.36(98.52%)d 0.04(0.06%)	(0.11692) LV (3)Ce 1<br>s(0.06%)p 3.95(0.24%)d99.99(98.98%)f11.35(0.68%)g 0.73(0.04%)	5.84
(0.85428) BD (2) C 28- C 30<br>(49.87%) 0.7062* C 28<br>s(1.47%)p66.94(98.47%)d 0.04(0.06%)<br>(50.13%) 0.7080* C 30<br>s(1.42%)p69.36(98.52%)d 0.04(0.06%)	(0.11537) LV (4)Ce 1<br>s(0.00%)p 1.00(0.17%)d99.99(98.31%)f 8.37(1.45%)g 0.40(0.07%)	6.97
(0.85428) BD (2) C 28- C 30<br>(49.87%) 0.7062* C 28<br>s(1.47%)p66.94(98.47%)d 0.04(0.06%)<br>(50.13%) 0.7080* C 30<br>s(1.42%)p69.36(98.52%)d 0.04(0.06%)	(0.04854) LV (7)Ce 1<br>s(92.48%)p 0.01(0.90%)d 0.01(1.25%)f 0.06(5.29%)g 0.00(0.07%)	3.63
(0.97749) BD (1) C 30- C 32<br>(50.03%) 0.7073* C 30 s(36.84%)p 1.71(63.10%)d 0.00(0.06%)<br>(49.97%) 0.7069* C 32 s(36.77%)p 1.72(63.16%)d 0.00(0.07%)	(0.11537) LV (4)Ce 1<br>s(0.00%)p 1.00(0.17%)d99.99(98.31%)f 8.37(1.45%)g 0.40(0.07%)	11.16
(0.97994) BD (1) C 32- C 34<br>(49.95%) 0.7067* C 32 s(35.42%)p 1.82(64.52%)d 0.00(0.06%)<br>(50.05%) 0.7075* C 34 s(35.32%)p 1.83(64.61%)d 0.00(0.06%)	(0.11692) LV (3)Ce 1<br>s(0.06%)p 3.95(0.24%)d99.99(98.98%)f11.35(0.68%)g 0.73(0.04%)	5.11
(0.97994) BD (1) C 32- C 34<br>(49.95%) 0.7067* C 32 s(35.42%)p 1.82(64.52%)d 0.00(0.06%)<br>(50.05%) 0.7075* C 34 s(35.32%)p 1.83(64.61%)d 0.00(0.06%)	(0.11537) LV (4)Ce 1<br>s(0.00%)p 1.00(0.17%)d99.99(98.31%)f 8.37(1.45%)g 0.40(0.07%)	4.84
(0.85292) BD (2) C 32- C 34<br>(49.82%) 0.7059* C 32<br>s(1.41%)p69.96(98.53%)d 0.04(0.06%)<br>(50.18%) 0.7084* C 34<br>s(1.40%)p70.43(98.54%)d 0.04(0.06%)	(0.19683) LV (2)Ce 1<br>s(0.00%)p 0.00(0.00%)d 1.00(90.71%)f 0.10(9.24%)g 0.00(0.05%)	8.50
(0.85292) BD (2) C 32- C 34<br>(49.82%) 0.7059* C 32<br>s(1.41%)p69.96(98.53%)d 0.04(0.06%)<br>(50.18%) 0.7084* C 34<br>s(1.40%)p70.43(98.54%)d 0.04(0.06%)	(0.11692) LV (3)Ce 1<br>s(0.06%)p 3.95(0.24%)d99.99(98.98%)f11.35(0.68%)g 0.73(0.04%)	6.75
(0.85292) BD (2) C 32- C 34<br>(49.82%) 0.7059* C 32<br>s(1.41%)p69.96(98.53%)d 0.04(0.06%)<br>(50.18%) 0.7084* C 34<br>s(1.40%)p70.43(98.54%)d 0.04(0.06%)	(0.11537) LV (4)Ce 1<br>s(0.00%)p 1.00(0.17%)d99.99(98.31%)f 8.37(1.45%)g 0.40(0.07%)	6.97
(0.85292) BD (2) C 32- C 34<br>(49.82%) 0.7059* C 32<br>s(1.41%)p69.96(98.53%)d 0.04(0.06%)	(0.04854) LV (7)Ce 1<br>s(92.48%)p 0.01(0.90%)d 0.01(1.25%)f 0.06(5.29%)g 0.00(0.07%)	4.00

(50.18%) 0.7084* C 34<br>s(1.40%)p70.43(98.54%)d 0.04(0.06%)		

*Table S29: NBO analysis of canonical molecular orbitals for **2-Sm-trans.***

HOMO-2, MO 73 (occ): orbital energy = -0.19354 a.u.<br>0.983*[31]: LP (1)Ce 1(lp)	(0.98653) LP (1)Ce 1<br>s(0.01%)p 0.16(0.00%)d15.14(0.22%) f99.99(99.76%)g 0.04(0.00%)
HOMO-1, MO 74 (occ): orbital energy = -0.18388 a.u.<br>-0.423*[127]: BD*(2) C32-C34*<br>-0.423*[108]: BD*(2) C15-C17*<br>0.422*[122]: BD*(2) C28-C30*<br>0.422*[75]: BD*(2) C11- C13*<br>0.329*[76]: LV (1)Ce 1(lv)	(0.27020) BD*(2) C 32- C 34<br>(50.18%) 0.7084* C 32 s(1.41%)p69.96(98.53%)d 0.04(0.06%)<br>(49.82%) -0.7059* C 34 s(1.40%)p70.43(98.54%)d 0.04(0.06%)<br>(0.27020) BD*(2) C 15- C 17<br>(50.18%) 0.7084* C 15 s(1.41%)p69.96(98.53%)d 0.04(0.06%)<br>(49.82%) -0.7059* C 17 s(1.40%)p70.43(98.54%)d 0.04(0.06%)<br>(0.27043) BD*(2) C 28- C 30<br>(50.13%) 0.7080* C 28 s(1.47%)p66.94(98.47%)d 0.04(0.06%)<br>(49.87%) -0.7062* C 30 s(1.42%)p69.36(98.52%)d 0.04(0.06%)<br>(0.27043) BD*(2) C 11- C 13<br>(50.13%) 0.7080* C 11 s(1.47%)p66.94(98.47%)d 0.04(0.06%)<br>(49.87%) -0.7062* C 13 s(1.42%)p69.36(98.52%)d 0.04(0.06%)<br>(0.19843) LV (1)Ce 1<br>s(0.01%)p 0.06(0.00%)d99.99(89.61%) f99.99(10.33%)g 3.87(0.05%)
HOMO, MO 75 (occ): orbital energy = -0.18307 a.u.<br>-0.421*[73]: BD (2) C32- C34<br>0.421*[54]: BD (2) C15- C17<br>0.421*[68]: BD (2) C28- C30<br>-0.421*[48]: BD (2) C11- C13<br>0.315*[77]: LV (2)Ce 1(lv)	(0.85292) BD (2) C 32- C 34<br>(49.82%) 0.7059* C 32 s(1.41%)p69.96(98.53%)d 0.04(0.06%)<br>(50.18%) 0.7084* C 34 s(1.40%)p70.43(98.54%)d 0.04(0.06%)<br>(0.85292) BD (2) C 15- C 17<br>(49.82%) 0.7059* C 15 s(1.41%)p69.96(98.53%)d 0.04(0.06%)<br>(50.18%) 0.7084* C 17 s(1.40%)p70.43(98.54%)d 0.04(0.06%)<br>(0.85428) BD (2) C 28- C 30<br>(49.87%) 0.7062* C 28 s(1.47%)p66.94(98.47%)d 0.04(0.06%)<br>(50.13%) 0.7080* C 30 s(1.42%)p69.36(98.52%)d 0.04(0.06%)<br>(0.85428) BD (2) C 11- C 13<br>(49.87%) 0.7062* C 11 s(1.47%)p66.94(98.47%)d 0.04(0.06%)<br>(50.13%) 0.7080* C 13 s(1.42%)p69.36(98.52%)d 0.04(0.06%)<br>(0.19683) LV (2)Ce 1

	s(0.00%)p 0.00(0.00%)d 1.00(90.71%) f 0.10(9.24%)g 0.00(0.05%)

Table S30**: Computed natural charges for **2-Ce-cis.

Atom labels	Natural charges
Ce1	1.41772
C2	-0.32989
C4	-0.32459
C6	-0.32283
C8	-0.32257
C10	-0.32787
C12	-0.32459
C14	-0.32283
C16	-0.32257
C18	-0.32787
C20	-0.37885
C22	-0.37291
C24	-0.37293
C26	-0.37875
C28	-0.37885
C30	-0.37291
C32	-0.37292
C34	-0.37875

Table S31**: Computed Wiberg bond index for **2-Ce-cis.

Atom labels	Wiberg bond index	Atom labels	Wiberg bond index	Atom labels	Wiberg bond index	Atom labels	Wiberg bond index
Ce1	0.0000	Ce1	0.0000	Ce1	0.0000	Ce1	0.0000
C2	0.0918	C4	0.0915	C6	0.0912	C8	0.0912
Atom labels	Wiberg bond index	Atom labels	Wiberg bond index	Atom labels	Wiberg bond index	Atom labels	Wiberg bond index
Ce1	0.0000	Ce1	0.0000	Ce1	0.0000	Ce1	0.0000
C10	0.0918	C12	0.0915	C14	0.0912	C16	0.0912
Atom labels	Wiberg bond index	Atom labels	Wiberg bond index	Atom labels	Wiberg bond index	Atom labels	Wiberg bond index
Ce1	0.0000	Ce1	0.0000	Ce1	0.0000	Ce1	0.0000
C18	0.0918	C20	0.2433	C22	0.2423	C24	0.2423
Atom labels	Wiberg bond index	Atom labels	Wiberg bond index	Atom labels	Wiberg bond index	Atom labels	Wiberg bond index
Ce1	0.0000	Ce1	0.0000	Ce1	0.0000	Ce1	0.0000
C26	0.2434	C28	0.2433	C30	0.2423	C32	0.2423
Atom labels	Wiberg bond index						
Ce1	0.0000						
C34	0.2434						

Table S32**: DFT computed NBO second-order perturbation analysis for **2-Ce-cis.

Donor NBO	Acceptor NBO	E(2) kcal/mol
(0.53224) LP (1) C 6 s(0.38%)p99.99(99.60%)d 0.04(0.01%)	(0.11283) LV (4)Ce 1 s(0.00%)p 1.00(0.21%)d99.99(98.18%)f 7.40(1.57%)g 0.20(0.04%)	4.24
(0.84389) BD (2) C 2- C 4 (50.03%) 0.7073* C 2 s(0.15%)p99.99(99.82%)d 0.21(0.03%) (49.97%) 0.7069* C 4 s(0.15%)p99.99(99.82%)d 0.22(0.03%)	(0.11335) LV (3)Ce 1 s(0.00%)p 1.00(0.26%)d99.99(98.12%)f 6.01(1.57%)g 0.17(0.04%)	4.94
(0.98692) BD (1) C 2- C 12 (50.07%) 0.7076* C 2 s(37.32%)p 1.68(62.62%)d 0.00(0.06%) (49.93%) 0.7066* C 12 s(37.36%)p 1.67(62.58%)d 0.00(0.06%)	(0.11335) LV (3)Ce 1 s(0.00%)p 1.00(0.26%)d99.99(98.12%)f 6.01(1.57%)g 0.17(0.04%)	2.58
(0.98638) BD (1) C 4- C 6 (50.02%) 0.7073* C 4 s(37.31%)p 1.68(62.64%)d 0.00(0.06%) (49.98%) 0.7069* C 6 s(37.21%)p 1.69(62.73%)d 0.00(0.06%)	(0.11283) LV (4)Ce 1 s(0.00%)p 1.00(0.21%)d99.99(98.18%)f 7.40(1.57%)g 0.20(0.04%)	2.04
(0.98617) BD (1) C 6- C 8 (50.01%) 0.7072* C 6 s(37.19%)p 1.69(62.75%)d 0.00(0.06%) (49.99%) 0.7070* C 8 s(37.33%)p 1.68(62.61%)d 0.00(0.06%)	(0.11283) LV (4)Ce 1 s(0.00%)p 1.00(0.21%)d99.99(98.18%)f 7.40(1.57%)g 0.20(0.04%)	2.63
(0.84394) BD (2) C 8- C 10 (49.97%) 0.7069* C 8 s(0.15%)p99.99(99.81%)d 0.21(0.03%) (50.03%) 0.7073* C 10 s(0.15%)p99.99(99.81%)d 0.21(0.03%)	(0.11335) LV (3)Ce 1 s(0.00%)p 1.00(0.26%)d99.99(98.12%)f 6.01(1.57%)g 0.17(0.04%)	3.28
(0.84394) BD (2) C 8- C 10 (49.97%) 0.7069* C 8 s(0.15%)p99.99(99.81%)d 0.21(0.03%) (50.03%) 0.7073* C 10 s(0.15%)p99.99(99.81%)d 0.21(0.03%)	(0.11283) LV (4)Ce 1 s(0.00%)p 1.00(0.21%)d99.99(98.18%)f 7.40(1.57%)g 0.20(0.04%)	2.27
(0.98699) BD (1) C 10- C 18 (50.00%) 0.7071* C 10 s(37.34%)p 1.68(62.60%)d 0.00(0.06%) (50.00%) 0.7071* C 18 s(37.34%)p 1.68(62.60%)d 0.00(0.06%)	(0.11335) LV (3)Ce 1 s(0.00%)p 1.00(0.26%)d99.99(98.12%)f 6.01(1.57%)g 0.17(0.04%)	2.93
(0.98660) BD (1) C 12- C 14 (50.04%) 0.7074* C 12 s(37.18%)p 1.69(62.76%)d 0.00(0.06%) (49.96%) 0.7068* C 14 s(37.23%)p 1.68(62.71%)d 0.00(0.06%)	(0.11283) LV (4)Ce 1 s(0.00%)p 1.00(0.21%)d99.99(98.18%)f 7.40(1.57%)g 0.20(0.04%)	2.01

(0.84384) BD (2) C 12- C 14<br>(50.01%) 0.7072* C 12 s(0.16%)p99.99(99.81%)d 0.21(0.03%)<br>(49.99%) 0.7071* C 14 s(0.16%)p99.99(99.81%)d 0.21(0.03%)	(0.11283) LV (4)Ce 1<br>s(0.00%)p 1.00(0.21%)d99.99(98.18%)f 7.40(1.57%)g 0.20(0.04%)	4.14
(0.98618) BD (1) C 14- C 16<br>(50.00%) 0.7071* C 14 s(37.35%)p 1.68(62.59%)d 0.00(0.06%)<br>(50.00%) 0.7071* C 16 s(37.33%)p 1.68(62.61%)d 0.00(0.06%)	(0.11283) LV (4)Ce 1<br>s(0.00%)p 1.00(0.21%)d99.99(98.18%)f 7.40(1.57%)g 0.20(0.04%)	2.72
(0.84394) BD (2) C 16- C 18<br>(49.97%) 0.7069* C 16 s(0.15%)p99.99(99.81%)d 0.21(0.03%)<br>(50.03%) 0.7073* C 18 s(0.15%)p99.99(99.81%)d 0.21(0.03%)	(0.11335) LV (3)Ce 1<br>s(0.00%)p 1.00(0.26%)d99.99(98.12%)f 6.01(1.57%)g 0.17(0.04%)	3.27
(0.84394) BD (2) C 16- C 18<br>(49.97%) 0.7069* C 16 s(0.15%)p99.99(99.81%)d 0.21(0.03%)<br>(50.03%) 0.7073* C 18 s(0.15%)p99.99(99.81%)d 0.21(0.03%)	(0.11283) LV (4)Ce 1<br>s(0.00%)p 1.00(0.21%)d99.99(98.18%)f 7.40(1.57%)g 0.20(0.04%)	2.27
(0.97959) BD (1) C 20- C 22<br>(50.08%) 0.7077* C 20 s(35.29%)p 1.83(64.64%)d 0.00(0.06%)<br>(49.92%) 0.7066* C 22 s(35.36%)p 1.83(64.58%)d 0.00(0.06%)	(0.11335) LV (3)Ce 1<br>s(0.00%)p 1.00(0.26%)d99.99(98.12%)f 6.01(1.57%)g 0.17(0.04%)	4.86
(0.97959) BD (1) C 20- C 22<br>(50.08%) 0.7077* C 20 s(35.29%)p 1.83(64.64%)d 0.00(0.06%)<br>(49.92%) 0.7066* C 22 s(35.36%)p 1.83(64.58%)d 0.00(0.06%)	(0.11283) LV (4)Ce 1<br>s(0.00%)p 1.00(0.21%)d99.99(98.18%)f 7.40(1.57%)g 0.20(0.04%)	4.86
(0.97959) BD (1) C 20- C 22<br>(50.08%) 0.7077* C 20 s(35.29%)p 1.83(64.64%)d 0.00(0.06%)<br>(49.92%) 0.7066* C 22 s(35.36%)p 1.83(64.58%)d 0.00(0.06%)	(0.04563) LV (7)Ce 1<br>s(96.49%)p 0.02(1.59%)d 0.01(0.71%)f 0.01(1.10%)g 0.00(0.11%)	2.42
(0.85266) BD (2) C 20- C 22<br>(50.06%) 0.7075* C 20 s(1.44%)p68.31(98.50%)d 0.04(0.06%)<br>(49.94%) 0.7067* C 22 s(1.42%)p69.19(98.51%)d 0.04(0.06%)	(0.19853) LV (2)Ce 1<br>s(0.00%)p 0.00(0.00%)d 1.00(89.04%)f 0.12(10.92%)g 0.00(0.04%)	8.15
(0.85266) BD (2) C 20- C 22<br>(50.06%) 0.7075* C 20 s(1.44%)p68.31(98.50%)d 0.04(0.06%)<br>(49.94%) 0.7067* C 22 s(1.42%)p69.19(98.51%)d 0.04(0.06%)	(0.11335) LV (3)Ce 1<br>s(0.00%)p 1.00(0.26%)d99.99(98.12%)f 6.01(1.57%)g 0.17(0.04%)	6.70
(0.85266) BD (2) C 20- C 22	(0.11283) LV (4)Ce 1<br>s(0.00%)p 1.00(0.21%)d99.99(98.18%)f 7.40(1.57%)g 0.20(0.04%)	6.75

(50.06%) 0.7075* C 20 s(1.44%)p68.31(98.50%)d 0.04(0.06%)<br>(49.94%) 0.7067* C 22 s(1.42%)p69.19(98.51%)d 0.04(0.06%)		
(0.85266) BD (2) C 20- C 22<br>(50.06%) 0.7075* C 20 s(1.44%)p68.31(98.50%)d 0.04(0.06%)<br>(49.94%) 0.7067* C 22 s(1.42%)p69.19(98.51%)d 0.04(0.06%)	(0.04563) LV (7)Ce 1<br>s(96.49%)p 0.02(1.59%)d 0.01(0.71%)f 0.01(1.10%)g 0.00(0.11%)	3.40
(0.97780) BD (1) C 20- C 28<br>(50.00%) 0.7071* C 20 s(36.76%)p 1.72(63.17%)d 0.00(0.06%)<br>(50.00%) 0.7071* C 28 s(36.76%)p 1.72(63.17%)d 0.00(0.06%)	(0.11335) LV (3)Ce 1<br>s(0.00%)p 1.00(0.26%)d99.99(98.12%)f 6.01(1.57%)g 0.17(0.04%)	11.21
(0.97780) BD (1) C 20- C 28<br>(50.00%) 0.7071* C 20 s(36.76%)p 1.72(63.17%)d 0.00(0.06%)<br>(50.00%) 0.7071* C 28 s(36.76%)p 1.72(63.17%)d 0.00(0.06%)	(0.04563) LV (7)Ce 1<br>s(96.49%)p 0.02(1.59%)d 0.01(0.71%)f 0.01(1.10%)g 0.00(0.11%)	2.86
(0.97717) BD (1) C 22- C 24<br>(50.00%) 0.7071* C 22 s(36.77%)p 1.72(63.16%)d 0.00(0.07%)<br>(50.00%) 0.7071* C 24 s(36.77%)p 1.72(63.16%)d 0.00(0.07%)	(0.11283) LV (4)Ce 1<br>s(0.00%)p 1.00(0.21%)d99.99(98.18%)f 7.40(1.57%)g 0.20(0.04%)	11.01
(0.97717) BD (1) C 22- C 24<br>(50.00%) 0.7071* C 22 s(36.77%)p 1.72(63.16%)d 0.00(0.07%)<br>(50.00%) 0.7071* C 24 s(36.77%)p 1.72(63.16%)d 0.00(0.07%)	(0.04563) LV (7)Ce 1<br>s(96.49%)p 0.02(1.59%)d 0.01(0.71%)f 0.01(1.10%)g 0.00(0.11%)	2.81
(0.97958) BD (1) C 24- C 26<br>(49.92%) 0.7065* C 24 s(35.36%)p 1.83(64.57%)d 0.00(0.06%)<br>(50.08%) 0.7077* C 26 s(35.29%)p 1.83(64.65%)d 0.00(0.06%)	(0.11335) LV (3)Ce 1<br>s(0.00%)p 1.00(0.26%)d99.99(98.12%)f 6.01(1.57%)g 0.17(0.04%)	4.90
(0.97958) BD (1) C 24- C 26<br>(49.92%) 0.7065* C 24 s(35.36%)p 1.83(64.57%)d 0.00(0.06%)<br>(50.08%) 0.7077* C 26 s(35.29%)p 1.83(64.65%)d 0.00(0.06%)	(0.11283) LV (4)Ce 1<br>s(0.00%)p 1.00(0.21%)d99.99(98.18%)f 7.40(1.57%)g 0.20(0.04%)	4.85
(0.97958) BD (1) C 24- C 26<br>(49.92%) 0.7065* C 24 s(35.36%)p 1.83(64.57%)d 0.00(0.06%)<br>(50.08%) 0.7077* C 26 s(35.29%)p 1.83(64.65%)d 0.00(0.06%)	(0.04563) LV (7)Ce 1<br>s(96.49%)p 0.02(1.59%)d 0.01(0.71%)f 0.01(1.10%)g 0.00(0.11%)	2.41
(0.85263) BD (2) C 24- C 26<br>(49.95%) 0.7067* C 24 s(1.42%)p69.20(98.51%)d 0.04(0.06%)	(0.19853) LV (2)Ce 1<br>s(0.00%)p 0.00(0.00%)d 1.00(89.04%)f 0.12(10.92%)g 0.00(0.04%)	8.16

(50.05%) 0.7075* C 26 s(1.45%)p68.14(98.49%)d 0.04(0.06%)		
(0.85263) BD (2) C 24- C 26 (49.95%) 0.7067* C 24 s(1.42%)p69.20(98.51%)d 0.04(0.06%) (50.05%) 0.7075* C 26 s(1.45%)p68.14(98.49%)d 0.04(0.06%)	(0.11335) LV (3)Ce 1 s(0.00%)p 1.00(0.26%)d99.99(98.12%)f 6.01(1.57%)g 0.17(0.04%)	6.73
(0.85263) BD (2) C 24- C 26 (49.95%) 0.7067* C 24 s(1.42%)p69.20(98.51%)d 0.04(0.06%) (50.05%) 0.7075* C 26 s(1.45%)p68.14(98.49%)d 0.04(0.06%)	(0.11283) LV (4)Ce 1 s(0.00%)p 1.00(0.21%)d99.99(98.18%)f 7.40(1.57%)g 0.20(0.04%)	6.72
(0.85263) BD (2) C 24- C 26 (49.95%) 0.7067* C 24 s(1.42%)p69.20(98.51%)d 0.04(0.06%) (50.05%) 0.7075* C 26 s(1.45%)p68.14(98.49%)d 0.04(0.06%)	(0.04563) LV (7)Ce 1 s(96.49%)p 0.02(1.59%)d 0.01(0.71%)f 0.01(1.10%)g 0.00(0.11%)	3.36
(0.97777) BD (1) C 26- C 34 (50.00%) 0.7071* C 26 s(36.77%)p 1.72(63.17%)d 0.00(0.06%) (50.00%) 0.7071* C 34 s(36.77%)p 1.72(63.17%)d 0.00(0.06%)	(0.11335) LV (3)Ce 1 s(0.00%)p 1.00(0.26%)d99.99(98.12%)f 6.01(1.57%)g 0.17(0.04%)	11.25
(0.97777) BD (1) C 26- C 34 (50.00%) 0.7071* C 26 s(36.77%)p 1.72(63.17%)d 0.00(0.06%) (50.00%) 0.7071* C 34 s(36.77%)p 1.72(63.17%)d 0.00(0.06%)	(0.04563) LV (7)Ce 1 s(96.49%)p 0.02(1.59%)d 0.01(0.71%)f 0.01(1.10%)g 0.00(0.11%)	2.85
(0.97959) BD (1) C 28- C 30 (50.08%) 0.7077* C 28 s(35.29%)p 1.83(64.64%)d 0.00(0.06%) (49.92%) 0.7066* C 30 s(35.36%)p 1.83(64.58%)d 0.00(0.06%)	(0.11335) LV (3)Ce 1 s(0.00%)p 1.00(0.26%)d99.99(98.12%)f 6.01(1.57%)g 0.17(0.04%)	4.88
(0.97959) BD (1) C 28- C 30 (50.08%) 0.7077* C 28 s(35.29%)p 1.83(64.64%)d 0.00(0.06%) (49.92%) 0.7066* C 30 s(35.36%)p 1.83(64.58%)d 0.00(0.06%)	(0.11283) LV (4)Ce 1 s(0.00%)p 1.00(0.21%)d99.99(98.18%)f 7.40(1.57%)g 0.20(0.04%)	4.85
(0.97959) BD (1) C 28- C 30 (50.08%) 0.7077* C 28 s(35.29%)p 1.83(64.64%)d 0.00(0.06%) (49.92%) 0.7066* C 30 s(35.36%)p 1.83(64.58%)d 0.00(0.06%)	(0.04563) LV (7)Ce 1 s(96.49%)p 0.02(1.59%)d 0.01(0.71%)f 0.01(1.10%)g 0.00(0.11%)	2.42
(0.85266) BD (2) C 28- C 30 (50.06%) 0.7075* C 28 s(1.44%)p68.31(98.50%)d 0.04(0.06%) (49.94%) 0.7067* C 30 s(1.42%)p69.19(98.51%)d 0.04(0.06%)	(0.19853) LV (2)Ce 1 s(0.00%)p 0.00(0.00%)d 1.00(89.04%)f 0.12(10.92%)g 0.00(0.04%)	8.15

(0.85266) BD (2) C 28- C 30<br>(50.06%) 0.7075* C 28 s(1.44%)p68.31(98.50%)d 0.04(0.06%)<br>(49.94%) 0.7067* C 30 s(1.42%)p69.19(98.51%)d 0.04(0.06%)	(0.11335) LV (3)Ce 1<br>s(0.00%)p 1.00(0.26%)d99.99(98.12%)f 6.01(1.57%)g 0.17(0.04%)	6.71
(0.85266) BD (2) C 28- C 30<br>(50.06%) 0.7075* C 28 s(1.44%)p68.31(98.50%)d 0.04(0.06%)<br>(49.94%) 0.7067* C 30 s(1.42%)p69.19(98.51%)d 0.04(0.06%)	(0.11283) LV (4)Ce 1<br>s(0.00%)p 1.00(0.21%)d99.99(98.18%)f 7.40(1.57%)g 0.20(0.04%)	6.74
(0.85266) BD (2) C 28- C 30<br>(50.06%) 0.7075* C 28 s(1.44%)p68.31(98.50%)d 0.04(0.06%)<br>(49.94%) 0.7067* C 30 s(1.42%)p69.19(98.51%)d 0.04(0.06%)	(0.04563) LV (7)Ce 1<br>s(96.49%)p 0.02(1.59%)d 0.01(0.71%)f 0.01(1.10%)g 0.00(0.11%)	3.40
(0.97717) BD (1) C 30- C 32<br>(50.00%) 0.7071* C 30 s(36.77%)p 1.72(63.16%)d 0.00(0.07%)<br>(50.00%) 0.7071* C 32 s(36.77%)p 1.72(63.16%)d 0.00(0.07%)	(0.11283) LV (4)Ce 1<br>s(0.00%)p 1.00(0.21%)d99.99(98.18%)f 7.40(1.57%)g 0.20(0.04%)	11.01
(0.97717) BD (1) C 30- C 32<br>(50.00%) 0.7071* C 30 s(36.77%)p 1.72(63.16%)d 0.00(0.07%)<br>(50.00%) 0.7071* C 32 s(36.77%)p 1.72(63.16%)d 0.00(0.07%)	(0.04563) LV (7)Ce 1<br>s(96.49%)p 0.02(1.59%)d 0.01(0.71%)f 0.01(1.10%)g 0.00(0.11%)	2.81
(0.97958) BD (1) C 32- C 34<br>(49.92%) 0.7065* C 32 s(35.36%)p 1.83(64.57%)d 0.00(0.06%)<br>(50.08%) 0.7077* C 34 s(35.29%)p 1.83(64.65%)d 0.00(0.06%)	(0.11335) LV (3)Ce 1<br>s(0.00%)p 1.00(0.26%)d99.99(98.12%)f 6.01(1.57%)g 0.17(0.04%)	4.89
(0.97958) BD (1) C 32- C 34<br>(49.92%) 0.7065* C 32 s(35.36%)p 1.83(64.57%)d 0.00(0.06%)<br>(50.08%) 0.7077* C 34 s(35.29%)p 1.83(64.65%)d 0.00(0.06%)	(0.11283) LV (4)Ce 1<br>s(0.00%)p 1.00(0.21%)d99.99(98.18%)f 7.40(1.57%)g 0.20(0.04%)	4.86
(0.85263) BD (2) C 32- C 34<br>(49.95%) 0.7067* C 32 s(1.42%)p69.21(98.51%)d 0.04(0.06%)<br>(50.05%) 0.7075* C 34 s(1.45%)p68.14(98.49%)d 0.04(0.06%)	(0.19853) LV (2)Ce 1<br>s(0.00%)p 0.00(0.00%)d 1.00(89.04%)f 0.12(10.92%)g 0.00(0.04%)	8.16
(0.85263) BD (2) C 32- C 34<br>(49.95%) 0.7067* C 32 s(1.42%)p69.21(98.51%)d 0.04(0.06%)<br>(50.05%) 0.7075* C 34 s(1.45%)p68.14(98.49%)d 0.04(0.06%)	(0.11335) LV (3)Ce 1<br>s(0.00%)p 1.00(0.26%)d99.99(98.12%)f 6.01(1.57%)g 0.17(0.04%)	6.71
(0.85263) BD (2) C 32- C 34	(0.11283) LV (4)Ce 1<br>s(0.00%)p 1.00(0.21%)d99.99(98.18%)f 7.40(1.57%)g 0.20(0.04%)	6.73

(49.95%) 0.7067* C 32 s(1.42%)p69.21(98.51%)d 0.04(0.06%)<br>(50.05%) 0.7075* C 34 s(1.45%)p68.14(98.49%)d 0.04(0.06%)		
(0.85263) BD (2) C 32- C 34<br>(49.95%) 0.7067* C 32 s(1.42%)p69.21(98.51%)d 0.04(0.06%)<br>(50.05%) 0.7075* C 34 s(1.45%)p68.14(98.49%)d 0.04(0.06%)	(0.04563) LV (7)Ce 1<br>s(96.49%)p 0.02(1.59%)d 0.01(0.71%)f 0.01(1.10%)g 0.00(0.11%)	3.36

Table S33: NBO analysis of canonical molecular orbitals for **2-Ce-cis.**

MO 73 (occ): orbital energy = -0.19089 a.u.<br>0.993*[31]: LP (1)Ce 1(lp)	(0.99737) LP (1)Ce 1<br>s(0.02%)p 0.03(0.00%)d 8.74(0.15%) f99.99(99.83%)g 0.04(0.00%)
MO 74 (occ): orbital energy = -0.18520 a.u.<br>0.419*[73]: BD (2) C32- C34<br>0.419*[57]: BD (2) C20- C22<br>-0.419*[68]: BD (2) C28- C30<br>-0.419*[63]: BD (2) C24- C26<br>0.323*[77]: LV (2)Ce 1(lv)	(0.85263) BD (2) C 32- C 34<br>(49.95%) 0.7067* C 32 s(1.42%)p69.21(98.51%)d 0.04(0.06%)<br>(50.05%) 0.7075* C 34 s(1.45%)p68.14(98.49%)d 0.04(0.06%)<br>(0.85266) BD (2) C 20- C 22<br>(50.06%) 0.7075* C 20 s(1.44%)p68.31(98.50%)d 0.04(0.06%)<br>(49.94%) 0.7067* C 22 s(1.42%)p69.19(98.51%)d 0.04(0.06%)<br>(0.85266) BD (2) C 28- C 30<br>(50.06%) 0.7075* C 28 s(1.44%)p68.31(98.50%)d 0.04(0.06%)<br>(49.94%) 0.7067* C 30 s(1.42%)p69.19(98.51%)d 0.04(0.06%)<br>(0.85263) BD (2) C 24- C 26<br>(49.95%) 0.7067* C 24 s(1.42%)p69.20(98.51%)d 0.04(0.06%)<br>(50.05%) 0.7075* C 26 s(1.45%)p68.14(98.49%)d 0.04(0.06%)
MO 75 (occ): orbital energy = -0.18505 a.u.<br>0.423*[112]: BD*(2) C20- C22*<br>0.423*[118]: BD*(2) C24- C26*<br>0.423*[127]: BD*(2) C32- C34*<br>0.423*[75]: BD*(2) C28- C30*<br>0.323*[76]: LV (1)Ce 1(lv)	(0.19876) LV (1)Ce 1<br>s(0.00%)p 0.00(0.00%)d 1.00(89.02%)f 0.12(10.94%)g 0.00(0.04%)

*Table S34: Energetics of different spin states optimized for **2-Sm-trans.***

	DFT, ΔH (ΔG), kcal/mol	
	Dispersion	No dispersion
s=5/2	0.0	0.0
s=3/2	41.3 (44.8)	42.5 (42.9)
s=1/2	60.6 (64.6)	65.0 (66.1)

*Table S35: Selected structural parameters for **2-Sm-trans.***

	No dispersion			Dispersion		
	s=5/2	s=3/2	s=1/2	s=5/2	s=3/2	s=1/2
Sm1-C2	2.89	2.89	2.89	2.87	2.87	2.86
Sm1-C4	2.98	2.97	2.98	2.96	2.94	2.94
Sm1-C6	2.91	2.90	2.90	2.89	2.87	2.87
Sm1-C8	2.82	2.81	2.81	2.80	2.79	2.79
Sm1-C10	2.77	2.75	2.76	2.75	2.74	2.74
Sm1-C11	2.69	2.63	2.62	2.65	2.61	2.60
Sm1-C13	2.69	2.63	2.62	2.66	2.62	2.61
Sm1-C15	2.69	2.64	2.63	2.66	2.62	2.61
Sm1-C17	2.69	2.63	2.62	2.66	2.61	2.61
Sm1-C20	2.89	2.89	2.89	2.87	2.87	2.86
Sm1-C22	2.98	2.97	2.99	2.96	2.94	2.94
Sm1-C24	2.91	2.90	2.91	2.89	2.87	2.87
Sm1-C26	2.82	2.81	2.81	2.80	2.79	2.79
Sm1-C28	2.69	2.63	2.62	2.65	2.61	2.60
Sm1-C30	2.69	2.63	2.63	2.66	2.62	2.61
Sm1-C32	2.69	2.64	2.63	2.66	2.62	2.61
Sm1-C34	2.69	2.63	2.62	2.66	2.61	2.61
Sm1-C8 (center of the ring)	1.96	1.88	1.86	1.92	1.86	1.84
Sm1-C9 (center of the ring)	2.10	2.09	2.09	2.07	2.06	2.06

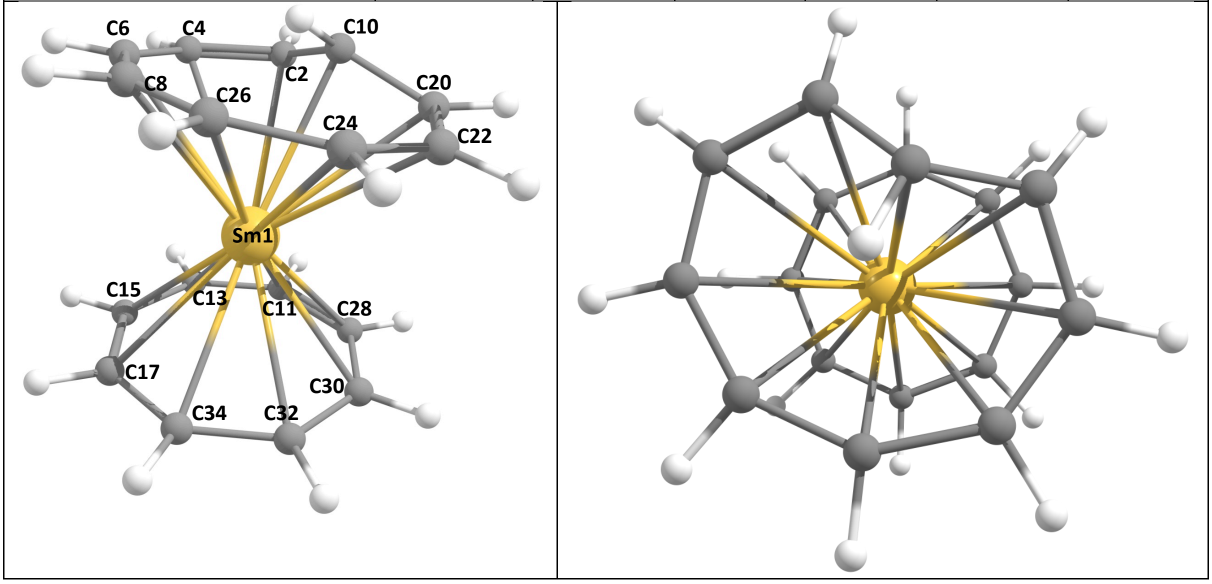

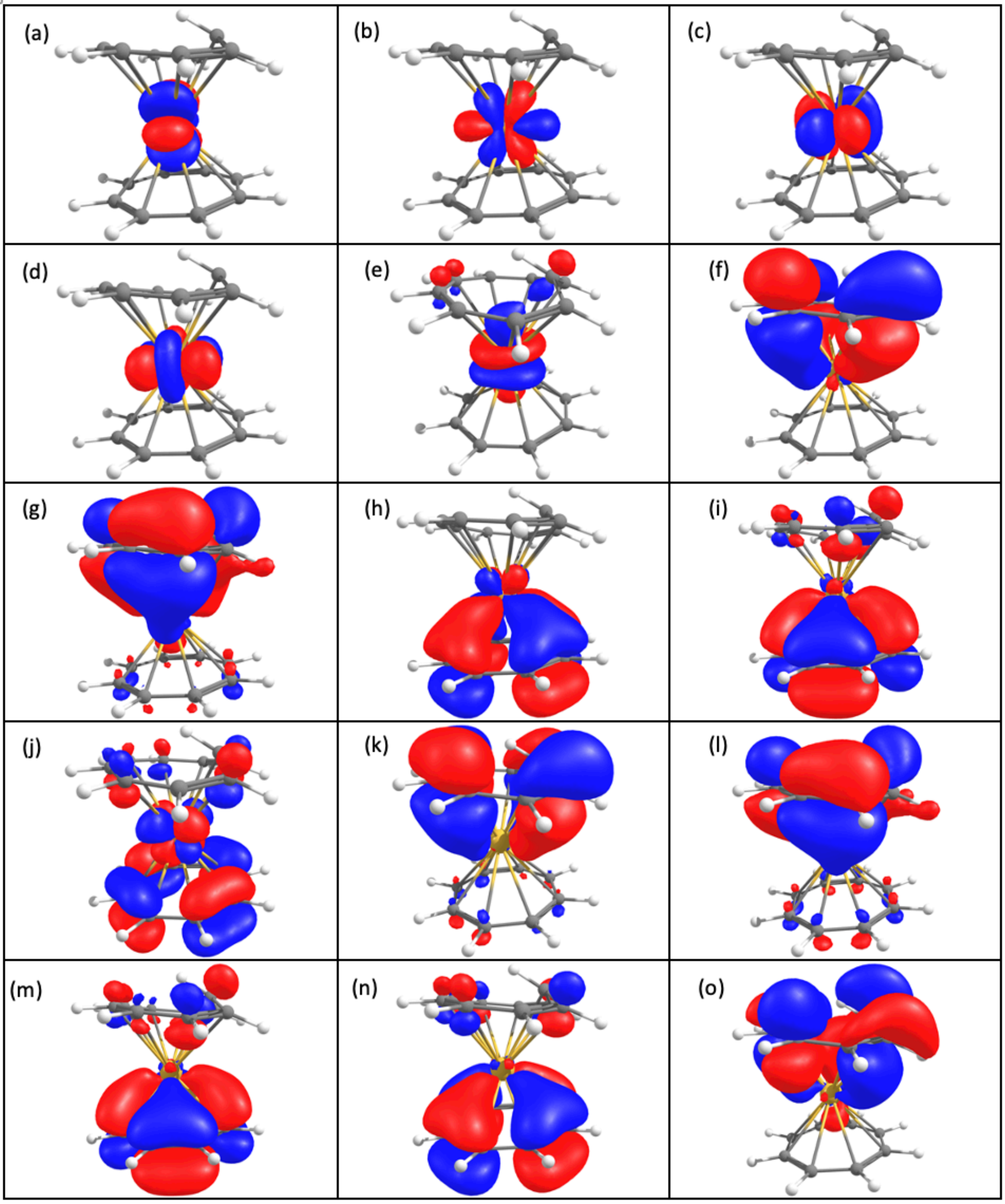

Figure S90: *DFT computed MO's for 1-disp (s=5/2). (a)AMO-HOMO-8 (b)AMO-HOMO-7 (c)AMO-HOMO-6 (d)AMO-HOMO-5 (e)AMO-HOMO-4 (f)AMO-HOMO-3 (g)AMO-HOMO-2 (h)AMO-HOMO-1 (i)AMO-HOMO (j)AMO-LUMO (k)BMO-HOMO-3 (l)BMO-HOMO-2 (m)BMO-HOMO-1 (n)BMO-HOMO (o)BMO-LUMO (p)spin density plot*

*Table S36: Energetics of different spin states optimized for **2-Sm-cis.***

	DFT, ΔH (ΔG), kcal/mol	
	Dispersion	No dispersion
s=5/2	0.0	0.0
s=3/2	44.1 (44.7)	44.5 (45.1)
s=1/2	63.7 (64.8)	64.3 (65.3)

*Table S37: Selected structural parameters for **2-Sm-cis.***

	No dispersion			Dispersion		
	s=5/2	s=3/2	s=1/2	s=5/2	s=3/2	s=1/2
Sm1-C2	2.91	2.90	2.90	2.89	2.88	2.88
Sm1-C4	2.91	2.90	2.90	2.89	2.88	2.88
Sm1-C6	2.91	2.90	2.90	2.89	2.88	2.88
Sm1-C8	2.92	2.90	2.90	2.90	2.88	2.88
Sm1-C10	2.91	2.90	2.90	2.89	2.88	2.88
Sm1-C12	2.68	2.63	2.62	2.65	2.62	2.60
Sm1-C14	2.68	2.63	2.62	2.65	2.62	2.61
Sm1-C16	2.68	2.63	2.62	2.65	2.62	2.60
Sm1-C18	2.68	2.63	2.62	2.65	2.62	2.61
Sm1-C20	2.91	2.90	2.90	2.89	2.88	2.88
Sm1-C22	2.91	2.90	2.90	2.89	2.88	2.88
Sm1-C24	2.92	2.90	2.90	2.90	2.88	2.88
Sm1-C26	2.91	2.90	2.90	2.89	2.88	2.88
Sm1-C28	2.68	2.63	2.62	2.65	2.62	2.60
Sm1-C30	2.68	2.63	2.62	2.65	2.62	2.61
Sm1-C32	2.68	2.63	2.62	2.65	2.62	2.60
Sm1-C34	2.68	2.63	2.62	2.65	2.62	2.61
Sm1-C8 (center of the ring)	1.94	1.88	1.86	1.90	1.86	1.84
Sm1-C9 (center of the ring)	2.06	2.05	2.05	2.04	2.02	2.02

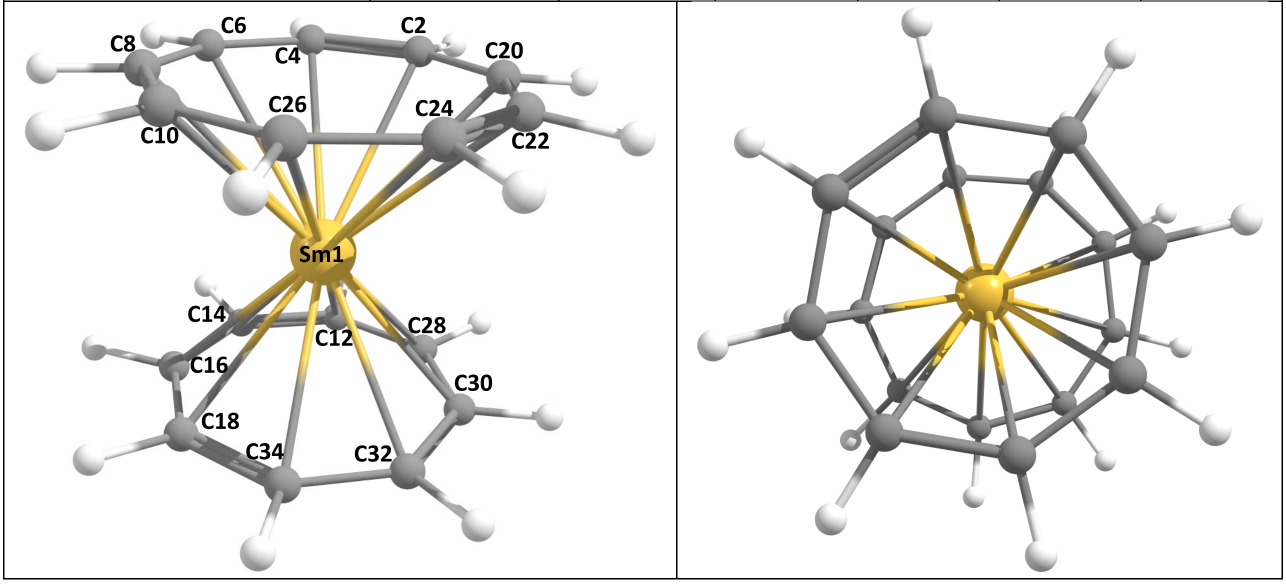

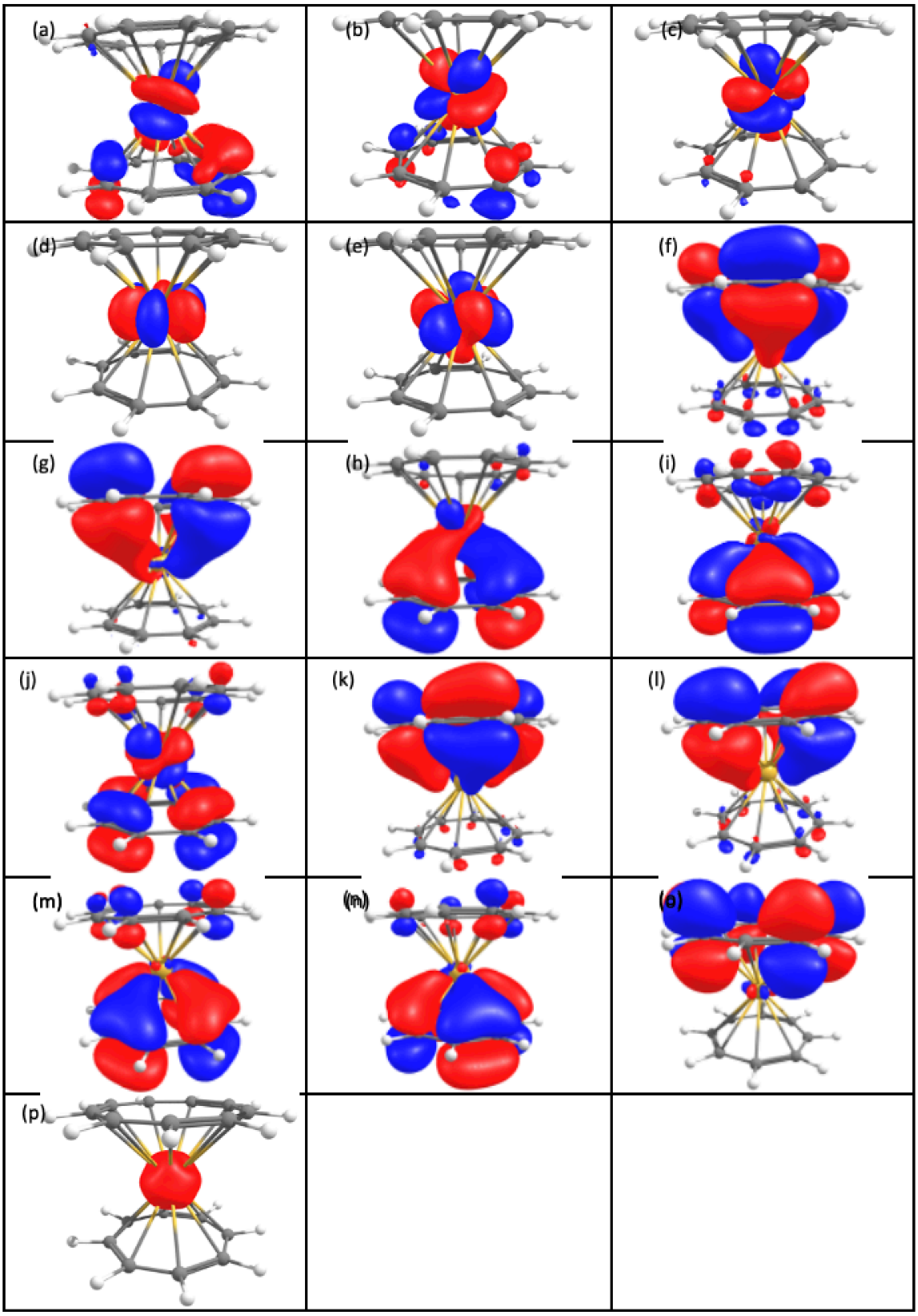

Figure S91: *DFT computed MO's for* ***2-Sm-cis****-disp (s=5/2). (a)AMO-HOMO-8 (b)AMO-HOMO-7 (c)AMO-HOMO-6 (d)AMO-HOMO-5 (e)AMO-HOMO-4 (f)AMO-HOMO-3 (g)AMO-HOMO-2 (h)AMO-HOMO-1 (i)AMO-HOMO (j)AMO-LUMO (k)BMO-HOMO-3 (l)BMO-HOMO-2 (m)BMO-HOMO-1 (n)BMO-HOMO (o)BMO-LUMO (p)spin density plot*

Table S38**: Computed natural charges for **2-Sm-trans.

Atom labels	Natural charges
Sm1	1.32085
C2	-0.29611
C4	-0.28653
C6	-0.33260
C8	-0.36719
C10	-0.31247
C11	-0.37112
C13	-0.36684
C15	-0.36724
C17	-0.37007
C20	-0.29607
C22	-0.28649
C24	-0.33262
C26	-0.36716
C28	-0.37164
C30	-0.36636
C32	-0.36775
C34	-0.36955

*Table S39: Computed Wiberg bond index for **2-Sm-trans.***

Atom labels	Wiberg bond index	Atom labels	Wiberg bond index	Atom labels	Wiberg bond index	Atom labels	Wiberg bond index
Sm1	0.0000	Sm1	0.0000	Sm1	0.0000	Sm1	0.0000
C2	0.0946	C4	0.1056	C6	0.1005	C8	0.1117
Atom labels	Wiberg bond index	Atom labels	Wiberg bond index	Atom labels	Wiberg bond index	Atom labels	Wiberg bond index
Sm1	0.0000	Sm1	0.0000	Sm1	0.0000	Sm1	0.0000
C10	0.1366	C11	0.2333	C13	0.2233	C15	0.2225
Atom labels	Wiberg bond index	Atom labels	Wiberg bond index	Atom labels	Wiberg bond index	Atom labels	Wiberg bond index
Sm1	0.0000	Sm1	0.0000	Sm1	0.0000	Sm1	0.0000
C17	0.2282	C20	0.0946	C22	0.1057	C24	0.1003
Atom labels	Wiberg bond index	Atom labels	Wiberg bond index	Atom labels	Wiberg bond index	Atom labels	Wiberg bond index
Sm1	0.0000	Sm1	0.0000	Sm1	0.0000	Sm1	0.0000
C26	0.1118	C28	0.2328	C30	0.2238	C32	0.2220
Atom labels	Wiberg bond index						
Sm1	0.0000						
C34	0.2287						

*Table S40: DFT computed NBO second order perturbation analysis for **2-Sm-trans.***

Donor NBO	Acceptor NBO	E(2) kcal/mol
(0.50122) LP (1) C 10<br>s(0.56%)p99.99(99.40%)d 0.07(0.04%)	(0.11728) LV (2)Sm 1<br>s(0.08%)p 1.60(0.12%)d99.99(99.75%)f 0.13(0.01%)g 0.42(0.03%)	8.06
(0.50122) LP (1) C 10<br>s(0.56%)p99.99(99.40%)d 0.07(0.04%)	(0.11091) LV (4)Sm 1<br>s(0.04%)p 0.12(0.00%)d99.99(88.33%)f99.99(11.58%)g 1.22(0.05%)	9.22
(0.50122) LP (1) C 10<br>s(0.56%)p99.99(99.40%)d 0.07(0.04%)	(0.05435) LV (5)Sm 1<br>s(94.83%)p 0.01(0.56%)d 0.05(4.33%)f 0.00(0.23%)g 0.00(0.05%)	7.70
(0.50122) LP (1) C 10<br>s(0.56%)p99.99(99.40%)d 0.07(0.04%)	(0.02782) LV (6)Sm 1<br>s(4.73%)p 0.03(0.14%)d19.07(90.18%) f 1.02(4.82%)g 0.03(0.13%)	6.10
(0.83135) BD (2) C 2- C 4<br>(51.09%) 0.7148* C 2<br>s(0.83%)p99.99(99.10%)d 0.09(0.07%)<br>(48.91%) 0.6994* C 4<br>s(0.43%)p99.99(99.52%)d 0.13(0.06%)	(0.18206) LV (1)Sm 1<br>s(0.00%)p 0.00(0.01%)d 1.00(99.82%)f 0.00(0.14%)g 0.00(0.03%)	4.03
(0.83135) BD (2) C 2- C 4<br>(51.09%) 0.7148* C 2<br>s(0.83%)p99.99(99.10%)d 0.09(0.07%)<br>(48.91%) 0.6994* C 4<br>s(0.43%)p99.99(99.52%)d 0.13(0.06%)	(0.11728) LV (2)Sm 1<br>s(0.08%)p 1.60(0.12%)d99.99(99.75%)f 0.13(0.01%)g 0.42(0.03%)	4.02

(0.83135) BD (2) C 2- C 4<br>(51.09%) 0.7148* C 2<br>s(0.83%)p99.99(99.10%)d 0.09(0.07%)<br>(48.91%) 0.6994* C 4<br>s(0.43%)p99.99(99.52%)d 0.13(0.06%)	(0.11458) LV (3)Sm 1<br>s(0.00%)p 1.00(0.04%)d99.99(99.61%)f<br>6.81(0.31%)g 0.87(0.04%)	6.47
(0.98712) BD (1) C 2- C 10<br>(49.42%) 0.7030* C 2 s(35.16%)p<br>1.84(64.78%)d 0.00(0.07%)<br>(50.58%) 0.7112* C 10 s(36.73%)p<br>1.72(63.21%)d 0.00(0.06%)	(0.11728) LV (2)Sm 1<br>s(0.08%)p 1.60(0.12%)d99.99(99.75%)f<br>0.13(0.01%)g 0.42(0.03%)	3.96
(0.98668) BD (1) C 4- C 6<br>(49.51%) 0.7036* C 4 s(35.64%)p<br>1.80(64.29%)d 0.00(0.07%)<br>(50.49%) 0.7106* C 6 s(37.18%)p<br>1.69(62.76%)d 0.00(0.06%)	(0.11458) LV (3)Sm 1<br>s(0.00%)p 1.00(0.04%)d99.99(99.61%)f<br>6.81(0.31%)g 0.87(0.04%)	3.69
(0.98668) BD (1) C 4- C 6<br>(49.51%) 0.7036* C 4 s(35.64%)p<br>1.80(64.29%)d 0.00(0.07%)<br>(50.49%) 0.7106* C 6 s(37.18%)p<br>1.69(62.76%)d 0.00(0.06%)	(0.11728) LV (2)Sm 1<br>s(0.08%)p 1.60(0.12%)d99.99(99.75%)f<br>0.13(0.01%)g 0.42(0.03%)	4.02
(0.85494) BD (2) C 6- C 8<br>(49.54%) 0.7038* C 6<br>s(0.52%)p99.99(99.44%)d 0.07(0.04%)<br>(50.46%) 0.7104* C 8<br>s(0.43%)p99.99(99.53%)d 0.09(0.04%)	(0.11458) LV (3)Sm 1<br>s(0.00%)p 1.00(0.04%)d99.99(99.61%)f<br>6.81(0.31%)g 0.87(0.04%)	4.95
(0.85494) BD (2) C 6- C 8<br>(49.54%) 0.7038* C 6<br>s(0.52%)p99.99(99.44%)d 0.07(0.04%)<br>(50.46%) 0.7104* C 8<br>s(0.43%)p99.99(99.53%)d 0.09(0.04%)	(0.05435) LV (5)Sm 1<br>s(94.83%)p 0.01(0.56%)d 0.05(4.33%)f<br>0.00(0.23%)g 0.00(0.05%)	3.42
(0.98400) BD (1) C 8- C 26<br>(50.00%) 0.7071* C 8 s(37.50%)p<br>1.66(62.44%)d 0.00(0.06%)<br>(50.00%) 0.7071* C 26 s(37.50%)p<br>1.66(62.44%)d 0.00(0.06%)	(0.11728) LV (2)Sm 1<br>s(0.08%)p 1.60(0.12%)d99.99(99.75%)f<br>0.13(0.01%)g 0.42(0.03%)	5.24
(0.98711) BD (1) C 10- C 20<br>(50.58%) 0.7112* C 10 s(36.73%)p<br>1.72(63.21%)d 0.00(0.06%)<br>(49.42%) 0.7030* C 20 s(35.16%)p<br>1.84(64.78%)d 0.00(0.07%)	(0.11728) LV (2)Sm 1<br>s(0.08%)p 1.60(0.12%)d99.99(99.75%)f<br>0.13(0.01%)g 0.42(0.03%)	3.95
(0.83121) BD (2) C 20- C 22<br>(51.09%) 0.7148* C 20<br>s(0.83%)p99.99(99.10%)d 0.09(0.07%)<br>(48.91%) 0.6993* C 22<br>s(0.43%)p99.99(99.52%)d 0.13(0.06%)	(0.18206) LV (1)Sm 1<br>s(0.00%)p 0.00(0.01%)d 1.00(99.82%)f<br>0.00(0.14%)g 0.00(0.03%)	4.02
(0.83121) BD (2) C 20- C 22<br>(51.09%) 0.7148* C 20<br>s(0.83%)p99.99(99.10%)d 0.09(0.07%)<br>(48.91%) 0.6993* C 22<br>s(0.43%)p99.99(99.52%)d 0.13(0.06%)	(0.11728) LV (2)Sm 1<br>s(0.08%)p 1.60(0.12%)d99.99(99.75%)f<br>0.13(0.01%)g 0.42(0.03%)	4.00
(0.83121) BD (2) C 20- C 22	(0.11458) LV (3)Sm 1	6.49

(51.09%) 0.7148* C 20<br>s(0.83%)p99.99(99.10%)d 0.09(0.07%)<br>(48.91%) 0.6993* C 22<br>s(0.43%)p99.99(99.52%)d 0.13(0.06%)	s(0.00%)p 1.00(0.04%)d99.99(99.61%)f<br>6.81(0.31%)g 0.87(0.04%)	
(0.98668) BD (1) C 22- C 24<br>(49.51%) 0.7036* C 22 s(35.64%)p<br>1.80(64.29%)d 0.00(0.07%)<br>(50.49%) 0.7106* C 24 s(37.18%)p<br>1.69(62.76%)d 0.00(0.06%)	(0.11458) LV (3)Sm 1<br>s(0.00%)p 1.00(0.04%)d99.99(99.61%)f<br>6.81(0.31%)g 0.87(0.04%)	3.69
(0.85489) BD (2) C 24- C 26<br>(49.55%) 0.7039* C 24<br>s(0.52%)p99.99(99.44%)d 0.07(0.04%)<br>(50.45%) 0.7103* C 26<br>s(0.43%)p99.99(99.53%)d 0.09(0.04%)	(0.11728) LV (2)Sm 1<br>s(0.08%)p 1.60(0.12%)d99.99(99.75%)f<br>0.13(0.01%)g 0.42(0.03%)	4.04
(0.85489) BD (2) C 24- C 26<br>(49.55%) 0.7039* C 24<br>s(0.52%)p99.99(99.44%)d 0.07(0.04%)<br>(50.45%) 0.7103* C 26<br>s(0.43%)p99.99(99.53%)d 0.09(0.04%)	(0.11458) LV (3)Sm 1<br>s(0.00%)p 1.00(0.04%)d99.99(99.61%)f<br>6.81(0.31%)g 0.87(0.04%)	4.93
(0.85489) BD (2) C 24- C 26<br>(49.55%) 0.7039* C 24<br>s(0.52%)p99.99(99.44%)d 0.07(0.04%)<br>(50.45%) 0.7103* C 26<br>s(0.43%)p99.99(99.53%)d 0.09(0.04%)	(0.05435) LV (5)Sm 1<br>s(94.83%)p 0.01(0.56%)d 0.05(4.33%)f<br>0.00(0.23%)g 0.00(0.05%)	3.42
(0.98010) BD (1) C 11- C 13<br>(50.09%) 0.7078* C 11 s(35.29%)p<br>1.83(64.65%)d 0.00(0.06%)<br>(49.91%) 0.7064* C 13 s(35.41%)p<br>1.82(64.53%)d 0.00(0.06%)	(0.11728) LV (2)Sm 1<br>s(0.08%)p 1.60(0.12%)d99.99(99.75%)f<br>0.13(0.01%)g 0.42(0.03%)	4.50
(0.98010) BD (1) C 11- C 13<br>(50.09%) 0.7078* C 11 s(35.29%)p<br>1.83(64.65%)d 0.00(0.06%)<br>(49.91%) 0.7064* C 13 s(35.41%)p<br>1.82(64.53%)d 0.00(0.06%)	(0.11458) LV (3)Sm 1<br>s(0.00%)p 1.00(0.04%)d99.99(99.61%)f<br>6.81(0.31%)g 0.87(0.04%)	4.86
(0.86593) BD (2) C 11- C 13<br>(49.75%) 0.7054* C 11<br>s(1.11%)p88.74(98.83%)d 0.05(0.06%)<br>(50.25%) 0.7089* C 13<br>s(1.03%)p96.43(98.91%)d 0.06(0.06%)	(0.18206) LV (1)Sm 1<br>s(0.00%)p 0.00(0.01%)d 1.00(99.82%)f<br>0.00(0.14%)g 0.00(0.03%)	5.76
(0.86593) BD (2) C 11- C 13<br>(49.75%) 0.7054* C 11<br>s(1.11%)p88.74(98.83%)d 0.05(0.06%)<br>(50.25%) 0.7089* C 13<br>s(1.03%)p96.43(98.91%)d 0.06(0.06%)	(0.11728) LV (2)Sm 1<br>s(0.08%)p 1.60(0.12%)d99.99(99.75%)f<br>0.13(0.01%)g 0.42(0.03%)	5.91
(0.86593) BD (2) C 11- C 13<br>(49.75%) 0.7054* C 11<br>s(1.11%)p88.74(98.83%)d 0.05(0.06%)<br>(50.25%) 0.7089* C 13<br>s(1.03%)p96.43(98.91%)d 0.06(0.06%)	(0.11458) LV (3)Sm 1<br>s(0.00%)p 1.00(0.04%)d99.99(99.61%)f<br>6.81(0.31%)g 0.87(0.04%)	6.71
(0.86593) BD (2) C 11- C 13	(0.05435) LV (5)Sm 1	4.16

(49.75%) 0.7054* C 11<br>s(1.11%)p88.74(98.83%)d 0.05(0.06%)<br>(50.25%) 0.7089* C 13<br>s(1.03%)p96.43(98.91%)d 0.06(0.06%)	s(94.83%)p 0.01(0.56%)d 0.05(4.33%)f<br>0.00(0.23%)g 0.00(0.05%)	
(0.97903) BD (1) C 11- C 28<br>(50.00%) 0.7071* C 11 s(37.09%)p<br>1.69(62.84%)d 0.00(0.07%)<br>(50.00%) 0.7071* C 28 s(37.09%)p<br>1.69(62.84%)d 0.00(0.07%)	(0.11728) LV (2)Sm 1<br>s(0.08%)p 1.60(0.12%)d99.99(99.75%)f<br>0.13(0.01%)g 0.42(0.03%)	10.92
(0.97880) BD (1) C 13- C 15<br>(50.01%) 0.7072* C 13 s(37.26%)p<br>1.68(62.67%)d 0.00(0.07%)<br>(49.99%) 0.7070* C 15 s(37.23%)p<br>1.68(62.70%)d 0.00(0.07%)	(0.11458) LV (3)Sm 1<br>s(0.00%)p 1.00(0.04%)d99.99(99.61%)f<br>6.81(0.31%)g 0.87(0.04%)	11.05
(0.97880) BD (1) C 13- C 15<br>(50.01%) 0.7072* C 13 s(37.26%)p<br>1.68(62.67%)d 0.00(0.07%)<br>(49.99%) 0.7070* C 15 s(37.23%)p<br>1.68(62.70%)d 0.00(0.07%)	(0.05435) LV (5)Sm 1<br>s(94.83%)p 0.01(0.56%)d 0.05(4.33%)f<br>0.00(0.23%)g 0.00(0.05%)	3.19
(0.98046) BD (1) C 15- C 17<br>(49.96%) 0.7068* C 15 s(35.46%)p<br>1.82(64.48%)d 0.00(0.06%)<br>(50.04%) 0.7074* C 17 s(35.34%)p<br>1.83(64.60%)d 0.00(0.06%)	(0.11728) LV (2)Sm 1<br>s(0.08%)p 1.60(0.12%)d99.99(99.75%)f<br>0.13(0.01%)g 0.42(0.03%)	5.12
(0.98046) BD (1) C 15- C 17<br>(49.96%) 0.7068* C 15 s(35.46%)p<br>1.82(64.48%)d 0.00(0.06%)<br>(50.04%) 0.7074* C 17 s(35.34%)p<br>1.83(64.60%)d 0.00(0.06%)	(0.11458) LV (3)Sm 1<br>s(0.00%)p 1.00(0.04%)d99.99(99.61%)f<br>6.81(0.31%)g 0.87(0.04%)	4.82
(0.86539) BD (2) C 15- C 17<br>(50.23%) 0.7087* C 15<br>s(1.01%)p98.42(98.93%)d 0.06(0.06%)<br>(49.77%) 0.7055* C 17<br>s(1.02%)p96.51(98.91%)d 0.06(0.06%)	(0.18206) LV (1)Sm 1<br>s(0.00%)p 0.00(0.01%)d 1.00(99.82%)f<br>0.00(0.14%)g 0.00(0.03%)	6.61
(0.86539) BD (2) C 15- C 17<br>(50.23%) 0.7087* C 15<br>s(1.01%)p98.42(98.93%)d 0.06(0.06%)<br>(49.77%) 0.7055* C 17<br>s(1.02%)p96.51(98.91%)d 0.06(0.06%)	(0.11728) LV (2)Sm 1<br>s(0.08%)p 1.60(0.12%)d99.99(99.75%)f<br>0.13(0.01%)g 0.42(0.03%)	7.03
(0.86539) BD (2) C 15- C 17<br>(50.23%) 0.7087* C 15<br>s(1.01%)p98.42(98.93%)d 0.06(0.06%)<br>(49.77%) 0.7055* C 17<br>s(1.02%)p96.51(98.91%)d 0.06(0.06%)	(0.11458) LV (3)Sm 1<br>s(0.00%)p 1.00(0.04%)d99.99(99.61%)f<br>6.81(0.31%)g 0.87(0.04%)	7.15
(0.86539) BD (2) C 15- C 17<br>(50.23%) 0.7087* C 15<br>s(1.01%)p98.42(98.93%)d 0.06(0.06%)<br>(49.77%) 0.7055* C 17<br>s(1.02%)p96.51(98.91%)d 0.06(0.06%)	(0.05435) LV (5)Sm 1<br>s(94.83%)p 0.01(0.56%)d 0.05(4.33%)f<br>0.00(0.23%)g 0.00(0.05%)	4.37
(0.97978) BD (1) C 17- C 34	(0.11728) LV (2)Sm 1	11.46

(50.00%) 0.7071* C 17 s(37.11%)p 1.69(62.82%)d 0.00(0.07%) (50.00%) 0.7071* C 34 s(37.11%)p 1.69(62.82%)d 0.00(0.07%)	s(0.08%)p 1.60(0.12%)d99.99(99.75%)f 0.13(0.01%)g 0.42(0.03%)	
(0.98010) BD (1) C 28- C 30 (50.10%) 0.7078* C 28 s(35.29%)p 1.83(64.65%)d 0.00(0.06%) (49.90%) 0.7064* C 30 s(35.41%)p 1.82(64.53%)d 0.00(0.06%)	(0.11728) LV (2)Sm 1 s(0.08%)p 1.60(0.12%)d99.99(99.75%)f 0.13(0.01%)g 0.42(0.03%)	4.48
(0.98010) BD (1) C 28- C 30 (50.10%) 0.7078* C 28 s(35.29%)p 1.83(64.65%)d 0.00(0.06%) (49.90%) 0.7064* C 30 s(35.41%)p 1.82(64.53%)d 0.00(0.06%)	(0.11458) LV (3)Sm 1 s(0.00%)p 1.00(0.04%)d99.99(99.61%)f 6.81(0.31%)g 0.87(0.04%)	4.88
(0.86595) BD (2) C 28- C 30 (49.85%) 0.7061* C 28 s(1.11%)p88.76(98.83%)d 0.05(0.06%) (50.15%) 0.7081* C 30 s(1.03%)p96.45(98.91%)d 0.06(0.06%)	(0.18206) LV (1)Sm 1 s(0.00%)p 0.00(0.01%)d 1.00(99.82%)f 0.00(0.14%)g 0.00(0.03%)	5.77
(0.86595) BD (2) C 28- C 30 (49.85%) 0.7061* C 28 s(1.11%)p88.76(98.83%)d 0.05(0.06%) (50.15%) 0.7081* C 30 s(1.03%)p96.45(98.91%)d 0.06(0.06%)	(0.11728) LV (2)Sm 1 s(0.08%)p 1.60(0.12%)d99.99(99.75%)f 0.13(0.01%)g 0.42(0.03%)	5.88
(0.86595) BD (2) C 28- C 30 (49.85%) 0.7061* C 28 s(1.11%)p88.76(98.83%)d 0.05(0.06%) (50.15%) 0.7081* C 30 s(1.03%)p96.45(98.91%)d 0.06(0.06%)	(0.11458) LV (3)Sm 1 s(0.00%)p 1.00(0.04%)d99.99(99.61%)f 6.81(0.31%)g 0.87(0.04%)	6.73
(0.86595) BD (2) C 28- C 30 (49.85%) 0.7061* C 28 s(1.11%)p88.76(98.83%)d 0.05(0.06%) (50.15%) 0.7081* C 30 s(1.03%)p96.45(98.91%)d 0.06(0.06%)	(0.05435) LV (5)Sm 1 s(94.83%)p 0.01(0.56%)d 0.05(4.33%)f 0.00(0.23%)g 0.00(0.05%)	4.16
(0.97880) BD (1) C 30- C 32 (50.01%) 0.7072* C 30 s(37.26%)p 1.68(62.67%)d 0.00(0.07%) (49.99%) 0.7070* C 32 s(37.23%)p 1.68(62.70%)d 0.00(0.07%)	(0.11458) LV (3)Sm 1 s(0.00%)p 1.00(0.04%)d99.99(99.61%)f 6.81(0.31%)g 0.87(0.04%)	11.05
(0.97880) BD (1) C 30- C 32 (50.01%) 0.7072* C 30 s(37.26%)p 1.68(62.67%)d 0.00(0.07%) (49.99%) 0.7070* C 32 s(37.23%)p 1.68(62.70%)d 0.00(0.07%)	(0.05435) LV (5)Sm 1 s(94.83%)p 0.01(0.56%)d 0.05(4.33%)f 0.00(0.23%)g 0.00(0.05%)	3.19
(0.98046) BD (1) C 32- C 34 (49.96%) 0.7068* C 32 s(35.46%)p 1.82(64.48%)d 0.00(0.06%) (50.04%) 0.7074* C 34 s(35.34%)p 1.83(64.60%)d 0.00(0.06%)	(0.11728) LV (2)Sm 1 s(0.08%)p 1.60(0.12%)d99.99(99.75%)f 0.13(0.01%)g 0.42(0.03%)	5.14
(0.98046) BD (1) C 32- C 34	(0.11458) LV (3)Sm 1	4.80

(49.96%) 0.7068* C 32 s(35.46%)p 1.82(64.48%)d 0.00(0.06%)<br>(50.04%) 0.7074* C 34 s(35.34%)p 1.83(64.60%)d 0.00(0.06%)	s(0.00%)p 1.00(0.04%)d99.99(99.61%)f 6.81(0.31%)g 0.87(0.04%)	
(0.86536) BD (2) C 32- C 34<br>(50.34%) 0.7095* C 32<br>s(1.01%)p98.41(98.93%)d 0.06(0.06%)<br>(49.66%) 0.7047* C 34<br>s(1.02%)p96.51(98.91%)d 0.06(0.06%)	(0.18206) LV (1)Sm 1<br>s(0.00%)p 0.00(0.01%)d 1.00(99.82%)f 0.00(0.14%)g 0.00(0.03%)	6.61
(0.86536) BD (2) C 32- C 34<br>(50.34%) 0.7095* C 32<br>s(1.01%)p98.41(98.93%)d 0.06(0.06%)<br>(49.66%) 0.7047* C 34<br>s(1.02%)p96.51(98.91%)d 0.06(0.06%)	(0.11728) LV (2)Sm 1<br>s(0.08%)p 1.60(0.12%)d99.99(99.75%)f 0.13(0.01%)g 0.42(0.03%)	7.06
(0.86536) BD (2) C 32- C 34<br>(50.34%) 0.7095* C 32<br>s(1.01%)p98.41(98.93%)d 0.06(0.06%)<br>(49.66%) 0.7047* C 34<br>s(1.02%)p96.51(98.91%)d 0.06(0.06%)	(0.11458) LV (3)Sm 1<br>s(0.00%)p 1.00(0.04%)d99.99(99.61%)f 6.81(0.31%)g 0.87(0.04%)	7.12
(0.86536) BD (2) C 32- C 34<br>(50.34%) 0.7095* C 32<br>s(1.01%)p98.41(98.93%)d 0.06(0.06%)<br>(49.66%) 0.7047* C 34<br>s(1.02%)p96.51(98.91%)d 0.06(0.06%)	(0.05435) LV (5)Sm 1<br>s(94.83%)p 0.01(0.56%)d 0.05(4.33%)f 0.00(0.23%)g 0.00(0.05%)	4.37

*Table S 41: NBO analysis of canonical molecular orbitals for **2-Sm-trans**.*

HOMO-3, MO 76 (occ): orbital energy = -0.22675 a.u.<br>0.445*[46]: BD (2) C 6- C 8<br>-0.445*[69]: BD (2) C24- C26<br>-0.384*[40]: BD (2) C 2- C 4<br>0.383*[64]: BD (2) C20- C22<br>0.323*[80]: LV (1)Sm 1(lv)	<u>(0.85494) BD (2) C 6- C 8</u><br>(49.54%) 0.7038* C 6 s(0.52%)p99.99(99.44%)d 0.07(0.04%)<br>(50.46%) 0.7104* C 8 s(0.43%)p99.99(99.53%)d 0.09(0.04%)<br><u>(0.85489) BD (2) C 24- C 26</u><br>(49.55%) 0.7039* C 24 s(0.52%)p99.99(99.44%)d 0.07(0.04%)<br>(50.45%) 0.7103* C 26 s(0.43%)p99.99(99.53%)d 0.09(0.04%)<br><u>(0.83135) BD (2) C 2- C 4</u><br>(51.09%) 0.7148* C 2 s(0.83%)p99.99(99.10%)d 0.09(0.07%)<br>(48.91%) 0.6994* C 4 s(0.43%)p99.99(99.52%)d 0.13(0.06%)<br><u>(0.83121) BD (2) C 20- C 22</u><br>(51.09%) 0.7148* C 20 s(0.83%)p99.99(99.10%)d 0.09(0.07%)<br>(48.91%) 0.6993* C 22 s(0.43%)p99.99(99.52%)d 0.13(0.06%)<br><u>(0.18206) LV (1)Sm 1</u><br>s(0.00%)p 0.00(0.01%)d 1.00(99.82%)f 0.00(0.14%)g 0.00(0.03%)

HOMO-2, MO 77 (occ): orbital energy = -0.22376 a.u.<br>0.412*[37]: LP (1) C10(lp)<br>-0.391*[95]: BD*(2) C 6- C 8*<br>-0.390*[118]: BD*(2) C24- C26*<br>0.339*[89]: BD*(2) C 2- C 4*<br>0.338*[113]: BD*(2) C20- C22*<br>0.305*[83]: LV (4)Sm 1(lv)	(0.50122) LP (1) C 10<br>s(0.56%)p99.99(99.40%)d 0.07(0.04%)<br>(0.22752) BD*(2) C 6- C 8<br>(50.46%) 0.7104* C 6 s(0.52%)p99.99(99.44%)d 0.07(0.04%)<br>(49.54%) -0.7038* C 8 s(0.43%)p99.99(99.53%)d 0.09(0.04%)<br>(0.22754) BD*(2) C 24- C 26<br>(50.45%) 0.7103* C 24 s(0.52%)p99.99(99.44%)d 0.07(0.04%)<br>(49.55%) -0.7039* C 26 s(0.43%)p99.99(99.53%)d 0.09(0.04%)<br>(0.20396) BD*(2) C 2- C 4<br>(48.91%) 0.6994* C 2 s(0.83%)p99.99(99.10%)d 0.09(0.07%)<br>(51.09%) -0.7148* C 4 s(0.43%)p99.99(99.52%)d 0.13(0.06%)<br>(0.20400) BD*(2) C 20- C 22<br>(48.91%) 0.6993* C 20 s(0.83%)p99.99(99.10%)d 0.09(0.07%)<br>(51.09%) -0.7148* C 22 s(0.43%)p99.99(99.52%)d 0.13(0.06%)<br>(0.11091) LV (4)Sm 1<br>s(0.04%)p 0.12(0.00%)d99.99(88.33%)f99.99(11.58%)g 1.22(0.05%)
HOMO-1, MO 78 (occ): orbital energy = -0.18642 a.u.<br>-0.629*[36]: LP (6)Sm 1(lp)<br>-0.361*[102]: BD*(2) C11- C13*<br>-0.361*[122]: BD*(2) C28- C30*<br>-0.359*[127]: BD*(2) C32- C34*<br>-0.359*[108]: BD*(2) C15- C17*	(0.02782) LV (6)Sm 1<br>s(4.73%)p 0.03(0.14%)d19.07(90.18%)f 1.02(4.82%)g 0.03(0.13%)<br>(0.21943) BD*(2) C 28- C 30<br>(50.15%) 0.7081* C 28 s(1.11%)p88.76(98.83%)d 0.05(0.06%)<br>(49.85%) -0.7061* C 30 s(1.03%)p96.45(98.91%)d 0.06(0.06%)<br>(0.21632) BD*(2) C 32- C 34<br>(49.66%) 0.7047* C 32 s(1.01%)p98.41(98.93%)d 0.06(0.06%)<br>(50.34%) -0.7095* C 34 s(1.02%)p96.51(98.91%)d 0.06(0.06%)<br>(0.21632) BD*(2) C 15- C 17<br>(49.77%) 0.7055* C 15 s(1.01%)p98.42(98.93%)d 0.06(0.06%)<br>(50.23%) -0.7087* C 17 s(1.02%)p96.51(98.91%)d 0.06(0.06%)
HOMO, MO 79 (occ): orbital energy = -0.16429 a.u.<br>0.433*[73]: BD (2) C28- C30<br>-0.433*[53]: BD (2) C11- C13<br>0.430*[59]: BD (2) C15- C17<br>-0.430*[78]: BD (2) C32- C34<br>0.250*[80]: LV (1)Sm 1(lv)	(0.86595) BD (2) C 28- C 30<br>(49.85%) 0.7061* C 28<br>s(1.11%)p88.76(98.83%)d 0.05(0.06%)<br>(50.15%) 0.7081* C 30<br>s(1.03%)p96.45(98.91%)d 0.06(0.06%)<br>(0.86593) BD (2) C 11- C 13<br>(49.75%) 0.7054* C 11<br>s(1.11%)p88.74(98.83%)d 0.05(0.06%)

	(50.25%) 0.7089* C 13<br>s(1.03%)p96.43(98.91%)d 0.06(0.06%)<br><u>(0.86539) BD (2) C 15- C 17</u><br>(50.23%) 0.7087* C 15<br>s(1.01%)p98.42(98.93%)d 0.06(0.06%)<br>(49.77%) 0.7055* C 17<br>s(1.02%)p96.51(98.91%)d 0.06(0.06%)<br><u>(0.86536) BD (2) C 32- C 34</u><br>(50.34%) 0.7095* C 32<br>s(1.01%)p98.41(98.93%)d 0.06(0.06%)<br>(49.66%) 0.7047* C 34<br>s(1.02%)p96.51(98.91%)d 0.06(0.06%)<br><u>(0.18206) LV (1)Sm 1</u><br>s(0.00%)p 0.00(0.01%)d 1.00(99.82%)f 0.00(0.14%)g 0.00(0.03%)

Table S42**: Computed natural charges for **2-Sm-trans.

Atom labels	Natural charges
Sm1	1.32301
C2	-0.32685
C4	-0.32465
C6	-0.32069
C8	-0.32108
C10	-0.32467
C12	-0.36640
C14	-0.36370
C16	-0.35721
C18	-0.37023
C20	-0.32519
C22	-0.31942
C24	-0.32130
C26	-0.32547
C28	-0.36566
C30	-0.36258
C32	-0.36296
C34	-0.36422

*Table S43: Computed Wiberg bond index for **2-Sm-trans.***

Atom labels	Wiberg bond index	Atom labels	Wiberg bond index	Atom labels	Wiberg bond index	Atom labels	Wiberg bond index
Sm1	0.0000	Sm1	0.0000	Sm1	0.0000	Sm1	0.0000
C2	0.1064	C4	0.0946	C6	0.1056	C8	0.0946
Atom labels	Wiberg bond index	Atom labels	Wiberg bond index	Atom labels	Wiberg bond index	Atom labels	Wiberg bond index
Sm1	0.0000	Sm1	0.0000	Sm1	0.0000	Sm1	0.0000
C10	0.1031	C12	0.2335	C14	0.2289	C16	0.2371
Atom labels	Wiberg bond index	Atom labels	Wiberg bond index	Atom labels	Wiberg bond index	Atom labels	Wiberg bond index
Sm1	0.0000	Sm1	0.0000	Sm1	0.0000	Sm1	0.0000
C18	0.2279	C20	0.0924	C22	0.1085	C24	0.0933
Atom labels	Wiberg bond index	Atom labels	Wiberg bond index	Atom labels	Wiberg bond index	Atom labels	Wiberg bond index
Sm1	0.0000	Sm1	0.0000	Sm1	0.0000	Sm1	0.0000
C26	0.1022	C28	0.2347	C30	0.2298	C32	0.2317
Atom labels	Wiberg bond index						
Sm1	0.0000						
C34	0.2334						

*Table S44: DFT computed NBO second-order perturbation analysis for **2-Sm-cis.***

Donor NBO	Acceptor NBO	E(2) kcal/mol
(0.44813) LP (6)Sm 1 s(0.00%)p 0.00(0.00%)d 1.00(14.23%)f 6.03(85.77%)g 0.00(0.00%)	102. BD*(2) C 12- C 14	5.74
(0.44813) LP (6)Sm 1 s(0.00%)p 0.00(0.00%)d 1.00(14.23%)f 6.03(85.77%)g 0.00(0.00%)	108. BD*(2) C 16- C 18	4.99
(0.44813) LP (6)Sm 1 s(0.00%)p 0.00(0.00%)d 1.00(14.23%)f 6.03(85.77%)g 0.00(0.00%)	122. BD*(2) C 28- C 30	5.18
(0.44813) LP (6)Sm 1 s(0.00%)p 0.00(0.00%)d 1.00(14.23%)f 6.03(85.77%)g 0.00(0.00%)	127. BD*(2) C 32- C 34	4.43
(0.52209) LP (1) C 2 s(0.58%)p99.99(99.41%)d 0.03(0.02%)	(0.11000) LV (2)Sm 1 s(0.00%)p 1.00(0.12%)d99.99(99.39%)f 3.95(0.46%)g 0.24(0.03%)	6.39
(0.98560) BD (1) C 2- C 4 (50.07%) 0.7076* C 2 s(37.05%)p 1.70(62.89%)d 0.00(0.06%) (49.93%) 0.7066* C 4 s(37.36%)p 1.67(62.58%)d 0.00(0.06%)	(0.11000) LV (2)Sm 1 s(0.00%)p 1.00(0.12%)d99.99(99.39%)f 3.95(0.46%)g 0.24(0.03%)	3.25
(0.98535) BD (1) C 4- C 6	(0.10970) LV (3)Sm 1	4.02

(50.06%) 0.7075* C 4 s(37.12%)p 1.69(62.82%)d 0.00(0.06%)<br>(49.94%) 0.7067* C 6 s(37.18%)p 1.69(62.76%)d 0.00(0.06%)	s(0.00%)p 1.00(0.10%)d99.99(99.68%)f 2.07(0.20%)g 0.28(0.03%)	
(0.84089) BD (2) C 4- C 6<br>(50.79%) 0.7127* C 4 s(0.22%)p99.99(99.74%)d 0.17(0.04%)<br>(49.21%) 0.7015* C 6 s(0.24%)p99.99(99.72%)d 0.16(0.04%)	(0.10970) LV (3)Sm 1<br>s(0.00%)p 1.00(0.10%)d99.99(99.68%)f 2.07(0.20%)g 0.28(0.03%)	7.22
(0.98490) BD (1) C 6- C 8<br>(50.01%) 0.7072* C 6 s(37.33%)p 1.68(62.61%)d 0.00(0.06%)<br>(49.99%) 0.7070* C 8 s(37.24%)p 1.68(62.70%)d 0.00(0.06%)	(0.10970) LV (3)Sm 1<br>s(0.00%)p 1.00(0.10%)d99.99(99.68%)f 2.07(0.20%)g 0.28(0.03%)	3.87
(0.98581) BD (1) C 8- C 10<br>(49.91%) 0.7065* C 8 s(37.21%)p 1.69(62.73%)d 0.00(0.06%)<br>(50.09%) 0.7077* C 10 s(37.18%)p 1.69(62.77%)d 0.00(0.06%)	(0.11000) LV (2)Sm 1<br>s(0.00%)p 1.00(0.12%)d99.99(99.39%)f 3.95(0.46%)g 0.24(0.03%)	3.35
(0.84421) BD (2) C 8- C 10<br>(50.41%) 0.7100* C 8 s(0.22%)p99.99(99.75%)d 0.17(0.04%)<br>(49.59%) 0.7042* C 10 s(0.24%)p99.99(99.73%)d 0.16(0.04%)	(0.11000) LV (2)Sm 1<br>s(0.00%)p 1.00(0.12%)d99.99(99.39%)f 3.95(0.46%)g 0.24(0.03%)	5.78
(0.98557) BD (1) C 10- C 26<br>(50.00%) 0.7071* C 10 s(37.29%)p 1.68(62.65%)d 0.00(0.06%)<br>(50.00%) 0.7071* C 26 s(37.28%)p 1.68(62.66%)d 0.00(0.06%)	(0.11000) LV (2)Sm 1<br>s(0.00%)p 1.00(0.12%)d99.99(99.39%)f 3.95(0.46%)g 0.24(0.03%)	3.85
(0.83984) BD (2) C 20- C 22<br>(50.99%) 0.7141* C 20 s(0.22%)p99.99(99.74%)d 0.16(0.04%)<br>(49.01%) 0.7001* C 22 s(0.25%)p99.99(99.71%)d 0.16(0.04%)	(0.11000) LV (2)Sm 1<br>s(0.00%)p 1.00(0.12%)d99.99(99.39%)f 3.95(0.46%)g 0.24(0.03%)	3.31
(0.83984) BD (2) C 20- C 22<br>(50.99%) 0.7141* C 20 s(0.22%)p99.99(99.74%)d 0.16(0.04%)<br>(49.01%) 0.7001* C 22 s(0.25%)p99.99(99.71%)d 0.16(0.04%)	(0.10970) LV (3)Sm 1<br>s(0.00%)p 1.00(0.10%)d99.99(99.68%)f 2.07(0.20%)g 0.28(0.03%)	3.51
(0.84575) BD (2) C 24- C 26<br>(50.42%) 0.7101* C 24 s(0.22%)p99.99(99.75%)d 0.17(0.04%)<br>(49.58%) 0.7041* C 26 s(0.23%)p99.99(99.73%)d 0.17(0.04%)	(0.10970) LV (3)Sm 1<br>s(0.00%)p 1.00(0.10%)d99.99(99.68%)f 2.07(0.20%)g 0.28(0.03%)	5.25
(0.98068) BD (1) C 12- C 14<br>(50.08%) 0.7077* C 12 s(36.19%)p 1.76(63.75%)d 0.00(0.06%)	(0.10970) LV (3)Sm 1<br>s(0.00%)p 1.00(0.10%)d99.99(99.68%)f 2.07(0.20%)g 0.28(0.03%)	7.41

(49.92%) 0.7065* C 14 s(36.17%)p 1.76(<br>63.77%)d 0.00(0.07%)		
(0.86778) BD (2) C 12- C 14<br>(49.66%) 0.7047* C 12 s(1.05%)p94.28(<br>98.88%)d 0.06(0.07%)<br>(50.34%) 0.7095* C 14 s(1.06%)p93.62(<br>98.88%)d 0.06(0.07%)	(0.18020) LV (1)Sm 1<br>s(0.00%)p 0.00(0.00%)d 1.00(86.88%)f<br>0.15(13.09%)g 0.00(0.03%)	5.67
(0.86778) BD (2) C 12- C 14<br>(49.66%) 0.7047* C 12 s(1.05%)p94.28(<br>98.88%)d 0.06(0.07%)<br>(50.34%) 0.7095* C 14 s(1.06%)p93.62(<br>98.88%)d 0.06(0.07%)	(0.10970) LV (3)Sm 1<br>s(0.00%)p 1.00(0.10%)d99.99(99.68%)f<br>2.07(0.20%)g 0.28(0.03%)	10.29
(0.86778) BD (2) C 12- C 14<br>(49.66%) 0.7047* C 12 s(1.05%)p94.28(<br>98.88%)d 0.06(0.07%)<br>(50.34%) 0.7095* C 14 s(1.06%)p93.62(<br>98.88%)d 0.06(0.07%)	(0.04915) LV (5)Sm 1<br>s(97.76%)p 0.01(0.73%)d 0.01(0.54%)f<br>0.01(0.85%)g 0.00(0.13%)	4.24
(0.97846) BD (1) C 12- C 28<br>(50.00%) 0.7071* C 12 s(36.41%)p 1.74(<br>63.53%)d 0.00(0.06%)<br>(50.00%) 0.7071* C 28 s(36.45%)p 1.74(<br>63.49%)d 0.00(0.06%)	(0.11000) LV (2)Sm 1<br>s(0.00%)p 1.00(0.12%)d99.99(99.39%)f<br>3.95(0.46%)g 0.24(0.03%)	10.03
(0.97779) BD (1) C 14- C 16<br>(49.99%) 0.7070* C 14 s(36.33%)p 1.75(<br>63.60%)d 0.00(0.07%)<br>(50.01%) 0.7072* C 16 s(36.50%)p 1.74(<br>63.43%)d 0.00(0.07%)	(0.10970) LV (3)Sm 1<br>s(0.00%)p 1.00(0.10%)d99.99(99.68%)f<br>2.07(0.20%)g 0.28(0.03%)	10.39
(0.98070) BD (1) C 16- C 18<br>(49.92%) 0.7066* C 16 s(36.22%)p 1.76(<br>63.71%)d 0.00(0.07%)<br>(50.08%) 0.7077* C 18 s(36.08%)p 1.77(<br>63.85%)d 0.00(0.06%)	(0.11000) LV (2)Sm 1<br>s(0.00%)p 1.00(0.12%)d99.99(99.39%)f<br>3.95(0.46%)g 0.24(0.03%)	7.24
(0.86373) BD (2) C 16- C 18<br>(49.18%) 0.7013* C 16 s(1.04%)p94.97(<br>98.89%)d 0.07(0.07%)<br>(50.82%) 0.7129* C 18 s(1.05%)p94.29(<br>98.89%)d 0.06(0.07%)	(0.18020) LV (1)Sm 1<br>s(0.00%)p 0.00(0.00%)d 1.00(86.88%)f<br>0.15(13.09%)g 0.00(0.03%)	6.59
(0.86373) BD (2) C 16- C 18<br>(49.18%) 0.7013* C 16 s(1.04%)p94.97(<br>98.89%)d 0.07(0.07%)<br>(50.82%) 0.7129* C 18 s(1.05%)p94.29(<br>98.89%)d 0.06(0.07%)	(0.11000) LV (2)Sm 1<br>s(0.00%)p 1.00(0.12%)d99.99(99.39%)f<br>3.95(0.46%)g 0.24(0.03%)	9.87
(0.86373) BD (2) C 16- C 18<br>(49.18%) 0.7013* C 16 s(1.04%)p94.97(<br>98.89%)d 0.07(0.07%)<br>(50.82%) 0.7129* C 18 s(1.05%)p94.29(<br>98.89%)d 0.06(0.07%)	(0.10970) LV (3)Sm 1<br>s(0.00%)p 1.00(0.10%)d99.99(99.68%)f<br>2.07(0.20%)g 0.28(0.03%)	3.07

(0.86373) BD (2) C 16- C 18<br>(49.18%) 0.7013* C 16 s(1.04%)p94.97(98.89%)d 0.07(0.07%)<br>(50.82%) 0.7129* C 18 s(1.05%)p94.29(98.89%)d 0.06(0.07%)	(0.04915) LV (5)Sm 1<br>s(97.76%)p 0.01(0.73%)d 0.01(0.54%)f 0.01(0.85%)g 0.00(0.13%)	3.98
(0.97838) BD (1) C 18- C 34<br>(50.00%) 0.7071* C 18 s(36.40%)p 1.75(63.53%)d 0.00(0.06%)<br>(50.00%) 0.7071* C 34 s(36.44%)p 1.74(63.50%)d 0.00(0.06%)	(0.11000) LV (2)Sm 1<br>s(0.00%)p 1.00(0.12%)d99.99(99.39%)f 3.95(0.46%)g 0.24(0.03%)	11.81
(0.98074) BD (1) C 28- C 30<br>(50.10%) 0.7078* C 28 s(36.15%)p 1.76(63.79%)d 0.00(0.06%)<br>(49.90%) 0.7064* C 30 s(36.17%)p 1.76(63.76%)d 0.00(0.07%)	(0.11000) LV (2)Sm 1<br>s(0.00%)p 1.00(0.12%)d99.99(99.39%)f 3.95(0.46%)g 0.24(0.03%)	7.06
(0.86485) BD (2) C 28- C 30<br>(49.82%) 0.7058* C 28 s(1.06%)p93.41(98.87%)d 0.06(0.07%)<br>(50.18%) 0.7084* C 30 s(1.04%)p94.65(98.89%)d 0.06(0.07%)	(0.18020) LV (1)Sm 1<br>s(0.00%)p 0.00(0.00%)d 1.00(86.88%)f 0.15(13.09%)g 0.00(0.03%)	6.34
(0.86485) BD (2) C 28- C 30<br>(49.82%) 0.7058* C 28 s(1.06%)p93.41(98.87%)d 0.06(0.07%)<br>(50.18%) 0.7084* C 30 s(1.04%)p94.65(98.89%)d 0.06(0.07%)	(0.11000) LV (2)Sm 1<br>s(0.00%)p 1.00(0.12%)d99.99(99.39%)f 3.95(0.46%)g 0.24(0.03%)	9.71
(0.86485) BD (2) C 28- C 30<br>(49.82%) 0.7058* C 28 s(1.06%)p93.41(98.87%)d 0.06(0.07%)<br>(50.18%) 0.7084* C 30 s(1.04%)p94.65(98.89%)d 0.06(0.07%)	(0.10970) LV (3)Sm 1<br>s(0.00%)p 1.00(0.10%)d99.99(99.68%)f 2.07(0.20%)g 0.28(0.03%)	3.43
(0.86485) BD (2) C 28- C 30<br>(49.82%) 0.7058* C 28 s(1.06%)p93.41(98.87%)d 0.06(0.07%)<br>(50.18%) 0.7084* C 30 s(1.04%)p94.65(98.89%)d 0.06(0.07%)	(0.04915) LV (5)Sm 1<br>s(97.76%)p 0.01(0.73%)d 0.01(0.54%)f 0.01(0.85%)g 0.00(0.13%)	4.04
(0.97753) BD (1) C 30- C 32<br>(49.97%) 0.7069* C 30 s(36.34%)p 1.75(63.59%)d 0.00(0.07%)<br>(50.03%) 0.7073* C 32 s(36.51%)p 1.74(63.42%)d 0.00(0.07%)	(0.10970) LV (3)Sm 1<br>s(0.00%)p 1.00(0.10%)d99.99(99.68%)f 2.07(0.20%)g 0.28(0.03%)	11.17
(0.98074) BD (1) C 32- C 34<br>(49.94%) 0.7067* C 32 s(36.20%)p 1.76(63.73%)d 0.00(0.07%)<br>(50.06%) 0.7075* C 34 s(36.03%)p 1.77(63.90%)d 0.00(0.06%)	(0.10970) LV (3)Sm 1<br>s(0.00%)p 1.00(0.10%)d99.99(99.68%)f 2.07(0.20%)g 0.28(0.03%)	7.14
(0.86229) BD (2) C 32- C 34	(0.18020) LV (1)Sm 1<br>s(0.00%)p 0.00(0.00%)d 1.00(86.88%)f 0.15(13.09%)g 0.00(0.03%)	7.09

(50.30%) 0.7092* C 32 s(1.05%)p94.51(98.89%)d 0.06(0.07%)<br>(49.70%) 0.7050* C 34 s(1.08%)p91.92(98.86%)d 0.06(0.07%)		
(0.86229) BD (2) C 32- C 34<br>(50.30%) 0.7092* C 32 s(1.05%)p94.51(98.89%)d 0.06(0.07%)<br>(49.70%) 0.7050* C 34 s(1.08%)p91.92(98.86%)d 0.06(0.07%)	(0.11000) LV (2)Sm 1<br>s(0.00%)p 1.00(0.12%)d99.99(99.39%)f 3.95(0.46%)g 0.24(0.03%)	3.37
(0.86229) BD (2) C 32- C 34<br>(50.30%) 0.7092* C 32 s(1.05%)p94.51(98.89%)d 0.06(0.07%)<br>(49.70%) 0.7050* C 34 s(1.08%)p91.92(98.86%)d 0.06(0.07%)	(0.10970) LV (3)Sm 1<br>s(0.00%)p 1.00(0.10%)d99.99(99.68%)f 2.07(0.20%)g 0.28(0.03%)	9.75
(0.86229) BD (2) C 32- C 34<br>(50.30%) 0.7092* C 32 s(1.05%)p94.51(98.89%)d 0.06(0.07%)<br>(49.70%) 0.7050* C 34 s(1.08%)p91.92(98.86%)d 0.06(0.07%)	(0.04915) LV (5)Sm 1<br>s(97.76%)p 0.01(0.73%)d 0.01(0.54%)f 0.01(0.85%)g 0.00(0.13%)	4.16

Table S45**: NBO analysis of canonical molecular orbitals for **2-Sm-cis.

HOMO-3, MO 76 (occ): orbital energy = -0.23005 a.u.<br>-0.454*[69]: BD (2) C24- C26<br>0.452*[48]: BD (2) C 8- C10<br>0.397*[64]: BD (2) C20- C22<br>-0.396*[43]: BD (2) C 4- C 6<br>0.271*[80]: LV (1)Sm 1(lv)	(0.84575) BD (2) C 24- C 26<br>(50.42%) 0.7101* C 24 s(0.22%)p99.99(99.75%)d 0.17(0.04%)<br>(49.58%) 0.7041* C 26 s(0.23%)p99.99(99.73%)d 0.17(0.04%)<br>(0.84421) BD (2) C 8- C 10<br>(50.41%) 0.7100* C 8 s(0.22%)p99.99(99.75%)d 0.17(0.04%)<br>(49.59%) 0.7042* C 10 s(0.24%)p99.99(99.73%)d 0.16(0.04%)<br>(0.83984) BD (2) C 20- C 22<br>(50.99%) 0.7141* C 20 s(0.22%)p99.99(99.74%)d 0.16(0.04%)<br>(49.01%) 0.7001* C 22 s(0.25%)p99.99(99.71%)d 0.16(0.04%)<br>(0.84089) BD (2) C 4- C 6<br>(50.79%) 0.7127* C 4 s(0.22%)p99.99(99.74%)d 0.17(0.04%)<br>(49.21%) 0.7015* C 6 s(0.24%)p99.99(99.72%)d 0.16(0.04%)<br>(0.18020) LV (1)Sm 1<br>s(0.00%)p 0.00(0.00%)d 1.00(86.88%) f 0.15(13.09%)g 0.00(0.03%)
HOMO-2, MO 77 (occ): orbital energy = -0.22804 a.u.<br>0.433*[37]: LP (1) C 2(lp)<br>0.387*[97]: BD*(2) C 8- C10*	(0.52209) LP (1) C 2<br>s(0.58%)p99.99(99.41%)d 0.03(0.02%)<br>(0.21317) BD*(2) C 8- C 10

0.386*[118]: BD*(2) C24- C26*<br>-0.343*[92]: BD*(2) C 4- C 6*<br>-0.343*[113]: BD*(2) C20- C22*<br>0.297*[83]: LV (4)Sm 1(lv)<br>-0.230*[43]: BD (2) C 4- C 6<br>-0.229*[64]: BD (2) C20- C22	(49.59%) 0.7042* C 8 s(0.22%)p99.99(99.75%)d 0.17(0.04%)<br>(50.41%) -0.7100* C 10 s(0.24%)p99.99(99.73%)d 0.16(0.04%)<br><u>(0.21339) BD*(2) C 24- C 26</u><br>(49.58%) 0.7041* C 24 s(0.22%)p99.99(99.75%)d 0.17(0.04%)<br>(50.42%) -0.7101* C 26 s(0.23%)p99.99(99.73%)d 0.17(0.04%)<br><u>(0.21588) BD*(2) C 4- C 6</u><br>(49.21%) 0.7015* C 4 s(0.22%)p99.99(99.74%)d 0.17(0.04%)<br><u>(0.21684) BD*(2) C 20- C 22</u><br>(49.01%) 0.7001* C 20 s(0.22%)p99.99(99.74%)d 0.16(0.04%)<br>(50.99%) -0.7141* C 22 s(0.25%)p99.99(99.71%)d 0.16(0.04%)<br>(50.79%) -0.7127* C 6 s(0.24%)p99.99(99.72%)d 0.16(0.04%)<br><u>(0.10344) LV (4)Sm 1</u><br>s(0.00%)p 0.00(0.01%)d 1.00(86.44%)f 0.16(13.52%)g 0.00(0.03%)<br><u>(0.84089) BD (2) C 4- C 6</u><br>(50.79%) 0.7127* C 4 s(0.22%)p99.99(99.74%)d 0.17(0.04%)<br>(49.21%) 0.7015* C 6 s(0.24%)p99.99(99.72%)d 0.16(0.04%)<br><u>(0.83984) BD (2) C 20- C 22</u><br>(50.99%) 0.7141* C 20 s(0.22%)p99.99(99.74%)d 0.16(0.04%)<br>(49.01%) 0.7001* C 22 s(0.25%)p99.99(99.71%)d 0.16(0.04%)
HOMO-1, MO 78 (occ): orbital energy = -0.18203 a.u.<br>0.659*[36]: LP (6)Sm 1(lp)<br>0.353*[127]: BD*(2) C32- C34*<br>-0.352*[108]: BD*(2) C16- C18*<br>0.352*[122]: BD*(2) C28- C30*<br>0.351*[102]: BD*(2) C12- C14*	<u>(0.44813) LP (6)Sm 1</u><br>s(0.00%)p 0.00(0.00%)d 1.00(14.23%)f 6.03(85.77%)g 0.00(0.00%)<br><u>(0.20409) BD*(2) C 32- C 34</u><br>(49.70%) 0.7050* C 32 s(1.05%)p94.51(98.89%)d 0.06(0.07%)<br>(50.30%) -0.7092* C 34 s(1.08%)p91.92(98.86%)d 0.06(0.07%)<br><u>(0.20421) BD*(2) C 16- C 18</u><br>(50.82%) 0.7129* C 16 s(1.04%)p94.97(98.89%)d 0.07(0.07%)<br>(49.18%) -0.7013* C 18 s(1.05%)p94.29(98.89%)d 0.06(0.07%)<br><u>(0.20428) BD*(2) C 28- C 30</u><br>(50.18%) 0.7084* C 28 s(1.06%)p93.41(98.87%)d 0.06(0.07%)<br>(49.82%) -0.7058* C 30 s(1.04%)p94.65(98.89%)d 0.06(0.07%)<br><u>(0.20441) BD*(2) C 12- C 14</u>

	(50.34%) 0.7095* C 12 s(1.05%)p94.28(98.88%)d 0.06(0.07%)<br>(49.66%) -0.7047* C 14 s(1.06%)p93.62(98.88%)d 0.06(0.07%)
HOMO, MO 79 (occ): orbital energy = -0.17642 a.u.<br>-0.434*[53]: BD (2) C12- C14<br>0.425*[73]: BD (2) C28- C30<br>0.425*[59]: BD (2) C16- C18<br>-0.419*[78]: BD (2) C32- C34<br>0.306*[80]: LV (1)Sm 1(lv)	(0.86778) BD (2) C 12- C 14<br>(49.66%) 0.7047* C 12 s(1.05%)p94.28(98.88%)d 0.06(0.07%)<br>(50.34%) 0.7095* C 14 s(1.06%)p93.62(98.88%)d 0.06(0.07%)<br>(0.86485) BD (2) C 28- C 30<br>(49.82%) 0.7058* C 28 s(1.06%)p93.41(98.87%)d 0.06(0.07%)<br>(50.18%) 0.7084* C 30 s(1.04%)p94.65(98.89%)d 0.06(0.07%)<br>(0.86373) BD (2) C 16- C 18<br>(49.18%) 0.7013* C 16 s(1.04%)p94.97(98.89%)d 0.07(0.07%)<br>(50.82%) 0.7129* C 18 s(1.05%)p94.29(98.89%)d 0.06(0.07%)<br>(0.86229) BD (2) C 32- C 34<br>(50.30%) 0.7092* C 32 s(1.05%)p94.51(98.89%)d 0.06(0.07%)<br>(49.70%) 0.7050* C 34 s(1.08%)p91.92(98.86%)d 0.06(0.07%)<br>(0.18020) LV (1)Sm 1<br>s(0.00%)p 0.00(0.00%)d 1.00(86.88%)f 0.15(13.09%)g 0.00(0.03%)

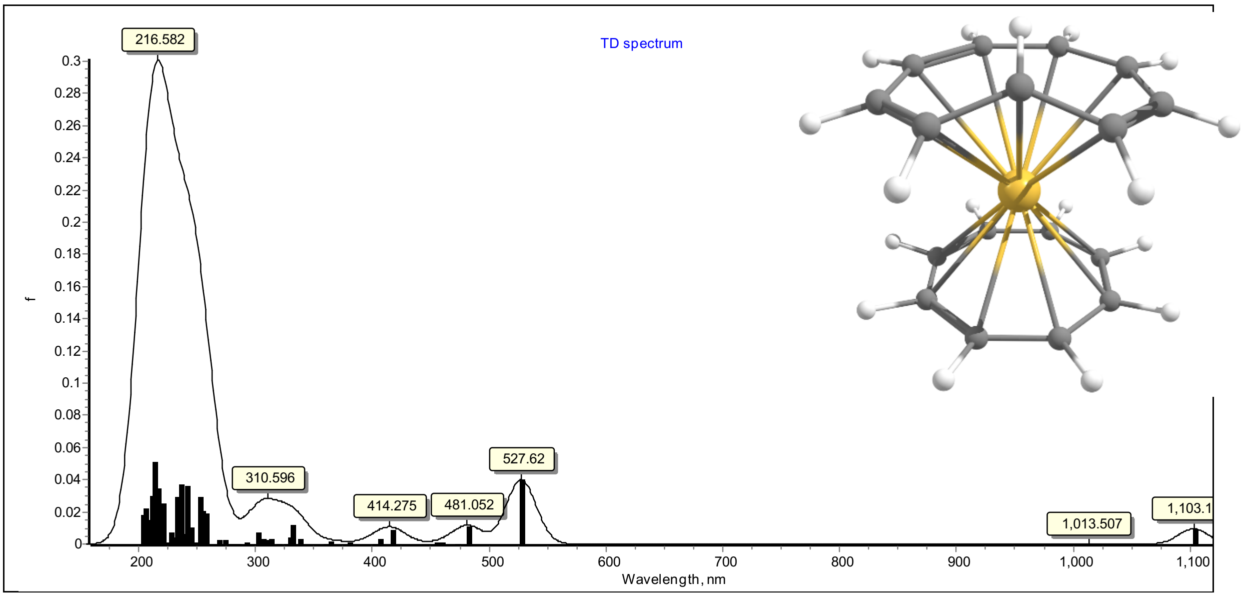

Figure S92**: Computed TD spectrum for **2-Sm-trans.

79A, 74B: HOMO		
Excited State 12: 6.255-?Sym<br>1.1242 eV 1102.88 nm<br>f=0.0094 <S**2>=9.532<br>**78A -> 80A 0.91156**<br>79A -> 81A -0.27684<br>79A -> 82A -0.31260<br>79A <- 82A -0.12342	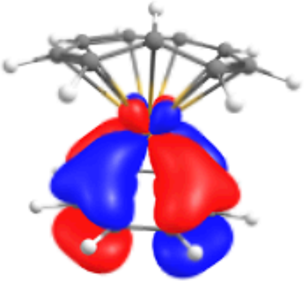<br>78A	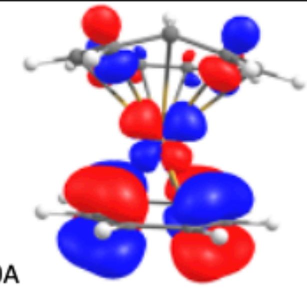<br>80A
Excited State 15: 6.407-?Sym<br>2.3506 eV 527.45 nm<br>f=0.0398 <S**2>=10.014<br>**76A -> 80A 0.79104**<br>78A -> 80A -0.19066<br>79A -> 81A -0.51500<br>79A -> 82A -0.10772<br>72B -> 75B -0.13367<br>73B -> 75B 0.10460	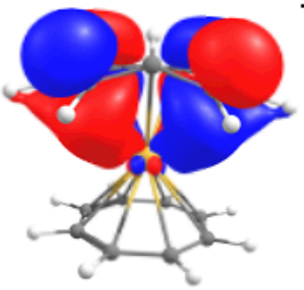<br>76A	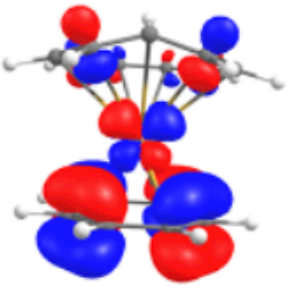<br>80A
Excited State 16: 6.574-?Sym<br>2.5750 eV 481.50 nm<br>f=0.0113 <S**2>=10.554<br>76A -> 80A 0.52647<br>77A -> 81A -0.12639<br>77A -> 82A 0.16114<br>78A -> 80A 0.13041<br>**79A -> 81A 0.64458**<br>79A -> 82A -0.22160<br>72B -> 75B 0.19522<br>73B -> 75B -0.37388	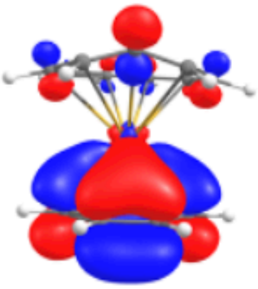<br>79A	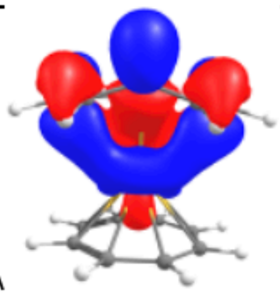<br>81A
Excited State 20: 6.260-?Sym<br>2.9718 eV 417.20 nm<br>f=0.0084 <S**2>=9.548<br>76A -> 80A 0.11676<br>78A -> 80A 0.20975<br>79A -> 81A -0.19456<br>**79A -> 82A 0.76280**<br>79A -> 84A -0.10641<br>73B -> 75B -0.47181<br>74B -> 76B -0.25561	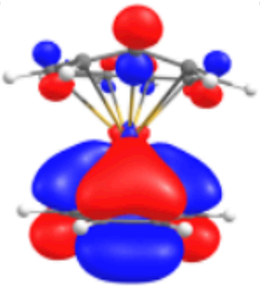<br>79A	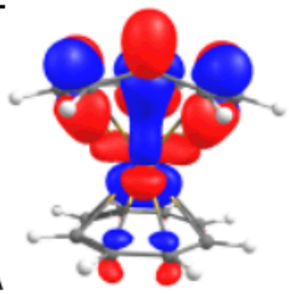<br>82A
Excited State 29: 6.460-?Sym<br>3.7475 eV 330.84 nm<br>f=0.0118 <S**2>=10.182<br>73A -> 82A -0.11253<br>75A -> 81A -0.37725<br>75A -> 84A -0.14879<br>76A -> 83A 0.27591<br>**77A -> 81A 0.53057**<br>**77A -> 82A 0.48288**<br>78A -> 83A -0.11078<br>71B -> 76B -0.36449	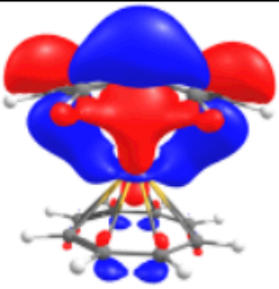<br>77A	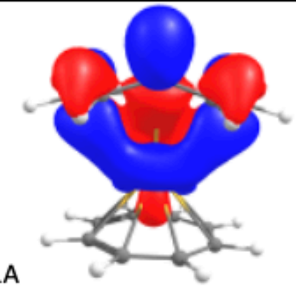<br>81A

72B -> 75B 0.11989<br>74B -> 76B 0.16455		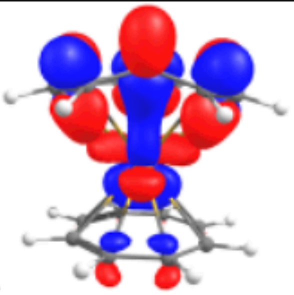<br>82A
Excited State 39: 6.167-?Sym<br>4.1003 eV 302.38 nm<br>f=0.0045 <S**2>=9.258<br>74A -> 81A 0.13868<br>74A -> 82A -0.16755<br>76A -> 81A 0.24562<br>76A -> 82A -0.55227<br>77A -> 83A 0.38545<br>**71B -> 75B 0.56034**<br>72B -> 76B 0.29581	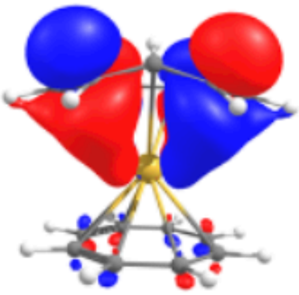<br>71B	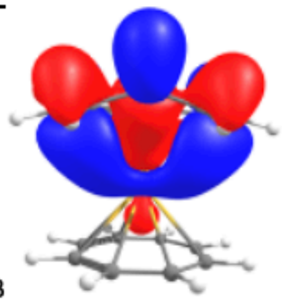<br>75B
Excited State 40: 6.165-?Sym<br>4.1010 eV 302.32 nm<br>f=0.0072 <S**2>=9.252<br>72A -> 81A -0.28104<br>72A -> 82A 0.26941<br>72A -> 84A -0.15225<br>73A -> 82A -0.11594<br>75A -> 81A 0.12878<br>75A -> 82A -0.20150<br>76A -> 83A 0.28849<br>77A -> 81A -0.25645<br>**77A -> 82A 0.53432**<br>71B -> 76B 0.22393<br>72B -> 75B -0.48611	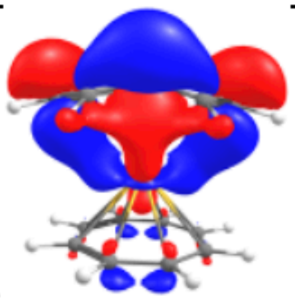<br>77A	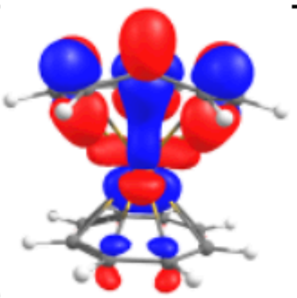<br>82A
Excited State 52: 6.258-?Sym<br>4.8246 eV 256.98 nm<br>f=0.0190 <S**2>=9.541<br>66A -> 80A -0.13806<br>74A -> 83A -0.46745<br>75A -> 81A -0.17572<br>75A -> 82A 0.18132<br>75A -> 84A 0.35394<br>76A -> 83A 0.29120<br>**77A -> 84A 0.56223**<br>79A -> 86A 0.11401<br>71B -> 76B 0.27639	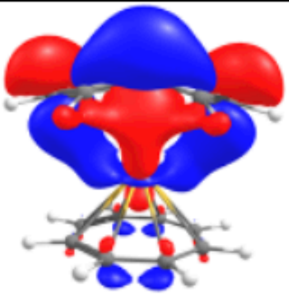<br>77A	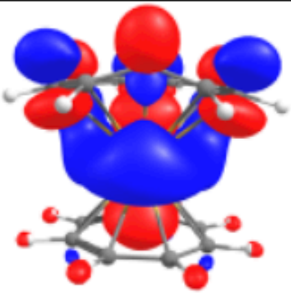<br>84A

<table>
<tr><td>Excited State 54: 6.196-?Sym
4.8691 eV 254.63 nm
f=0.0206 <S**2>=9.346
68A -> 80A 0.15460
73A -> 83A 0.77626
75A -> 83A -0.14989
76A -> 82A 0.10511
76A -> 84A -0.39828
77A -> 83A 0.27427
71B -> 75B -0.10504
72B -> 76B 0.23454</td><td>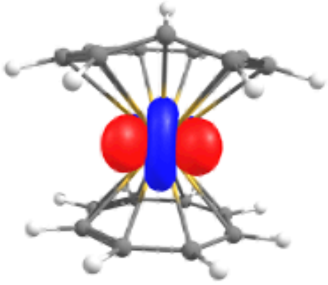
73A</td><td>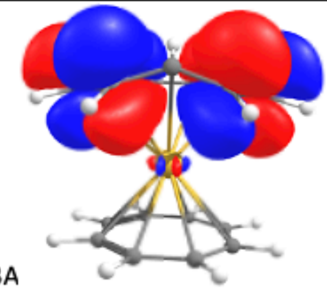
83A</td></tr>
<tr><td>Excited State 56: 6.247-?Sym
4.9174 eV 252.13 nm
f=0.0286 <S**2>=9.507
68A -> 80A -0.22767
72A -> 83A 0.10181
73A -> 83A 0.62289
75A -> 83A 0.13344
76A -> 81A 0.10964
76A -> 82A -0.13490
76A -> 84A 0.53905
77A -> 83A -0.27968
72B -> 76B -0.28954</td><td>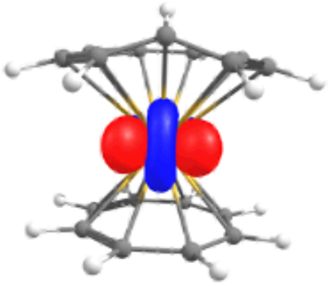
73A</td><td>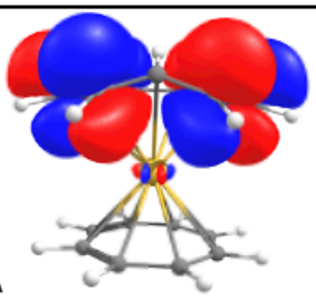
83A</td></tr>
<tr><td>Excited State 57: 6.189-?Sym
4.9294 eV 251.52 nm
f=0.0291 <S**2>=9.327
66A -> 80A 0.19499
74A -> 83A -0.22906
75A -> 81A -0.15243
75A -> 82A 0.14133
75A -> 84A 0.68134
76A -> 83A -0.36165
77A -> 81A -0.12308
77A -> 82A 0.14635
77A -> 84A -0.21633
71B -> 76B -0.37170</td><td>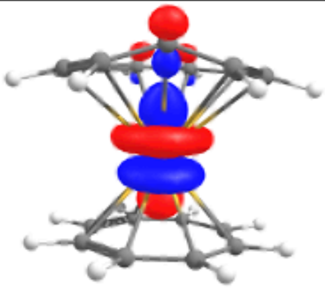
75A</td><td>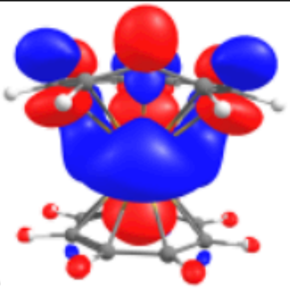
84A</td></tr>
<tr><td>Excited State 59: 6.300-?Sym
5.0847 eV 243.84 nm
f=0.0106 <S**2>=9.673
67A -> 80A -0.21485
68A -> 80A 0.58194
74A -> 81A 0.16413
74A -> 82A -0.16473
74A -> 84A -0.53823
76A -> 84A 0.34480
77A -> 83A 0.16627
71B -> 78B -0.21467
72B -> 76B 0.10277</td><td>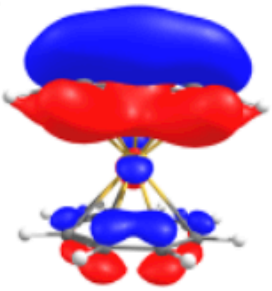
68A</td><td>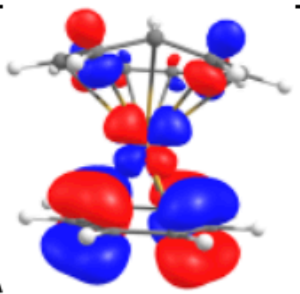
80A</td></tr>
</table>

Excited State 63: 6.435-?Sym<br>5.1402 eV 241.20 nm<br>f=0.0361 <S**2>=10.102<br>65A -> 80A 0.17559<br>66A -> 80A -0.14967<br>71A -> 83A -0.11361<br>73A -> 81A 0.11296<br>73A -> 82A -0.10705<br>73A -> 84A -0.35181<br>75A -> 84A 0.25689<br>76A -> 83A 0.33033<br>77A -> 84A -0.40775<br>79A -> 86A 0.10009<br>71B -> 76B 0.24768<br>72B -> 75B 0.17312<br>**72B -> 78B 0.49979**<br>73B -> 81B -0.11059	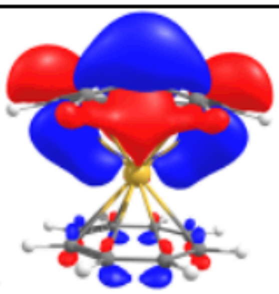<br>72B	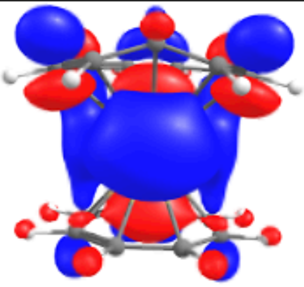<br>78B
Excited State 64: 6.241-?Sym<br>5.2057 eV 238.17 nm<br>f=0.0056 <S**2>=9.487<br>73A -> 81A -0.25764<br>73A -> 82A 0.24877<br>**73A -> 84A 0.80426**<br>74A -> 83A 0.11084<br>75A -> 84A 0.11427<br>77A -> 84A -0.16855<br>72B -> 78B 0.30126	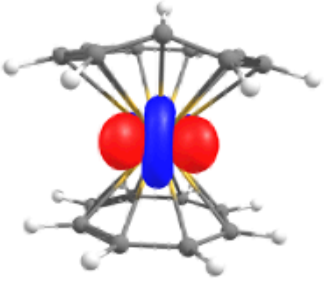<br>73A	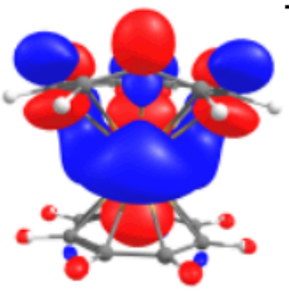<br>84A
Excited State 65: 6.325-?Sym<br>5.2326 eV 236.95 nm<br>f=0.0064 <S**2>=9.750<br>65A -> 80A -0.18169<br>**66A -> 80A 0.86962**<br>72A -> 84A -0.14178<br>77A -> 84A 0.20327<br>71B -> 76B 0.12293<br>72B -> 78B 0.23330	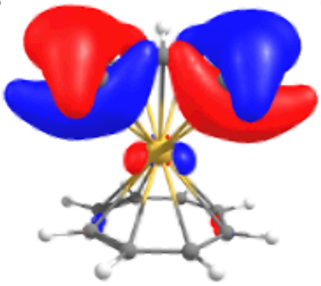<br>66A	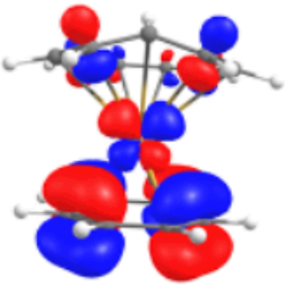<br>80A
Excited State 66: 6.397-?Sym<br>5.2522 eV 236.06 nm<br>f=0.0372 <S**2>=9.981<br>59A -> 80A -0.10695<br>61A -> 80A -0.12509<br>**64A -> 80A 0.65707**<br>68A -> 80A 0.27642<br>74A -> 84A -0.16231<br>76A -> 84A -0.28062<br>77A -> 83A -0.23188<br>71B -> 75B 0.15719<br>71B -> 78B 0.36429<br>72B -> 76B -0.19757<br>74B -> 81B 0.15056	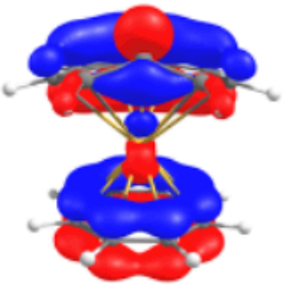<br>64A	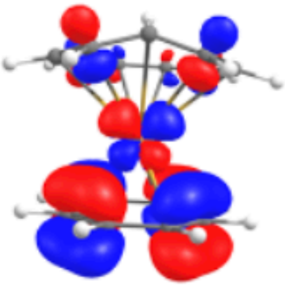<br>80A

Excited State 68: 6.377-?Sym<br>5.3385 eV 232.25 nm<br>f=0.0292 <S**2>=9.917<br>61A -> 80A -0.10605<br>**64A -> 80A 0.64638**<br>68A -> 80A -0.39719<br>77A -> 83A 0.16568<br>78A -> 88A -0.18745<br>78A -> 89A -0.10685<br>71B -> 75B -0.13342<br>71B -> 78B -0.41499<br>72B -> 76B 0.17251<br>74B -> 81B -0.17579	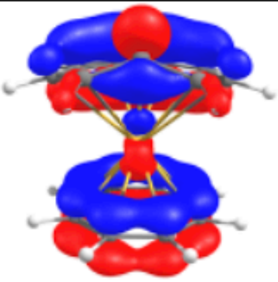 64A	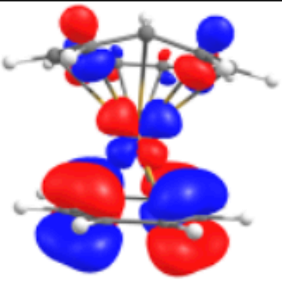 80A
Excited State 70: 6.142-?Sym<br>5.3953 eV 229.80 nm<br>f=0.0042 <S**2>=9.180<br>**71A -> 83A 0.97891**	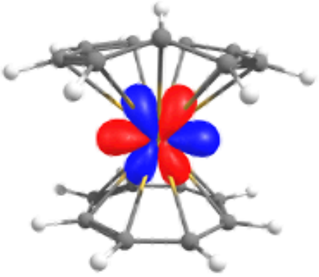 71A	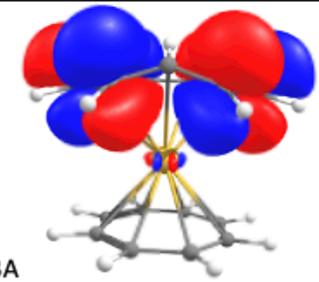 83A
Excited State 72: 6.262-?Sym<br>5.4601 eV 227.07 nm<br>f=0.0074 <S**2>=9.552<br>78A -> 86A 0.48848<br>**78A -> 88A 0.51496**<br>78A -> 89A 0.25462<br>78A -> 92A 0.12499<br>79A -> 87A -0.40063<br>71B -> 78B -0.14086<br>73B -> 79B 0.32867<br>74B -> 80B -0.20562<br>74B -> 81B 0.10247	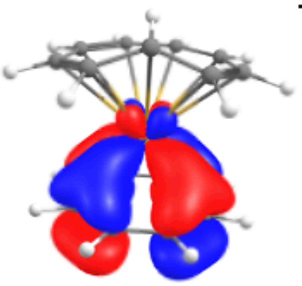 78A	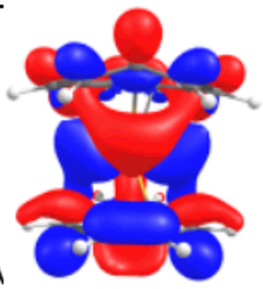 88A
Excited State 80: 6.600-?Sym<br>5.6341 eV 220.06 nm<br>f=0.0255 <S**2>=10.639<br>63A -> 80A 0.24782<br>66A -> 80A 0.13198<br>68A -> 81A 0.15108<br>77A -> 84A -0.18722<br>77A -> 85A -0.13934<br>78A -> 90A 0.36632<br>78A -> 91A -0.16036<br>79A -> 86A -0.12816<br>**79A -> 88A 0.51868**<br>79A -> 89A 0.20985<br>68B -> 75B -0.13817<br>73B -> 81B 0.37245<br>73B -> 84B -0.20203<br>73B -> 91B -0.12245<br>74B -> 90B 0.16536	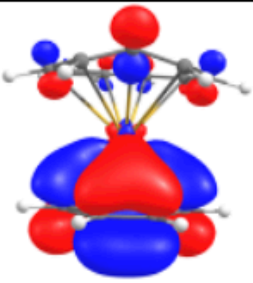 79A	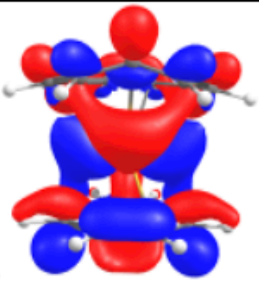 88A

Excited State 83: 6.333-?Sym<br>5.6888 eV 217.94 nm<br>f=0.0182 <S**2>=9.778<br>61A -> 80A -0.18809<br>69A -> 81A -0.12391<br>71A -> 84A -0.10348<br>76A -> 84A 0.24690<br>77A -> 83A 0.11490<br>79A -> 87A 0.13430<br>71B -> 77B -0.47020<br>72B -> 76B 0.12729<br>73B -> 79B -0.10626<br>**74B -> 81B 0.56918**<br>74B -> 82B -0.36860	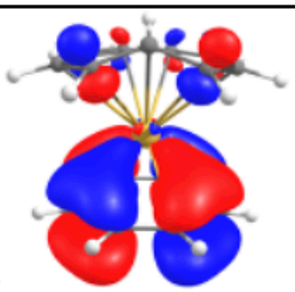<br>74B	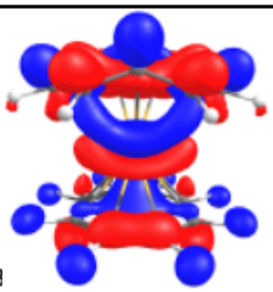<br>81B
Excited State 85: 6.287-?Sym<br>5.7352 eV 216.18 nm<br>f=0.0347 <S**2>=9.631<br>62A -> 80A 0.15318<br>66A -> 80A -0.19793<br>68A -> 81A -0.17144<br>68A -> 82A 0.11191<br>70A -> 82A -0.13679<br>75A -> 84A -0.11734<br>76A -> 83A -0.18273<br>77A -> 84A 0.31358<br>78A -> 87A 0.21185<br>78A -> 90A 0.14042<br>79A -> 86A -0.18781<br>79A -> 88A 0.27720<br>79A -> 89A 0.11669<br>71B -> 76B -0.16284<br>72B -> 75B -0.13836<br>**72B -> 78B 0.48861**<br>73B -> 80B 0.10023<br>73B -> 81B -0.23867<br>73B -> 82B 0.17602<br>74B -> 79B -0.10267<br>74B -> 90B 0.11614	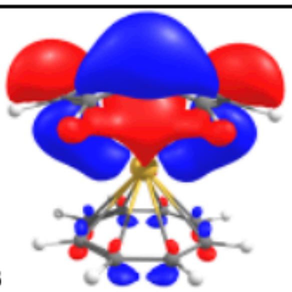<br>72B	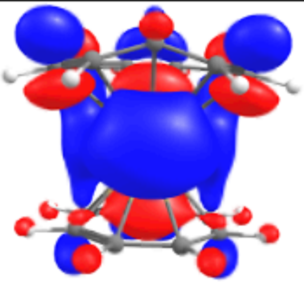<br>78B
Excited State 86: 6.317-?Sym<br>5.7365 eV 216.13 nm<br>f=0.0099 <S**2>=9.725<br>60A -> 80A 0.21398<br>61A -> 80A -0.14152<br>76A -> 84A 0.14917<br>71B -> 78B 0.16889<br>74B -> 81B 0.11287<br>**74B -> 82B 0.78376**<br>74B -> 84B -0.39664	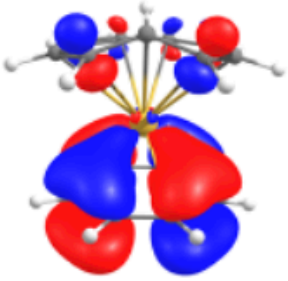<br>74B	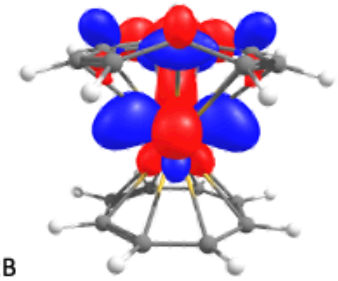<br>82B

Excited State 87: 6.329-?Sym<br>5.7749 eV 214.69 nm<br>f=0.0089 <S**2>=9.763<br>59A -> 80A 0.22360<br>60A -> 80A -0.18872<br>**61A -> 80A 0.64513**<br>64A -> 80A 0.11646<br>76A -> 84A -0.13443<br>79A -> 90A 0.20198<br>71B -> 77B -0.16110<br>71B -> 78B -0.27971<br>74B -> 81B 0.38528<br>74B -> 82B 0.16220<br>74B -> 84B -0.11783	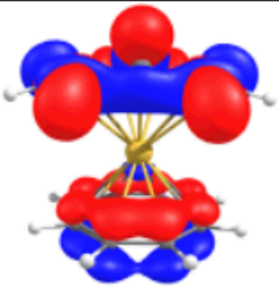<br>61A	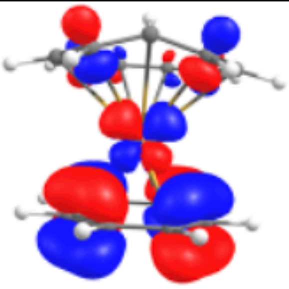<br>80A
Excited State 88: 6.321-?Sym<br>5.8292 eV 212.69 nm<br>f=0.0514 <S**2>=9.739<br>59A -> 80A 0.12389<br>60A -> 80A -0.19070<br>**61A -> 80A 0.47172**<br>64A -> 80A 0.19658<br>76A -> 84A 0.26480<br>77A -> 83A 0.15804<br>78A -> 88A 0.14695<br>79A -> 90A -0.38398<br>79A -> 91A 0.24363<br>71B -> 75B -0.12486<br>**71B -> 78B 0.41846**<br>72B -> 76B 0.16752	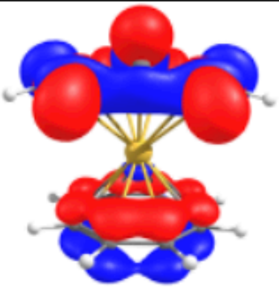<br>61A<br>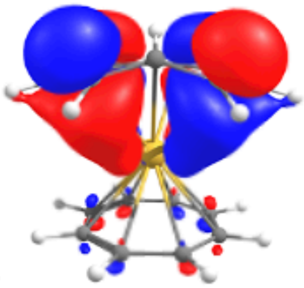<br>71B	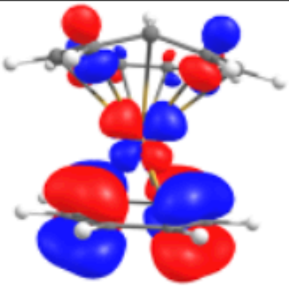<br>80A<br>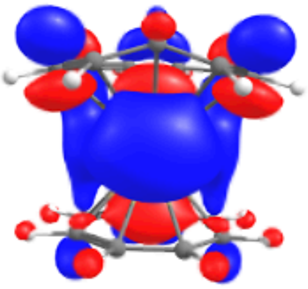<br>78B
Excited State 89: 6.449-?Sym<br>5.8422 eV 212.22 nm<br>f=0.0107 <S**2>=10.146<br>61A -> 80A 0.17481<br>76A -> 84A 0.12524<br>78A -> 88A -0.16532<br>78A -> 94A 0.10344<br>**79A -> 90A 0.69364**<br>79A -> 91A -0.26914<br>79A -> 93A 0.10164<br>71B -> 77B 0.13687<br>71B -> 78B 0.31240<br>74B -> 81B -0.31487<br>74B -> 82B -0.17071	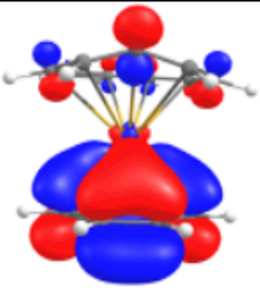<br>79A	90A<br>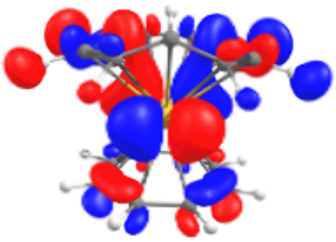

Excited State 90: 6.469-?Sym<br>5.8473 eV 212.04 nm<br>f=0.0082 <S**2>=10.213<br>**62A -> 80A 0.38232**<br>68A -> 81A -0.33425<br>68A -> 82A 0.13438<br>70A -> 82A -0.11674<br>75A -> 85A -0.31390<br>77A -> 84A -0.16024<br>78A -> 91A -0.13340<br>79A -> 89A 0.12651<br>79A -> 92A 0.14813<br>**68B -> 75B 0.39741**<br>70B -> 75B -0.21682<br>71B -> 76B 0.12049<br>72B -> 78B -0.33849<br>72B -> 81B 0.10228<br>72B -> 84B -0.10460<br>73B -> 82B 0.13377	62A<br>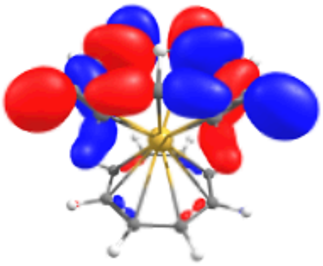	80A 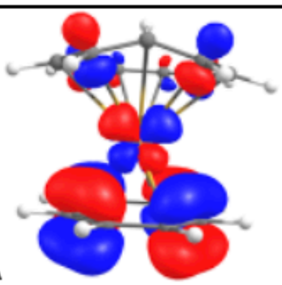
Excited State 91: 6.327-?Sym<br>5.8572 eV 211.68 nm<br>f=0.0073 <S**2>=9.757<br>**62A -> 80A 0.72655**<br>63A -> 80A -0.14402<br>75A -> 85A 0.33300<br>72B -> 78B 0.16722<br>73B -> 81B 0.33301<br>73B -> 82B -0.36038<br>73B -> 84B 0.12857	62A<br>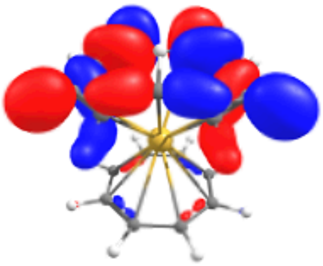	80A 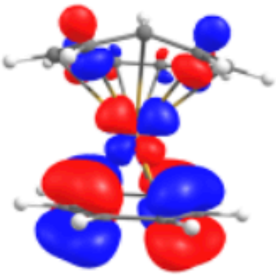
Excited State 93: 6.385-?Sym<br>5.8962 eV 210.28 nm<br>f=0.0296 <S**2>=9.942<br>62A -> 80A -0.44773<br>63A -> 80A 0.10854<br>68A -> 81A -0.19202<br>70A -> 82A -0.13233<br>75A -> 85A -0.11214<br>77A -> 84A 0.10820<br>78A -> 91A -0.18317<br>78A -> 93A -0.10755<br>68B -> 75B 0.15351<br>70B -> 75B -0.11342<br>72B -> 77B 0.12350<br>72B -> 78B 0.22699<br>**73B -> 81B 0.51666**<br>73B -> 82B -0.38002<br>73B -> 84B 0.16440<br>74B -> 85B 0.10591	73B 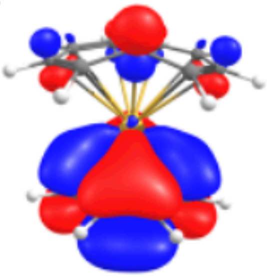	81B 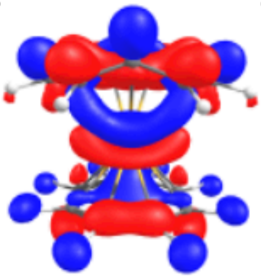

Excited State 96: 6.349-?Sym<br>5.9489 eV 208.42 nm<br>f=0.0151 <S**2>=9.827<br>78A -> 91A 0.39644<br>78A -> 93A 0.19367<br>79A -> 88A -0.18866<br>72B -> 77B 0.11073<br>72B -> 78B 0.15377<br>73B -> 81B 0.50080<br>**73B -> 82B 0.57975**<br>73B -> 84B -0.19913<br>74B -> 90B -0.17551	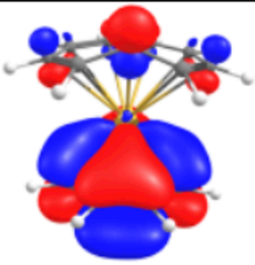<br>73B	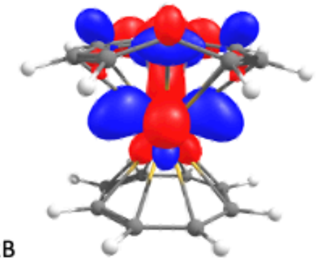<br>82B
Excited State 98: 6.506-?Sym<br>6.0447 eV 205.11 nm<br>f=0.0218 <S**2>=10.332<br>70A -> 81A -0.21352<br>70A -> 82A 0.40742<br>77A -> 84A 0.11188<br>78A -> 87A -0.12325<br>79A -> 86A 0.19134<br>79A -> 88A -0.24673<br>**79A -> 89A 0.68558**<br>79A -> 92A 0.25361	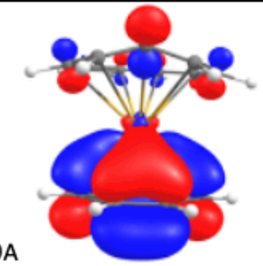<br>79A	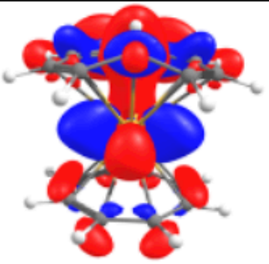<br>89A
Excited State 100: 6.471-?Sym 6.0882 eV 203.65 nm<br>f=0.0184 <S**2>=10.218<br>68A -> 81A -0.18610<br>70A -> 81A -0.39425<br>**70A -> 82A 0.60026**<br>70A -> 84A -0.11603<br>78A -> 87A -0.14533<br>79A -> 88A 0.30610<br>79A -> 89A -0.41250<br>79A -> 94A -0.15408<br>73B -> 80B -0.11038	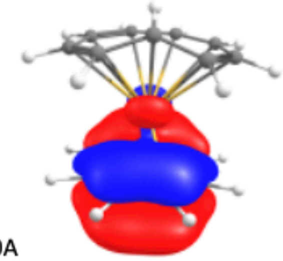<br>70A	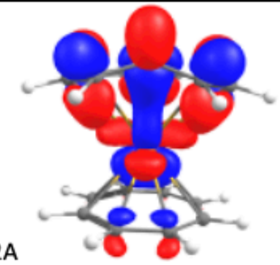<br>82A

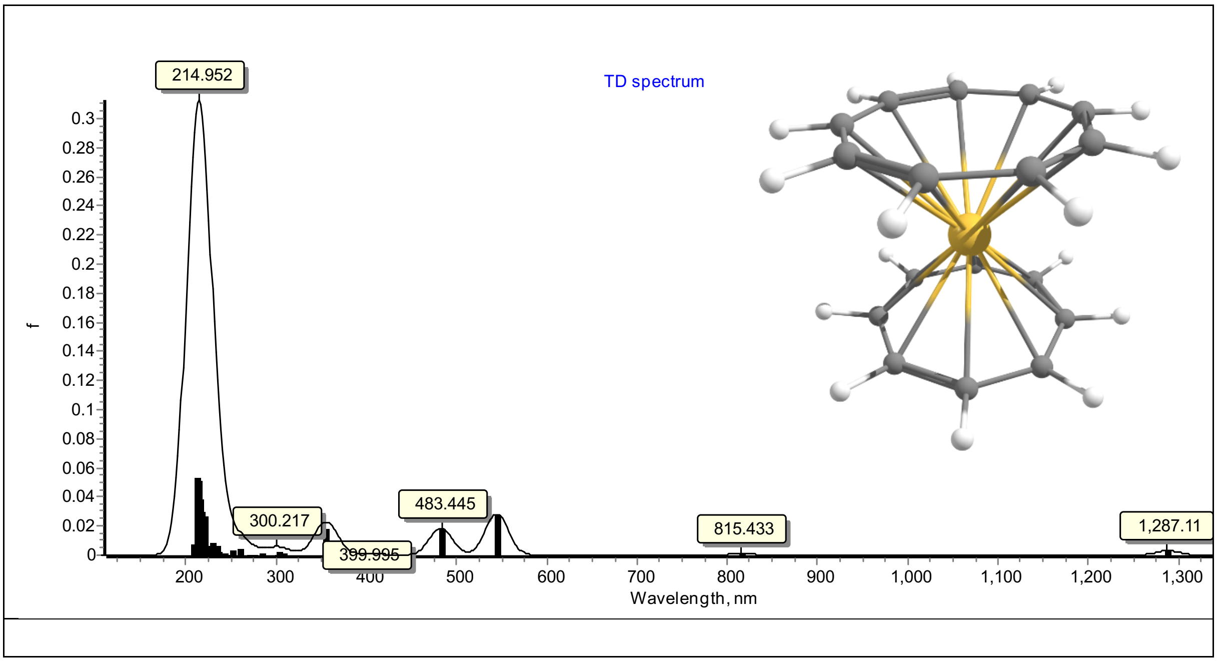

*Figure S93: Computed TD spectrum for **2-Sm-cis.***

79A,74B: HOMO		
Excited State 14: 6.398-?Sym<br>2.2764 eV 544.66 nm<br>f=0.0280 <S**2>=9.984<br>72A -> 81A 0.21663<br>76A -> 81A 0.11243<br>77A -> 80A -0.46410<br>78A -> 80A -0.42772<br>**79A -> 81A 0.73873**<br>78A <- 80A 0.18161	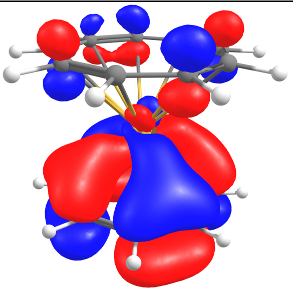<br>79A	81A<br>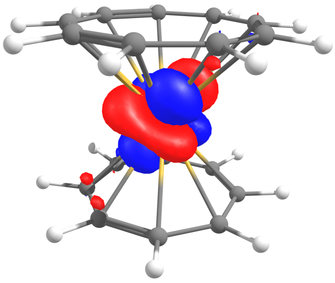
Excited State 16: 6.360-?Sym<br>2.5685 eV 482.72 nm<br>f=0.0182 <S**2>=9.861<br>71A -> 80A -0.18864<br>76A -> 81A 0.16330<br>**77A -> 80A 0.85501**<br>78A -> 80A -0.23750<br>79A -> 81A 0.33430	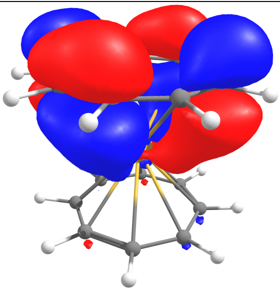<br>77A	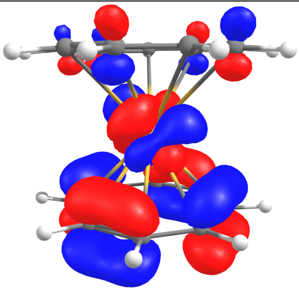<br>80A
Excited State 28: 6.431-?Sym<br>3.5026 eV 353.98 nm<br>f=0.0181 <S**2>=10.090<br>72A -> 81A -0.13570<br>**76A -> 81A 0.96807**<br>79A -> 81A -0.11552	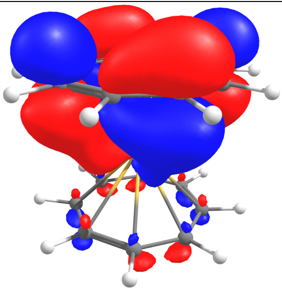<br>76A	81A<br>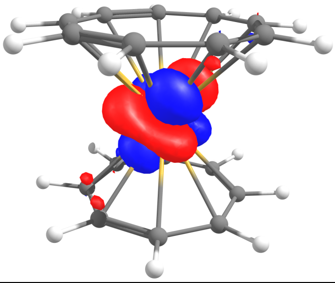
Excited State 49: 6.592-?Sym<br>4.7879 eV 258.95 nm<br>f=0.0044 <S**2>=10.613<br>69A -> 81A -0.16307<br>70A -> 80A 0.34176<br>78A -> 87A 0.28728<br>**79A -> 86A 0.54295**<br>73B -> 80B 0.47509<br>74B -> 79B 0.45812	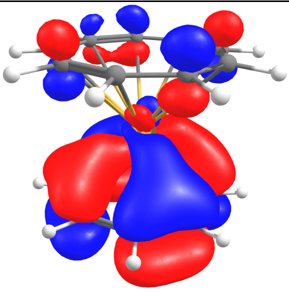<br>79A	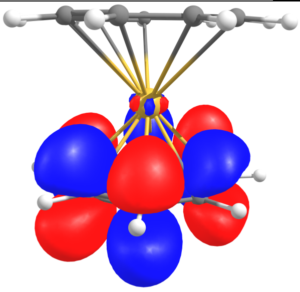<br>86A
Excited State 50: 6.592-?Sym<br>4.7892 eV 258.88 nm<br>f=0.0045 <S**2>=10.613<br>69A -> 80A -0.31312<br>70A -> 81A -0.18044<br>71A -> 80A 0.15642<br>78A -> 86A -0.29043<br>**79A -> 87A 0.54191**<br>73B -> 79B -0.47241<br>74B -> 80B 0.45995	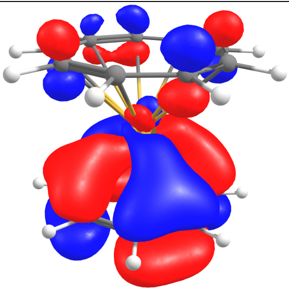<br>79A	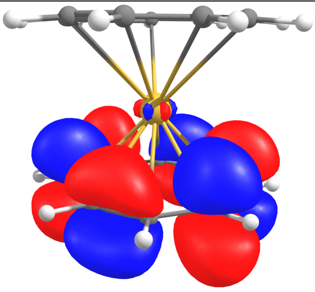<br>87A

Excited State 63: 6.320-?Sym<br>5.3074 eV 233.61 nm<br>f=0.0067 <S**2>=9.736<br>62A -> 80A -0.13540<br>63A -> 80A 0.19812<br>**66A -> 80A 0.90873**<br>71A -> 84A -0.11427<br>76A -> 82A -0.17135<br>77A -> 83A -0.16465<br>72B -> 75B 0.10075	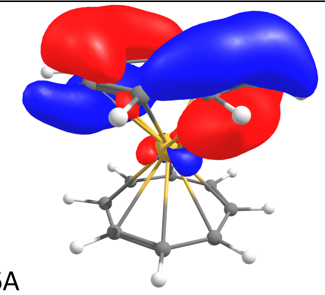<br>66A	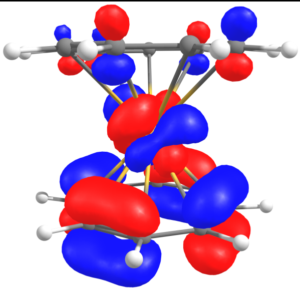<br>80A
Excited State 64: 6.322-?Sym<br>5.3179 eV 233.15 nm<br>f=0.0053 <S**2>=9.741<br>64A -> 80A -0.25526<br>**65A -> 80A 0.89118**<br>72A -> 84A -0.15247<br>76A -> 83A 0.15539<br>77A -> 82A -0.14959<br>78A -> 91A 0.14379	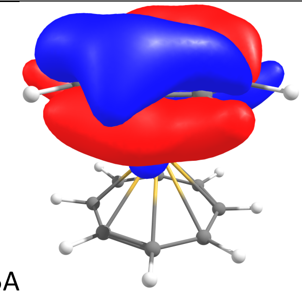<br>65A	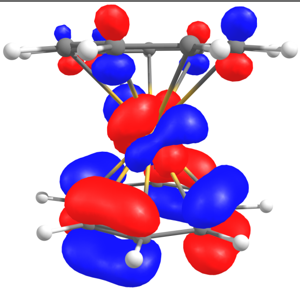<br>80A
Excited State 73: 6.174-?Sym<br>5.4335 eV 228.18 nm<br>f=0.0042 <S**2>=9.279<br>**74A -> 82A 0.86171**<br>75A -> 83A 0.11266<br>78A -> 90A 0.11461<br>78A -> 92A 0.29121<br>74B -> 83B 0.34786<br>74B -> 84B -0.11603	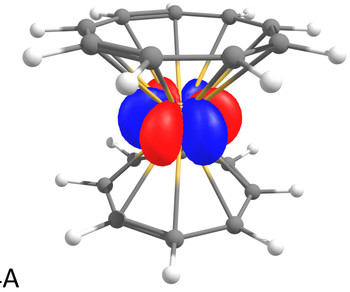<br>74A	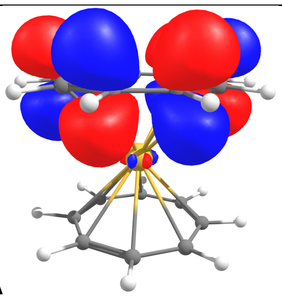<br>82A
Excited State 78: 6.238-?Sym<br>5.5190 eV 224.65 nm<br>f=0.0045 <S**2>=9.479<br>61A -> 80A 0.27873<br>74A -> 82A -0.37799<br>75A -> 83A -0.13455<br>78A -> 90A 0.29890<br>78A -> 92A 0.18750<br>79A -> 91A 0.27013<br>**74B -> 83B 0.68361**<br>74B -> 86B -0.14385	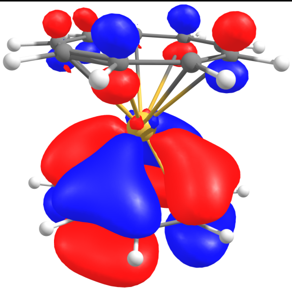<br>74B	83B<br>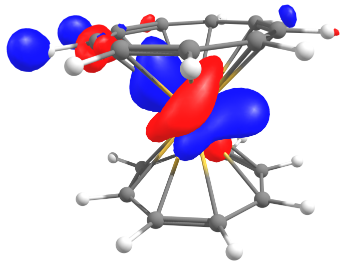
Excited State 83: 6.371-?Sym<br>5.5945 eV 221.62 nm<br>f=0.0060 <S**2>=9.896<br>**61A -> 80A 0.70874**<br>74A -> 82A 0.10114<br>78A -> 90A 0.39765<br>78A -> 92A -0.20075<br>78A -> 94A -0.16530<br>79A -> 91A -0.41177<br>74B -> 83B -0.19892	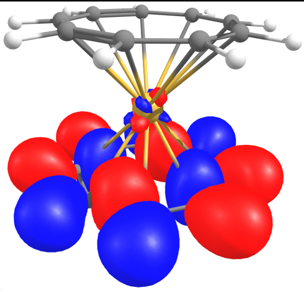<br>61A	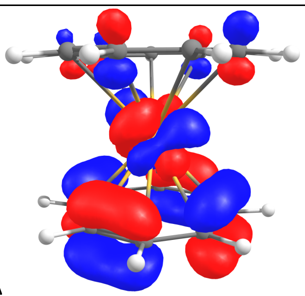<br>80A

Excited State 85: 6.507-?Sym<br>5.6300 eV 220.22 nm<br>f=0.0267 <S**2>=10.334<br>61A -> 80A 0.36482<br>74A -> 82A 0.13124<br>78A -> 92A -0.18152<br>**79A -> 91A 0.75075**<br>73B -> 81B -0.10824<br>73B -> 89B -0.25997<br>74B -> 83B -0.24494<br>74B -> 90B 0.25462	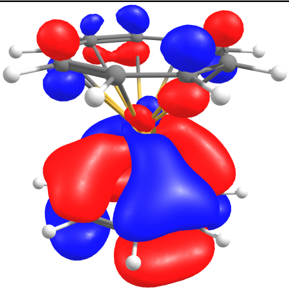<br>79A	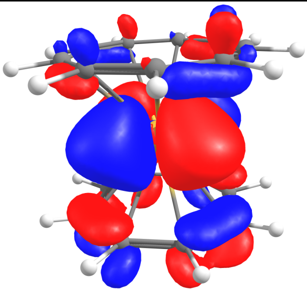<br>91A
Excited State 89: 6.359-?Sym<br>5.7397 eV 216.01 nm<br>f=0.0302 <S**2>=9.860<br>66A -> 80A 0.11094<br>73A -> 83A -0.13819<br>76A -> 82A 0.19943<br>77A -> 83A 0.19366<br>**79A -> 89A 0.81627**<br>79A -> 93A -0.22217<br>71B -> 76B 0.18806<br>72B -> 75B -0.18338<br>74B -> 84B -0.12284	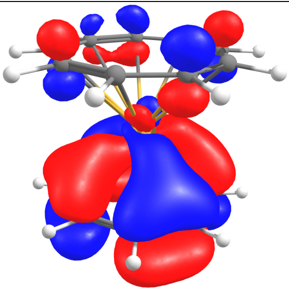<br>79A	89A<br>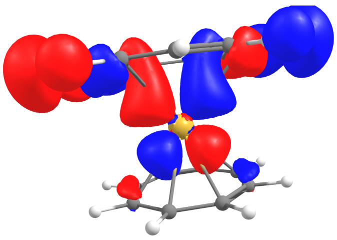
Excited State 90: 6.346-?Sym<br>5.7505 eV 215.61 nm<br>f=0.0388 <S**2>=9.817<br>65A -> 80A -0.13202<br>73A -> 82A 0.11290<br>76A -> 83A 0.22404<br>77A -> 82A -0.22019<br>79A -> 88A 0.17400<br>**79A -> 90A 0.68238**<br>79A -> 92A -0.36894<br>79A -> 94A -0.21440<br>71B -> 75B 0.20839<br>72B -> 76B 0.21423<br>74B -> 79B -0.10421	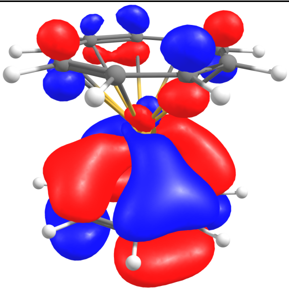<br>79A	90A<br>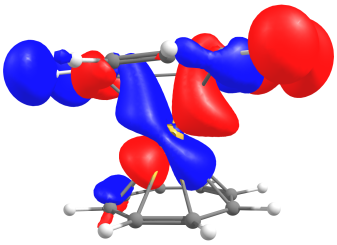
Excited State 91: 6.095-?Sym<br>5.7829 eV 214.40 nm<br>f=0.0048 <S**2>=9.038<br>73A -> 83A 0.18201<br>**74B -> 84B 0.82656**<br>74B -> 86B -0.44009	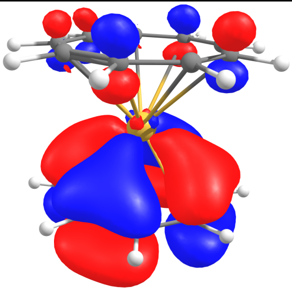<br>74B	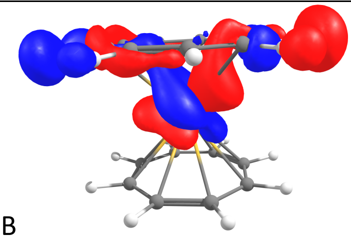<br>84B

Excited State 92: 6.179-?Sym<br>5.8133 eV 213.28 nm<br>f=0.0429 <S**2>=9.294<br>66A -> 80A -0.13130<br>72A -> 82A -0.10484<br>**73A -> 83A 0.71267**<br>76A -> 82A -0.22161<br>77A -> 83A -0.21942<br>78A -> 86A -0.15237<br>79A -> 87A 0.14084<br>79A -> 89A 0.37392<br>79A -> 93A -0.11016<br>71B -> 76B -0.21540<br>72B -> 75B 0.20979<br>73B -> 79B 0.12637	73A<br>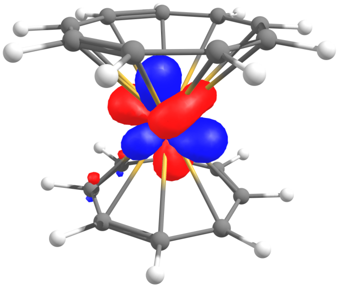	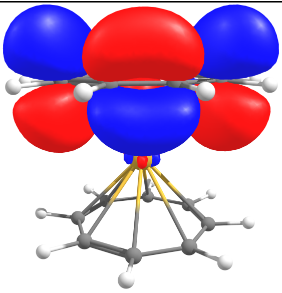<br>83A
Excited State 93: 6.163-?Sym<br>5.8138 eV 213.26 nm<br>f=0.0190 <S**2>=9.246<br>65A -> 80A -0.11137<br>**73A -> 82A 0.84460**<br>76A -> 83A 0.14260<br>77A -> 82A -0.14974<br>78A -> 87A 0.10522<br>79A -> 88A -0.16944<br>79A -> 90A -0.27908<br>79A -> 92A 0.11107<br>71B -> 75B 0.13199<br>72B -> 76B 0.14904	73A<br>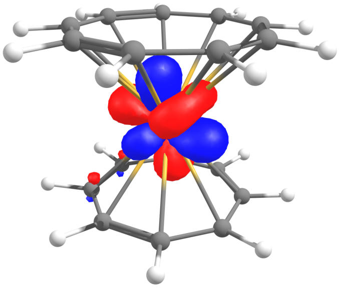	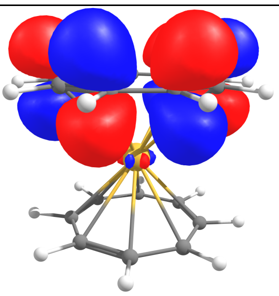<br>82A
Excited State 95: 6.167-?Sym<br>5.8411 eV 212.26 nm<br>f=0.0507 <S**2>=9.260<br>65A -> 80A 0.14727<br>72A -> 83A -0.13244<br>**73A -> 82A 0.48132**<br>76A -> 83A -0.25489<br>77A -> 82A 0.23552<br>78A -> 87A -0.19477<br>79A -> 86A -0.17782<br>79A -> 90A 0.33079<br>79A -> 92A -0.11104<br>79A -> 94A -0.10986<br>71B -> 75B -0.24750<br>72B -> 76B -0.23909<br>73B -> 80B 0.16267<br>73B -> 84B -0.28883<br>73B -> 86B 0.21124<br>74B -> 79B 0.11386<br>74B -> 85B -0.15865<br>74B -> 87B -0.18554	73A<br>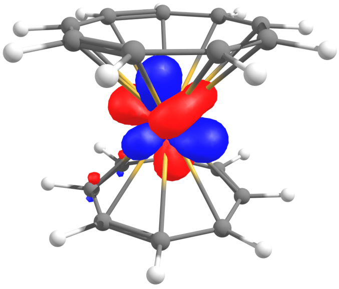	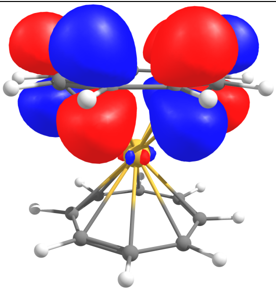<br>82A

Excited State 96: 6.160-?Sym<br>5.8578 eV 211.66 nm<br>f=0.0532 <S**2>=9.238<br>66A -> 80A 0.16964<br>72A -> 82A 0.14740<br>**73A -> 83A 0.64601**<br>76A -> 82A 0.25553<br>77A -> 83A 0.24187<br>78A -> 86A 0.18665<br>79A -> 87A -0.18354<br>79A -> 89A -0.24945<br>71B -> 76B 0.25215<br>72B -> 75B -0.24521<br>73B -> 79B -0.15719<br>74B -> 80B 0.12044<br>74B -> 84B -0.17131	73A<br>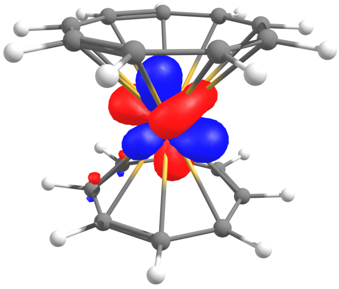	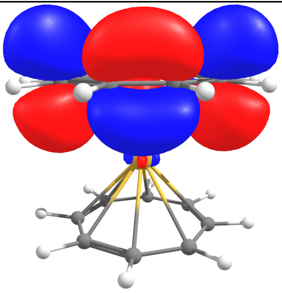<br>83A
Excited State 97: 6.091-?Sym<br>5.8608 eV 211.55 nm<br>f=0.0041 <S**2>=9.026<br>**74B -> 85B 0.76434**<br>74B -> 87B 0.56771<br>74B -> 89B 0.14049	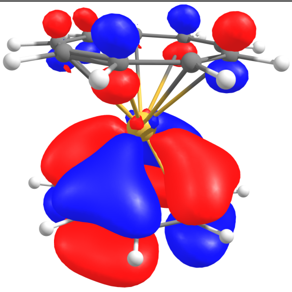<br>74B	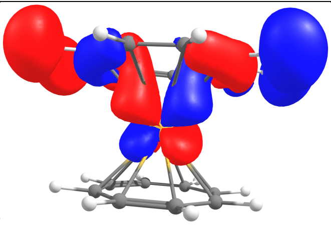<br>85B
Excited State 98: 6.175-?Sym<br>5.8774 eV 210.95 nm<br>f=0.0184 <S**2>=9.283<br>73A -> 82A 0.10812<br>76A -> 83A -0.13190<br>77A -> 82A 0.13347<br>79A -> 90A 0.12873<br>71B -> 75B -0.12913<br>72B -> 76B -0.13009<br>73B -> 83B 0.13559<br>**73B -> 84B 0.80505**<br>73B -> 86B -0.39485	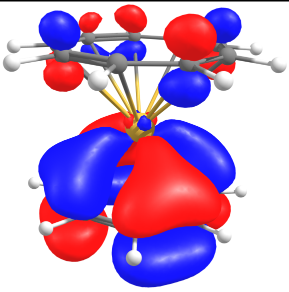<br>73B	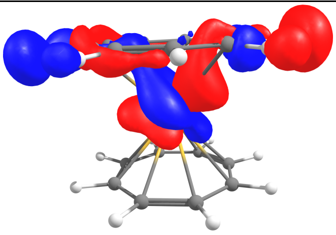<br>84B

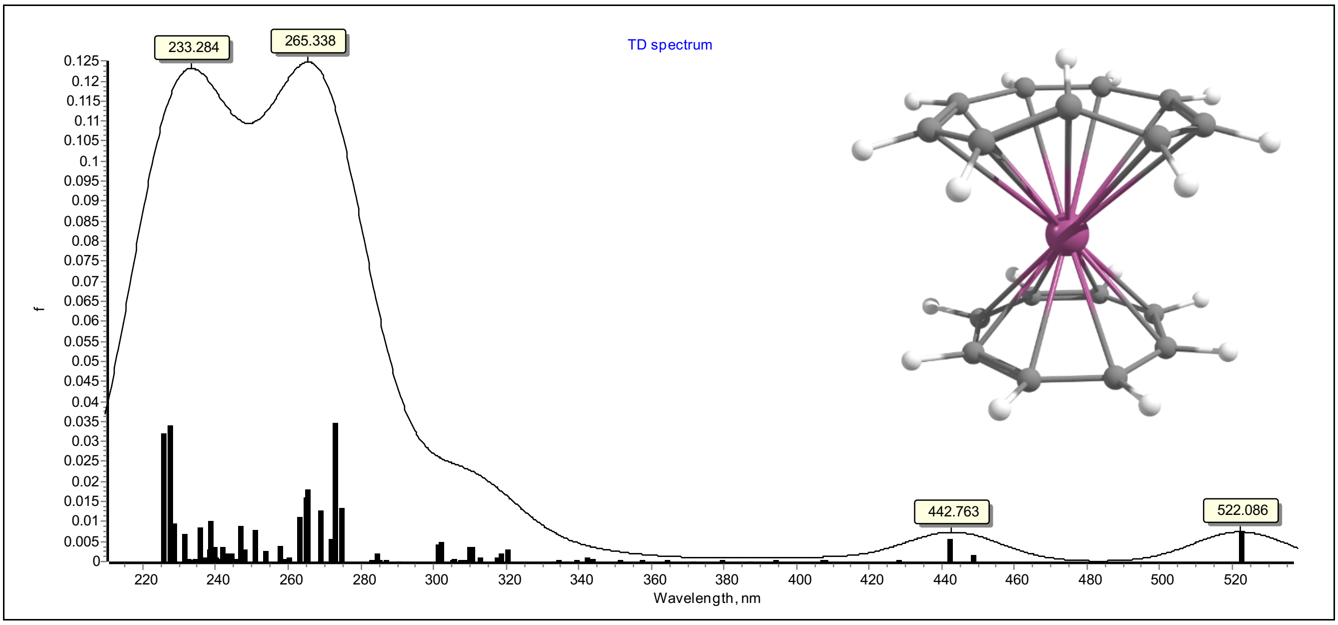

Figure S94*: Computed TD spectrum for* ***2-Ce-trans.***

75A and 74B: HOMO		
Excited State 7: 2.015-?Sym<br>2.3746 eV **522.12** nm<br>f=0.0074 <S**2>=0.765<br>**73A -> 76A 0.85893**<br>73A -> 80A -0.11851<br>73A -> 81A 0.45661	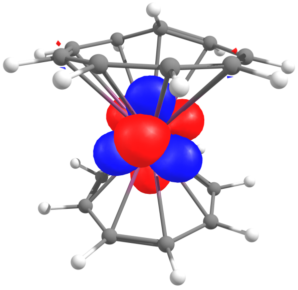<br>73A	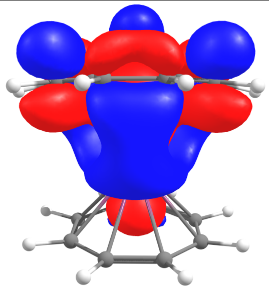<br>76A
Excited State 11: 2.144-?Sym<br>2.8053 eV **441.97** nm<br>f=0.0056 <S**2>=0.899<br>72A -> 76A 0.11227<br>**74A -> 76A 0.67846**<br>74A -> 81A 0.12618<br>**73B -> 75B 0.66309**<br>74B -> 76B -0.15041	74A (73B)<br>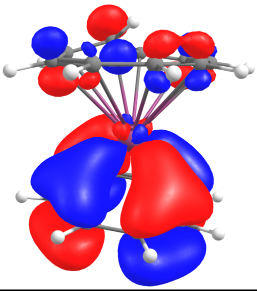	76A (75B)<br>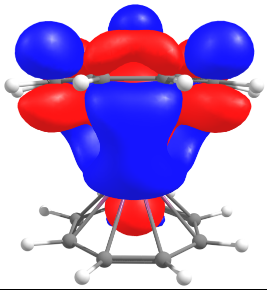
Excited State 43: 2.954-?Sym<br>4.0024 eV **309.77** nm<br>f=0.0035 <S**2>=1.932<br>71A -> 77A -0.40987<br>75A -> 77A -0.29122<br>**75A -> 82A 0.53320**<br>75A -> 83A -0.45897<br>71B -> 76B 0.22155<br>72B -> 75B 0.19255<br>73B -> 82B 0.26234<br>74B -> 83B 0.20979	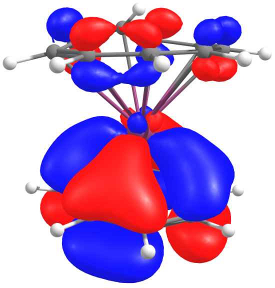<br>75A	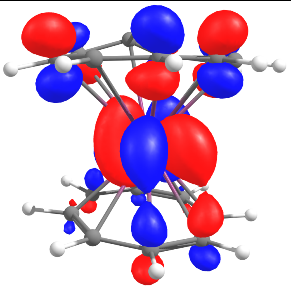<br>82A
Excited State 49: 2.658-?Sym<br>4.1106 eV **301.62** nm<br>f=0.0049 <S**2>=1.516<br>71A -> 77A -0.11900<br>74A -> 81A -0.10164<br>75A -> 82A -0.12304<br>71B -> 76B 0.24754<br>73B -> 82B 0.13278<br>73B -> 84B -0.11077<br>74B -> 76B 0.24246<br>74B -> 79B -0.42606<br>**74B -> 81B 0.72942**<br>74B -> 83B 0.15442	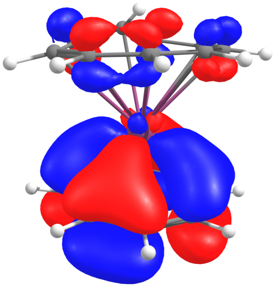<br>74B	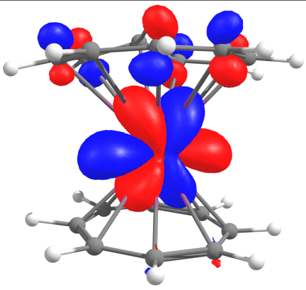<br>81B

Excited State 50: 2.624-?Sym<br>4.1169 eV **301.16** nm<br>f=0.0043 <S**2>=1.472<br>74A -> 82A -0.14367<br>72B -> 76B 0.25692<br>73B -> 76B 0.23005<br>73B -> 79B -0.41885<br>**73B -> 81B** 0.67980<br>73B -> 83B 0.38944	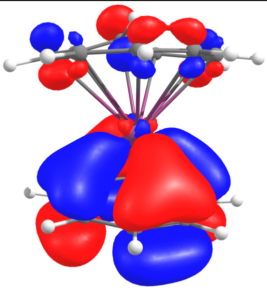<br>73B	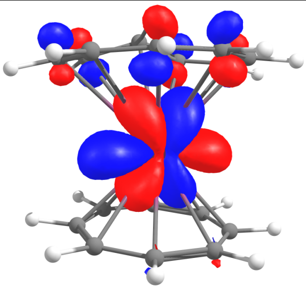<br>81B
Excited State 59: 2.561-?Sym<br>4.5213 eV **274.22** nm<br>f=0.0135 <S**2>=1.389<br>71A -> 77A 0.42315<br>71A -> 79A 0.23642<br>71A -> 83A -0.14003<br>**72A -> 80A** 0.54680<br>71B -> 76B 0.36051<br>72B -> 75B 0.11473<br>72B -> 77B 0.40639<br>73B -> 80B -0.10888<br>73B -> 85B -0.23370	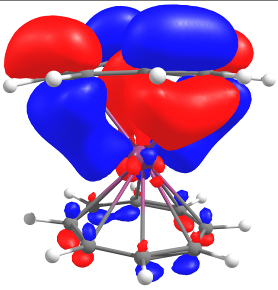<br>72A	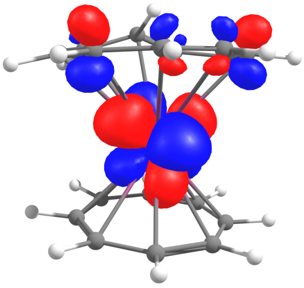<br>80A
Excited State 60: 2.425-?Sym<br>4.5477 eV **272.63** nm<br>f=0.0346 <S**2>=1.220<br>71A -> 76A -0.18540<br>71A -> 78A -0.16972<br>71A -> 80A -0.33073<br>**72A -> 77A** 0.42156<br>72A -> 82A 0.23841<br>72A -> 83A -0.15227<br>71B -> 75B -0.21729<br>71B -> 77B -0.37649<br>72B -> 76B 0.39699<br>74B -> 80B 0.14323<br>74B -> 85B 0.34449	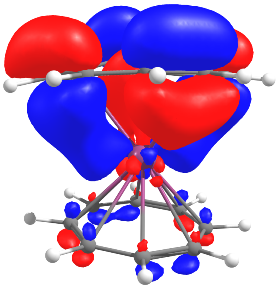<br>72A	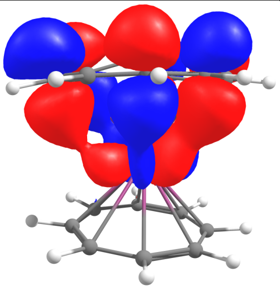<br>77A
Excited State 61: 2.768-?Sym<br>4.5654 eV **271.57** nm<br>f=0.0055 <S**2>=1.665<br>71A -> 77A -0.22476<br>71A -> 79A 0.27958<br>71A -> 82A -0.12322<br>**72A -> 80A** 0.63813<br>73A -> 84A -0.23945<br>73A -> 85A -0.23815<br>71B -> 76B -0.18554<br>72B -> 75B -0.10761<br>72B -> 77B -0.39637<br>73B -> 85B 0.22892	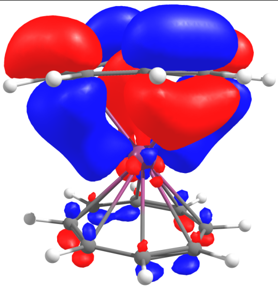<br>72A	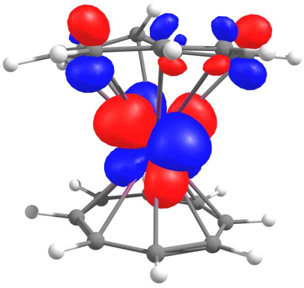<br>80A

Excited State 63: 2.834-?Sym<br>4.6175 eV **268.51** nm<br>f=0.0127 <S**2>=1.758<br>71A -> 77A -0.21368<br>**71A -> 79A** 0.56896<br>71A -> 83A 0.10882<br>72A -> 76A -0.12307<br>72A -> 78A -0.21156<br>72A -> 81A -0.49393<br>74A -> 87A 0.11144<br>71B -> 76B -0.30253<br>72B -> 77B 0.22558<br>73B -> 85B -0.23440	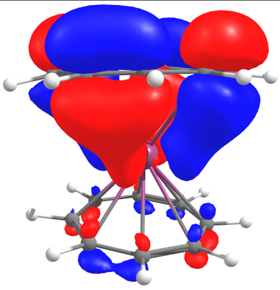<br>71A	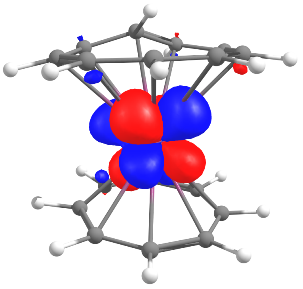<br>79A
Excited State 65: 2.843-?Sym<br>4.6787 eV **265.00** nm<br>f=0.0179 <S**2>=1.771<br>71A -> 77A 0.17300<br>71A -> 79A 0.42721<br>71A -> 82A 0.17033<br>72A -> 80A -0.26624<br>73A -> 84A -0.16121<br>73A -> 85A -0.12416<br>73A -> 86A 0.22598<br>73A -> 87A -0.19960<br>74A -> 86A -0.17140<br>74A -> 87A -0.18039<br>75A -> 88A -0.10628<br>71B -> 76B 0.12455<br>71B -> 83B -0.11432<br>72B -> 82B 0.10991<br>**73B -> 85B** 0.49742<br>73B -> 86B 0.10086<br>74B -> 83B -0.12036<br>74B -> 87B 0.15151	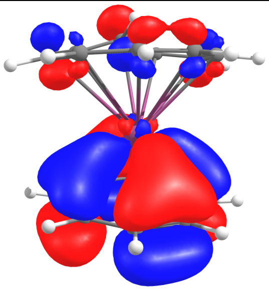<br>73B	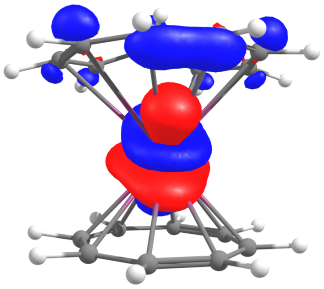<br>85B
Excited State 66: 2.789-?Sym<br>4.6815 eV **264.84** nm<br>f=0.0160 <S**2>=1.694<br>71A -> 76A 0.10116<br>71A -> 78A 0.14864<br>71A -> 80A -0.40270<br>71A -> 81A 0.16417<br>72A -> 77A -0.17910<br>72A -> 82A -0.28513<br>73A -> 89A 0.31989<br>75A -> 86A -0.18892<br>75A -> 87A -0.21133<br>71B -> 77B 0.12272<br>72B -> 76B -0.12434<br>73B -> 87B -0.13524<br>74B -> 78B 0.10169<br>**74B -> 85B** 0.56194	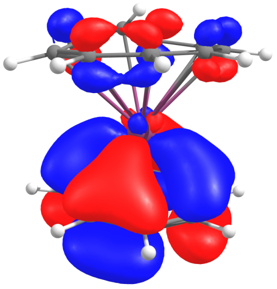<br>74B	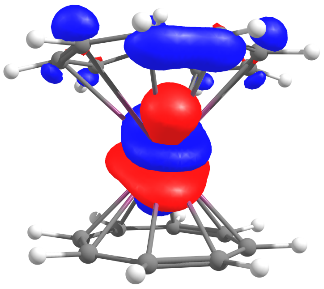<br>85B

74B -> 86B 0.13926		
Excited State 67: 3.099-?Sym<br>4.7157 eV **262.92** nm<br>f=0.0110 <S**2>=2.150<br>71A -> 79A 0.39050<br>72A -> 80A -0.19410<br>**72A -> 81A** 0.47865<br>74A -> 86A 0.10037<br>74A -> 87A 0.16787<br>75A -> 88A 0.11893<br>71B -> 76B 0.13974<br>71B -> 83B -0.10546<br>72B -> 77B -0.36396<br>72B -> 82B 0.15742<br>73B -> 82B 0.10326<br>73B -> 85B -0.42261<br>73B -> 86B -0.10729<br>74B -> 87B -0.11350	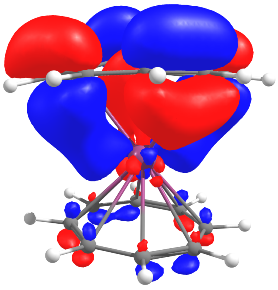<br>72A	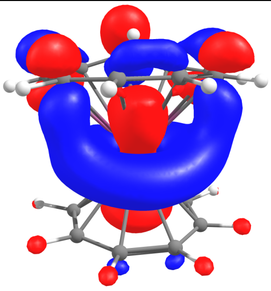<br>81A
Excited State 74: 2.946-?Sym<br>4.9452 eV **250.71** nm<br>f=0.0078 <S**2>=1.919<br>71A -> 77A 0.12711<br>71A -> 83A -0.20567<br>72A -> 81A -0.35504<br>72A -> 84A 0.24139<br>72A -> 85A -0.20245<br>73A -> 86A -0.24463<br>73A -> 87A 0.13450<br>**75A -> 88A** 0.43686<br>72B -> 77B -0.26775<br>72B -> 78B 0.22056<br>72B -> 80B -0.14672<br>73B -> 85B 0.23498<br>74B -> 87B -0.38785	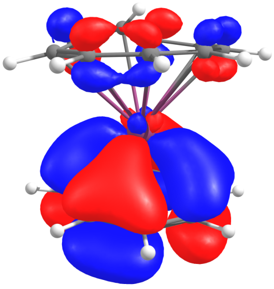<br>75A	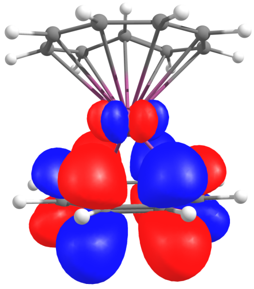<br>88A
Excited State 77: 2.913-?Sym<br>5.0285 eV **246.56** nm<br>f=0.0088 <S**2>=1.871<br>71A -> 81A 0.39119<br>71A -> 84A -0.18908<br>71A -> 85A 0.15562<br>73A -> 89A 0.11432<br>73A -> 91A -0.19933<br>74A -> 88A 0.21505<br>75A -> 86A 0.14555<br>75A -> 87A 0.19361<br>71B -> 77B 0.31975<br>71B -> 78B -0.30119<br>71B -> 80B 0.18254<br>72B -> 76B 0.12686<br>**72B -> 79B** 0.48572<br>72B -> 81B -0.11330	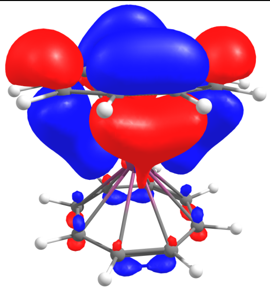<br>72B	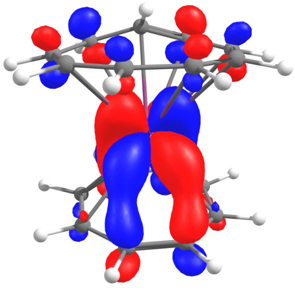<br>79B

73B -> 87B -0.20114<br>74B -> 86B -0.12657<br>74B -> 88B -0.14334		
Excited State 88: 2.841-?Sym<br>5.2006 eV **238.40** nm<br>f=0.0102 <S**2>=1.768<br>71A -> 82A 0.14948<br>71A -> 83A -0.18155<br>72A -> 85A -0.11378<br>73A -> 90A 0.29129<br>73A -> 92A 0.18877<br>71B -> 76B -0.13254<br>71B -> 79B 0.18970<br>71B -> 81B 0.49600<br>71B -> 83B 0.12227<br>72B -> 77B 0.11810<br>**72B -> 82B** 0.59280<br>72B -> 84B 0.17147	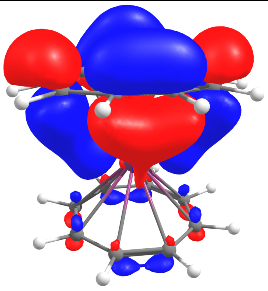 72B	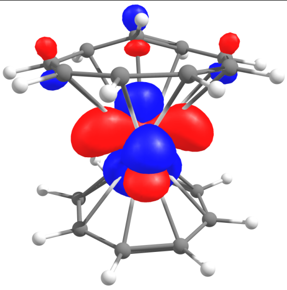 82B
Excited State 96: 2.704-?Sym<br>5.3611 eV **231.27** nm<br>f=0.0070 <S**2>=1.578<br>72A -> 82A -0.10185<br>71B -> 78B -0.10818<br>71B -> 80B -0.36256<br>71B -> 82B 0.38810<br>**71B -> 84B** 0.78815<br>72B -> 79B -0.10451<br>72B -> 81B 0.10245	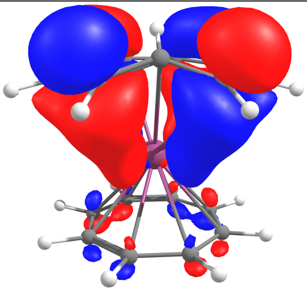 71B	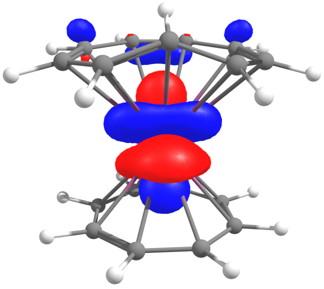 84B
Excited State 97: 3.182-?Sym<br>5.4300 eV **228.33** nm<br>f=0.0094 <S**2>=2.281<br>72A -> 84A -0.24022<br>72A -> 85A -0.31392<br>71B -> 81B -0.14804<br>71B -> 83B -0.34128<br>72B -> 78B 0.39596<br>**72B -> 80B** 0.64956<br>72B -> 84B 0.26654	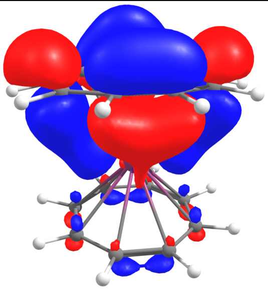 72B	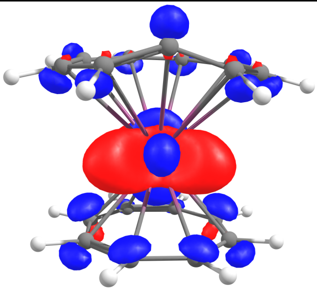 80B
Excited State 99: 2.570-?Sym<br>5.4607 eV **227.05** nm<br>f=0.0341 <S**2>=1.401<br>71A -> 82A 0.10059<br>**72A -> 84A** 0.53125<br>**72A -> 85A** 0.51786<br>71B -> 83B -0.52455<br>72B -> 80B 0.11405<br>72B -> 82B 0.21782<br>72B -> 84B -0.11946	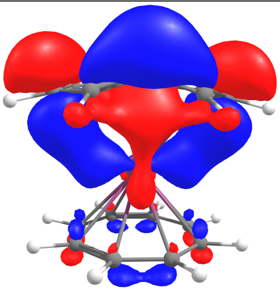 72A	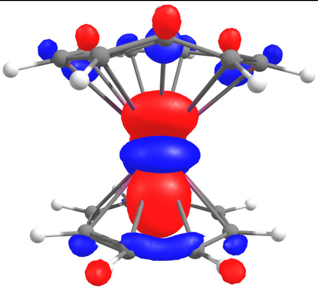 84A

		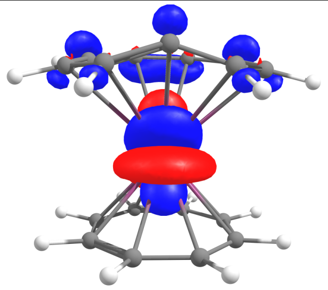<br>85A
Excited State 100: 2.165-?Sym<br>5.4986 eV **225.48** nm<br>f=0.0320 <S**2>=0.922<br>71A -> 82A -0.11971<br>72A -> 84A 0.28117<br>**72A -> 85A** 0.40535<br>73A -> 86A -0.10891<br>73A -> 87A -0.16459<br>71B -> 81B 0.11677<br>**71B -> 83B** 0.40901<br>72B -> 78B 0.19893<br>72B -> 80B 0.35291<br>72B -> 82B -0.25174<br>72B -> 84B 0.39366<br>73B -> 86B 0.16818<br>74B -> 89B 0.16732	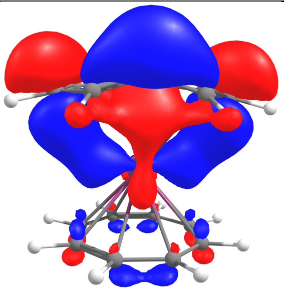<br>72A<br>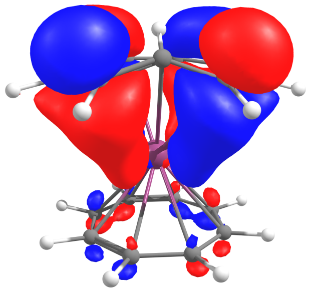<br>71B	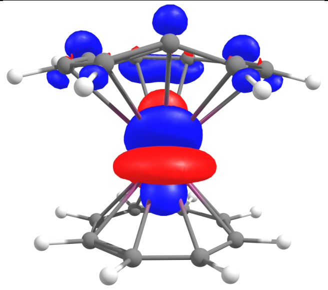<br>85A<br>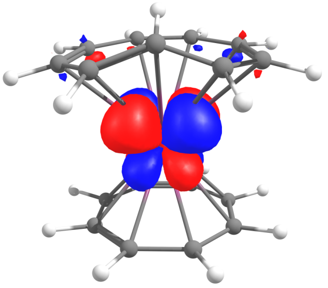<br>83B

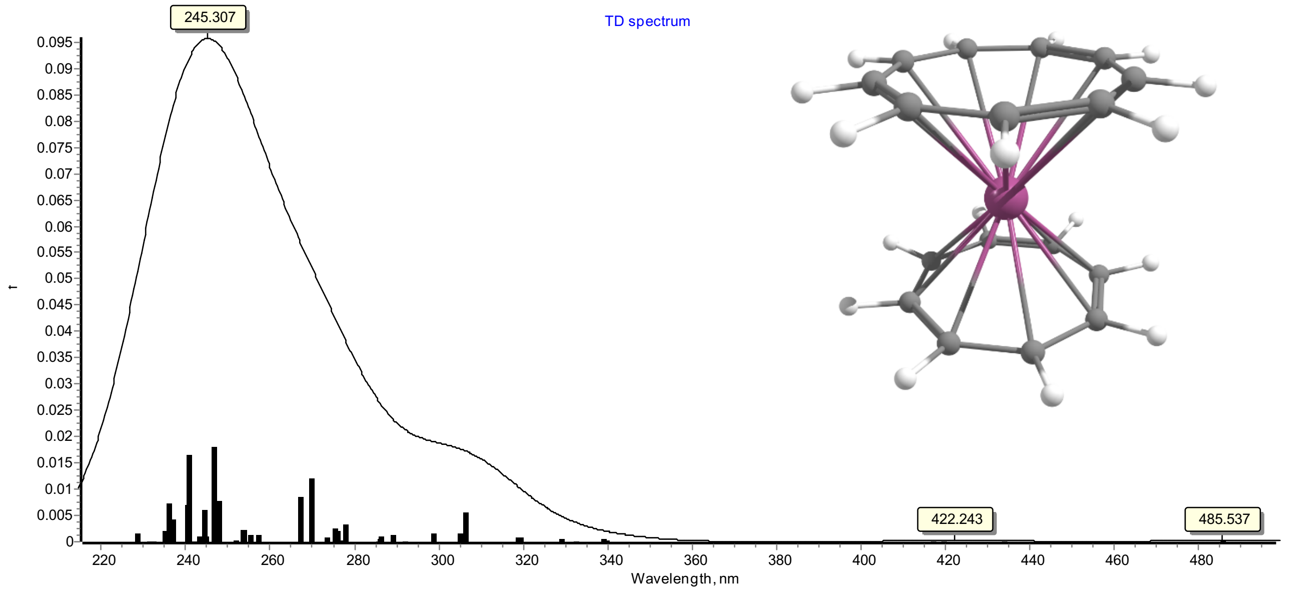

Figure S95**: Computed TD spectrum for **2-Ce-trans

75A and 74B: HOMO		
Excited State 43: 2.633-?Sym<br>4.0495 eV 306.17 nm<br>f=0.0049 <S**2>=1.483<br>72A -> 77A -0.29714<br>74A -> 77A -0.24321<br>74A -> 79A 0.17003<br>**74A -> 83A 0.62727**<br>71B -> 75B 0.20132<br>72B -> 76B 0.18002<br>73B -> 82B 0.27399<br>74B -> 76B 0.16439<br>74B -> 81B -0.45996<br>74B -> 85B -0.12250	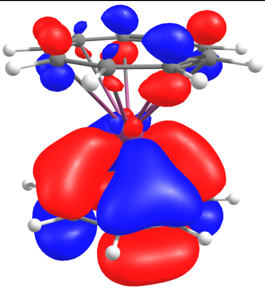<br>74A	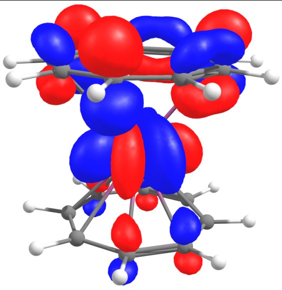<br>83A
Excited State 44: 2.315-?Sym<br>4.0508 eV 306.08 nm<br>f=0.0054 <S**2>=1.089<br>71A -> 77A -0.23997<br>72A -> 76A -0.30091<br>74A -> 81A 0.21539<br>75A -> 77A -0.20968<br>75A -> 79A 0.15162<br>**75A -> 83A 0.62482**<br>72B -> 75B 0.17370<br>73B -> 81B 0.17562<br>74B -> 75B -0.10517<br>74B -> 82B 0.49663	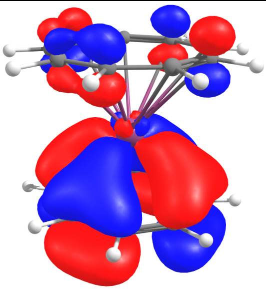<br>75A	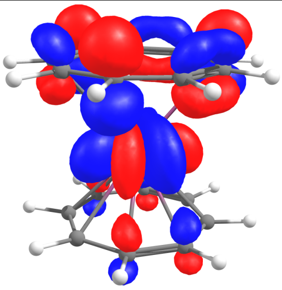<br>83A
Excited State 58: 2.771-?Sym<br>4.4635 eV 277.77 nm<br>f=0.0032 <S**2>=1.670<br>71A -> 76A -0.27219<br>71A -> 81A -0.22225<br>72A -> 77A 0.21385<br>**72A -> 79A 0.83361**<br>72B -> 76B -0.19728<br>74B -> 85B 0.24478	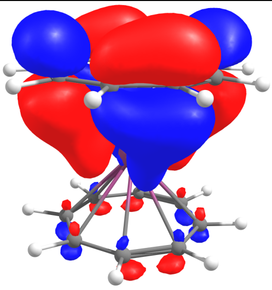<br>72A	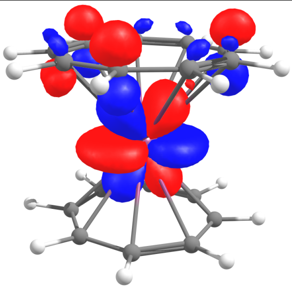<br>79A
Excited State 67: 2.678-?Sym<br>4.6418 eV 267.11 nm<br>f=0.0084 <S**2>=1.543<br>71A -> 77A 0.28870<br>71A -> 79A -0.30547<br>71A -> 83A 0.13573<br>72A -> 76A 0.18387<br>**72A -> 81A 0.74747**<br>72B -> 75B 0.40823	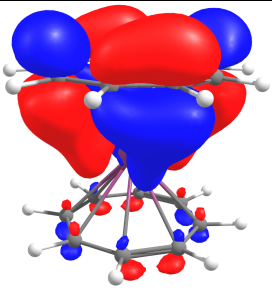<br>72A	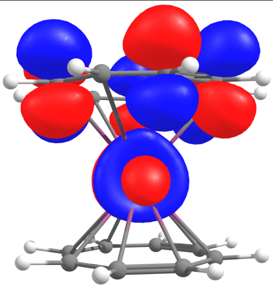<br>81A

Excited State 68: 2.634-?Sym<br>4.6427 eV 267.05 nm<br>f=0.0084 <S**2>=1.485<br>71A -> 76A 0.16550<br>**71A -> 81A 0.70196**<br>72A -> 77A -0.26998<br>72A -> 79A 0.37840<br>72A -> 83A -0.13882<br>73A -> 87A -0.16094<br>71B -> 75B -0.41436	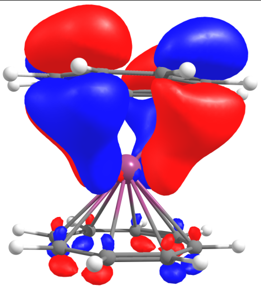<br>71A	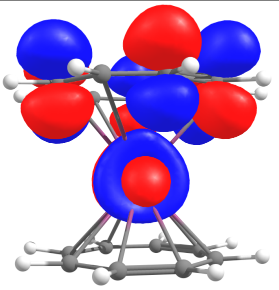<br>81A
Excited State 75: 2.815-?Sym<br>5.0087 eV 247.54 nm<br>f=0.0078 <S**2>=1.731<br>71A -> 77A 0.15962<br>**71A -> 83A 0.58867**<br>72A -> 81A -0.15237<br>72A -> 84A -0.33932<br>73A -> 89A -0.42714<br>73A -> 91A 0.12270<br>75A -> 88A 0.26879<br>71B -> 76B 0.20231<br>71B -> 81B 0.18133<br>72B -> 79B -0.10894<br>74B -> 86B -0.21416	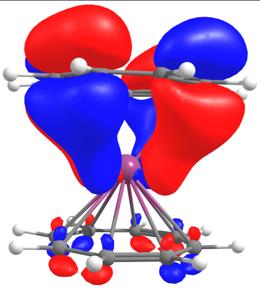<br>71A	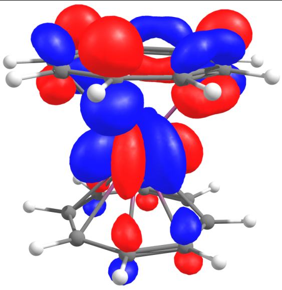<br>83A
Excited State 76: 3.021-?Sym<br>5.0103 eV 247.46 nm<br>f=0.0073 <S**2>=2.032<br>71A -> 81A 0.15811<br>71A -> 84A -0.11976<br>72A -> 77A 0.15043<br>**72A -> 83A 0.63991**<br>73A -> 87A -0.35691<br>74A -> 88A 0.37029<br>71B -> 79B 0.12752<br>71B -> 82B -0.11076<br>72B -> 76B -0.18962<br>72B -> 81B -0.17980<br>73B -> 86B -0.30482<br>74B -> 85B 0.12747	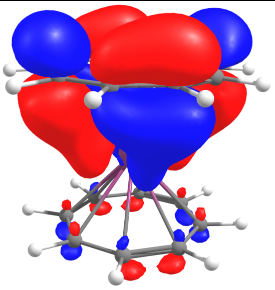<br>72A	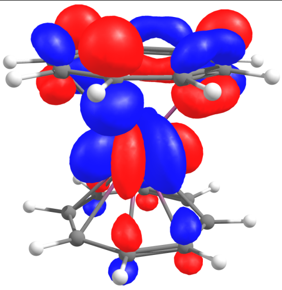<br>83A
Excited State 78: 2.970-?Sym<br>5.0293 eV 246.52 nm<br>f=0.0180 <S**2>=1.955<br>**71A -> 84A 0.76960**<br>72A -> 83A 0.11670<br>73A -> 90A 0.41443<br>73A -> 93A -0.10933<br>75A -> 84A -0.11653<br>75A -> 87A 0.15224<br>71B -> 78B -0.11192<br>71B -> 80B 0.14637<br>71B -> 83B 0.18341	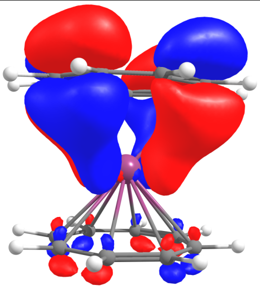<br>71A	84A<br>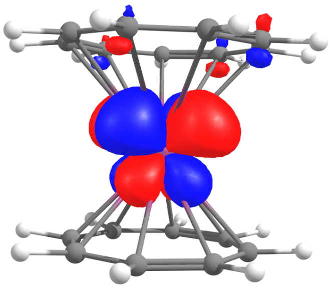

72B -> 84B -0.23758		
Excited State 81: 2.339-?Sym<br>5.0762 eV 244.25 nm<br>f=0.0059 <S**2>=1.118<br>71A -> 84A -0.40126<br>**73A -> 90A 0.81339**<br>73A -> 93A -0.20560<br>73A -> 95A -0.14595<br>74A -> 89A 0.10117<br>71B -> 83B -0.16005<br>72B -> 84B 0.11375<br>73B -> 88B -0.12299	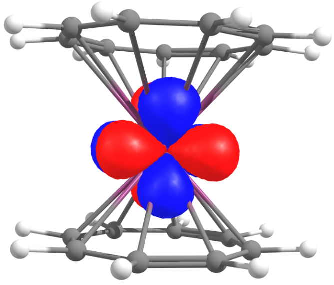<br>73A	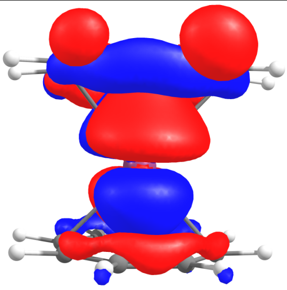<br>90A
Excited State 87: 2.808-?Sym<br>5.1539 eV 240.56 nm<br>f=0.0042 <S**2>=1.721<br>73A -> 90A 0.11700<br>71B -> 77B -0.13279<br>**71B -> 78B 0.74986**<br>71B -> 80B -0.59254<br>72B -> 84B -0.13940	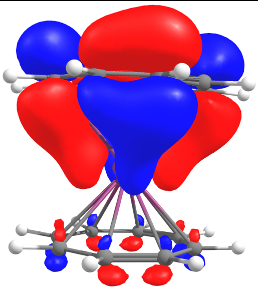<br>71B	78B<br>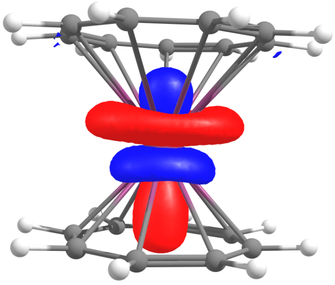
Excited State 88: 3.027-?Sym<br>5.1571 eV 240.42 nm<br>f=0.0165 <S**2>=2.040<br>71A -> 83A -0.26753<br>74A -> 86A -0.19163<br>75A -> 88A -0.10653<br>71B -> 76B 0.16805<br>**71B -> 81B 0.66936**<br>72B -> 75B 0.19575<br>72B -> 82B 0.43583<br>73B -> 87B 0.24384<br>74B -> 86B 0.22114	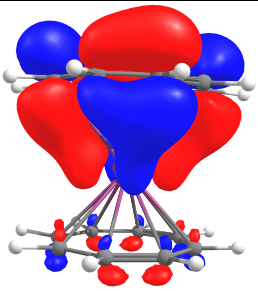<br>71B	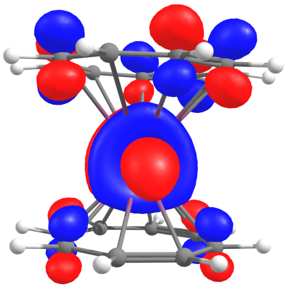<br>81B
Excited State 89: 3.072-?Sym<br>5.1612 eV 240.22 nm<br>f=0.0069 <S**2>=2.109<br>72A -> 83A 0.17318<br>73A -> 87A 0.18538<br>74A -> 89A 0.25753<br>75A -> 86A -0.18742<br>75A -> 87A -0.28876<br>71B -> 75B -0.12636<br>71B -> 79B 0.20357<br>71B -> 82B -0.17738<br>72B -> 76B 0.11320<br>**72B -> 81B 0.60111**<br>73B -> 86B -0.16295<br>73B -> 88B -0.28641<br>74B -> 87B 0.23375<br>74B -> 90B 0.22015	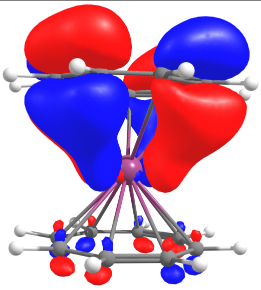<br>72B	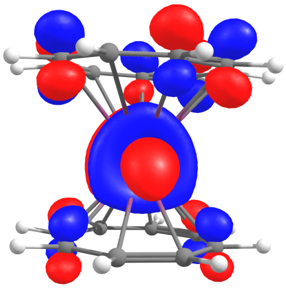<br>81B

Excited State 90: 3.269-?Sym<br>5.1623 eV 240.17 nm<br>f=0.0037 <S**2>=2.422<br>71A -> 84A -0.18428<br>72A -> 83A 0.10007<br>73A -> 87A 0.12533<br>74A -> 89A -0.36718<br>75A -> 86A -0.12673<br>**75A -> 87A 0.43950**<br>71B -> 79B 0.14285<br>71B -> 82B -0.13033<br>72B -> 81B 0.39392<br>73B -> 86B -0.10929<br>**73B -> 88B 0.42412**<br>74B -> 87B 0.15606<br>74B -> 90B -0.32362	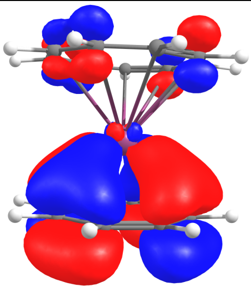<br>75A	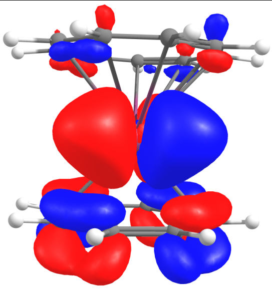<br>87A
Excited State 91: 2.581-?Sym<br>5.2397 eV 236.63 nm<br>f=0.0042 <S**2>=1.416<br>71A -> 83A 0.10781<br>72A -> 81A -0.19861<br>71B -> 81B -0.55304<br>**72B -> 82B 0.75165**<br>72B -> 83B -0.14074	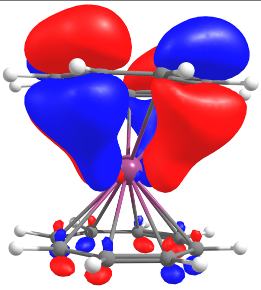<br>72B	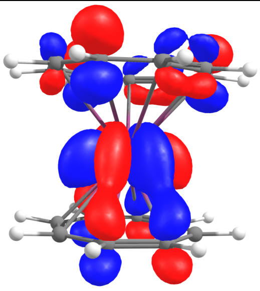<br>82B
Excited State 92: 2.554-?Sym<br>5.2573 eV 235.83 nm<br>f=0.0073 <S**2>=1.380<br>71A -> 81A 0.19729<br>72A -> 83A 0.13430<br>73A -> 87A -0.12713<br>71B -> 75B 0.10071<br>**71B -> 82B 0.81990**<br>72B -> 76B -0.10215<br>72B -> 81B 0.40081<br>72B -> 84B 0.10540<br>73B -> 86B 0.10404	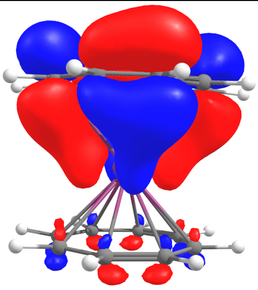<br>71B	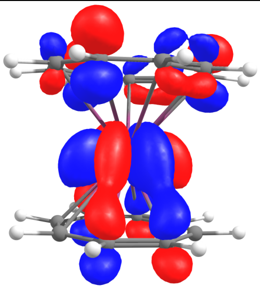<br>82B

Optimized structure

3-disp

Ce	4.577791000	3.235332000	4.478832000
C	1.912585000	4.517356000	4.107971000
H	1.496519000	4.775668000	5.079991000
C	2.717308000	5.427973000	3.407535000
H	2.740962000	6.463965000	3.737212000
C	3.605211000	5.113529000	2.350205000
H	4.097291000	6.002171000	1.960703000
C	4.113146000	3.951845000	1.698845000
H	4.875098000	4.274114000	0.990636000
C	1.871955000	3.235404000	3.563154000
C	4.555302000	3.942013000	7.044089000
H	3.740918000	4.358746000	7.631978000
C	5.315568000	4.936055000	6.391385000
H	4.937522000	5.937230000	6.584552000
C	6.378318000	4.936268000	5.461183000
H	6.623086000	5.937809000	5.115482000
C	7.127827000	3.941664000	4.796894000
H	7.817873000	4.358558000	4.067139000
H	2.132183000	3.235374000	2.509816000
C	1.912499000	1.953469000	4.108021000
H	1.496415000	1.695225000	5.080051000
C	2.717162000	1.042771000	3.407623000
H	2.740746000	0.006790000	3.737343000
C	3.605088000	1.357111000	2.350282000
H	4.097111000	0.468421000	1.960816000
C	4.113099000	2.518736000	1.698875000
H	4.875030000	2.196388000	0.990679000
C	4.555361000	2.528539000	7.044061000
H	3.741011000	2.111715000	7.631931000
C	5.315706000	1.534586000	6.391314000
H	4.937741000	0.533372000	6.584440000
C	6.378453000	1.534497000	5.461109000
H	6.623302000	0.532989000	5.115367000
C	7.127882000	2.529189000	4.796862000
H	7.817963000	2.112383000	4.067089000

4-disp

Ce	4.507571000	3.235360000	4.616365000
C	1.496798000	3.235352000	4.546377000
H	0.792927000	3.235354000	5.376205000
C	1.830006000	4.554977000	4.200999000
H	1.300531000	5.252807000	4.846498000
C	2.676817000	5.256802000	3.328305000
H	2.594161000	6.326356000	3.511021000
C	3.643153000	5.013074000	2.339094000
H	4.072176000	5.953513000	1.999111000
C	4.276734000	3.937579000	1.696636000
H	5.042929000	4.308786000	1.019030000
C	1.830001000	1.915727000	4.201001000
H	1.300522000	1.217897000	4.846498000
C	2.676827000	1.213898000	3.328322000

H	2.594178000	0.144346000	3.511048000
C	3.643165000	1.457623000	2.339113000
H	4.072201000	0.517184000	1.999147000
C	4.276739000	2.533116000	1.696645000
H	5.042942000	2.161907000	1.019049000
C	4.692375000	3.941635000	7.169573000
H	3.921039000	4.355764000	7.814281000
C	5.390067000	4.940011000	6.455660000
H	5.028208000	5.939996000	6.682330000
C	6.372947000	4.939991000	5.441921000
H	6.588336000	5.939979000	5.073254000
C	7.064966000	3.941646000	4.722509000
H	7.685411000	4.355737000	3.931525000
C	4.692362000	2.529056000	7.169561000
H	3.921023000	2.114928000	7.814265000
C	5.390046000	1.530687000	6.455630000
H	5.028172000	0.530701000	6.682277000
C	6.372945000	1.530710000	5.441912000
H	6.588340000	0.530724000	5.073241000
C	7.064966000	2.529059000	4.722508000
H	7.685408000	2.114970000	3.931521000

1-disp, s=5/2

Sm	4.438188000	3.235350000	4.562342000
C	1.548011000	3.235350000	4.596266000
H	0.843057000	3.235350000	5.424458000
C	1.883544000	4.553679000	4.251770000
H	1.360590000	5.251328000	4.901905000
C	2.736738000	5.254179000	3.386372000
H	2.662608000	6.322222000	3.578137000
C	3.703665000	5.012580000	2.397250000
H	4.138250000	5.952484000	2.064873000
C	4.338509000	3.937860000	1.760429000
H	5.113747000	4.307335000	1.093020000
C	4.634957000	3.945213000	7.106222000
H	3.868815000	4.355599000	7.759658000
C	5.322257000	4.937927000	6.394265000
H	4.972179000	5.940264000	6.627755000
C	6.304443000	4.934782000	5.369759000
H	6.514965000	5.935930000	5.001155000
C	6.991631000	3.944895000	4.652431000
H	7.616968000	4.357486000	3.864682000
C	1.883545000	1.917021000	4.251770000
H	1.360591000	1.219372000	4.901905000
C	2.736738000	1.216521000	3.386373000
H	2.662608000	0.148478000	3.578138000
C	3.703665000	1.458120000	2.397250000
H	4.138251000	0.518216000	2.064873000
C	4.338509000	2.532841000	1.760429000
H	5.113747000	2.163365000	1.093020000
C	4.634957000	2.525487000	7.106221000
H	3.868814000	2.115102000	7.759657000
C	5.322257000	1.532772000	6.394264000
H	4.972179000	0.530436000	6.627754000
C	6.304442000	1.535917000	5.369759000
H	6.514965000	0.534769000	5.001155000

C	6.991631000	2.525805000	4.652431000
H	7.616967000	2.113215000	3.864681000

1-disp, s=3/2

Sm	4.456149000	3.235350000	4.564075000
C	1.578302000	3.235350000	4.622477000
H	0.885541000	3.235350000	5.460799000
C	1.911292000	4.553937000	4.278457000
H	1.392658000	5.250364000	4.933187000
C	2.759468000	5.255200000	3.409115000
H	2.689963000	6.322903000	3.603522000
C	3.724995000	5.010523000	2.421297000
H	4.167574000	5.949120000	2.095914000
C	4.353925000	3.936597000	1.773978000
H	5.126751000	4.307865000	1.105014000
C	4.602677000	3.940389000	7.080479000
H	3.828343000	4.355044000	7.721144000
C	5.297475000	4.936944000	6.363382000
H	4.926826000	5.935798000	6.580633000
C	6.280999000	4.938814000	5.352447000
H	6.492017000	5.938380000	4.980705000
C	6.974173000	3.940754000	4.636927000
H	7.589361000	4.353955000	3.841335000
C	1.911293000	1.916763000	4.278457000
H	1.392659000	1.220336000	4.933188000
C	2.759468000	1.215499000	3.409115000
H	2.689963000	0.147797000	3.603522000
C	3.724995000	1.460177000	2.421297000
H	4.167574000	0.521580000	2.095914000
C	4.353925000	2.534103000	1.773979000
H	5.126751000	2.162835000	1.105014000
C	4.602677000	2.530310000	7.080479000
H	3.828344000	2.115656000	7.721145000
C	5.297475000	1.533756000	6.363382000
H	4.926826000	0.534902000	6.580633000
C	6.280999000	1.531886000	5.352447000
H	6.492016000	0.532320000	4.980705000
C	6.974173000	2.529946000	4.636927000
H	7.589361000	2.116745000	3.841336000

1-disp, s=1/2

Sm	4.461367000	3.235322000	4.568253000
C	1.584855000	3.235605000	4.629785000
H	0.892181000	3.235633000	5.468081000
C	1.918844000	4.553757000	4.285304000
H	1.402653000	5.250329000	4.941817000
C	2.766551000	5.255183000	3.415926000
H	2.698157000	6.322641000	3.611893000
C	3.730761000	5.011279000	2.426311000
H	4.172552000	5.949940000	2.100182000
C	4.360435000	3.937216000	1.781496000
H	5.135397000	4.307763000	1.114633000
C	4.594706000	3.940873000	7.071830000
H	3.816186000	4.354446000	7.708047000
C	5.291498000	4.937562000	6.358711000
H	4.919649000	5.936312000	6.574088000
C	6.271377000	4.937725000	5.344191000
H	6.476942000	5.936406000	4.967081000
C	6.968750000	3.939784000	4.630516000

H	7.581613000	4.353588000	3.833520000
C	1.918729000	1.917483000	4.285309000
H	1.402572000	1.220870000	4.941792000
C	2.767023000	1.216209000	3.416363000
H	2.699391000	0.148832000	3.612980000
C	3.731170000	1.460060000	2.426606000
H	4.173368000	0.521401000	2.101047000
C	4.360451000	2.534001000	1.781301000
H	5.135345000	2.163521000	1.114336000
C	4.594702000	2.529341000	7.071571000
H	3.815921000	2.115742000	7.707491000
C	5.291672000	1.532417000	6.358886000
H	4.920403000	0.533621000	6.575017000
C	6.271873000	1.532267000	5.344588000
H	6.478450000	0.533483000	4.968285000
C	6.969235000	2.530247000	4.631053000
H	7.582208000	2.116393000	3.834142000

1, s=5/2

Sm	4.491774000	3.235350000	4.447602000
C	1.917403000	4.513888000	4.111436000
H	1.487309000	4.775311000	5.076825000
C	2.738128000	5.422144000	3.424451000
H	2.755905000	6.458661000	3.754010000
C	3.645168000	5.109651000	2.385768000
H	4.141414000	6.000259000	2.005501000
C	4.166307000	3.950126000	1.736465000
H	4.935030000	4.276202000	1.037391000
C	1.866671000	3.235389000	3.560381000
C	4.546258000	3.935424000	7.039686000
H	3.760293000	4.357422000	7.662622000
C	5.300736000	4.938903000	6.366778000
H	4.943018000	5.940953000	6.593983000
C	6.333367000	4.938629000	5.422714000
H	6.591324000	5.940600000	5.086344000
C	7.072833000	3.935047000	4.733978000
H	7.761287000	4.357077000	4.004925000
H	2.141027000	3.235362000	2.509725000
C	1.917347000	1.956910000	4.111487000
H	1.487242000	1.695544000	5.076887000
C	2.738032000	1.048590000	3.424539000
H	2.755762000	0.012085000	3.754139000
C	3.645086000	1.361000000	2.385844000
H	4.141293000	0.470355000	2.005613000
C	4.166277000	2.520477000	1.736495000
H	4.934984000	2.194338000	1.037434000
C	4.546295000	2.535159000	7.039653000
H	3.760352000	2.113091000	7.662569000
C	5.300824000	1.531749000	6.366699000
H	4.943156000	0.529671000	6.593856000
C	6.333455000	1.532120000	5.422634000
H	6.591462000	0.530178000	5.086216000
C	7.072868000	2.535772000	4.733943000
H	7.761343000	2.113812000	4.004870000

1, s=3/2

Sm	4.528968000	3.235341000	4.455653000
C	1.954971000	4.515628000	4.154902000
H	1.527121000	4.776723000	5.120865000

C	2.773766000	5.421408000	3.467097000
H	2.801607000	6.455794000	3.801697000
C	3.673013000	5.107856000	2.418961000
H	4.173358000	5.997574000	2.042465000
C	4.183827000	3.951463000	1.761931000
H	4.950422000	4.276383000	1.060237000
C	1.910339000	3.235388000	3.605765000
C	4.502954000	3.941342000	6.990042000
H	3.698844000	4.359944000	7.591356000
C	5.261368000	4.934484000	6.331531000
H	4.888183000	5.936542000	6.532615000
C	6.312492000	4.937129000	5.389987000
H	6.558621000	5.939766000	5.047641000
C	7.049227000	3.941372000	4.711996000
H	7.728362000	4.357979000	3.971261000
H	2.175447000	3.235365000	2.552848000
C	1.954911000	1.955163000	4.154944000
H	1.527048000	1.694120000	5.120915000
C	2.773665000	1.049323000	3.467170000
H	2.801457000	0.014947000	3.801804000
C	3.672928000	1.362799000	2.419024000
H	4.173231000	0.473046000	2.042556000
C	4.183793000	2.519147000	1.761955000
H	4.950372000	2.194168000	1.060270000
C	4.502994000	2.529249000	6.990017000
H	3.698906000	2.110580000	7.591314000
C	5.261464000	1.536172000	6.331470000
H	4.888334000	0.534087000	6.532521000
C	6.312586000	1.533619000	5.389925000
H	6.558770000	0.531007000	5.047543000
C	7.049264000	2.529442000	4.711969000
H	7.728421000	2.112900000	3.971217000

1, s=1/2

Sm	4.518720000	3.240110000	4.479438000
C	1.949900000	4.510694000	4.143827000
H	1.514939000	4.766421000	5.108308000
C	2.768591000	5.424029000	3.463346000
H	2.785041000	6.459450000	3.795508000
C	3.679685000	5.111405000	2.427918000
H	4.182779000	6.000309000	2.053210000
C	4.201258000	3.951220000	1.782298000
H	4.981485000	4.275212000	1.095313000
C	1.909637000	3.233346000	3.589768000
C	4.517610000	3.943884000	7.007732000
H	3.721400000	4.364235000	7.618113000
C	5.265774000	4.936224000	6.332328000
H	4.896787000	5.938822000	6.537601000
C	6.310556000	4.937241000	5.383357000
H	6.552010000	5.939458000	5.036657000
C	7.034511000	3.940928000	4.691745000
H	7.702415000	4.358138000	3.941259000
H	2.180260000	3.233704000	2.538447000
C	1.948490000	1.952518000	4.141865000
H	1.505730000	1.692698000	5.101613000
C	2.769451000	1.043799000	3.462470000
H	2.789597000	0.008367000	3.794447000
C	3.683625000	1.359757000	2.428430000
H	4.188289000	0.471615000	2.054077000

C	4.200338000	2.519495000	1.780416000
H	4.980616000	2.196633000	1.092916000
C	4.512755000	2.533445000	7.002300000
H	3.715370000	2.114643000	7.612282000
C	5.264122000	1.537038000	6.333560000
H	4.892089000	0.535743000	6.539398000
C	6.301938000	1.534858000	5.380045000
H	6.537745000	0.532388000	5.029690000
C	7.029990000	2.528893000	4.686762000
H	7.697526000	2.110530000	3.937023000

2-disp, s=5/2

Sm	4.438188000	3.235350000	4.562342000
C	1.548011000	3.235350000	4.596266000
H	0.843057000	3.235350000	5.424458000
C	1.883544000	4.553679000	4.251770000
H	1.360590000	5.251328000	4.901905000
C	2.736738000	5.254179000	3.386372000
H	2.662608000	6.322222000	3.578137000
C	3.703665000	5.012580000	2.397250000
H	4.138250000	5.952484000	2.064873000
C	4.338509000	3.937860000	1.760429000
H	5.113747000	4.307335000	1.093020000
C	4.634957000	3.945213000	7.106222000
H	3.868815000	4.355599000	7.759658000
C	5.322257000	4.937927000	6.394265000
H	4.972179000	5.940264000	6.627755000
C	6.304443000	4.934782000	5.369759000
H	6.514965000	5.935930000	5.001155000
C	6.991631000	3.944895000	4.652431000
H	7.616968000	4.357486000	3.864682000
C	1.883545000	1.917021000	4.251770000
H	1.360591000	1.219372000	4.901905000
C	2.736738000	1.216521000	3.386373000
H	2.662608000	0.148478000	3.578138000
C	3.703665000	1.458120000	2.397250000
H	4.138251000	0.518216000	2.064873000
C	4.338509000	2.532841000	1.760429000
H	5.113747000	2.163365000	1.093020000
C	4.634957000	2.525487000	7.106221000
H	3.868814000	2.115102000	7.759657000
C	5.322257000	1.532772000	6.394264000
H	4.972179000	0.530436000	6.627754000
C	6.304442000	1.535917000	5.369759000
H	6.514965000	0.534769000	5.001155000
C	6.991631000	2.525805000	4.652431000
H	7.616967000	2.113215000	3.864681000

2-disp, s=3/2

Sm	4.456149000	3.235350000	4.564075000
C	1.578302000	3.235350000	4.622477000
H	0.885541000	3.235350000	5.460799000
C	1.911292000	4.553937000	4.278457000
H	1.392658000	5.250364000	4.933187000
C	2.759468000	5.255200000	3.409115000
H	2.689963000	6.322903000	3.603522000
C	3.724995000	5.010523000	2.421297000
H	4.167574000	5.949120000	2.095914000
C	4.353925000	3.936597000	1.773978000

H	5.126751000	4.307865000	1.105014000
C	4.602677000	3.940389000	7.080479000
H	3.828343000	4.355044000	7.721144000
C	5.297475000	4.936944000	6.363382000
H	4.926826000	5.935798000	6.580633000
C	6.280999000	4.938814000	5.352447000
H	6.492017000	5.938380000	4.980705000
C	6.974173000	3.940754000	4.636927000
H	7.589361000	4.353955000	3.841335000
C	1.911293000	1.916763000	4.278457000
H	1.392659000	1.220336000	4.933188000
C	2.759468000	1.215499000	3.409115000
H	2.689963000	0.147797000	3.603522000
C	3.724995000	1.460177000	2.421297000
H	4.167574000	0.521580000	2.095914000
C	4.353925000	2.534103000	1.773979000
H	5.126751000	2.162835000	1.105014000
C	4.602677000	2.530310000	7.080479000
H	3.828344000	2.115656000	7.721145000
C	5.297475000	1.533756000	6.363382000
H	4.926826000	0.534902000	6.580633000
C	6.280999000	1.531886000	5.352447000
H	6.492016000	0.532320000	4.980705000
C	6.974173000	2.529946000	4.636927000
H	7.589361000	2.116745000	3.841336000

2-disp, s=1/2

Sm	4.461367000	3.235322000	4.568253000
C	1.584855000	3.235605000	4.629785000
H	0.892181000	3.235633000	5.468081000
C	1.918844000	4.553757000	4.285304000
H	1.402653000	5.250329000	4.941817000
C	2.766551000	5.255183000	3.415926000
H	2.698157000	6.322641000	3.611893000
C	3.730761000	5.011279000	2.426311000
H	4.172552000	5.949940000	2.100182000
C	4.360435000	3.937216000	1.781496000
H	5.135397000	4.307763000	1.114633000
C	4.594706000	3.940873000	7.071830000
H	3.816186000	4.354446000	7.708047000
C	5.291498000	4.937562000	6.358711000
H	4.919649000	5.936312000	6.574088000
C	6.271377000	4.937725000	5.344191000
H	6.476942000	5.936406000	4.967081000
C	6.968750000	3.939784000	4.630516000
H	7.581613000	4.353588000	3.833520000
C	1.918729000	1.917483000	4.285309000
H	1.402572000	1.220870000	4.941792000
C	2.767023000	1.216209000	3.416363000
H	2.699391000	0.148832000	3.612980000
C	3.731170000	1.460060000	2.426606000
H	4.173368000	0.521401000	2.101047000
C	4.360451000	2.534001000	1.781301000
H	5.135345000	2.163521000	1.114336000
C	4.594702000	2.529341000	7.071571000
H	3.815921000	2.115742000	7.707491000
C	5.291672000	1.532417000	6.358886000
H	4.920403000	0.533621000	6.575017000
C	6.271873000	1.532267000	5.344588000

H	6.478450000	0.533483000	4.968285000
C	6.969235000	2.530247000	4.631053000
H	7.582208000	2.116393000	3.834142000

2, s=5/2

Sm	4.438216000	3.235347000	4.556735000
C	1.529510000	3.235352000	4.580234000
H	0.819198000	3.235353000	5.404567000
C	1.864000000	4.554651000	4.234544000
H	1.334469000	5.253075000	4.879363000
C	2.714815000	5.255480000	3.365722000
H	2.632688000	6.324892000	3.549520000
C	3.680388000	5.013573000	2.373878000
H	4.106153000	5.954658000	2.031832000
C	4.314501000	3.938390000	1.735285000
H	5.080678000	4.308388000	1.056920000
C	4.654686000	3.946727000	7.126921000
H	3.895447000	4.357028000	7.789345000
C	5.343162000	4.938578000	6.417265000
H	5.001778000	5.942632000	6.658796000
C	6.330852000	4.935754000	5.393720000
H	6.553109000	5.938918000	5.036023000
C	7.019546000	3.946525000	4.679418000
H	7.658758000	4.358789000	3.901899000
C	1.863995000	1.916052000	4.234544000
H	1.334463000	1.217630000	4.879363000
C	2.714808000	1.215221000	3.365721000
H	2.632676000	0.145809000	3.549519000
C	3.680382000	1.457125000	2.373877000
H	4.106144000	0.516039000	2.031832000
C	4.314498000	2.532306000	1.735285000
H	5.080674000	2.162306000	1.056919000
C	4.654689000	2.523969000	7.126921000
H	3.895453000	2.113664000	7.789346000
C	5.343169000	1.532120000	6.417265000
H	5.001789000	0.528065000	6.658796000
C	6.330859000	1.534949000	5.393720000
H	6.553119000	0.531786000	5.036023000
C	7.019549000	2.524180000	4.679417000
H	7.658764000	2.111919000	3.901899000

2, s=3/2

Sm	4.461632000	3.235350000	4.568566000
C	1.559274000	3.235350000	4.605772000
H	0.858063000	3.235350000	5.437780000
C	1.892361000	4.554794000	4.261499000
H	1.365666000	5.252022000	4.909742000
C	2.740467000	5.256523000	3.391034000
H	2.663084000	6.325558000	3.578104000
C	3.706169000	5.011768000	2.402162000
H	4.141136000	5.951542000	2.068297000
C	4.335190000	3.937041000	1.754301000
H	5.101027000	4.308705000	1.076691000
C	4.623061000	3.940816000	7.101745000
H	3.855152000	4.356035000	7.750639000
C	5.318333000	4.938036000	6.384292000
H	4.954849000	5.938368000	6.609369000
C	6.302789000	4.939914000	5.373032000
H	6.522036000	5.940907000	5.008393000

C	6.996600000	3.941217000	4.657299000
H	7.621088000	4.355002000	3.868558000
C	1.892362000	1.915906000	4.261499000
H	1.365666000	1.218678000	4.909742000
C	2.740467000	1.214177000	3.391034000
H	2.663084000	0.145142000	3.578104000
C	3.706169000	1.458932000	2.402162000
H	4.141136000	0.519158000	2.068297000
C	4.335190000	2.533659000	1.754301000
H	5.101027000	2.161996000	1.076691000
C	4.623061000	2.529884000	7.101745000
H	3.855152000	2.114665000	7.750639000
C	5.318333000	1.532664000	6.384292000
H	4.954849000	0.532332000	6.609368000
C	6.302789000	1.530786000	5.373032000
H	6.522036000	0.529793000	5.008393000
C	6.996600000	2.529483000	4.657299000
H	7.621088000	2.115697000	3.868559000

2, s=1/2

Sm	4.467377000	3.235362000	4.574846000
C	1.564564000	3.235672000	4.611752000
H	0.862617000	3.235797000	5.443062000
C	1.900444000	4.554516000	4.268644000
H	1.376088000	5.252169000	4.918318000
C	2.748407000	5.256247000	3.397497000
H	2.672556000	6.324982000	3.586885000
C	3.712872000	5.012799000	2.407866000
H	4.146957000	5.952574000	2.072996000
C	4.342239000	3.937154000	1.761366000
H	5.109543000	4.308406000	1.085196000
C	4.615959000	3.940068000	7.094756000
H	3.843949000	4.354400000	7.739361000
C	5.309673000	4.938852000	6.376739000
H	4.945682000	5.938964000	6.601005000
C	6.295785000	4.938665000	5.367300000
H	6.508096000	5.938850000	4.996002000
C	6.988525000	3.941249000	4.649254000
H	7.610150000	4.355438000	3.858637000
C	1.900525000	1.917005000	4.269068000
H	1.376440000	1.219325000	4.918922000
C	2.749024000	1.215220000	3.398130000
H	2.673868000	0.146584000	3.588279000
C	3.713032000	1.458534000	2.408226000
H	4.147560000	0.518769000	2.073909000
C	4.342589000	2.534144000	1.761667000
H	5.109922000	2.162850000	1.085578000
C	4.616943000	2.529631000	7.095606000
H	3.844941000	2.115231000	7.740235000
C	5.309809000	1.531035000	6.377018000
H	4.946753000	0.530779000	6.602150000
C	6.296004000	1.531305000	5.367156000
H	6.509157000	0.531084000	4.996480000
C	6.988813000	2.528865000	4.649738000
H	7.610119000	2.114724000	3.858786000

9. References

1 M. Tricoire, L. Münzfeld, J. Moutet, N. Mahieu, L. La Droitte, E. Moreno-Pineda, F. Gendron, J. D. Hilgar, J. D. Rinehart, M. Ruben, B. Le Guennic, O. Cador, P. W. Roesky and G. Nocton, *Chem. – Eur. J.*, 2021, **27**, 13558–13567.

2 K. Mashima, Y. Nakayama, A. Nakamura, N. Kanehisa, Y. Kai and H. Takaya, *J. Organomet. Chem.*, 1994, **473**, 85–91.

3 K. Mashima and H. Takaya, *Tetrahedron Lett.*, 1989, **30**, 3697–3700.

4 M. Xémard, S. Zimmer, M. Cordier, V. Goudy, L. Ricard, C. Clavaguéra and G. Nocton, *J. Am. Chem. Soc.*, 2018, **140**, 14433–14439.

5 A. D. Becke, *J. Chem. Phys.*, 1993, **98**, 5648–5652.

6 M. Dolg, H. Stoll and H. Preuss, *Theor. Chim. Acta*, 1993, **85**, 441–450.

7 M. Dolg, H. Stoll, A. Savin and H. Preuss, *Theor. Chim. Acta*, 1989, **75**, 173–194.

8 R. Ditchfield, W. J. Hehre and J. A. Pople, *J. Chem. Phys.*, 1971, **54**, 724–728.

9 W. J. Hehre, R. Ditchfield and J. A. Pople, *J. Chem. Phys.*, 1972, **56**, 2257–2261.

10 K. O. Hodgson, F. Mares, D. F. Starks and A. Streitwieser, *J. Am. Chem. Soc.*, 1973, **95**, 8650–8658.

11 Citation | Gaussian.com, https://gaussian.com/citation/, (accessed July 9, 2024).

12 K. Wolinski, J. F. Hinton and P. Pulay, *J. Am. Chem. Soc.*, 1990, **112**, 8251–8260.

13 P. von R. Schleyer, C. Maerker, A. Dransfeld, H. Jiao and N. J. R. van Eikema Hommes, *J. Am. Chem. Soc.*, 1996, **118**, 6317–6318.

14 A. Stanger, *J. Org. Chem.*, 2006, **71**, 883–893.

15 K. K. Zborowski, I. Alkorta, J. Elguero and L. M. Proniewicz, *Struct. Chem.*, 2012, **23**, 595–600.
